# Identification of key genes and pathways in endometriosis by integrated expression profiles analysis

**DOI:** 10.7717/peerj.10171

**Published:** 2020-12-07

**Authors:** Ding Cui, Yang Liu, Junyan Ma, Kaiqing Lin, Kaihong Xu, Jun Lin

**Affiliations:** 1 Department of Laboratory, Women’s Hospital, Zhejiang University School of Medicine, Hangzhou, China; 2 Department of Gynecology and Obstetrics, Women’s Hospital, Zhejiang University School of Medicine, Hangzhou, China

**Keywords:** Endometriosis, Expression profiles, Bioinformatical analysis, WGCNA, Hippo signaling pathway

## Abstract

The purpose of this study was to integrate the existing expression profile data on endometriosis (EM)-related tissues in order to identify the differentially expressed genes. In this study, three series of raw expression data were downloaded from GEO database. Differentially expressed genes (DEGs) in three tissue types were screened. GO, KEGG pathway enrichment analysis, core differential genes (CDGs) protein–protein interaction (PPI) network and weighted gene co-expression network analysis (WGCNA) were performed, finally, the dysregulation of Hippo pathway in ectopic endometrium (EC) was detected by Western blotting. A total of 1,811 DEGs between eutopic (EU) and normal endometrium (NE), 5,947 DEGs between EC and EU, and 3,192 DEGs between EC and NE datasets were identified. After screening, 394 CDGs were obtained, and 5 hub genes identified in the PPI network. CDGs enrichment and WGCNA network analysis revealed cell proliferation, differentiation, migration and other biological processes, Hippo and Wnt signaling pathways, and a variety of tumor-related pathways. Western blotting results showed that YAP/TAZ was upregulated, and MOB1, pMOB1, SAV1, LATS1 and LATS2 were downregulated in EC. Moreover, CDGs, especially the hub genes, are potential biomarkers and therapeutic targets. Finally, the Hippo pathway might play a key role in the development of endometriosis.

## Introduction

Endometriosis (EM) is a common gynecological disease and one of the leading causes of female infertility and chronic pelvic pain (Giudice & Kao, 2004). Although the prevalence of EM in fertile women is approximately 10% to 15% (Olive & Pritts, 2001), its exact pathogenesis is not yet clear. A classic hypothesis of EM etiology is the “retrograde menstruation theory” proposed by Sampson (Sampson, 1927) in 1927. According to this theory, endometrial debris enters the abdominal cavity with menstrual reflux, and then adheres in this ectopic position forming lesions. However, there are still many gaps in this theory, and it has not been proven conclusively. Subsequent studies found that EM is also associated with genetic, immunological, and inflammatory factors, along with endometrial stem/progenitor cells (Burney & Giudice, 2012; Riccio et al., 2018; Cousins, Dorien & Gargett, 2018). As a chronic disease, EM seriously affects the quality of life of patients and causes a heavy social and economic burden. Therefore, it is extremely important to explore its underlying molecular mechanisms, and discover novel markers to facilitate early diagnosis and individualized treatment.

Gene chip technology is a high-throughput detection method that can analyze the expression of all genes in a given tissue/organism at one time, making it an ideal technique for screening disease-related genes and biomarkers. Since the advent of gene-chip technology, a large amount of high-throughput data has been generated, uploaded and stored in public databases. Unfortunately, the use of these data is limited by the narrow objectives of most studies, and its full potential has not been tapped yet.

To understand the pathogenesis of EM, it is essential to compare the ectopic (EC), eutopic (EU) and normal (NE) endometrial tissues since all three are concurrently involved in EM development. Ectopic endometrium (EC) is defined as a condition that endometrium-like tissue grows outside the uterine cavity in patients with endometriosis, ectopic endometriotic nodules are formed due to the cyclical bleeding of EC and the fibrosis of the surrounding tissues, which could eventually lead to symptoms of dysmenorrhea, chronic pelvic pain, abnormal menstruation and infertility. Eutopic endometrium (EU) refers to the is the endometrial tissue grows in the uterine cavity in patients with endometriosis, while, the normal endometrium (NE) refers to the endometrial tissue grows in uterine cavity in patients with non-endometriosis. Some studies have reported that the EU in endometriosis patients has stronger proliferation, migration and invasion ability than the NE in non-endometriosis patients, thus, EU cells attach and colonize more easier outside of uterine cavity, thereby causing the occurrence of endometriosis. However, studies profiling the gene expression in EM have only focused on two out the three tissues, either due to the limitation of the research scale or the experimental design (Hever et al., 2007; Burney et al., 2007). In addition, traditional expression studies ultimately focus on the structural and/or functional changes in a single or a few genes. However, interactions between genes are complex, and any pathophysiological condition is a result of the synergistic effects of a large number of genes.

To overcome these limitations and gain new insights into EM, we first used a bioinformatics approach to integrate the existing expression profile data on all EM-related tissues in order to identify the differentially expressed genes. The interaction between these genes was then elucidated using biological networks.

## Materials & Methods

### Data mining

The gene expression profiles used in this study were downloaded from the Gene Expression Omnibus (GEO) (https://www.ncbi.nlm.nih.gov/geo) in three different series: GSE7305, GSE6364 and GSE4888 (Data S1). GSE7305 contains datasets of 10 paired EC and EU samples (Hever et al., 2007), GSE6364 contains datasets of 21 EU and 16 NE samples (Burney et al., 2007), and GSE4888 contains datasets of 27 NE samples (Talbi et al., 2006). After removing duplicate samples, 69 samples (EC = 10, EU = 31, NE = 28) were included for the samples (Table 1).

**Table 1:** Source datasets overview.

**Series**	**Samples**			**Platform**
	**NE**	**EU**	**EC**	
GSE7305	–	10 / 10	10 / 10	HG-U133 PLUS 2.0
GSE6364	16 / 16	21 / 21	–	HG-U133 PLUS 2.0
GSE4888	27 / 12	–	–	HG-U133 PLUS 2.0

**Notes.**

Before “/” is the number of original samples contained in the data set, and after “/” is the actual number of samples used for analysis after excluding duplicate samples.

### Data preprocessing and principal component analysis

The raw data was analyzed by the Affy package and batch effect removal was performed by sva package in R. After obtaining the normalized gene expression profiles, the principal component analysis (PCA) of 69 samples was performed based on the matrix.

### Identification of differentially expressed genes

The annotation for the probes was downloaded from Affymetrix official website ( http://www.affymetrix.com/support/technical/annotationfilesmain.affx). limma package was used to perform the pairwise comparisons of the three groups to screen for differentially expressed genes (DEGs) at *P* value < 0.01. Core differential genes (CDGs) shared by the three pairwise comparisons were thus obtained.

### GO and KEGG pathway analysis

Gene Ontology (GO) and Kyoto Encyclopedia of Genes and Genomes (KEGG) (Kanehisa & Goto, 2000) analysis of the candidate genes were performed using DAVID online tool (https://david.ncifcrf.gov/summary.jsp). The enrichment significance threshold was set to *P* < 0.05.

### Protein–protein interaction network analysis

A CDGs-based protein–protein interaction (PPI) network was constructed using STRING (https://string-db.org) with high confidence >0.7) (Franceschini et al., 2013). Cytoscape (Shannon et al., 2003) was used to visualize the PPI network and analyze the connectivity degree. A node with a degree of connectivity greater than 10 was defined as a hub gene and is indicated in the figure.

### Weighted gene co-expression network analysis

To further study the synergy between genes, a weighted gene co-expression network was constructed using WGCNA package (Langfelder & Horvath, 2008). The probe with coefficient of variation (CV) ≥ 0.2 was selected for co-expression network construction. The module was then used to correlate the source trait (EC/ EU/ NE) of the endometrial tissues with the expression value of eigengene. Finally, candidate gene/probe expression values were aggregated into 28 different co-expression modules.

### Clinical sample collection

In order to verify the association of the Hippo pathway with EM, we analyzed protein expression in EC, EU and NE endometrium by Western blotting. All subjects gave their written informed consent for inclusion before they participated in the study. The study was conducted in accordance with the Declaration of Helsinki, and the protocol was approved by the Ethics Committee of the Obstetrics and Gynecology Hospital of Zhejiang University School of Medicine (IRB approval number: 20160054). The patients’ age was between 25–40 years, their menstrual cycles were regular, and all tissue samples were collected in the proliferative phase. No steroids were given to the patients at least 6 months prior to study inclusion.

Three paired EC and EU tissue samples were collected from patients at stage III/IV ovarian EM (diagnosed under a laparoscope according to the revised AFS classification), and three NE tissue samples were obtained from women without visible endometriosis.

### Protein extraction and western blot

Total protein was extracted from the tissues using an immunoprecipitation lysate containing protease and phosphatase inhibitors, and measured using a BCA protein quantification kit (Thermo Fisher Scientific). Equal amounts (30 µg) of protein samples were separated in a 10% SDS polyacrylamide gel and transferred to polyvinylidene fluoride (PVDF) membranes (Thermo Fisher Scientific). The membranes were incubated overnight with primary rabbit antibodies against YAP/TAZ (1:1000, #8579; Cell Signaling Technology), SAV1 (1:1000, #8579; Cell Signaling Technology), MOB1 (1:1000, #8579; Cell Signaling Technology), phospho-MOB1 (1:1000, #8579; Cell Signaling Technology), LATS1 (1:1000, #8579; Cell Signaling Technology), LATS2 (1:1000, DF7516; Affinity Biosciences) and GAPDH (1:1000, ab9485; Abcam) at 4 °C, followed by HRP-conjugated goat anti-rabbit secondary antibody (1:1000; Abcam) for 1 h at room temperature. The signals were detected with an enhanced chemiluminescence detection reagent (Thermo Fisher Scientific), and the band densities were quantified and analyzed with Quantity One software (Bio-Rad Laboratories, Hercules, CA, USA). Relative protein levels were normalized to GAPDH.

## Results

### Principal component analysis

**Figure 1:**
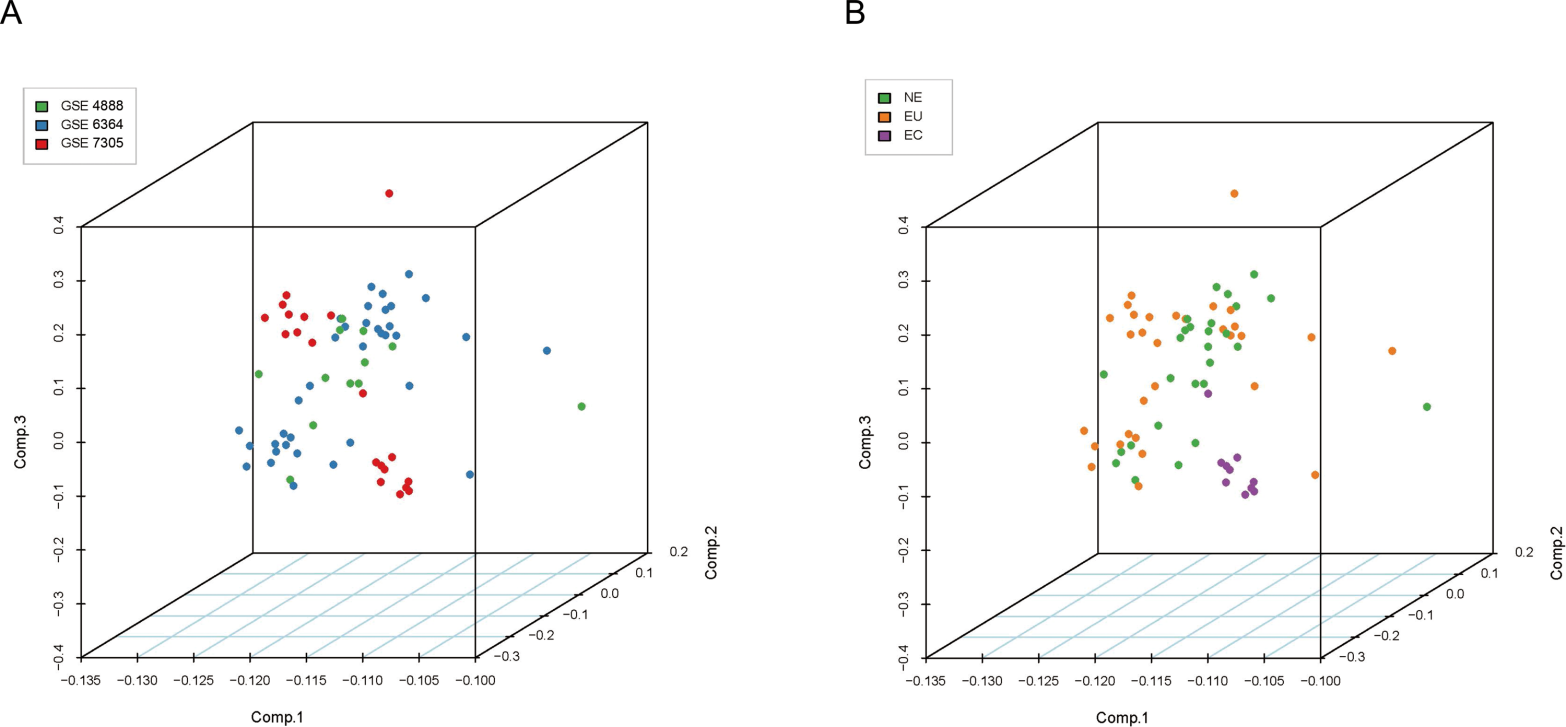
Principal component analysis (PCA) of gene expression profiles. Samples are marked with different colors based on the batch or the histologic origin. (A) Green for GSE4888, blue for GSE7305, and red for GSE6364; (B) Green for NE, orange for EU, and purple for EC.

The downloaded raw data was standardized to eliminate inter-assay differences. PCA was performed based on the expression profile of the 69 samples. The results showed that sample clustering was not related to the batch (Fig. 1A, Data S2), which eliminated the batch effect. The EC samples were significantly different from the other two groups, while the latter did not differ significantly amongst themselves (Fig. 1B, Data S2).

### Identification of DEGs between EC, EU and NE

**Figure 2:**
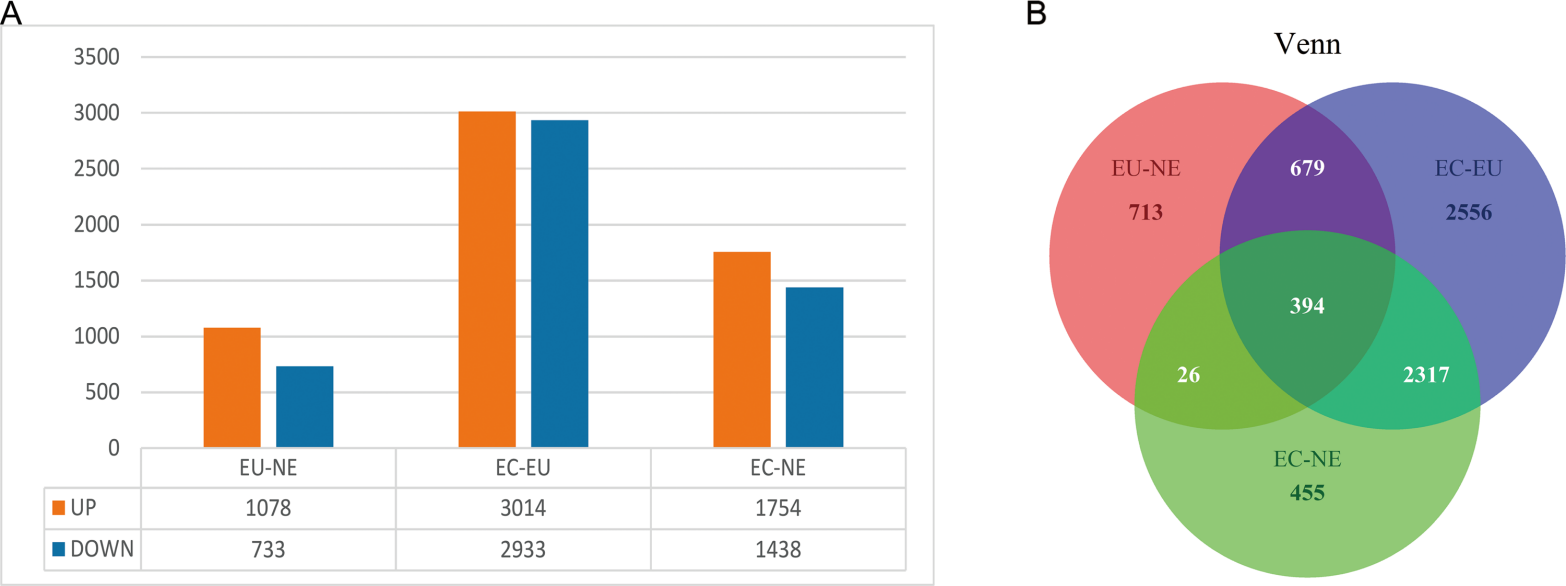
DEGs between EC, EU and NE. (A) Statistical analysis of DEGs between the three pairwise comparisons. Orange represents the number of up-regulated DEGs, and blue represents the number of down-regulated DEGs; (B) Venn diagrams of DEGs. Red represents DEGs between EU and NE, blue represents DEGs between EC and EU, and green represents DEGs between EC and NE. Crossing areas represent shared DEGs.

EC, EU and NE samples were compared in pairs to identify DEGs, and Venn diagrams were drawn. The threshold was set at *P* < 0.01. A total of 1,811 DEGs (10,78 upregulated and 733 downregulated) between EU and NE, 5947 DEGs (3014 upregulated and 2,933 downregulated) between EC and EU, and 3,192 DEGs (1,754 upregulated and 1,438 downregulated) between EC and NE were observed (Fig. 2A, Data S3, Data S4). The top 5 up- and down-regulated genes in the three pairwise comparisons based on log2FC are listed in Table 2. To summarize, the least number of DEGs was seen between EU and NE (*n* = 1811) and the highest between EC and EU (*n* = 5,947). This was contrary to our expectation that greater differences ought to be seen between EC and NE rather than EC and EU. A total of 394 CDGs were shared across all three pairs (Fig. 2B, Data S5).

### GO and KEGG pathway analysis of CDGs

**Figure 3:**
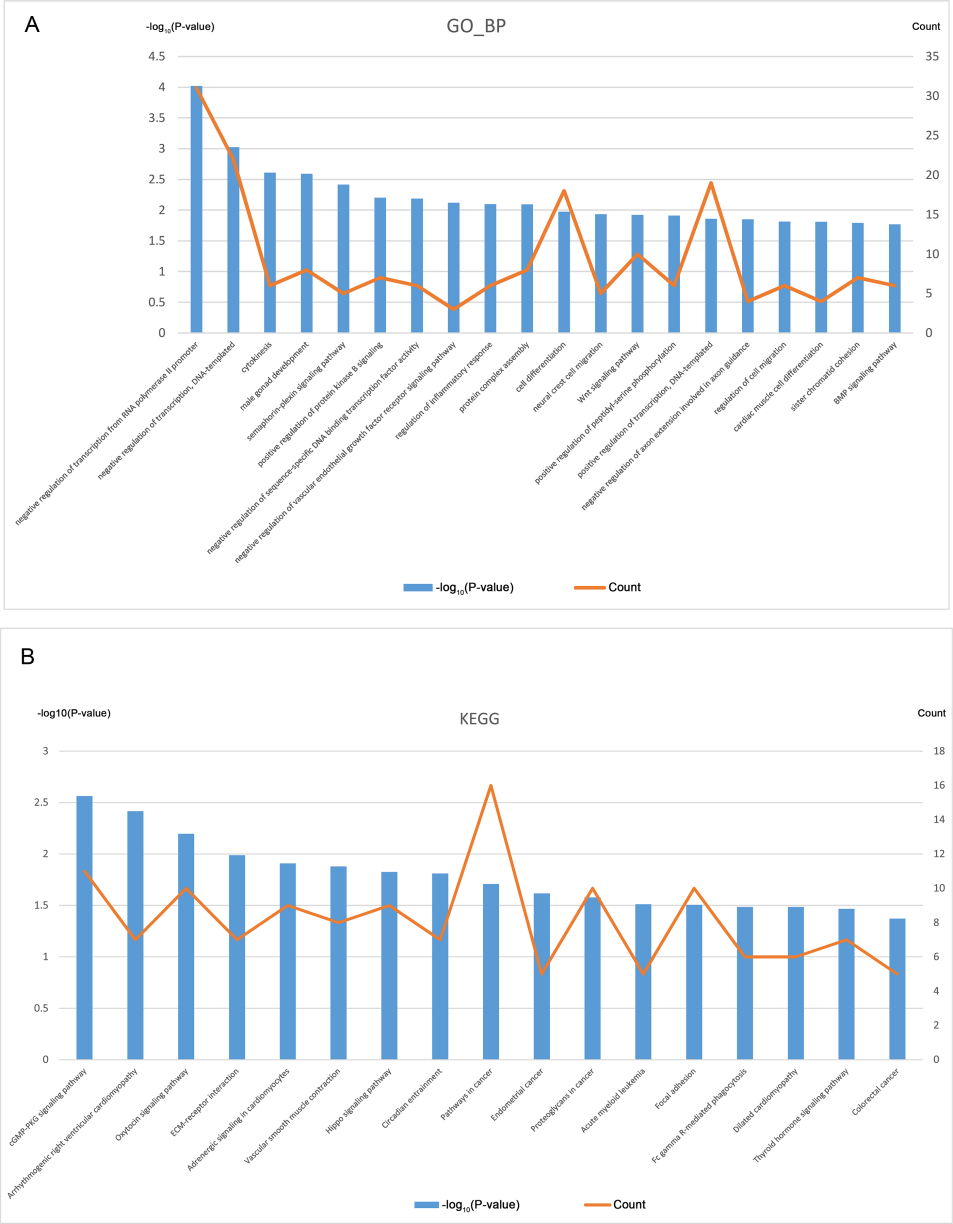
GO and KEGG analysis of CDGs (top 20). (A) GO (BP) enrichment analysis for 394 CDGs. (B) KEGG enrichment analysis for 394 CDGs.

GO analysis was performed based on the 394 CDGs (Data S6), and Fig. 3A shows the top 20 entries of BP terms. The enriched CDGs included not only those involved in important cellular activities like cytokinesis, cell differentiation, and cell migration but also the regulation of the key biological processes such as the EGFR and Wnt signaling pathways, inflammatory response etc.

**Table 2:** Differentially expressed genes between EC, EU, and NE (top five).

**Up-regulated**			**Down-regulated**		
**EU-NE**	**EC-EU**	**EC-NE**	**EU-NE**	**EC-EU**	**EC-NE**
RRM2	DLK1	CLDN11	ABCA8	SCGB1D2	MMP26
CDCA7	CLDN11	DLK1	CEMIP	LINC01541	SCGB1D2
NDC80	GATA6	CHL1	GATA6	SCGB2A1	SCGB2A1
GDF7	SCN7A	SCN7A	CHI3L1	GABRP	LINC01541
HOXA10	COL8A1	GATA6	LOC102723694	SLC26A7	KIAA1324

**Figure 4:**
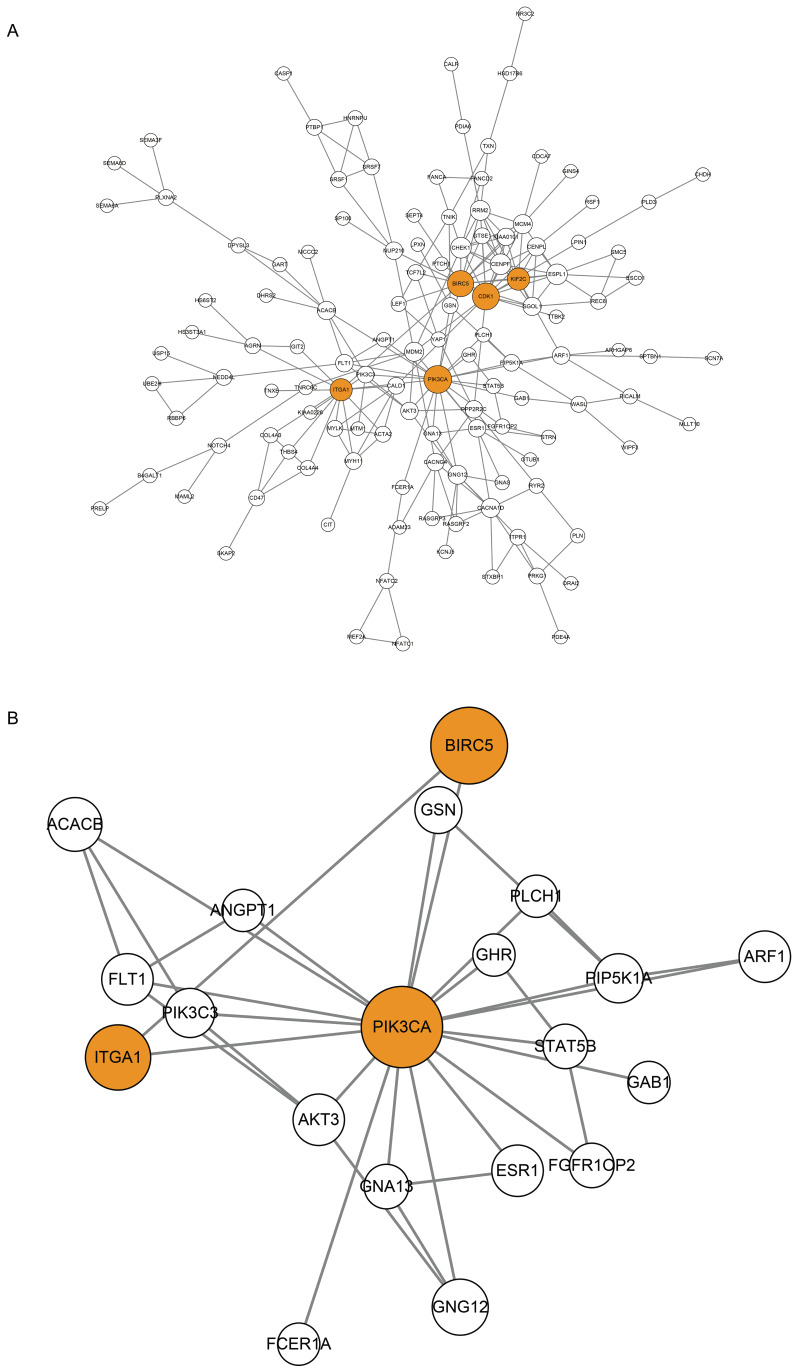
Protein-Protein Interaction (PPI) Network of CDGs. (A) Nodes represent CDGs, and edges represent interactions. Hub genes with connectivity degree >10 are marked in orange. (B) Subnetwork constructed with PIK3CA as the core.

KEGG pathway analysis was also performed on the CDGs (Data S7) and the top 20 entries with significant enrichment are shown in Fig. 3B. The enriched CDGs in pathways relevant to endometrial cancer and colorectal cancer may be related to the malignant features of EM. Furthermore, enriched CDGs in the Hippo signaling pathway suggests its involvement in the pathophysiological process of EM.

### Protein–protein interaction network of CDGs

Using STRING database (Franceschini et al., 2013) and Cytoscape software (Shannon et al., 2003), CDGs were identified for protein-protien interactions and a PPI network was constructed. After screening, 134 CDGs formed a PPI network consisting of 134 nodes and 227 edges (Fig. 4A). The size of the node is proportional to the degree of connectivity and a node with a connectivity degree > 10 is defined as a hub gene. A total of five hub genes were obtained: PIK3CA, CDK1, BIRC5, KIF2C, ITGA1 (Table 3), and marked in orange in the Fig. 4B. Using PIK3CA, the most connected hub gene, as the core, the nodes were directly connected to it to form the subnetwork containing 20 nodes and 33 edges (Fig. 4B).

### Weighted gene co-expression network analysis

**Figure 5:**
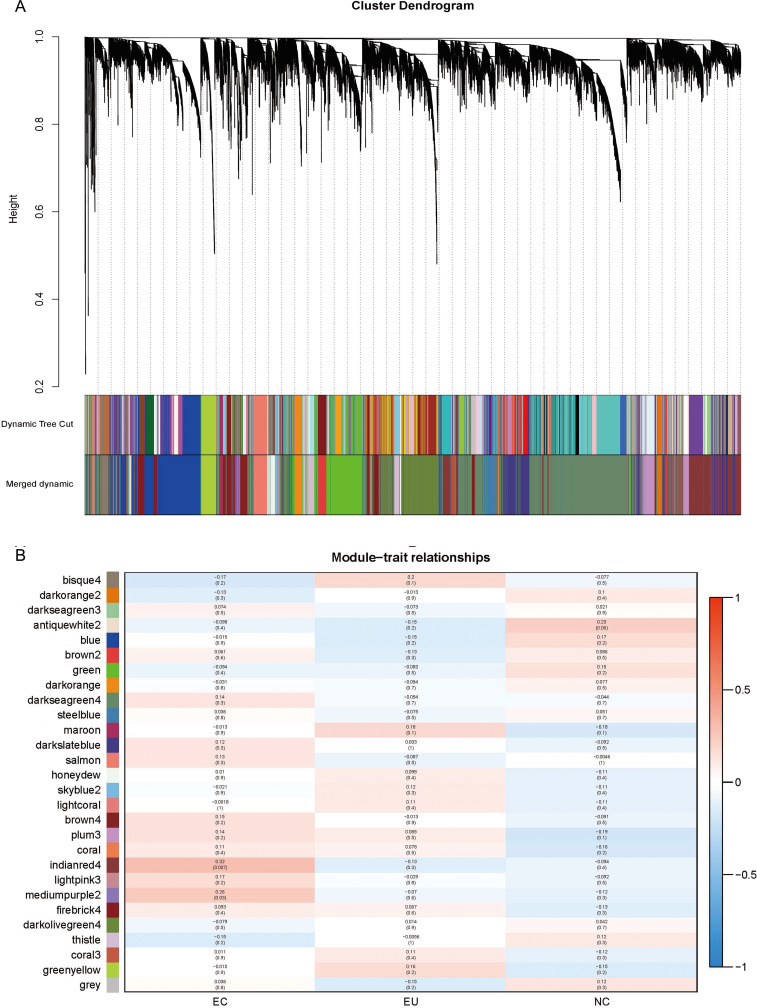
Weighted gene co-expression network analysis (WGCNA) and Module trait relationship. (A) The co-expression modules for each gene below the hierarchical clustering tree are marked with different colors. Dynamic Tree Cut corresponds to the original module; Merged dynamic corresponds to the merged modules finally obtained. (B) Red indicates positive correlation and blue indicates negative correlation. The number above each cell indicates the correlation coefficient between the module and the corresponding endometrial tissue, and the number in parentheses indicates the corresponding *P* value.

To further understand the synergy between genes, we performed WGCNA analysis on the gene expression profiles. The gene co-expression network was constructed on the basis of the correlation between the expression values of all the sample genes. Highly related genes formed a module, and 28 co-expression modules were finally identified (Fig. 5A; marked with different colors). The association of different modules with traits (i.e., different sources of endometrium: EC/ EU/ NE) showed EC correlated most with the “indianred4” module (*R* = 0.32, *P* = 0.007), followed by the “mediumpurple2” module (*R* = 0.26, *P* = 0.03) (Fig. 5B). The high correlation of the genes in these two modules with EC suggests their involvement in EM.

### Function annotation of EC-related modules

**Figure 6:**
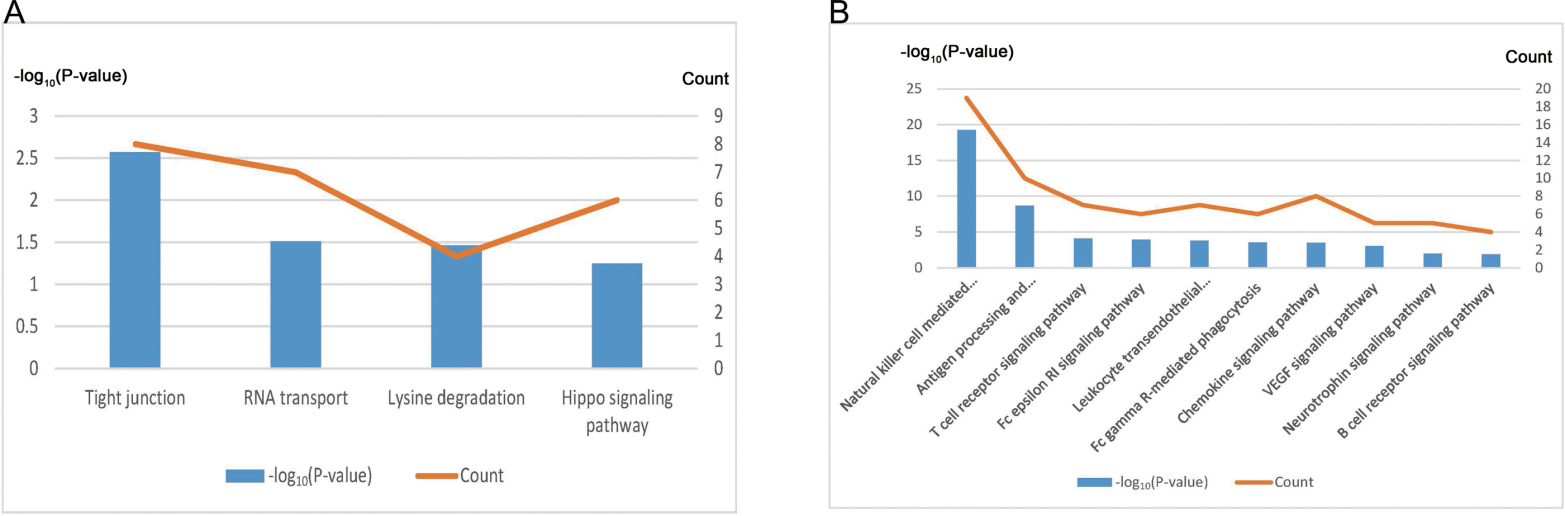
Functional annotation of EC-related modules. (A) KEGG pathway analysis of genes within the “indianred4” module. (B) KEGG pathway analysis of genes within the “mediumpurple2” module.

We conducted further analysis on the two modules showing highest correlation with EC. After annotating, the “indianred4” module contained 421 genes, and the “mediumpurple2” module contained 121 genes (Table 4, Data S8). KEGG pathway enrichment analysis revealed that the genes of the “indianred4” module were enriched in the Tight junction, RNA transport, Lysine degradation and Hippo signaling pathway entries (Fig. 6A, Table 4), while those of the “mediumpurple2” module were enriched mainly in Natural killer cell mediated cytotoxicity, Antigen processing and presentation, T cell and B cell receptor signaling pathway and other immune-related pathways, and VEGF Signaling pathway and Neurotrophin signaling pathway entries (Fig. 6B, Table 4). Therefore, the “indianred4” module may be related to the adhesion and growth of ectopic endometrium while the ”mediumpurple2” module to EMs-related immune responses, and the formation of new blood vessels and nerves.

**Table 3:** Hub genes in PPI networks of CDGs (degree of connectivity >10).

**Gene symbol**	**Connectivity degree**
PIK3CA	19
CDK1	18
BIRC5	17
KIF2C	12
ITGA1	12

### Dysregulation of Hippo signaling pathway in EMs

**Figure 7:**
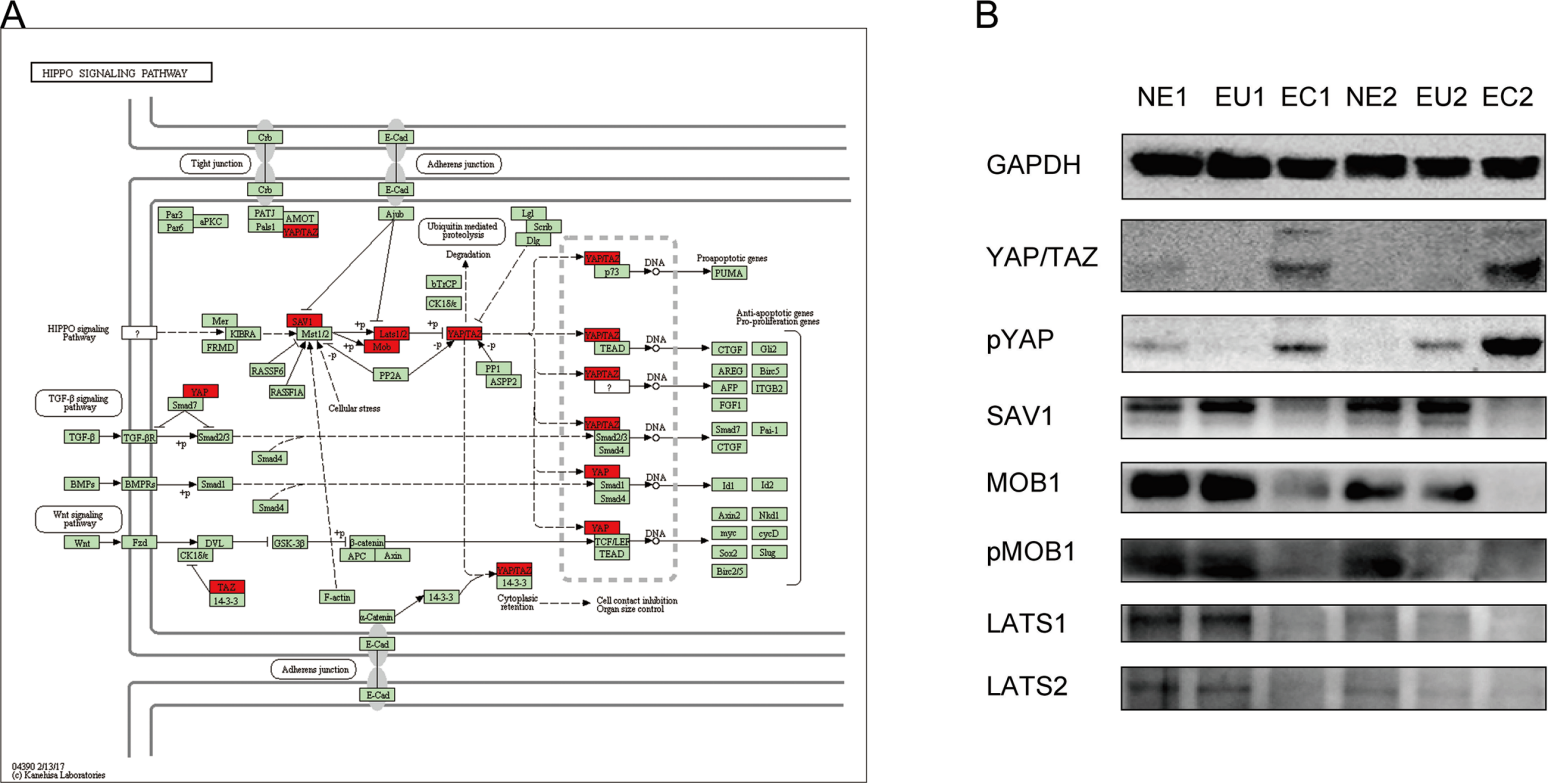
Dysregulation of Hippo signaling pathway in EC. (A) Schematic diagram of the Hippo signaling pathway. The key genes of interest are marked in red. (Image credit: the KEGG archive at https://www.kegg.jp). (B) Western blotting was performed to detect the expression and phosphorylation of Hippo signaling pathway proteins in EC, EU and NE.

KEGG results indicated a key role of the Hippo signaling pathway in EM pathogenesis. In order to verify the association between the Hippo signaling pathway and EM, we confirmed the expression of the proteins encoded by the key genes (indicated on the map) of this pathway by Western blotting (Fig. 7A). Compared to the EU and NE samples, YAP/TAZ expression was increased, and SAV1, MOB1, p-MOB1, LATS1, and LATS2 were decreased in EC (Fig. 7B). These result indicate that the Hippo signaling pathway is abnormally activated in EC compared to EU and NE.

## Discussion

In order to compare the overall molecular phenotypes of ectopic (EC) and eutopic (EU) endometrial and non-endometrial (NE) tissues, and to identify key genes and pathways associated with EM, we performed bioinformatics analysis on gene-chip expression profiles from GEO database. We screened DEGs, constructed PPIs and weighted gene co-expression networks, and identified hub nodes and important co-expression modules in the networks. Finally, Western blotting was performed to validate the dysregulation of Hippo signaling pathway in EC tissues. These results provide new insights into EM.

Due to the different screening thresholds set in various studies, and inconsistencies in the batches, platforms, and observation priorities, it was difficult to directly compare those studies. According to the classical implantation theory (Sampson, 1927), ectopic endometrial lesions may originate from the recurrent endometrial tissue that flows back into the abdominal cavity with menstrual blood. Subsequent studies also reported that compared to normal endometrium, ectopic and eutopic endometria show stronger proliferation, adhesion, migration and invasiveness (Lin et al., 2018; Yotova et al., 2011; Wingfield et al., 1995; Sundqvist et al., 2012), which is important for the ectopic implantation and growth of the endometrial tissues. Ideally, the EC tissues ought to be closer to EU in terms of biological behavior and overall molecular phenotype. However, our results showed the highest number of DEGs between EC and EU, which prompts us to rethink the histologic origin of the ectopic endometrium.

**Table 4:** Genes within EC-related modules and KEGG pathway analysis (top 10).

**Module**	**Gene**	**KEGG Term**	**Count**	***P*-value**
indianred4	421	Tight junction	8	2.68 ×10^−3^
		RNA transport	7	3.07 ×10^−2^
		Lysine degradation	4	3.43 ×10^−2^
		Hippo signaling pathway	6	5.64 ×10^−2^
mediumpurple2	121	Natural killer cell mediated cytotoxicity	19	5.47 ×10^−20^
		Antigen processing and presentation	10	2.09 ×10^−9^
		T cell receptor signaling pathway	7	7.88 ×10^−5^
		Fc epsilon RI signaling pathway	6	1.08 ×10^−4^
		Leukocyte transendothelial migration	7	1.68 ×10^−4^
		Fc gamma R-mediated phagocytosis	6	2.95 ×10^−4^
		Chemokine signaling pathway	8	3.02 ×10^−4^
		VEGF signaling pathway	5	8.66 ×10^−4^
		Neurotrophin signaling pathway	5	1.00 ×10^−2^
		B cell receptor signaling pathway	4	1.27 ×10^−2^

The implantation hypothesis proposed by Sampson (1927) is accepted by most researchers. However, this theory cannot explain why only 10% of menstruating women are effected by EM when most women have retrograde menstruation (Olive & Pritts, 2001), or why extra-pelvic EM and pre-menarche EM occur (Jubanyik & Comite, 1997; Schifrin, Erez & Moore, 1973). With the discovery of endometrial stem cells, Sampson’s theory has been expanded; according to the new stem cell theory, endometrial stem cells are abnormally shed during menstruation and enter the abdominal cavity with retrograde menstruation and are ectopically implanted, eventually leading to EM (Starzinski-Powitz et al., 2001; Leyendecker et al., 2002; Gargett, 2007). This theory enhances the interpretive capacity of the morbidity model of EM, and at the same time explains our results; it is likely that the EC tissue does not originate from completely differentiated EU cells, but may be formed as a separate lineage during the initial differentiation.

The PPI network analysis revealed 5 hub genes: PIK3CA, CDK1, BIRC5, KIF2C and ITGA1. Anglesio et al. (2017) reported several cancer driver mutations, including PIK3CA, in 5 out of 27 non-cancer EM. Other studies have reported aberrant expression of CDK1 in gynecologic tumors such as ovarian cancer, cervical cancer and endometrial cancer (Bae et al., 2015; Luo et al., 2016; Li, Qu & Li, 2017), but its association with EM has not been verified. BIRC5, also known as Survivin, is an anti-apoptotic and pro-proliferative factor associated with various tumors (Ghaffari et al., 2016; Wang et al., 2014; Wang et al., 2016), and has recently been confirmed as a crucial factor in EM development as well as a valid diagnostic biomarker (Uegaki et al., 2015; Acimovic et al., 2016). KIF2C, the most representative member of the mitotic and cell cycle regulating Kinesin-13 family, is closely related to hepatocellular carcinoma, breast cancer and other tumors (Chen et al., 2017; Shimo et al., 2008), but its role in EMs is unclear. ITGA1 indirectly influences the proliferation, differentiation, and apoptosis of tumor cells by mediating cell adhesion processes (Yim et al., 2013; Boudjadi et al., 2016), but its association with EMs has not been reported.

The Hippo signaling pathway was first discovered in 1995 (Justice et al., 1995; Xu et al., 1995). It is highly conserved in mammals and regulates organ size and homeostasis by maintaining the balance between cell proliferation and apoptosis. The key component of the human Hippo signaling pathway is the core kinase chain consisting of MST1/2, SAV1, LATS1/2, and MOB1, which inhibit cell proliferation and promote apoptosis by inhibiting the downstream YAP/TAZ transcriptional coactivator (Yu, Zhao & Guan, 2015). YAP is the main effector molecule of the Hippo signaling pathway, which can promote proliferation, inhibit apoptosis, and ultimately lead to loss of cellular contact inhibition and malignant transformation. Dysregulation of the Hippo signaling pathway in tumors has been confirmed by a large number of studies in recent years (Janse van Rensburg & Yang, 2016; Cao & Huang, 2017). Interestingly, the Hippo signaling pathway also regulates the proliferation and differentiation of stem cells in various tissues (Li et al., 2015a; Yin & Zhang, 2015; Hiemer & Varelas, 2013). However, its association with EMs has only been reported for the downstream effector molecule YAP (Song et al., 2016). Our results suggests that the Hippo signaling pathway is abnormally activated in EC and may play an important role in the occurrence of EM.

Interestingly, three of the five identified hub genes are closely related to the Hippo pathway and its core factors. Studies have shown that the overexpression of PIK3CA is associated with the poor clinical prognosis in squamous cell carcinoma of the head and neck (HNSCC) (Garcia-Escudero et al., 2018). The tissue array showed that the expression of PIK3CA gene protein product (PI3Kp110α) was related to the YAP nuclear localization in HNSCC. Zhao et al. (2018) found that PIK3CA can enhance the activity of YAP and TAZ in breast tumors in vitro via multiple pathways. This indicated that YAP and TAZ were the key mediators of PIK3CA-induced breast tumorigenesis, and had a synergistic effect with PIK3CA in transforming the breast cells. Another study showed that the simultaneous activation of the PI3K and YAP pathways triggered the rapid development of liver tumors in mice, and a synergistic effect was observed between PI3K and YAP (Li et al., 2015b). The CDK1 phosphorylation by YAP can promote the mitotic defects, migration and invasion of tumor cells, which is essential for malignant transformation (Yang et al., 2013). On the other hand, CDK1 phosphorylation of TAZ may inhibit its carcinogenic activity (Zhang et al., 2015). BIRC5 is an important downstream effectors of the Hippo pathway, and its expression is regulated by the phosphorylation of YAP / TAZ, which in turn affects the cell proliferation, apoptosis, epithelial-mesenchymal transition, invasion and metastasis (Moriyama & Hori, 2019).

## Conclusions

Through comprehensive bioinformatics analysis of common gene-chip data, we compared gene expression between EC, EU, and NE tissues. Surprisingly, the number of DEGs between EC and EU was far greater than that between EC and NE. This may have implications for our understating of the underlying mechanism of EM. A total of 394 CDGs were shared by the three pair-wise comparisons, and five hub genes in the PPI network to which the CDGs belonged were finally screened. Through weighted gene co-expression network analysis, EM-related gene modules were identified. The CDGs enrichment analysis and EM-related modules functional annotation suggest that the Hippo signaling pathway may play an important role in EM. Western blotting results confirmed that compared to EU and NE, the expression and phosphorylation of the Hippo signaling pathway proteins were abnormal in EC, suggesting that the dysregulation of this pathway is of great significance in endometrial development. These above findings will help us better understand the pathological basis of EM and provide new directions for discovering diagnostic biomarkers and therapeutic targets.

##  Supplemental Information

10.7717/peerj.10171/supp-1 Supplemental Information 1 Each sample ID in data set and each sample information

10.7717/peerj.10171/supp-2 Supplemental Information 2 Continuous screenshots of the PCA of gene expression profiles

10.7717/peerj.10171/supp-3 Supplemental Information 3 Continuous screenshots of the PCA of gene expression profiles

10.7717/peerj.10171/supp-4 Supplemental Information 4 The volcano plot of DEGs for each pair.

10.7717/peerj.10171/supp-5 Supplemental Information 5 The volcano plot of DEGs for each pair.

10.7717/peerj.10171/supp-6 Supplemental Information 6 The volcano plot of DEGs for each pair.

10.7717/peerj.10171/supp-7 Supplemental Information 7 Detailed analysis results of the three pairs of comparisons

10.7717/peerj.10171/supp-8 Supplemental Information 8 Detailed analysis results of the three pairs of comparisons

10.7717/peerj.10171/supp-9 Supplemental Information 9 Detailed analysis results of the three pairs of comparisons

10.7717/peerj.10171/supp-10 Supplemental Information 10 Gene symbols of 394 CDGs.

10.7717/peerj.10171/supp-11 Supplemental Information 11 GO analysis of 394 CDGs.

10.7717/peerj.10171/supp-12 Supplemental Information 12 KEGG pathway analysis of 394 CDGs.

10.7717/peerj.10171/supp-13 Supplemental Information 13 Genes within the EC-related modules.

10.7717/peerj.10171/supp-14 Supplemental Information 14 Full-length blots of Fig. 7.

## Abbreviations

EM: endometriosis
GEO: Gene Expression Omnibus database
DEGs: differentially expressed genes
GO: Gene Ontology
KEGG: Kyoto Encyclopedia of Genes and Genome
CDGs: core differential genes
PPI: protein–protein internaction
WGCNA: weighted gene co-expression network analysis
EC: ectopic endometrium
EU: eutopic endometrium
NE: non-endometriosis endometrium
PPI: protein–protein internaction
YAP: Yes Associated Protein
TAZ: Tafazzin
MOB1: Mps One Binder Homolog A
SAV1: Salvador Family WW Domain Containing Protein 1
LATS1: Large Tumor Suppressor Kinase 1
LATS2: Large Tumor Suppressor Kinase 2
PCA: principal component analysis
GAPDH: glyceraldehyde phosphate dehydrogenase
BP: biological process
PIK3CA: Phosphatidylinositol-4,5-Bisphosphate 3-Kinase Catalytic Subunit Alpha
CDK1: Cyclin Dependent Kinases 1
BIRC5: Baculoviral Inhibitor of Apoptosis Repeat Containing 5
KIF2C: Kinesin Family Member 2C
ITGA1: Integrin Subunit Alpha 1

## Additional Information and Declarations

### Data Deposition

The following information was supplied regarding data availability: The raw data are available in the Supplemental Files.
