# Supplementary material for: Identification of key genes and pathways in endometriosis by integrated expression profiles analysis
Source: PeerJ. 2020 Dec 7;8:e10171. doi: 10.7717/peerj.10171 (PMC7727381; doi:10.7717/peerj.10171)
Supplement: Supplemental Information 3 [file peerj-08-10171-s003.pdf]

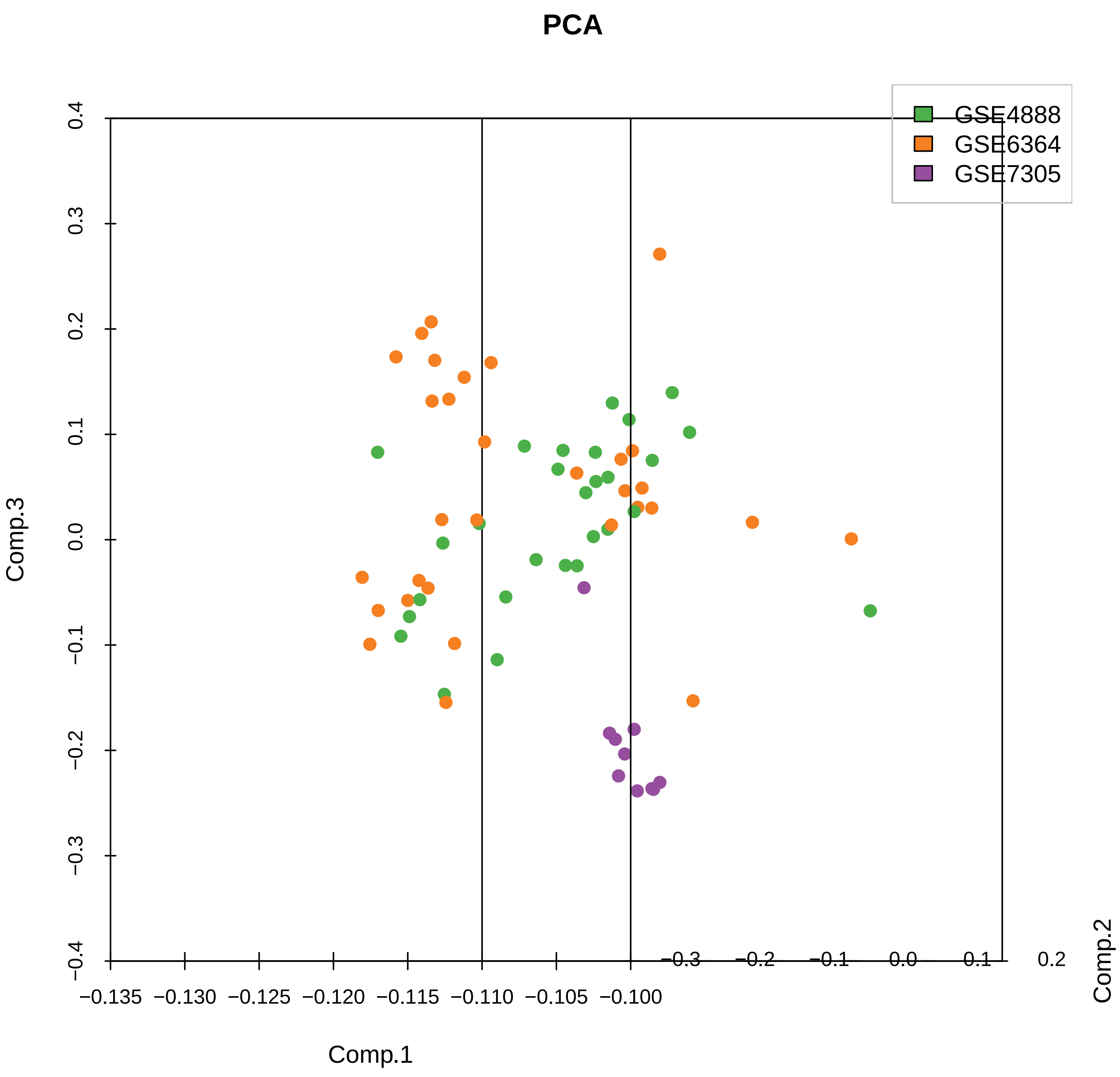
PCA
GSE4888
GSE6364
GSE7305
Comp.1
Comp.2
Comp.3
−0.135 −0.130 −0.125 −0.120 −0.115 −0.110 −0.105 −0.100
−0.3 −0.2 −0.1 0.0 0.1 0.2
−0.4 −0.3 −0.2 −0.1 0.0 0.1 0.2 0.3 0.4

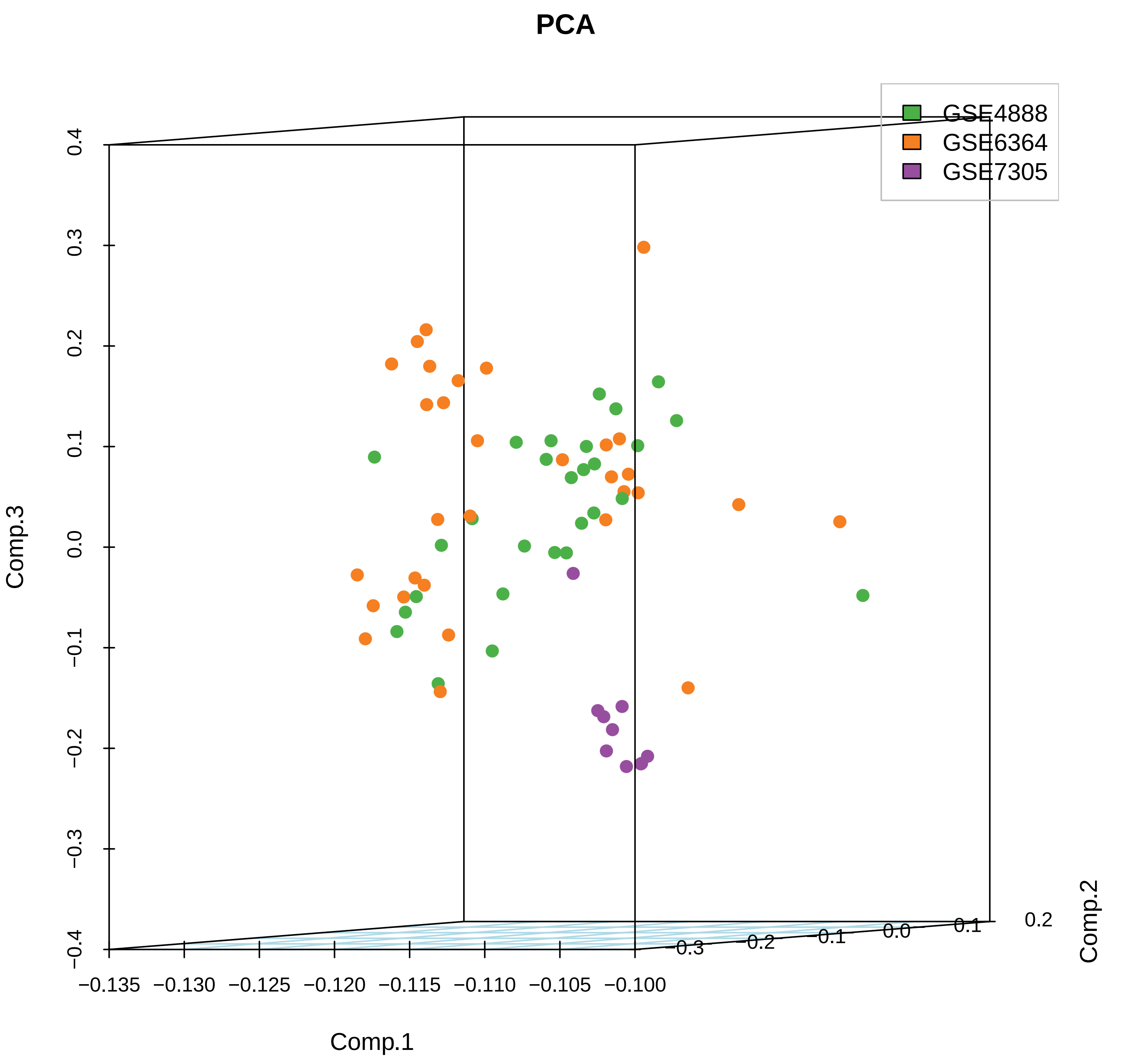

PCA
GSE4888
GSE6364
GSE7305
Comp.3
0.4
0.3
0.2
0.1
0.0
−0.1
−0.2
−0.3
−0.4
−0.135
−0.130
−0.125
−0.120
−0.115
−0.110
−0.105
−0.100
Comp.1
−0.3
−0.2
−0.1
0.0
0.1
0.2
Comp.2

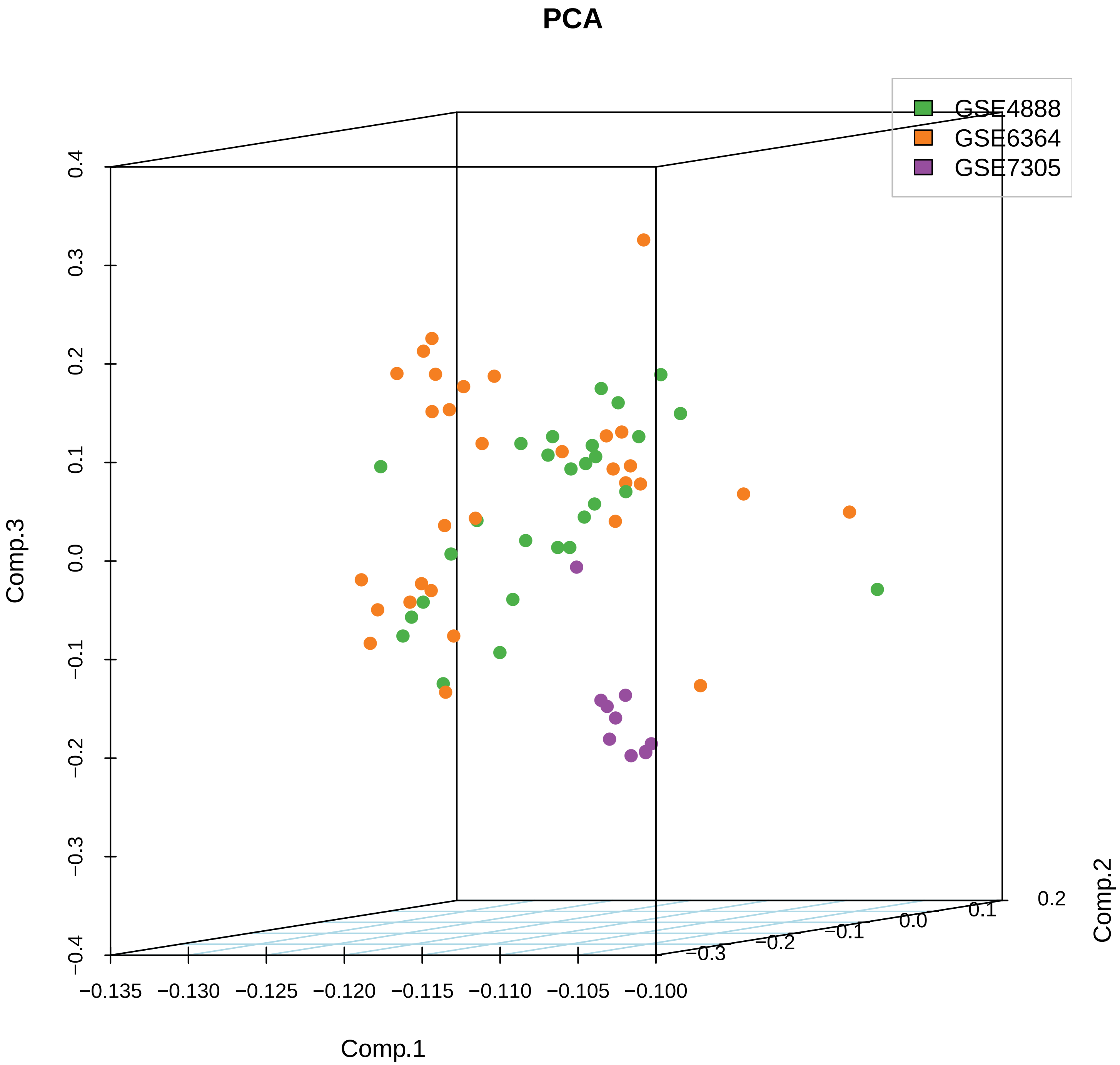
PCA
GSE4888
GSE6364
GSE7305
Comp.1
Comp.2
Comp.3
−0.135
−0.130
−0.125
−0.120
−0.115
−0.110
−0.105
−0.100
−0.3
−0.2
−0.1
0.0
0.1
0.2
−0.4
−0.3
−0.2
−0.1
0.0
0.1
0.2
0.3
0.4

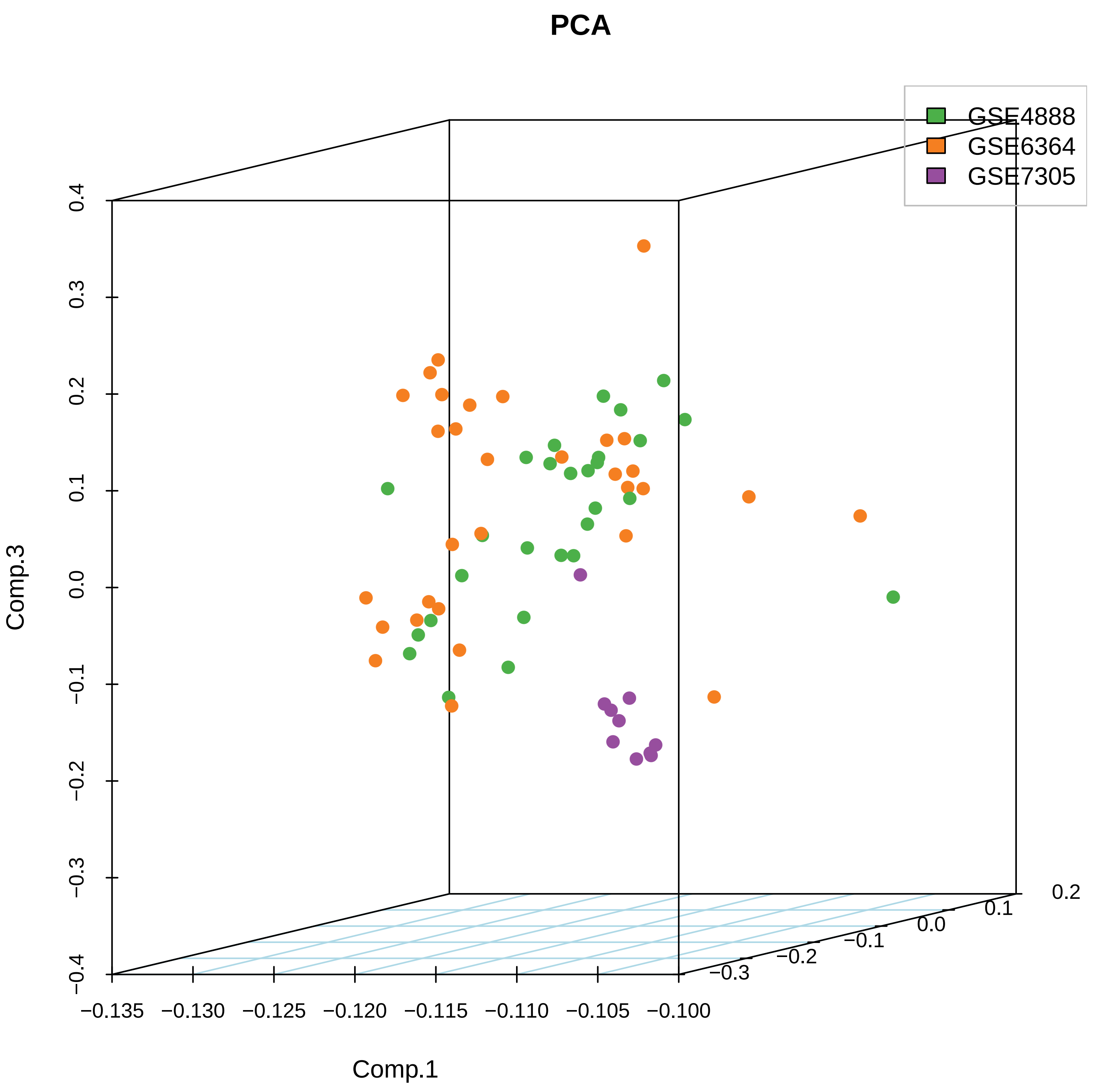
PCA
GSE4888
GSE6364
GSE7305
Comp.1
Comp.2
Comp.3
−0.135
−0.130
−0.125
−0.120
−0.115
−0.110
−0.105
−0.100
−0.3
−0.2
−0.1
0.0
0.1
0.2
−0.4
−0.3
−0.2
−0.1
0.0
0.1
0.2
0.3
0.4

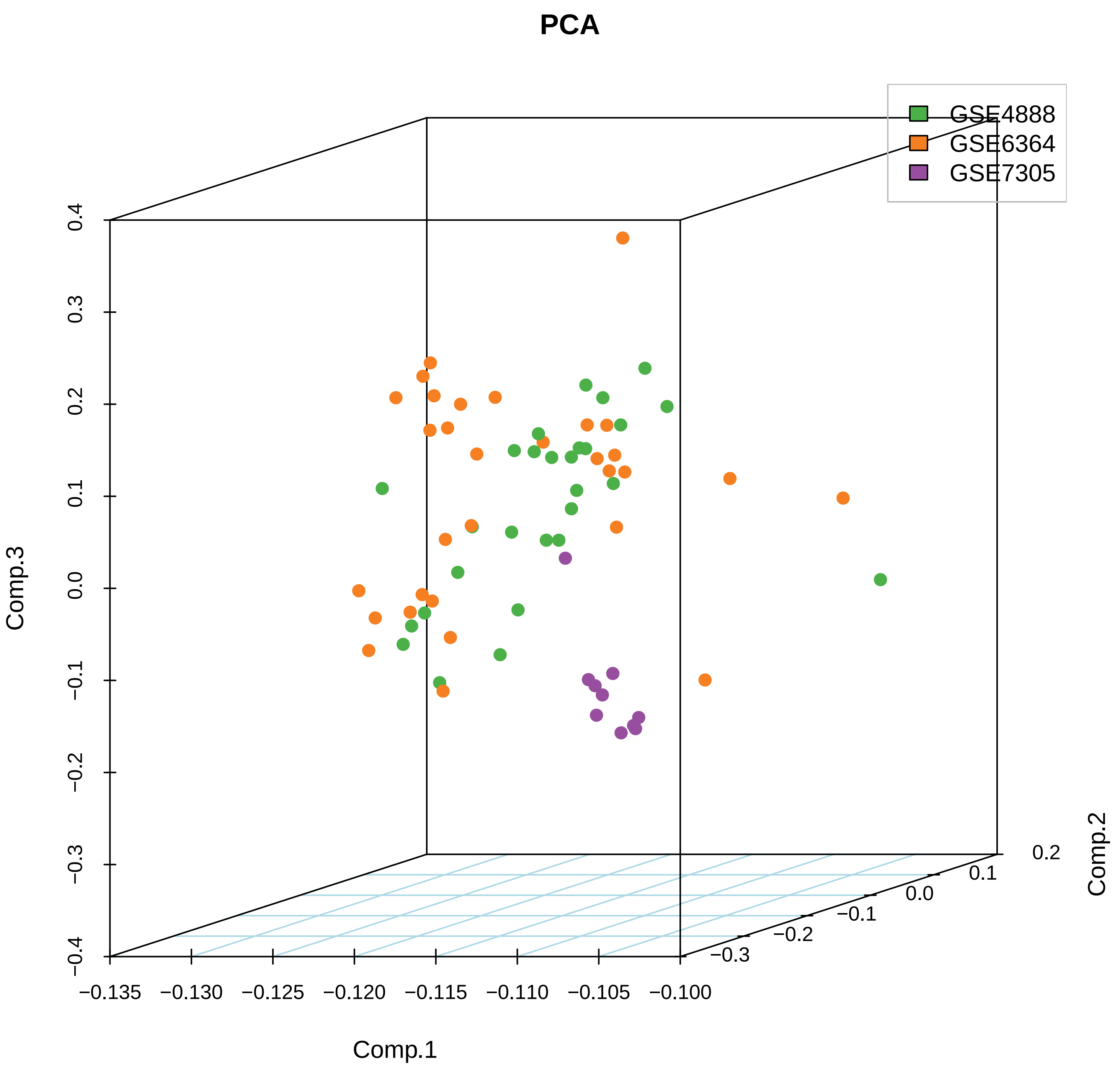
PCA
GSE4888
GSE6364
GSE7305
Comp.3
Comp.1
Comp.2
0.4
0.3
0.2
0.1
0.0
−0.1
−0.2
−0.3
−0.4
−0.135
−0.130
−0.125
−0.120
−0.115
−0.110
−0.105
−0.100
0.2
0.1
0.0
−0.1
−0.2
−0.3

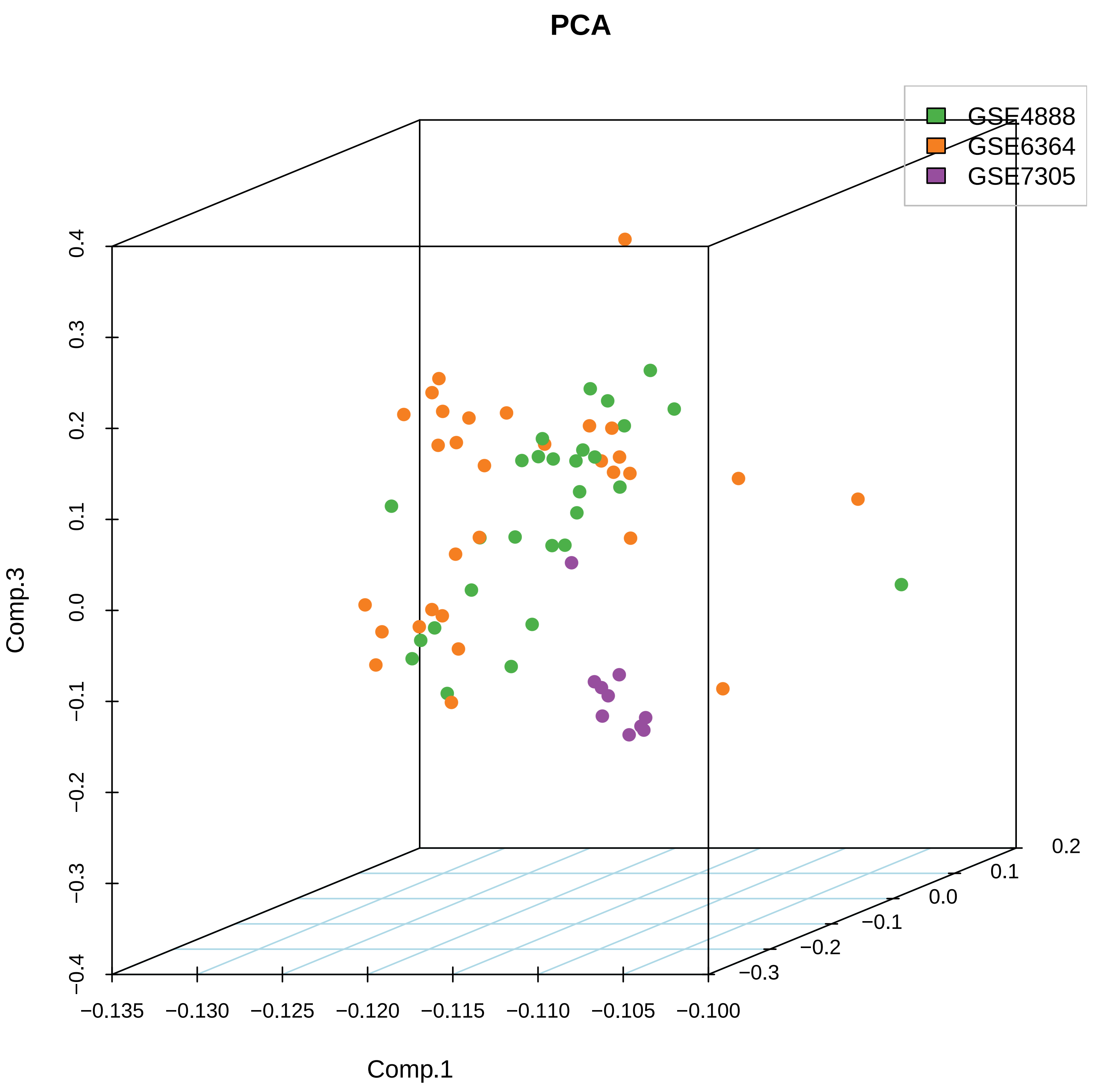
PCA
GSE4888
GSE6364
GSE7305
Comp.1
Comp.2
Comp.3
−0.135
−0.130
−0.125
−0.120
−0.115
−0.110
−0.105
−0.100
−0.3
−0.2
−0.1
0.0
0.1
0.2
−0.4
−0.3
−0.2
−0.1
0.0
0.1
0.2
0.3
0.4

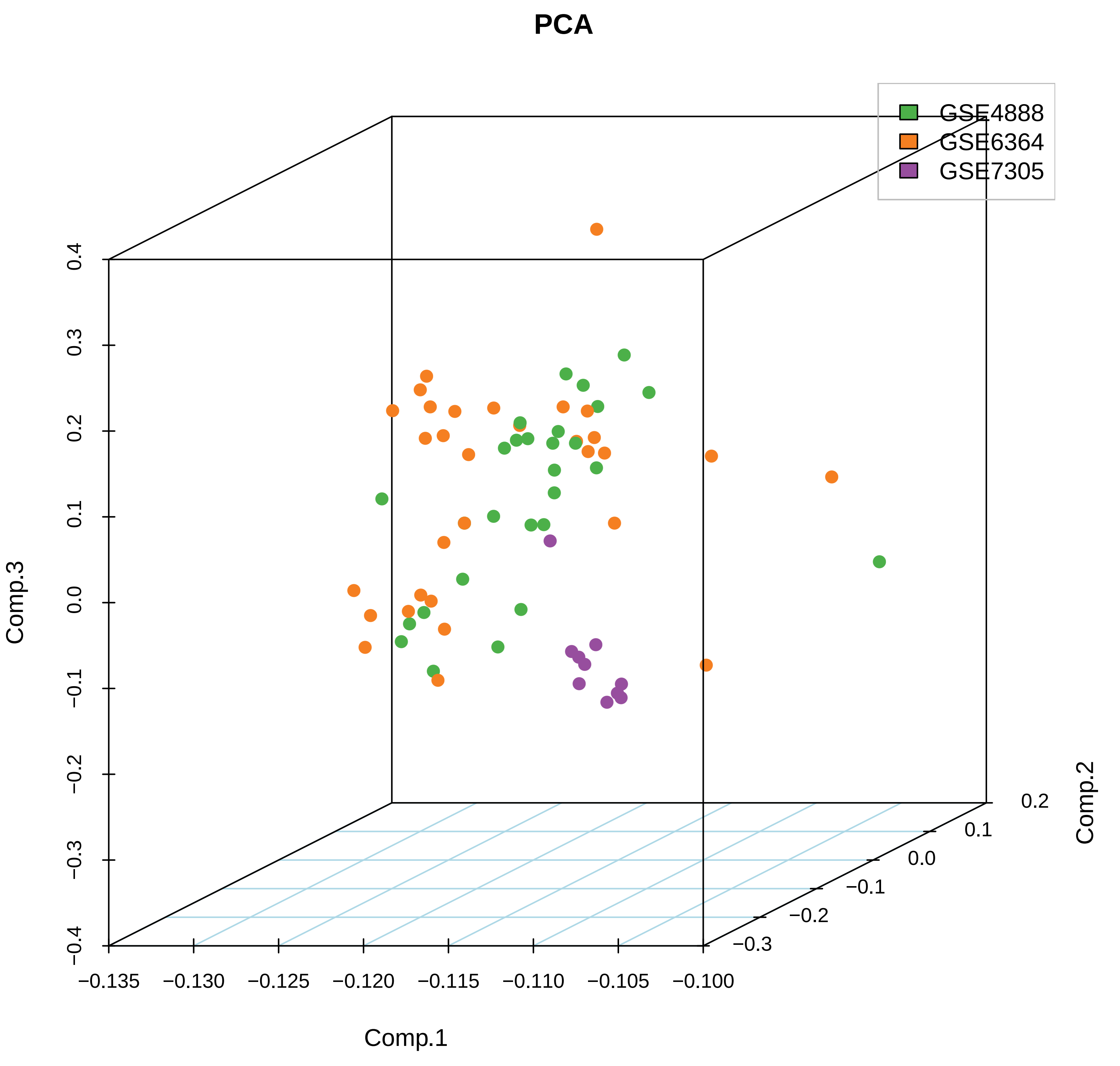

PCA
GSE4888
GSE6364
GSE7305
Comp.1
Comp.2
Comp.3
−0.135
−0.130
−0.125
−0.120
−0.115
−0.110
−0.105
−0.100
−0.3
−0.2
−0.1
0.0
0.1
0.2
−0.4
−0.3
−0.2
−0.1
0.0
0.1
0.2
0.3
0.4

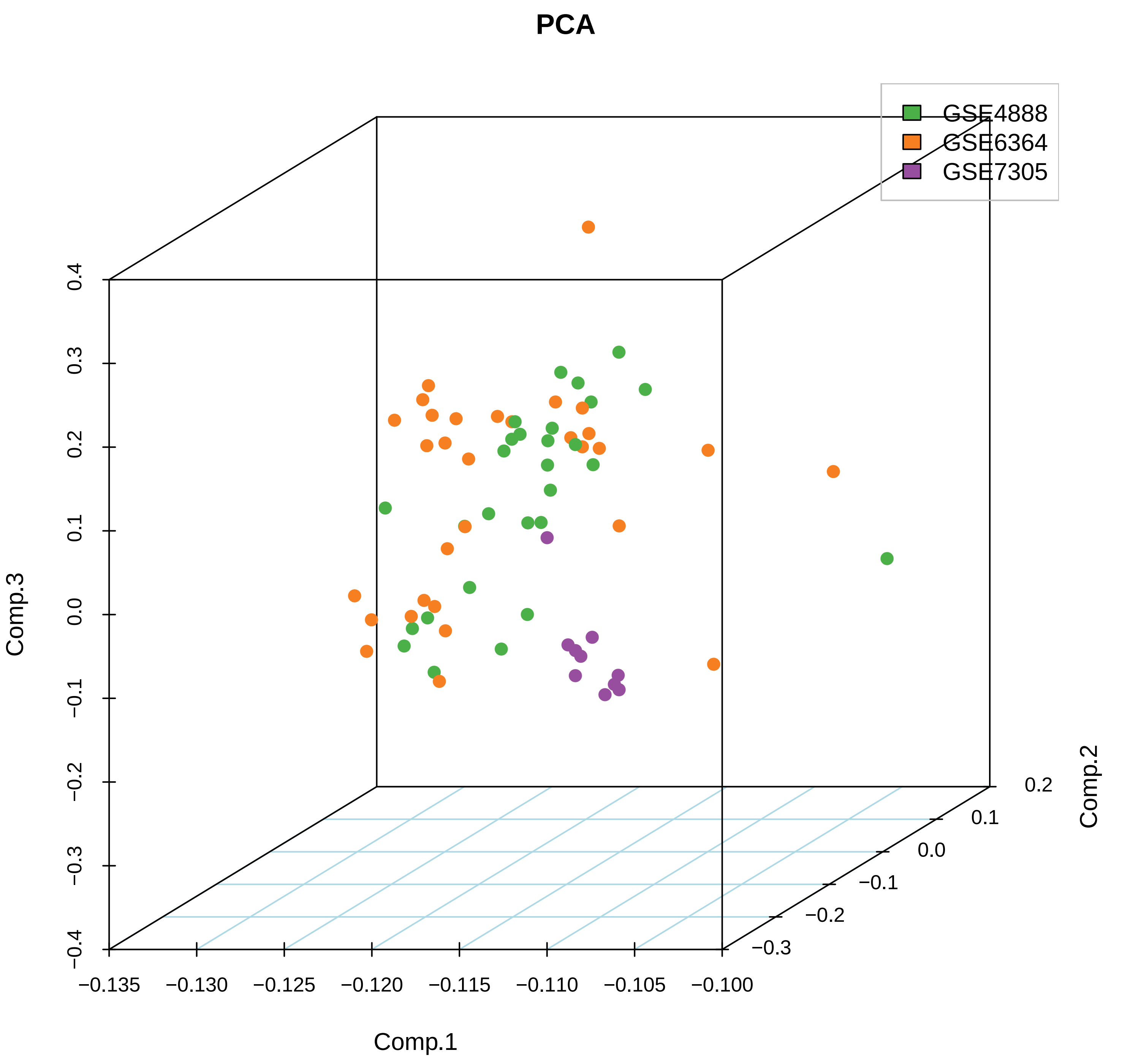

PCA
GSE4888
GSE6364
GSE7305
Comp.3
0.4
0.3
0.2
0.1
0.0
−0.1
−0.2
−0.3
−0.4
Comp.2
0.2
0.1
0.0
−0.1
−0.2
−0.3
−0.135
−0.130
−0.125
−0.120
−0.115
−0.110
−0.105
−0.100
Comp.1

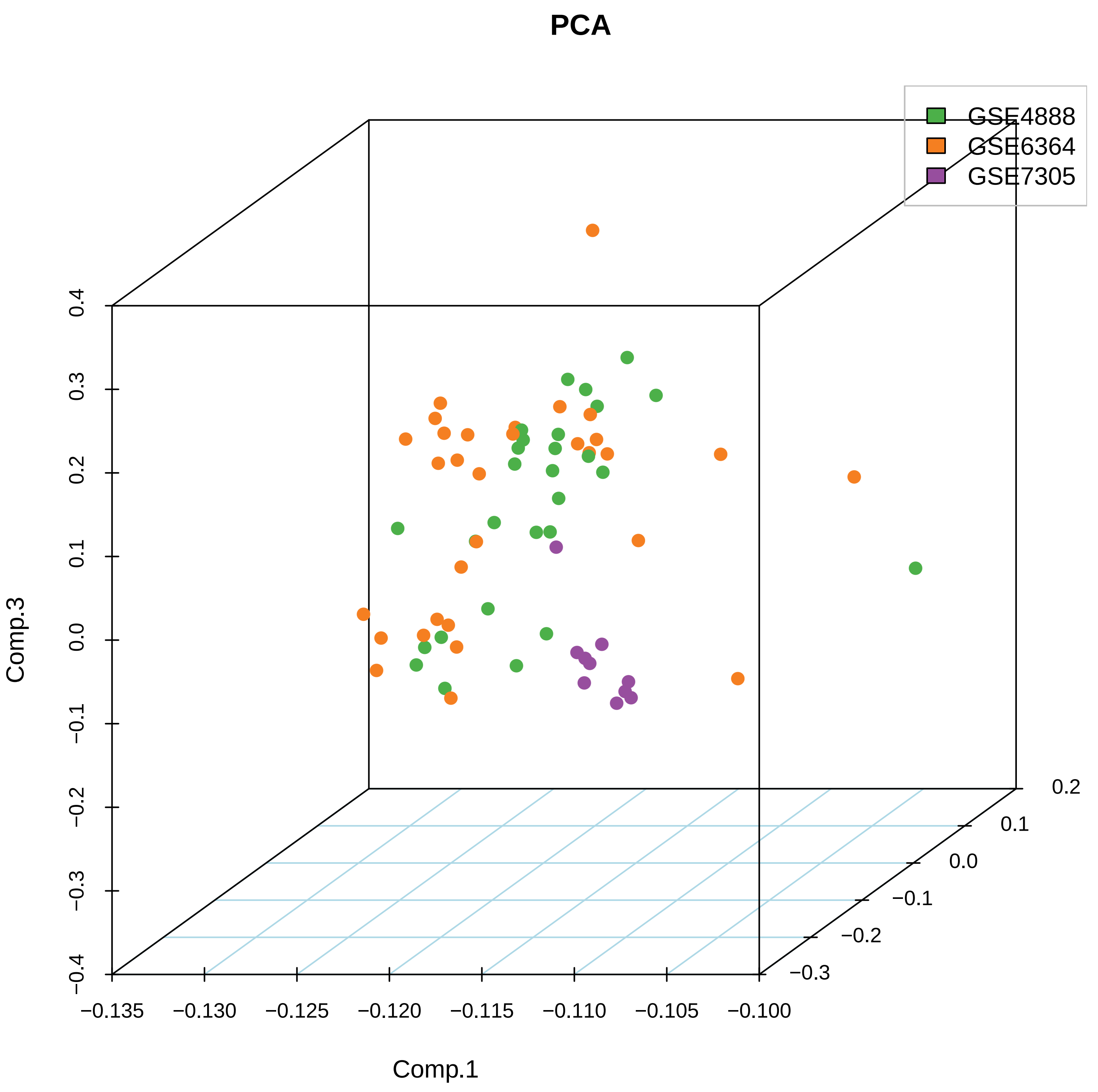
PCA
GSE4888
GSE6364
GSE7305
Comp.3
0.4
0.3
0.2
0.1
0.0
−0.1
−0.2
−0.3
−0.4
Comp.2
0.2
0.1
0.0
−0.1
−0.2
−0.3
−0.135
−0.130
−0.125
−0.120
−0.115
−0.110
−0.105
−0.100
Comp.1

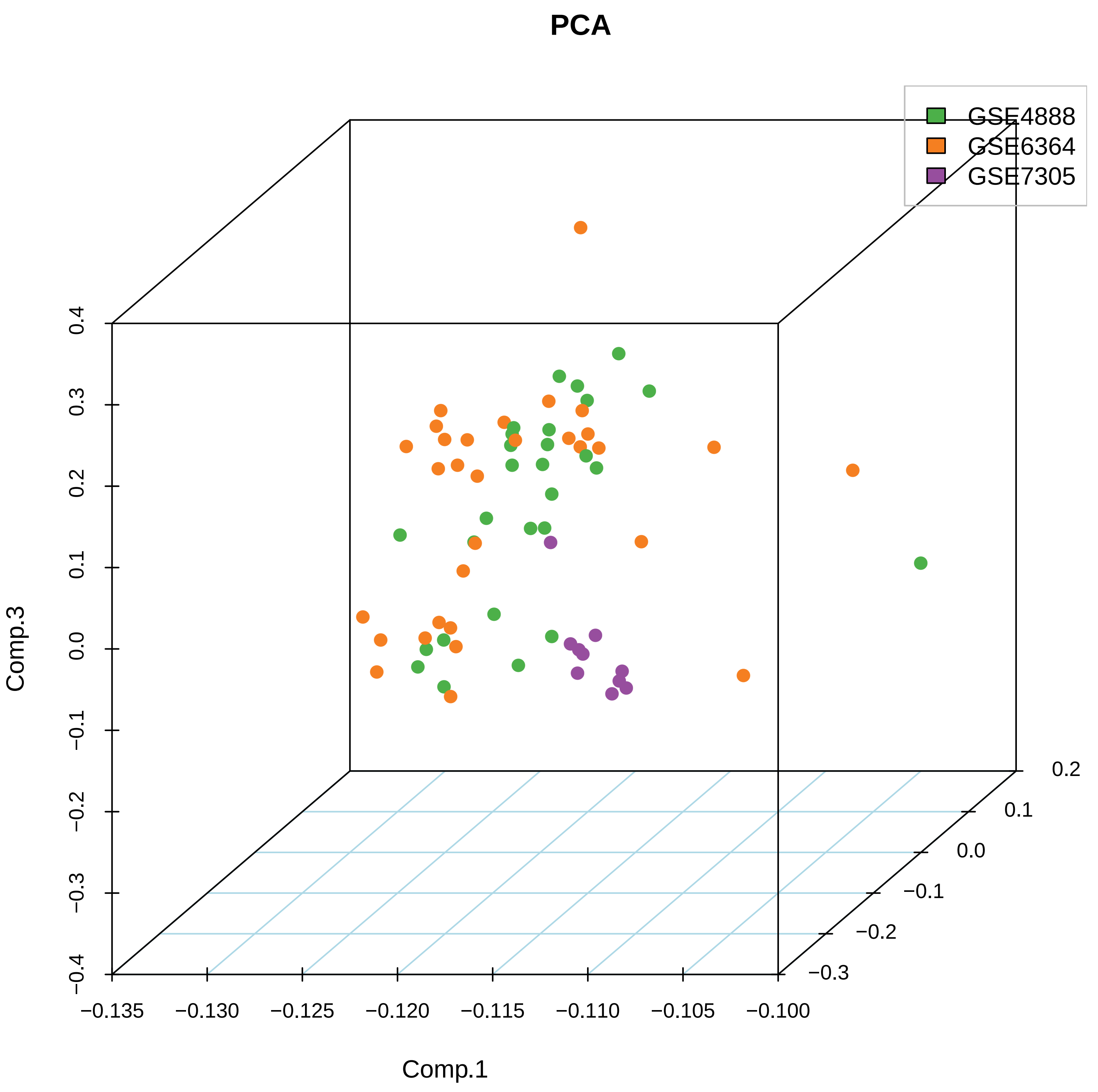
PCA
GSE4888
GSE6364
GSE7305
Comp.1
Comp.2
Comp.3
−0.135
−0.130
−0.125
−0.120
−0.115
−0.110
−0.105
−0.100
0.4
0.3
0.2
0.1
0.0
−0.1
−0.2
−0.3
−0.4
0.2
0.1
0.0
−0.1
−0.2
−0.3

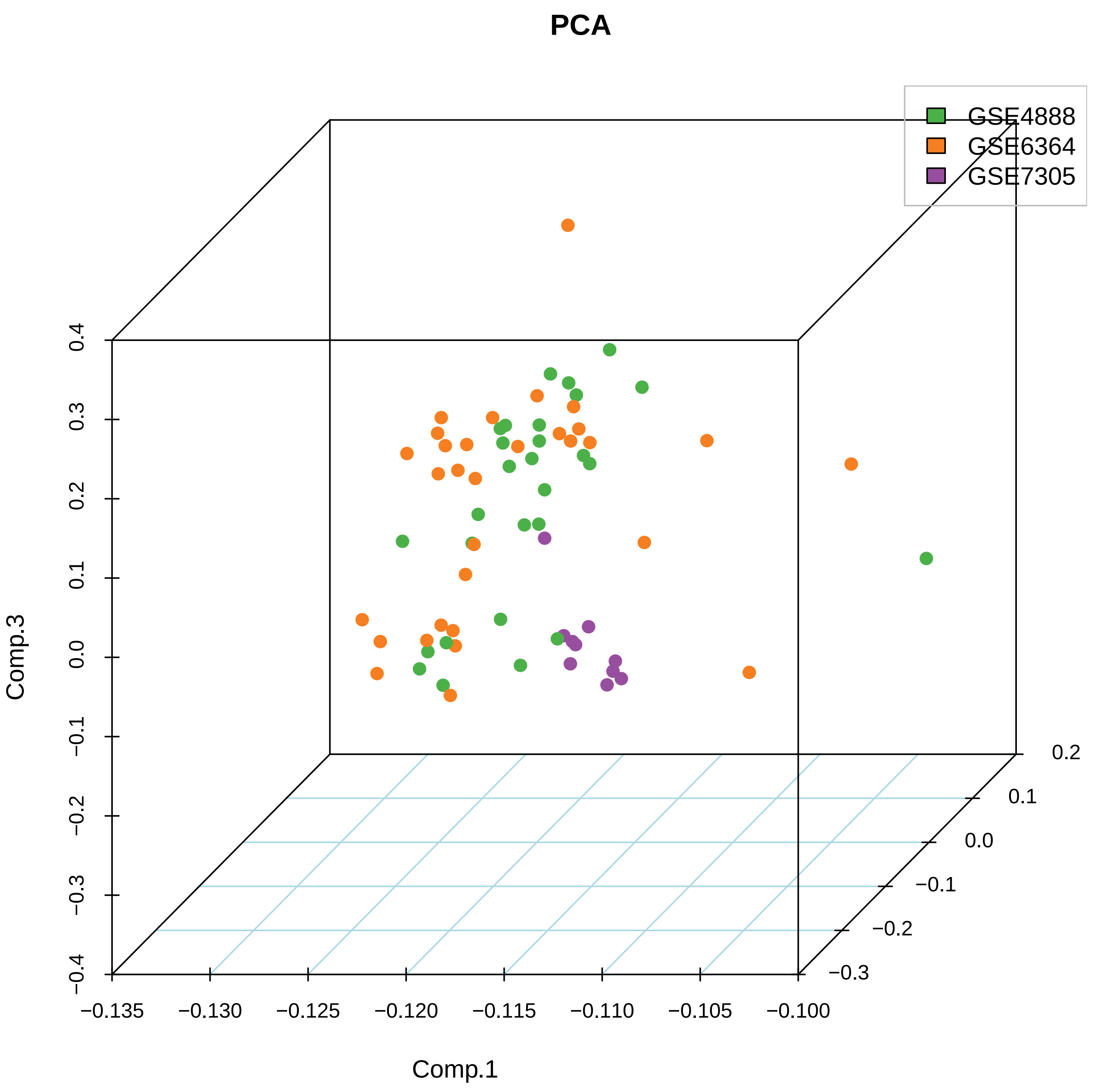
PCA
GSE4888
GSE6364
GSE7305
Comp.3
0.4
0.3
0.2
0.1
0.0
−0.1
−0.2
−0.3
−0.4
Comp.2
0.2
0.1
0.0
−0.1
−0.2
−0.3
Comp.1
−0.135
−0.130
−0.125
−0.120
−0.115
−0.110
−0.105
−0.100

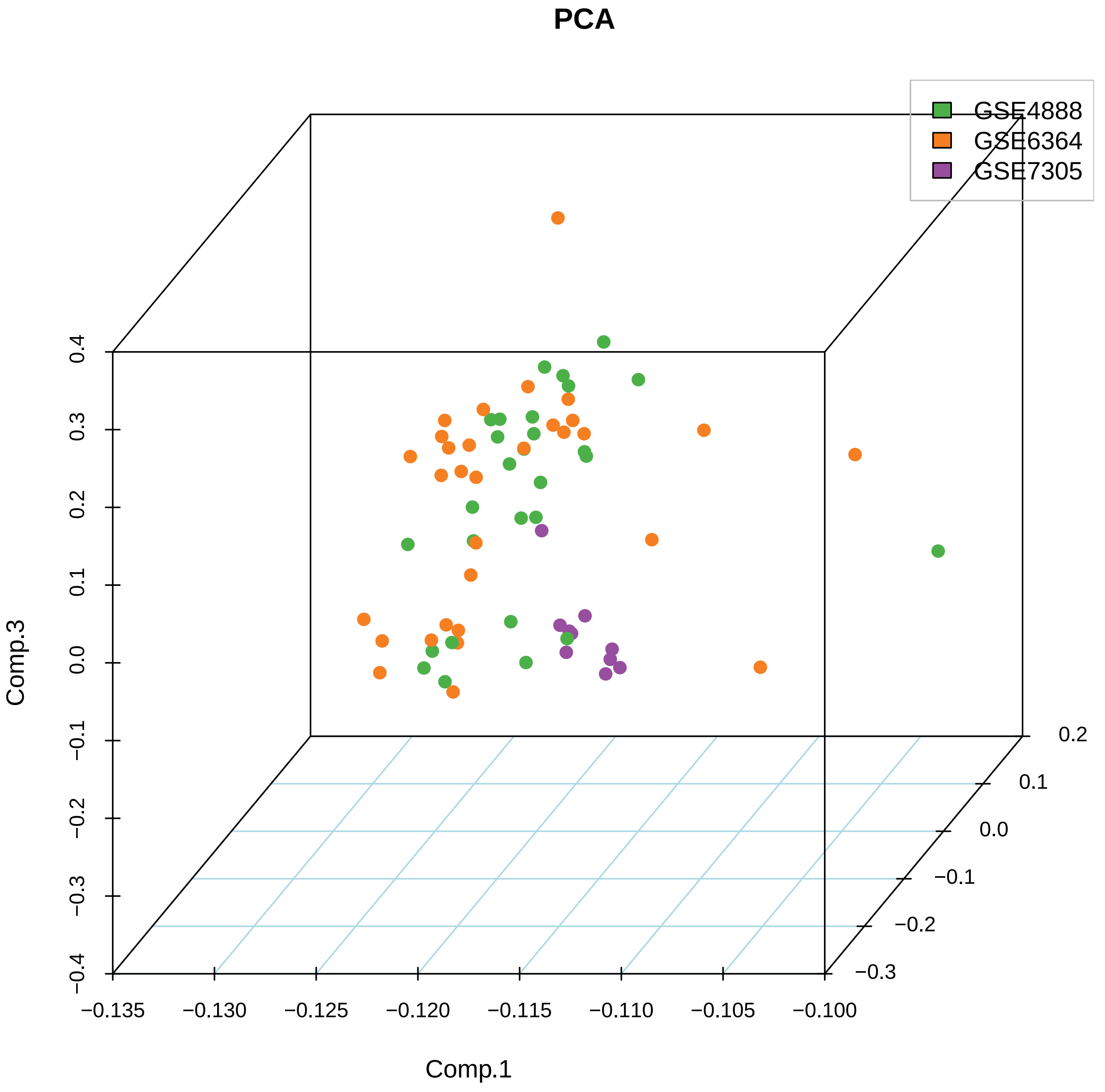
PCA
GSE4888
GSE6364
GSE7305
Comp.1
Comp.2
Comp.3
−0.135
−0.130
−0.125
−0.120
−0.115
−0.110
−0.105
−0.100
−0.3
−0.2
−0.1
0.0
0.1
0.2
−0.4
−0.3
−0.2
−0.1
0.0
0.1
0.2
0.3
0.4

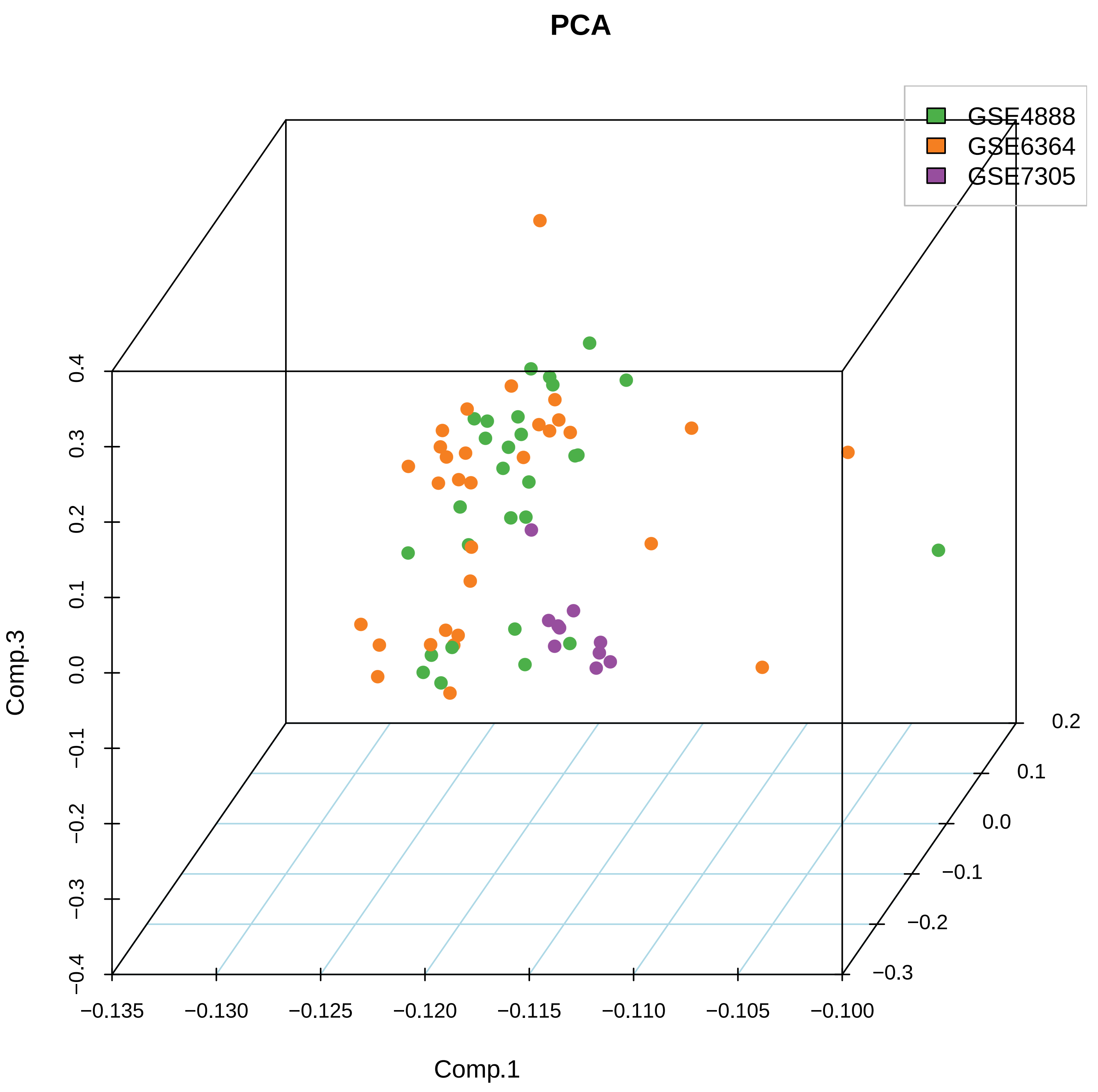

PCA
GSE4888
GSE6364
GSE7305
Comp.1
Comp.2
Comp.3
−0.135
−0.130
−0.125
−0.120
−0.115
−0.110
−0.105
−0.100
−0.3
−0.2
−0.1
0.0
0.1
0.2
−0.4
−0.3
−0.2
−0.1
0.0
0.1
0.2
0.3
0.4

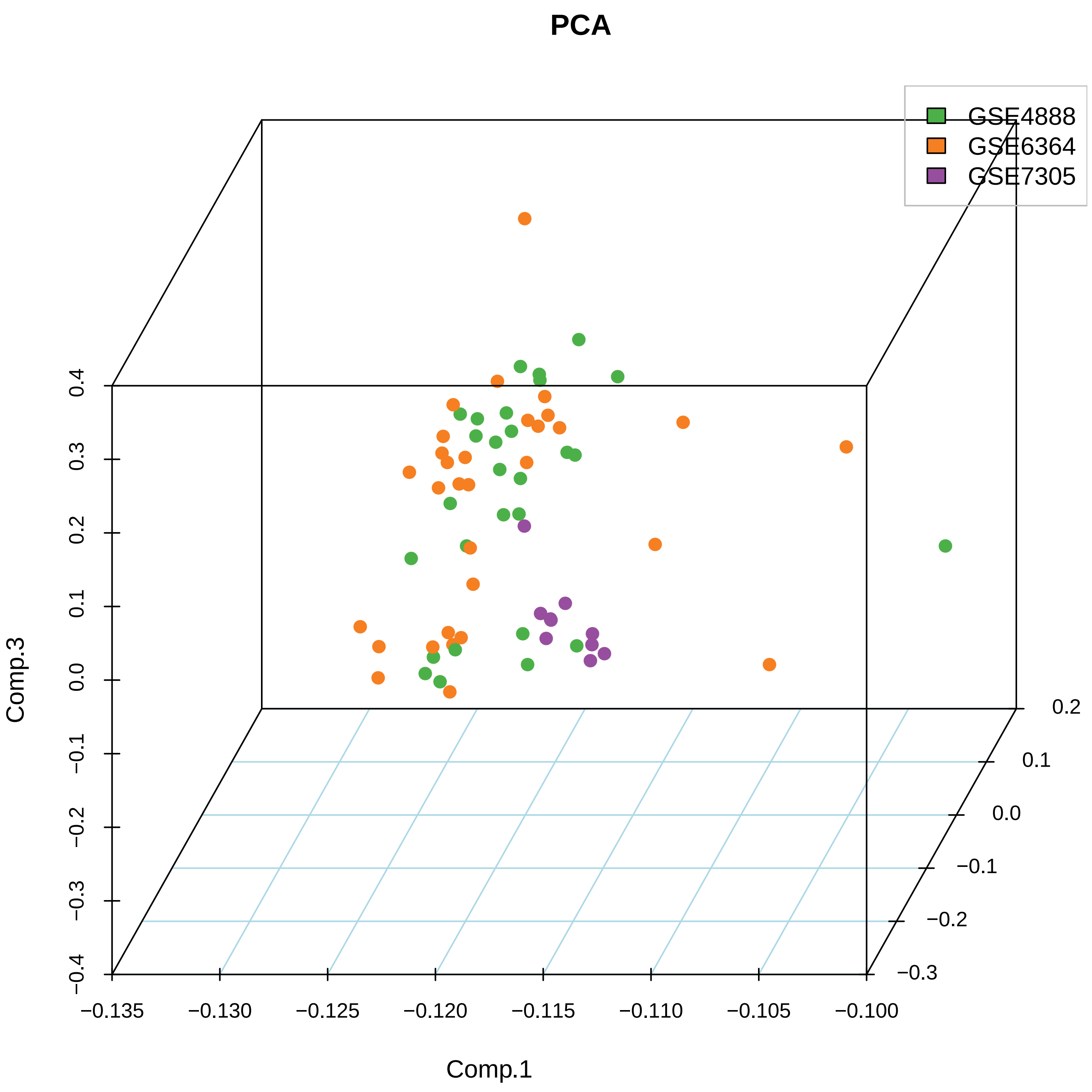

PCA
GSE4888
GSE6364
GSE7305
Comp.1
Comp.2
Comp.3
−0.135
−0.130
−0.125
−0.120
−0.115
−0.110
−0.105
−0.100
−0.3
−0.2
−0.1
0.0
0.1
0.2
−0.4
−0.3
−0.2
−0.1
0.0
0.1
0.2
0.3
0.4

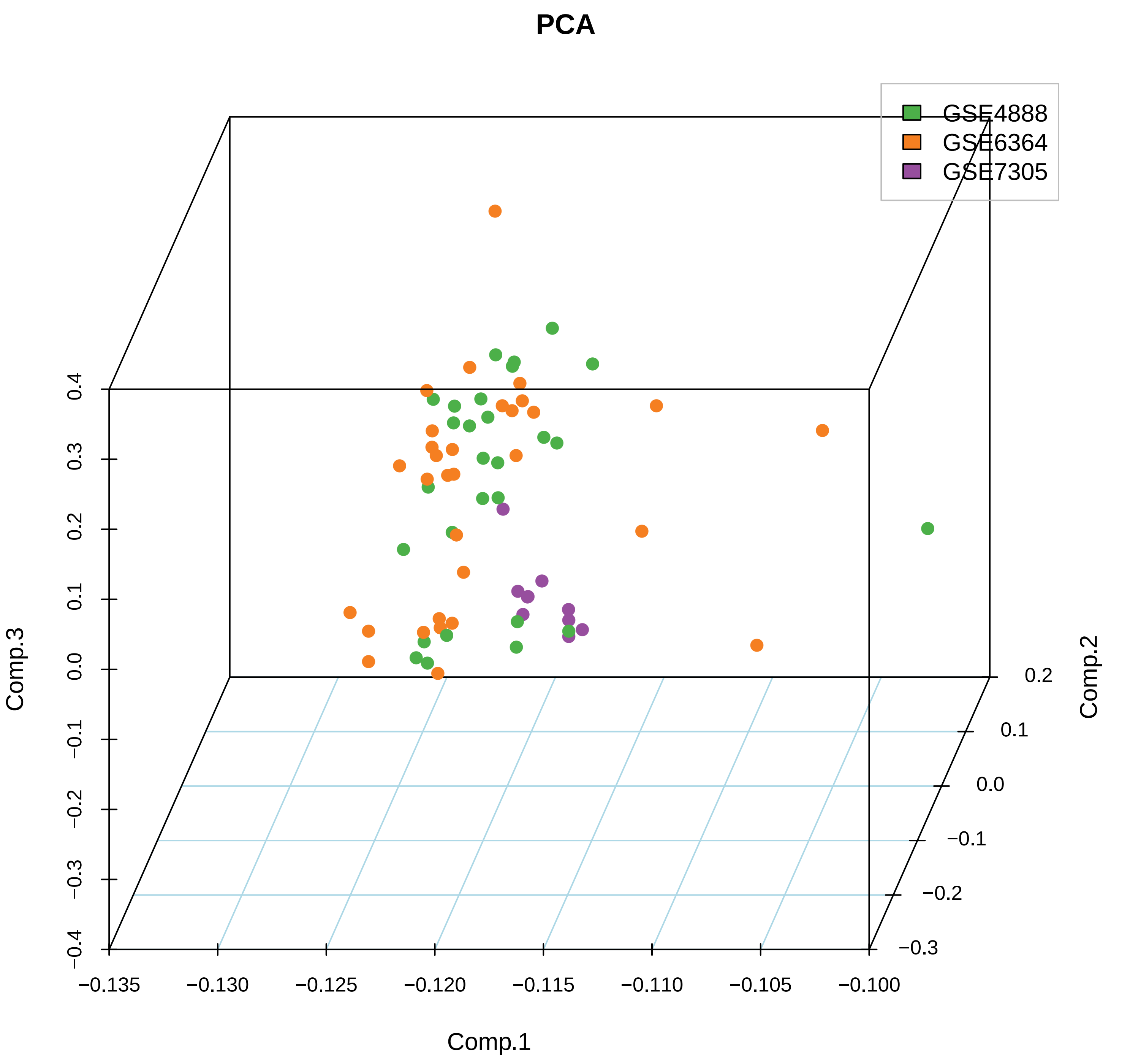
PCA
GSE4888
GSE6364
GSE7305
Comp.1
Comp.2
Comp.3
−0.135
−0.130
−0.125
−0.120
−0.115
−0.110
−0.105
−0.100
−0.3
−0.2
−0.1
0.0
0.1
0.2
−0.4
−0.3
−0.2
−0.1
0.0
0.1
0.2
0.3
0.4

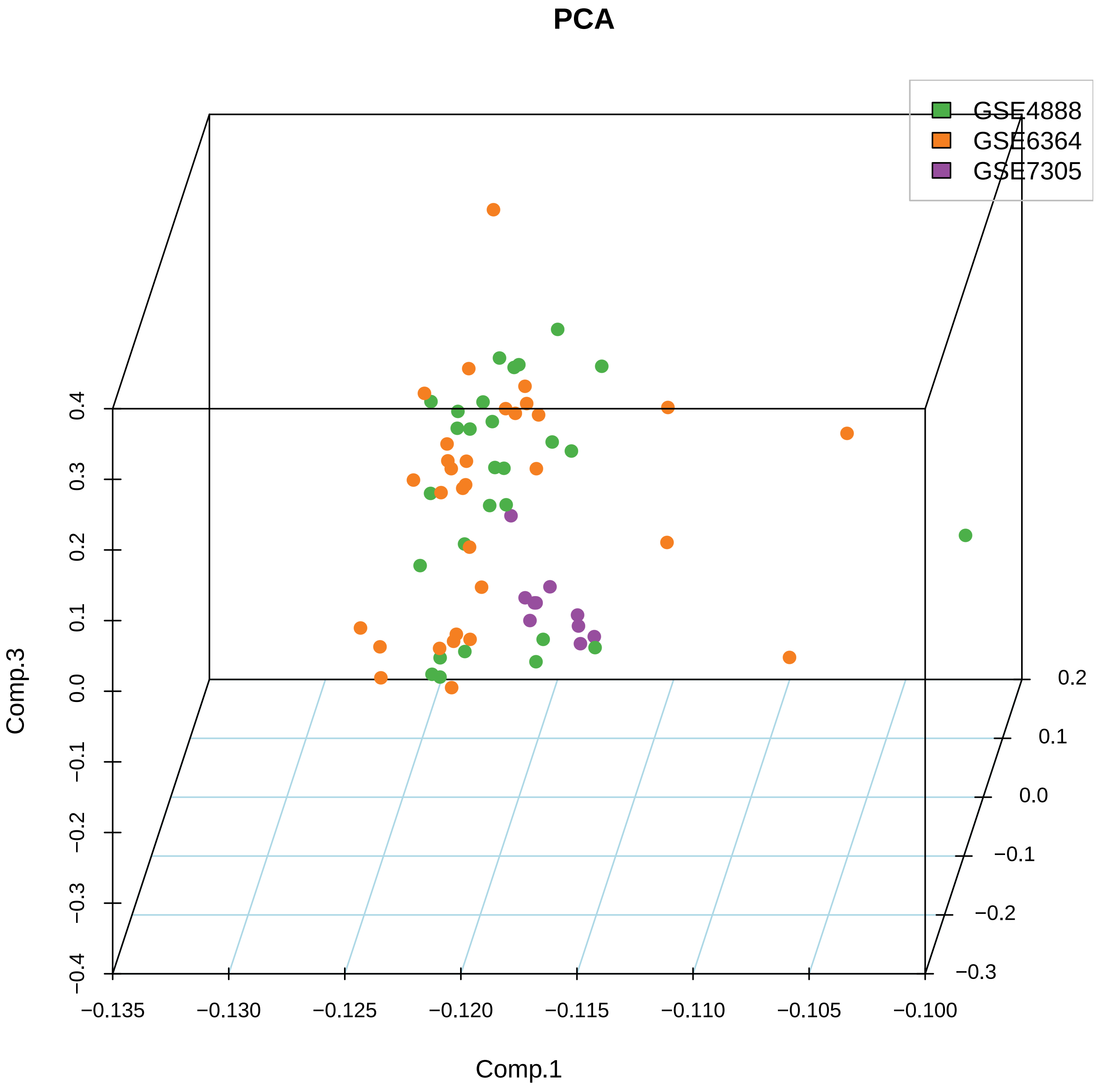

PCA
GSE4888
GSE6364
GSE7305
Comp.1
Comp.2
Comp.3
−0.135
−0.130
−0.125
−0.120
−0.115
−0.110
−0.105
−0.100
−0.3
−0.2
−0.1
0.0
0.1
0.2
−0.4
−0.3
−0.2
−0.1
0.0
0.1
0.2
0.3
0.4

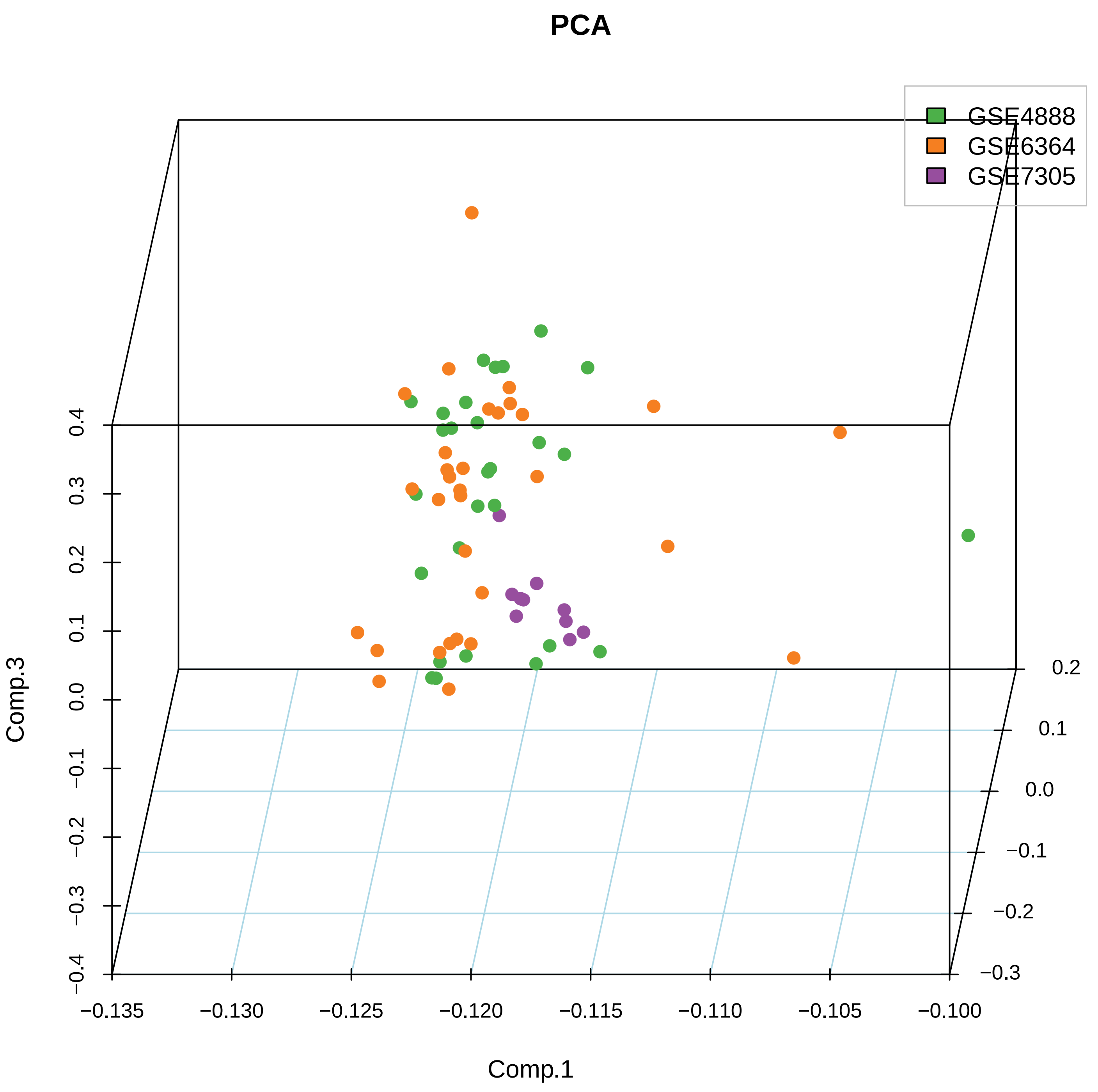
PCA
GSE4888
GSE6364
GSE7305
Comp.3
0.4
0.3
0.2
0.1
0.0
−0.1
−0.2
−0.3
−0.4
Comp.2
0.2
0.1
0.0
−0.1
−0.2
−0.3
Comp.1
−0.135
−0.130
−0.125
−0.120
−0.115
−0.110
−0.105
−0.100

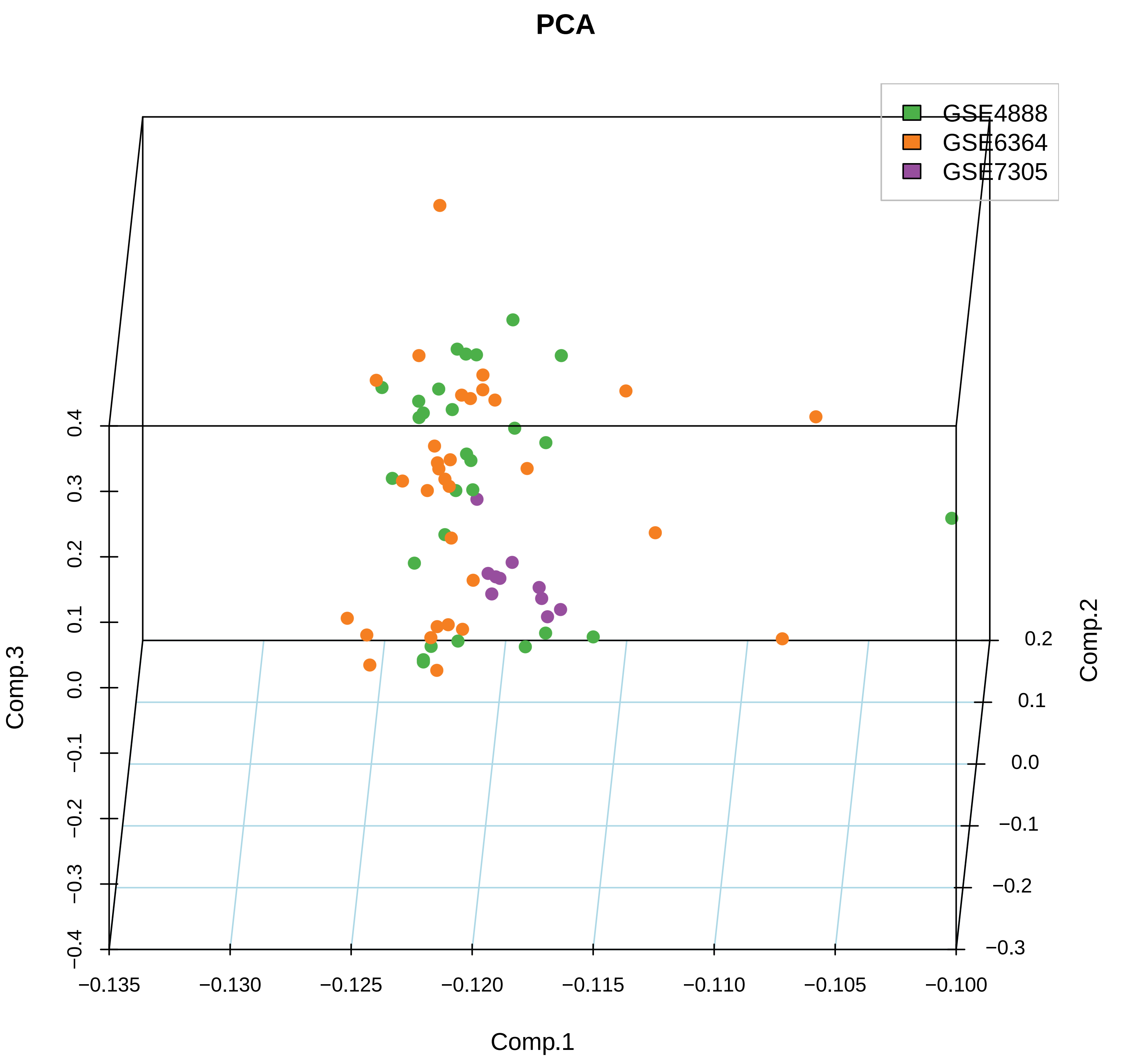

PCA
GSE4888
GSE6364
GSE7305
Comp.1
Comp.2
Comp.3
−0.135
−0.130
−0.125
−0.120
−0.115
−0.110
−0.105
−0.100
0.2
0.1
0.0
−0.1
−0.2
−0.3
0.4
0.3
0.2
0.1
0.0
−0.1
−0.2
−0.3
−0.4

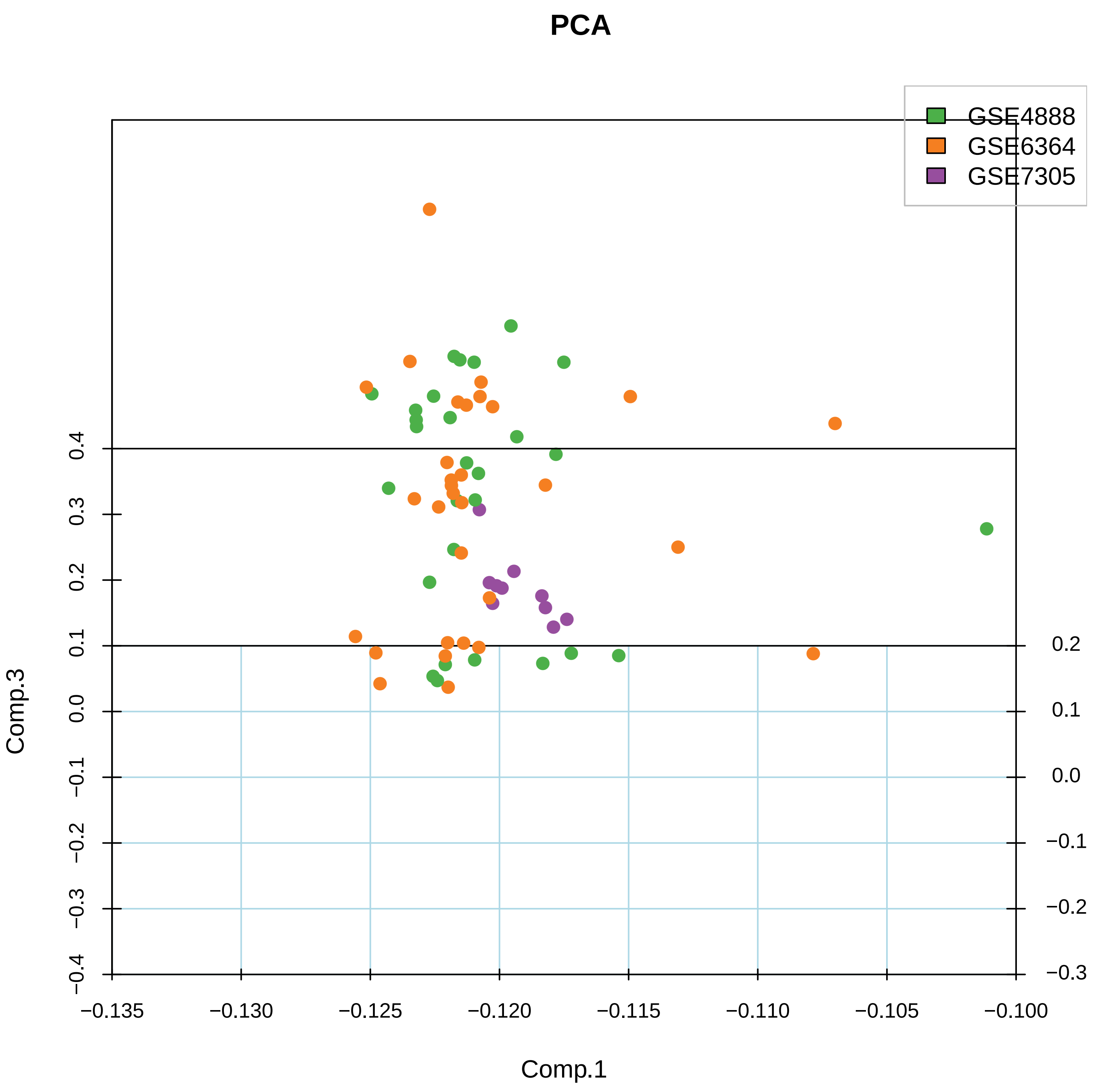
PCA
GSE4888
GSE6364
GSE7305
Comp.1
Comp.2
Comp.3
−0.135
−0.130
−0.125
−0.120
−0.115
−0.110
−0.105
−0.100
−0.4
−0.3
−0.2
−0.1
0.0
0.1
0.2
0.3
0.4
−0.3
−0.2
−0.1
0.0
0.1
0.2

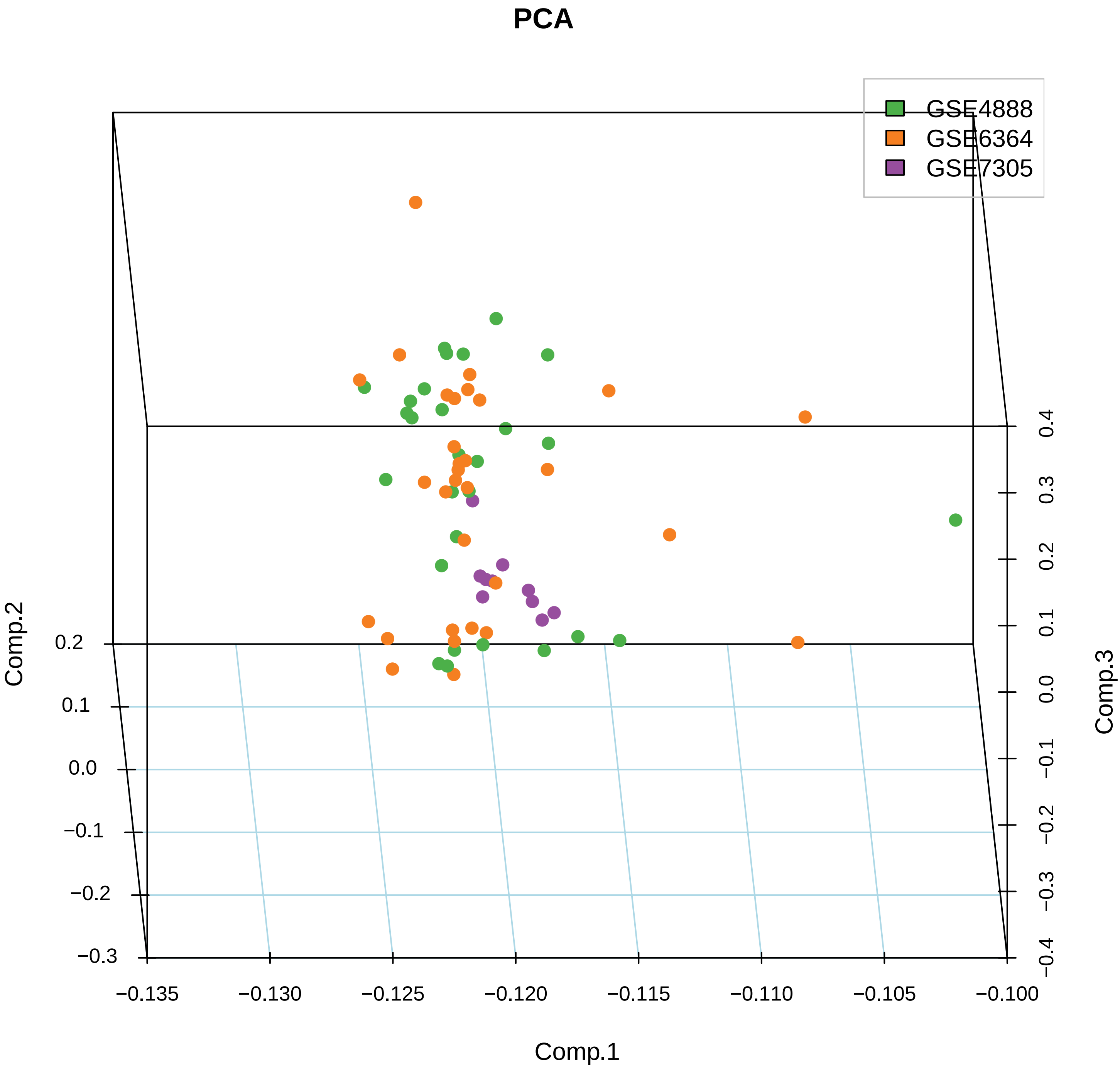
PCA
GSE4888
GSE6364
GSE7305
Comp.1
Comp.2
Comp.3
−0.135
−0.130
−0.125
−0.120
−0.115
−0.110
−0.105
−0.100
0.2
0.1
0.0
−0.1
−0.2
−0.3
0.4
0.3
0.2
0.1
0.0
−0.1
−0.2
−0.3
−0.4

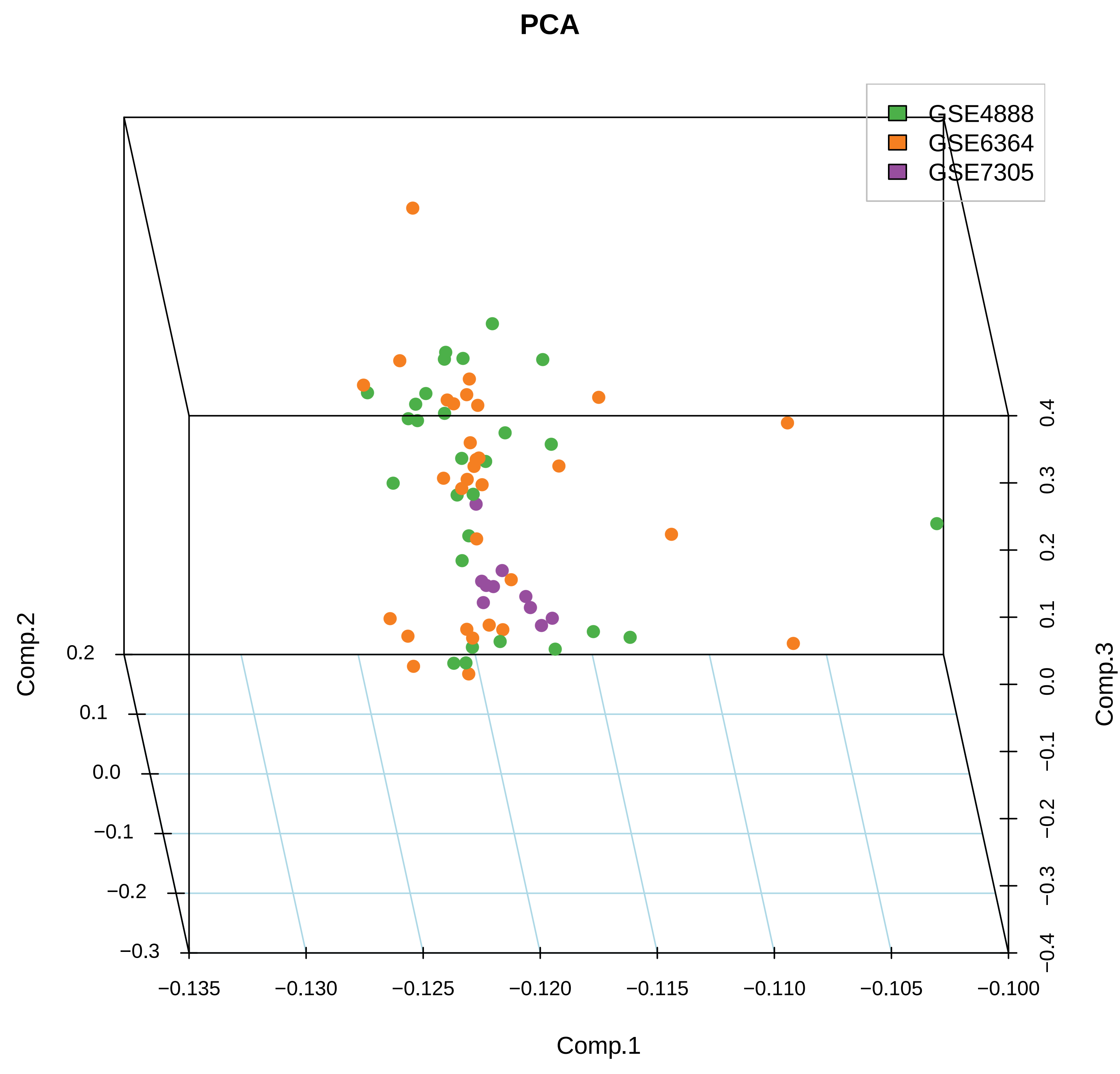
PCA
GSE4888
GSE6364
GSE7305
Comp.1
Comp.2
Comp.3
−0.135
−0.130
−0.125
−0.120
−0.115
−0.110
−0.105
−0.100
0.2
0.1
0.0
−0.1
−0.2
−0.3
0.4
0.3
0.2
0.1
0.0
−0.1
−0.2
−0.3
−0.4

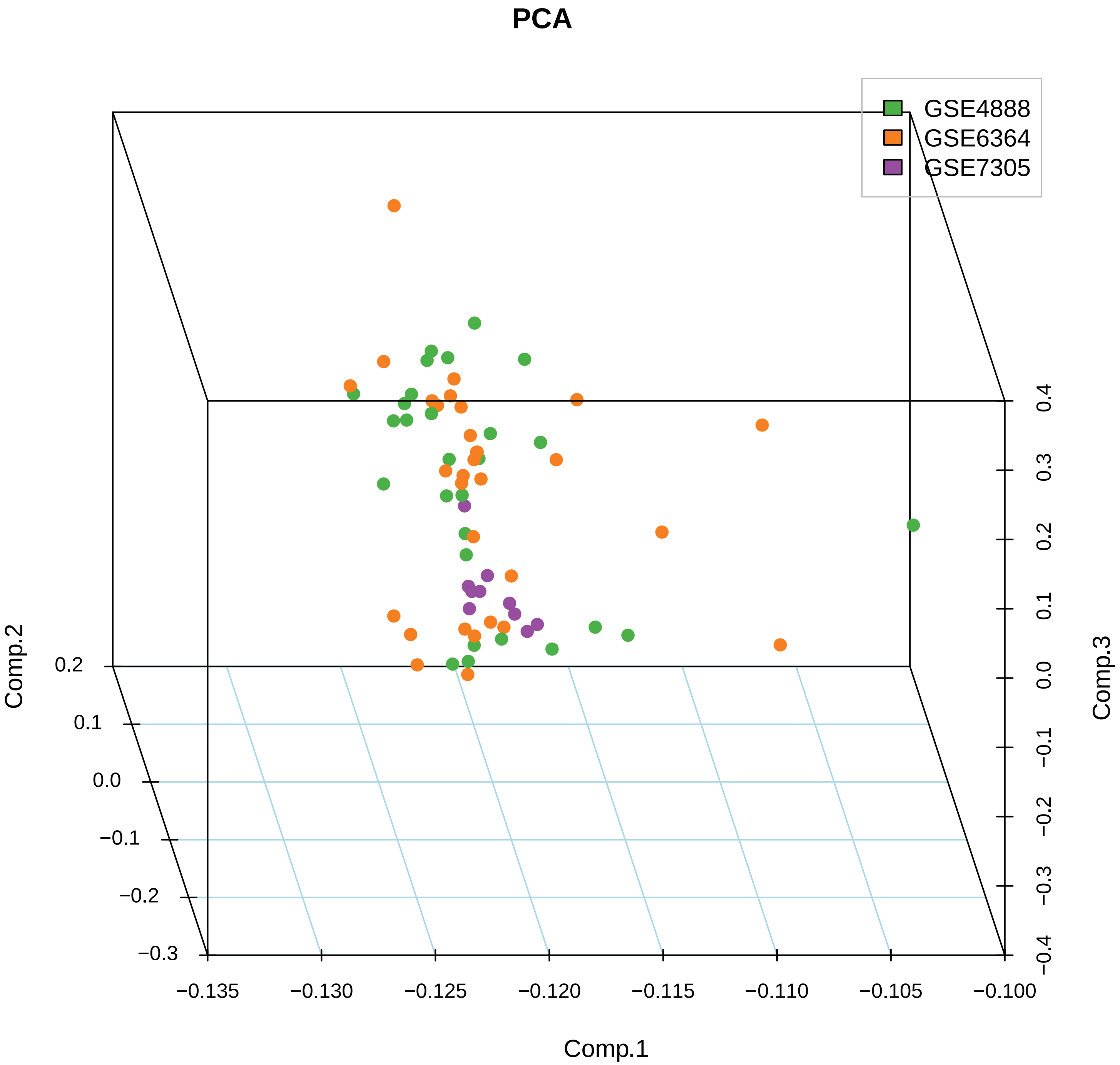
PCA
GSE4888
GSE6364
GSE7305
Comp.1
Comp.2
Comp.3
−0.135
−0.130
−0.125
−0.120
−0.115
−0.110
−0.105
−0.100
0.2
0.1
0.0
−0.1
−0.2
−0.3
0.4
0.3
0.2
0.1
0.0
−0.1
−0.2
−0.3
−0.4

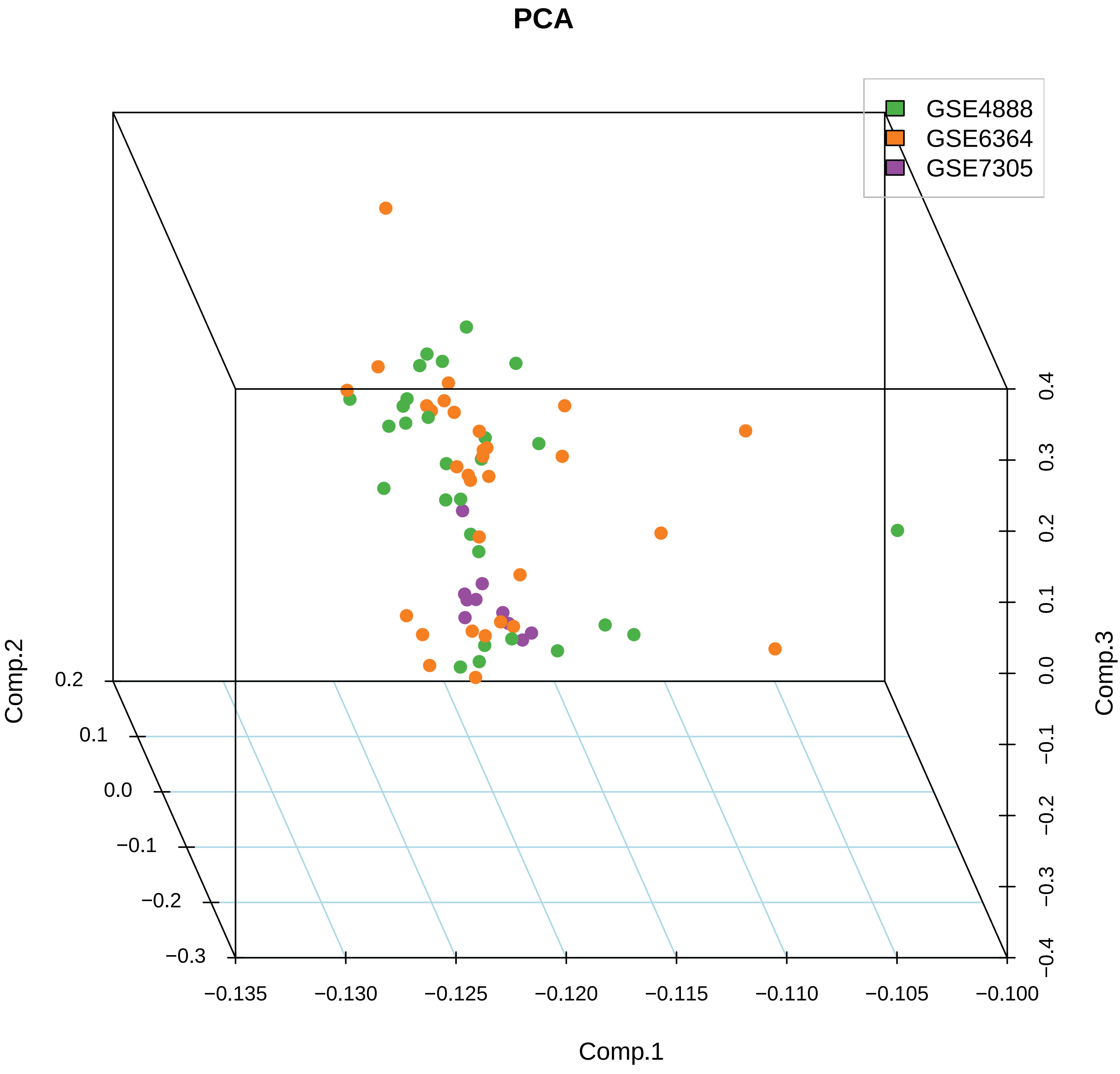

PCA
GSE4888
GSE6364
GSE7305
Comp.1
Comp.2
Comp.3
−0.135
−0.130
−0.125
−0.120
−0.115
−0.110
−0.105
−0.100
0.2
0.1
0.0
−0.1
−0.2
−0.3
0.4
0.3
0.2
0.1
0.0
−0.1
−0.2
−0.3
−0.4

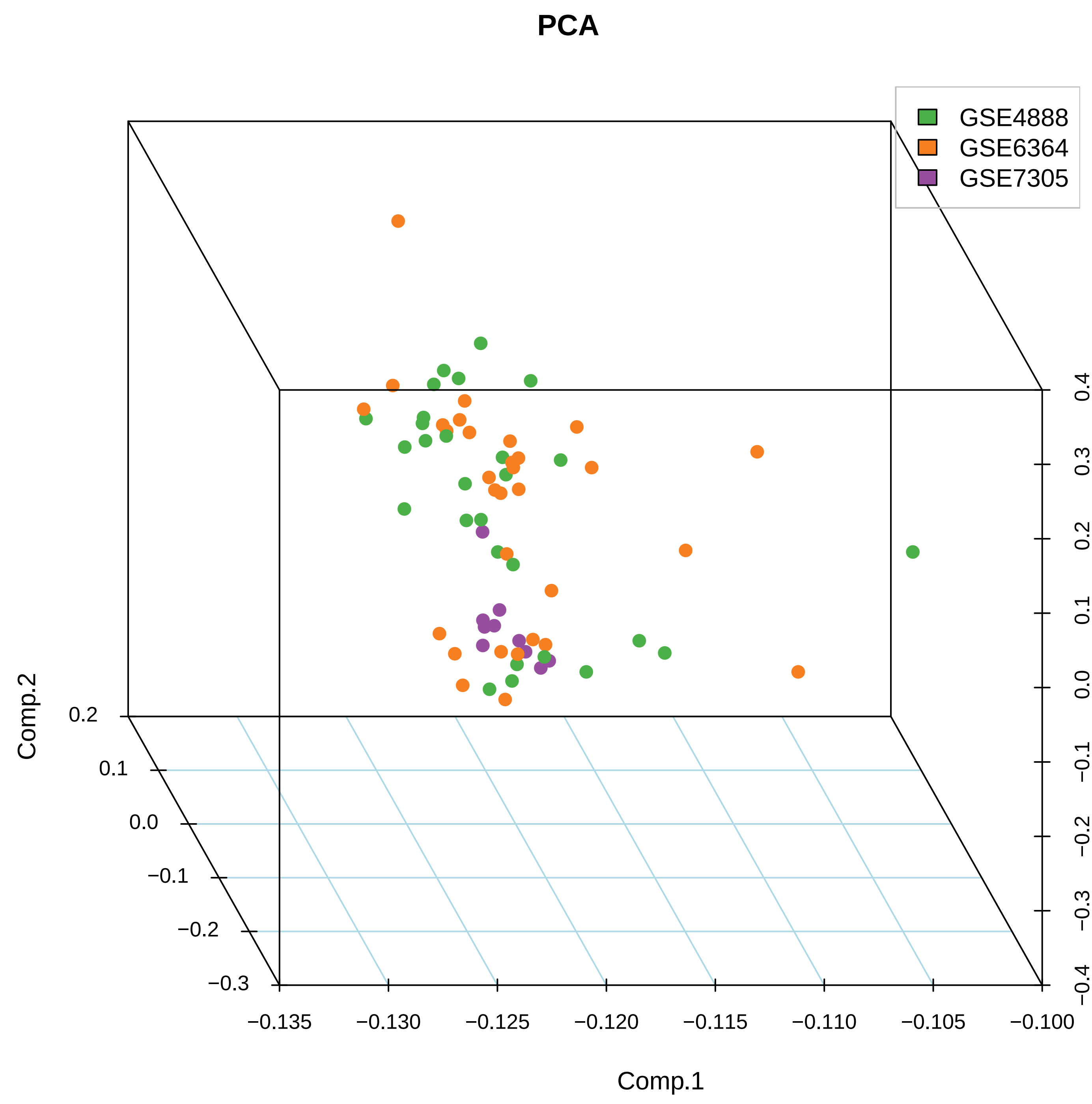
PCA
GSE4888
GSE6364
GSE7305
Comp.1
Comp.2
Comp.3
−0.135
−0.130
−0.125
−0.120
−0.115
−0.110
−0.105
−0.100
0.2
0.1
0.0
−0.1
−0.2
−0.3
0.4
0.3
0.2
0.1
0.0
−0.1
−0.2
−0.3
−0.4

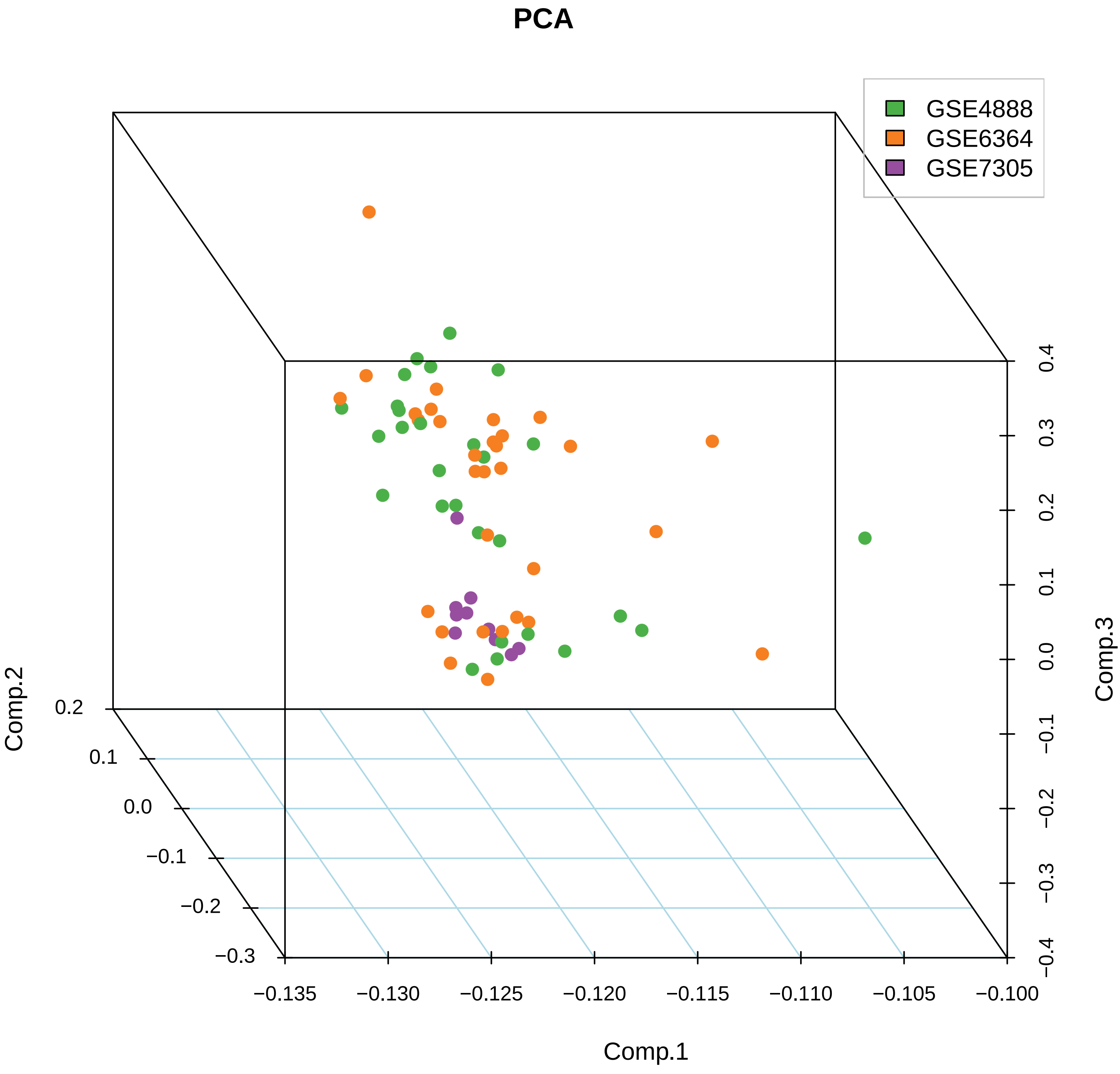

PCA
GSE4888
GSE6364
GSE7305
Comp.1
Comp.2
Comp.3
−0.135
−0.130
−0.125
−0.120
−0.115
−0.110
−0.105
−0.100
0.2
0.1
0.0
−0.1
−0.2
−0.3
0.4
0.3
0.2
0.1
0.0
−0.1
−0.2
−0.3
−0.4

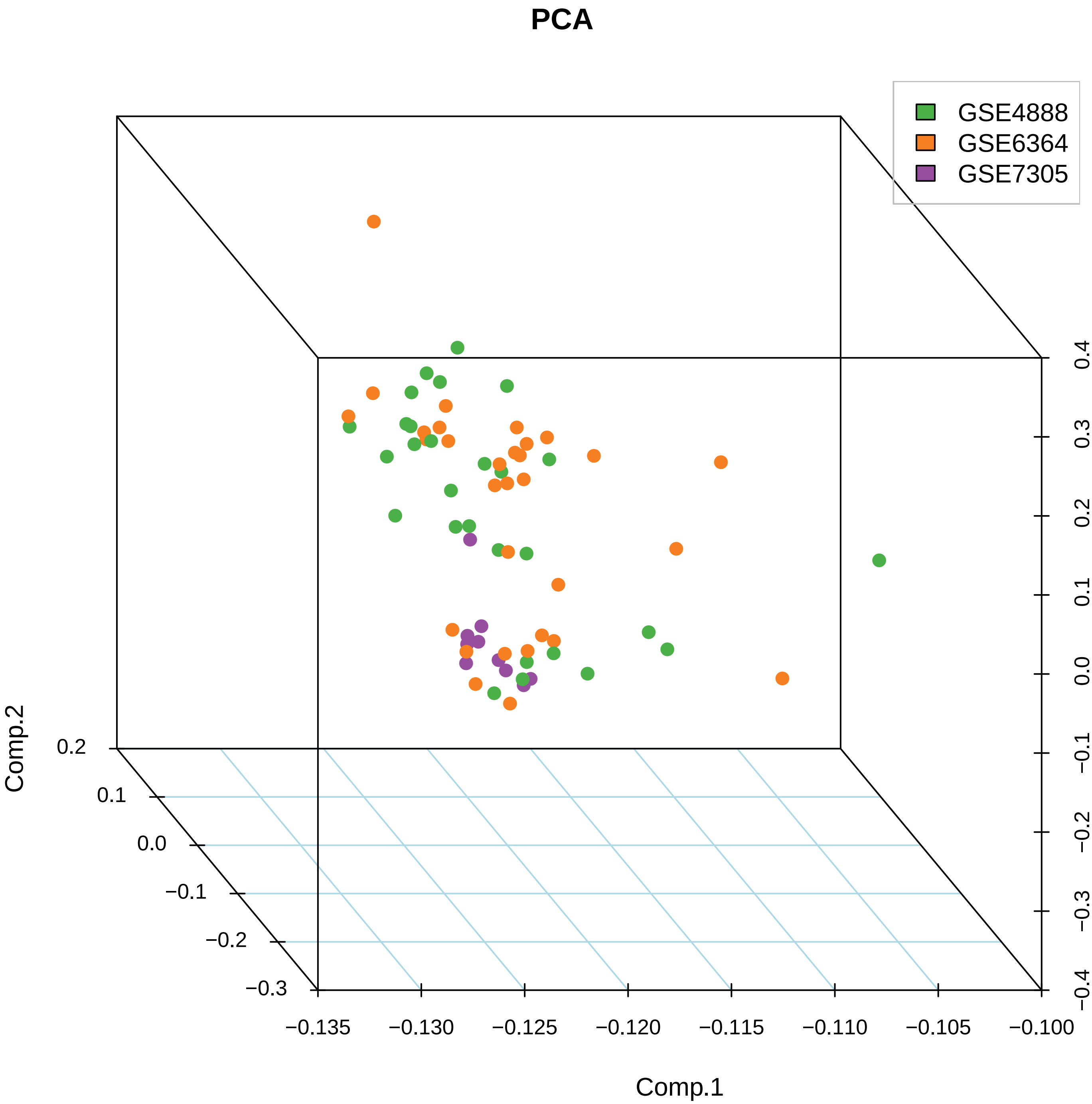

PCA
GSE4888
GSE6364
GSE7305
Comp.1
Comp.2
Comp.3
−0.135
−0.130
−0.125
−0.120
−0.115
−0.110
−0.105
−0.100
0.2
0.1
0.0
−0.1
−0.2
−0.3
0.4
0.3
0.2
0.1
0.0
−0.1
−0.2
−0.3
−0.4

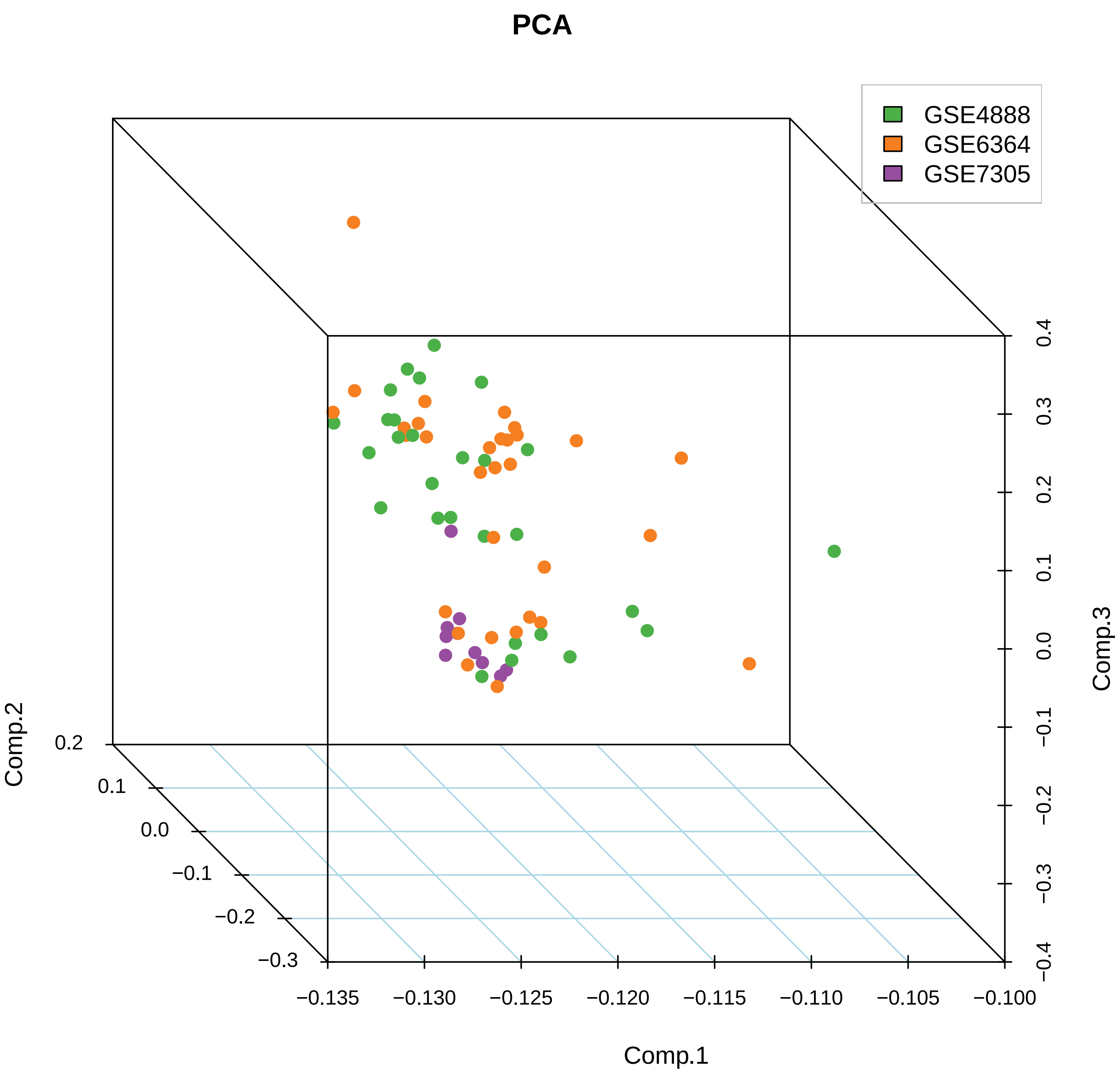
PCA
GSE4888
GSE6364
GSE7305
Comp.1
Comp.2
Comp.3
−0.135
−0.130
−0.125
−0.120
−0.115
−0.110
−0.105
−0.100
0.2
0.1
0.0
−0.1
−0.2
−0.3
0.4
0.3
0.2
0.1
0.0
−0.1
−0.2
−0.3
−0.4

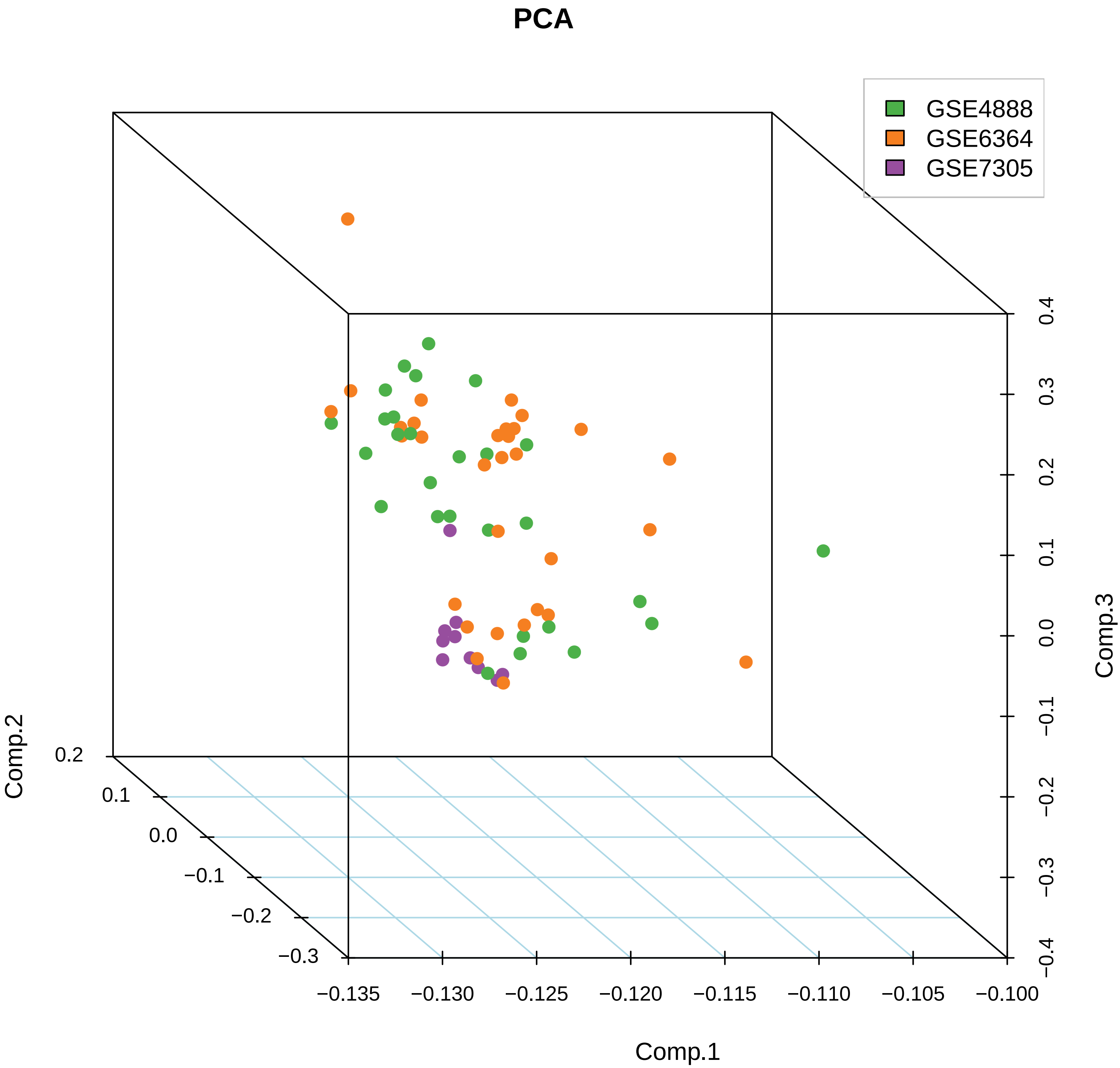
PCA
GSE4888
GSE6364
GSE7305
Comp.1
Comp.2
Comp.3
−0.135
−0.130
−0.125
−0.120
−0.115
−0.110
−0.105
−0.100
0.2
0.1
0.0
−0.1
−0.2
−0.3
0.4
0.3
0.2
0.1
0.0
−0.1
−0.2
−0.3
−0.4

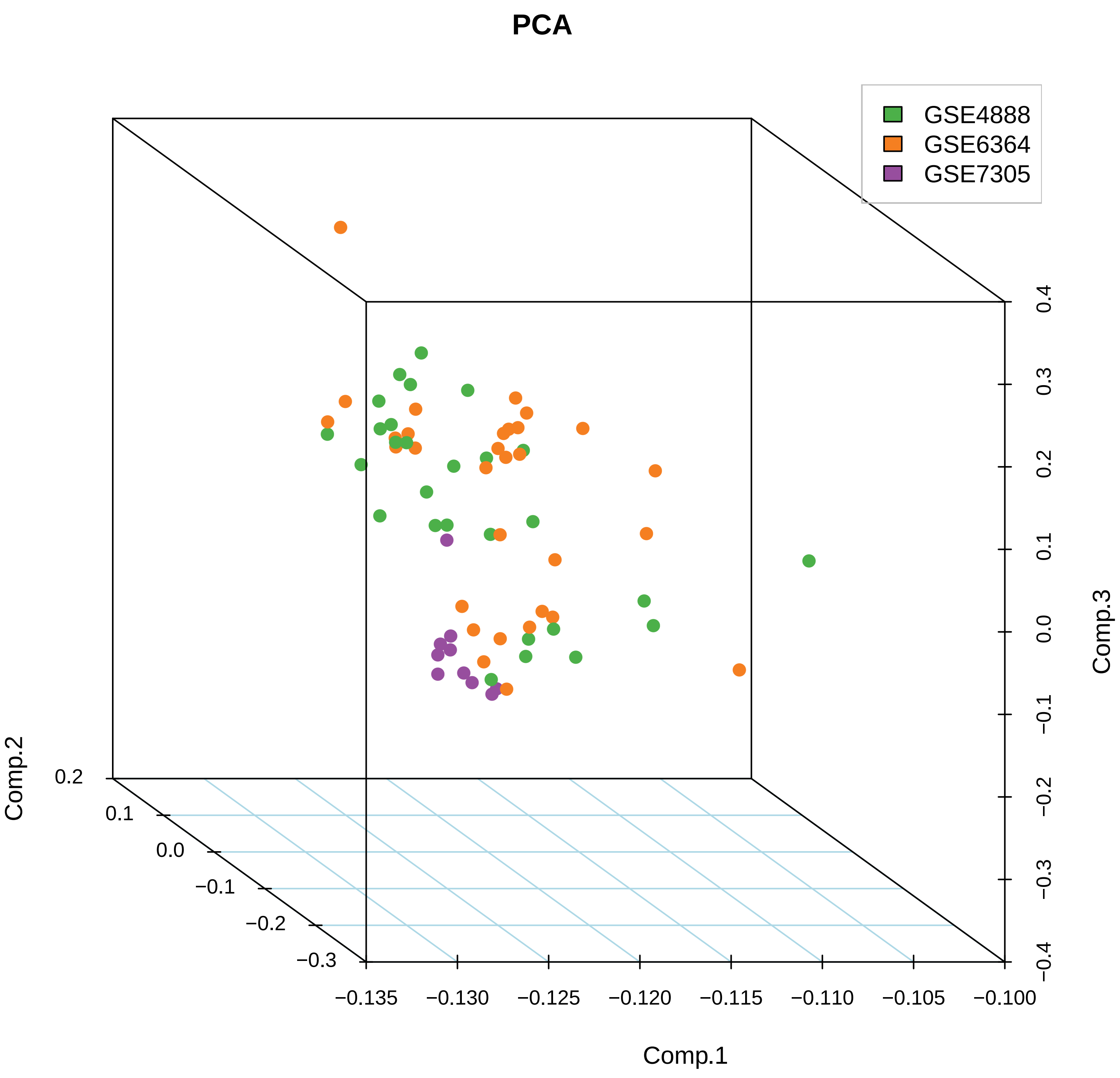

PCA
GSE4888
GSE6364
GSE7305
Comp.1
Comp.2
Comp.3
−0.135
−0.130
−0.125
−0.120
−0.115
−0.110
−0.105
−0.100
0.2
0.1
0.0
−0.1
−0.2
−0.3
0.4
0.3
0.2
0.1
0.0
−0.1
−0.2
−0.3
−0.4

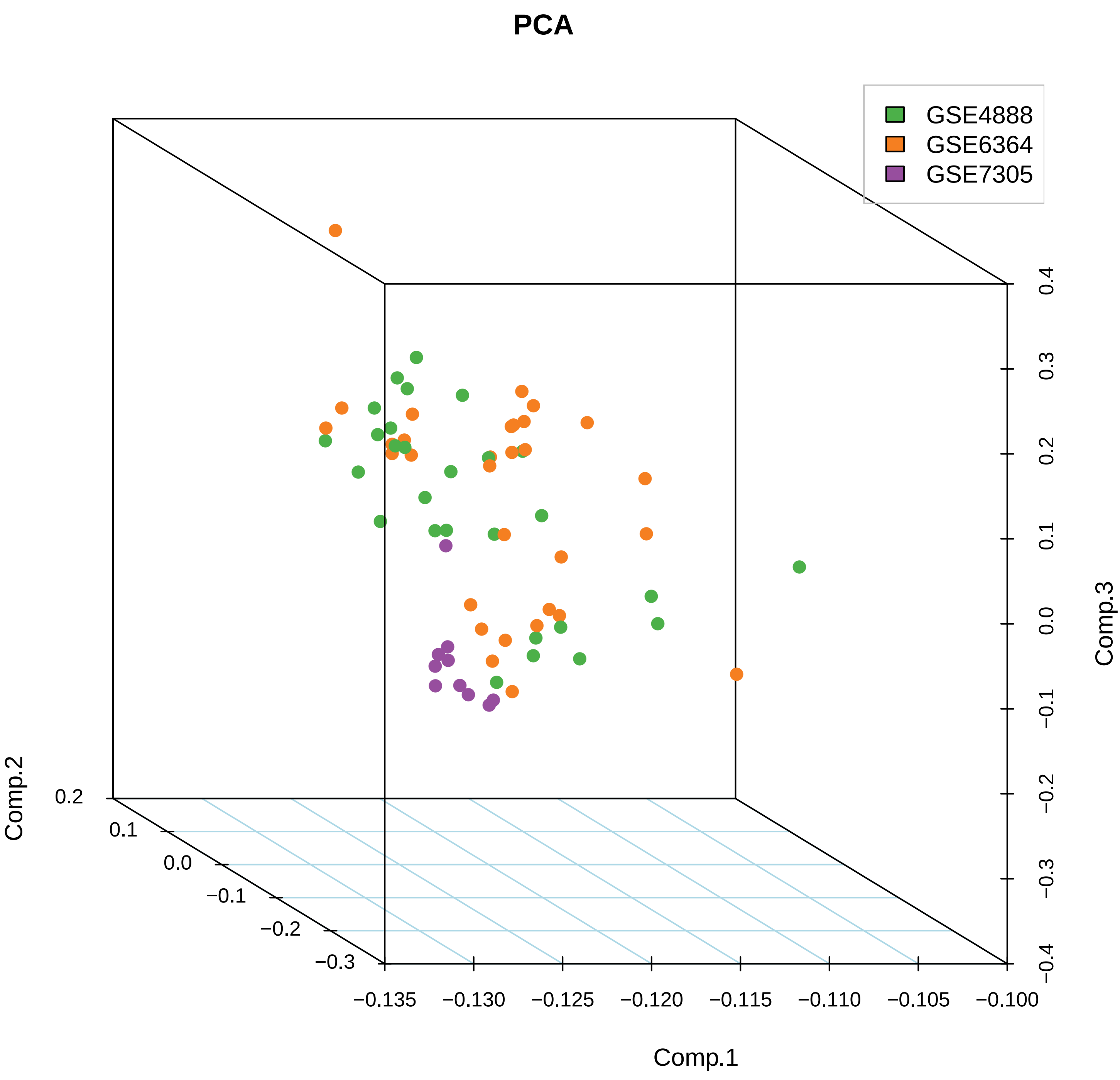

PCA
GSE4888
GSE6364
GSE7305
Comp.1
Comp.2
Comp.3
−0.135
−0.130
−0.125
−0.120
−0.115
−0.110
−0.105
−0.100
0.2
0.1
0.0
−0.1
−0.2
−0.3
0.4
0.3
0.2
0.1
0.0
−0.1
−0.2
−0.3
−0.4

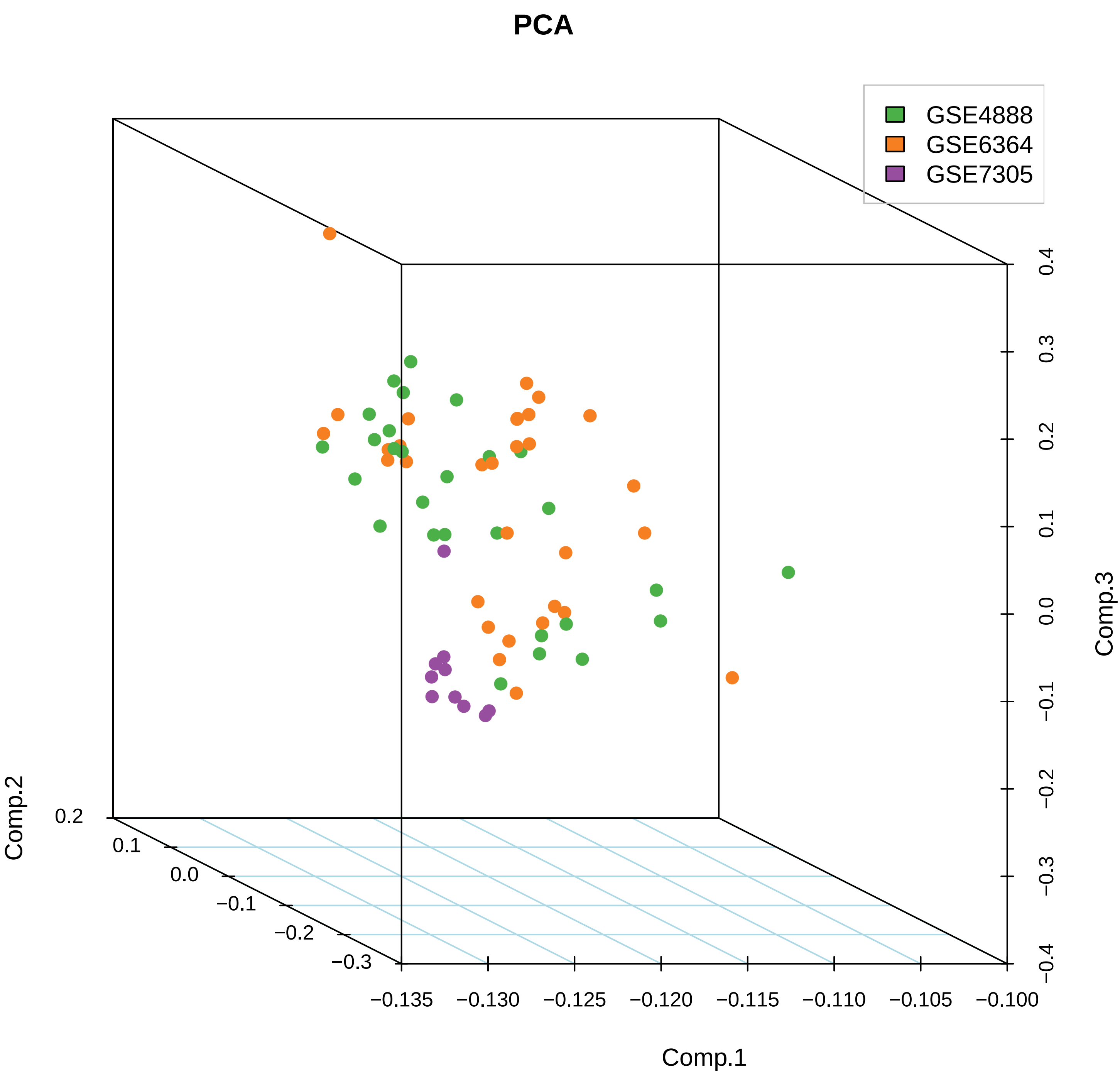

PCA
GSE4888
GSE6364
GSE7305
Comp.1
Comp.2
Comp.3
−0.135
−0.130
−0.125
−0.120
−0.115
−0.110
−0.105
−0.100
0.2
0.1
0.0
−0.1
−0.2
−0.3
0.4
0.3
0.2
0.1
0.0
−0.1
−0.2
−0.3
−0.4

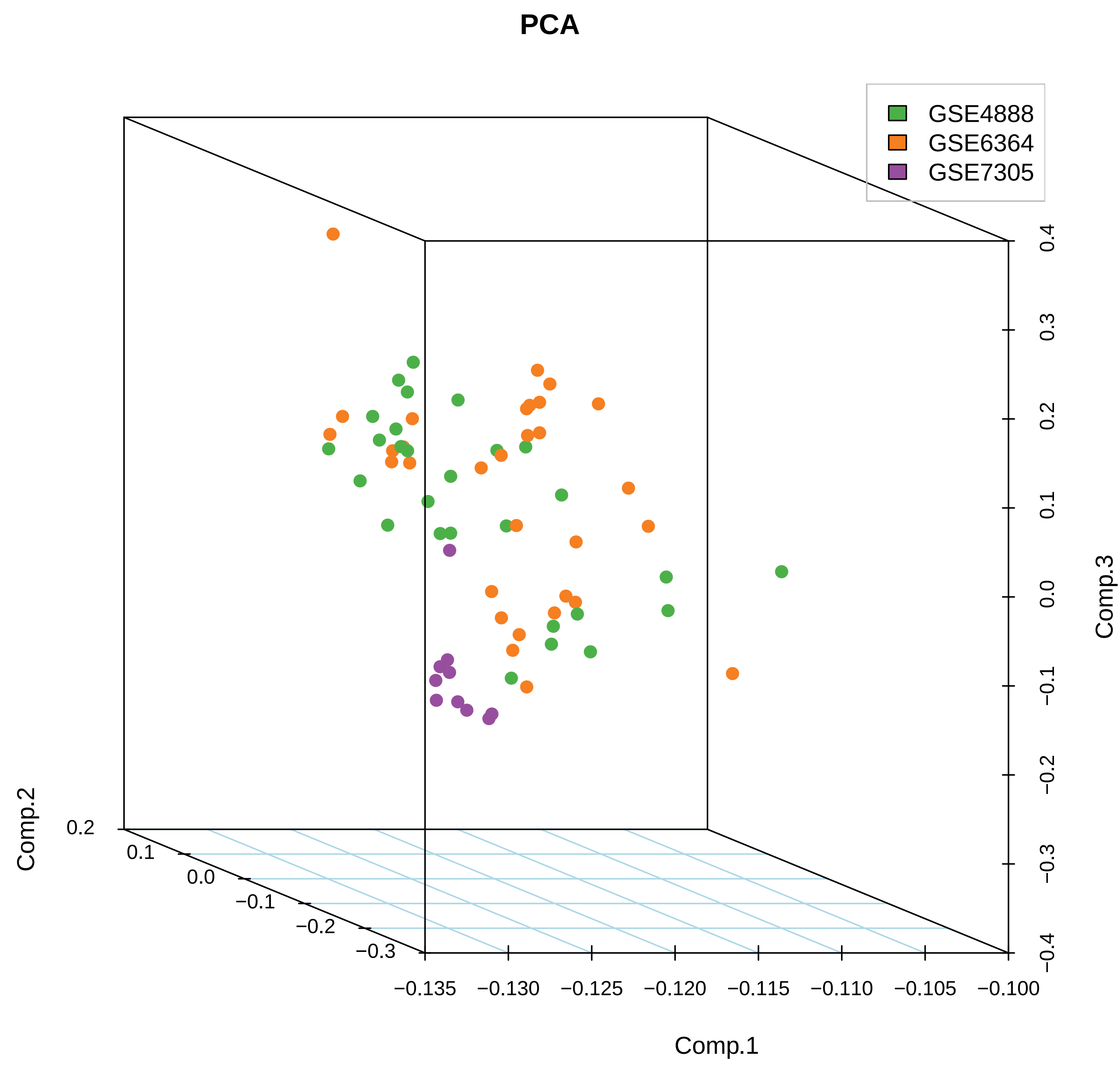
PCA
GSE4888
GSE6364
GSE7305
Comp.1
Comp.2
Comp.3
−0.135
−0.130
−0.125
−0.120
−0.115
−0.110
−0.105
−0.100
0.2
0.1
0.0
−0.1
−0.2
−0.3
0.4
0.3
0.2
0.1
0.0
−0.1
−0.2
−0.3
−0.4

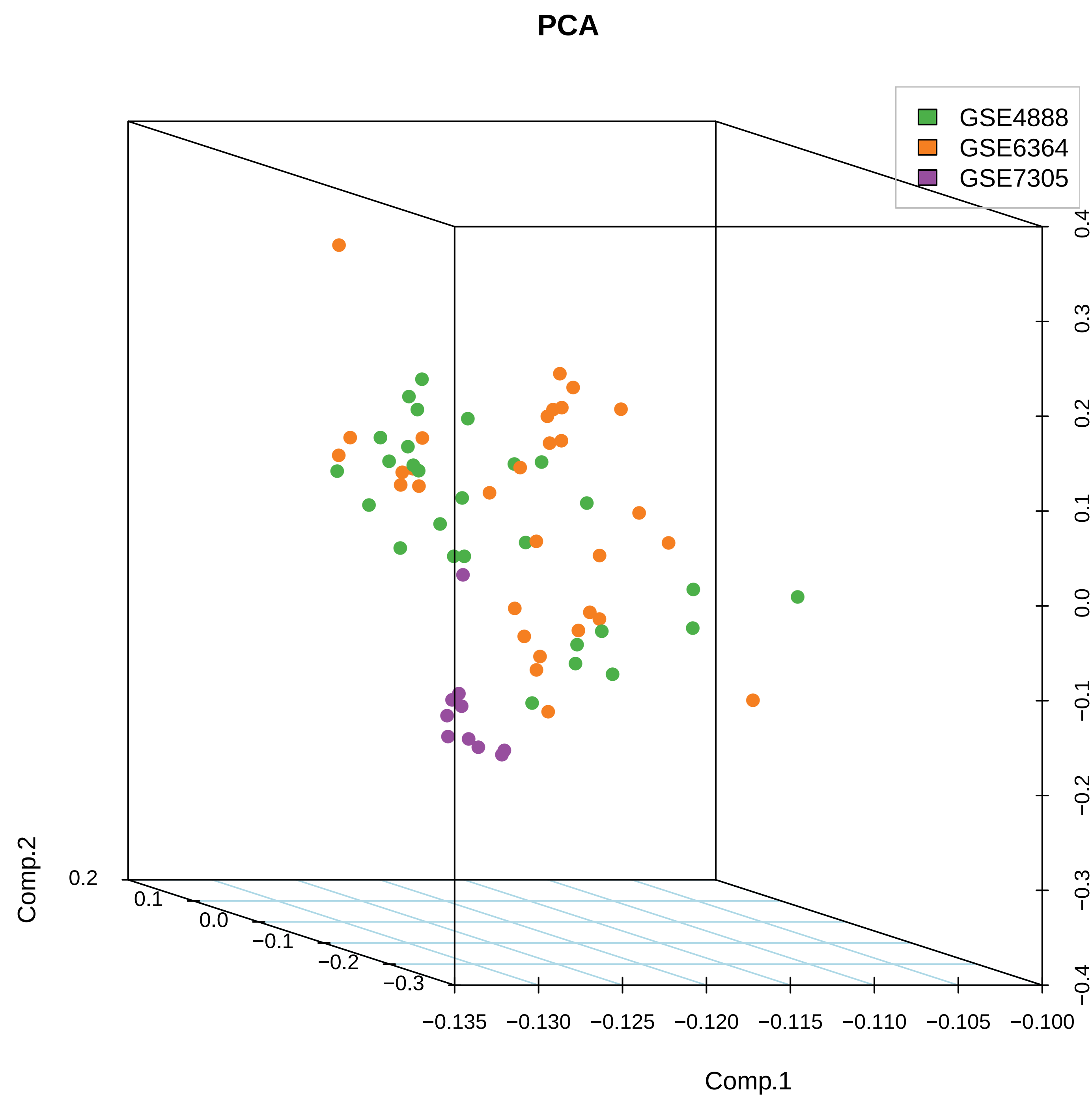
PCA
GSE4888
GSE6364
GSE7305
Comp.1
Comp.2
Comp.3
−0.135
−0.130
−0.125
−0.120
−0.115
−0.110
−0.105
−0.100
0.2
0.1
0.0
−0.1
−0.2
−0.3
0.4
0.3
0.2
0.1
0.0
−0.1
−0.2
−0.3
−0.4

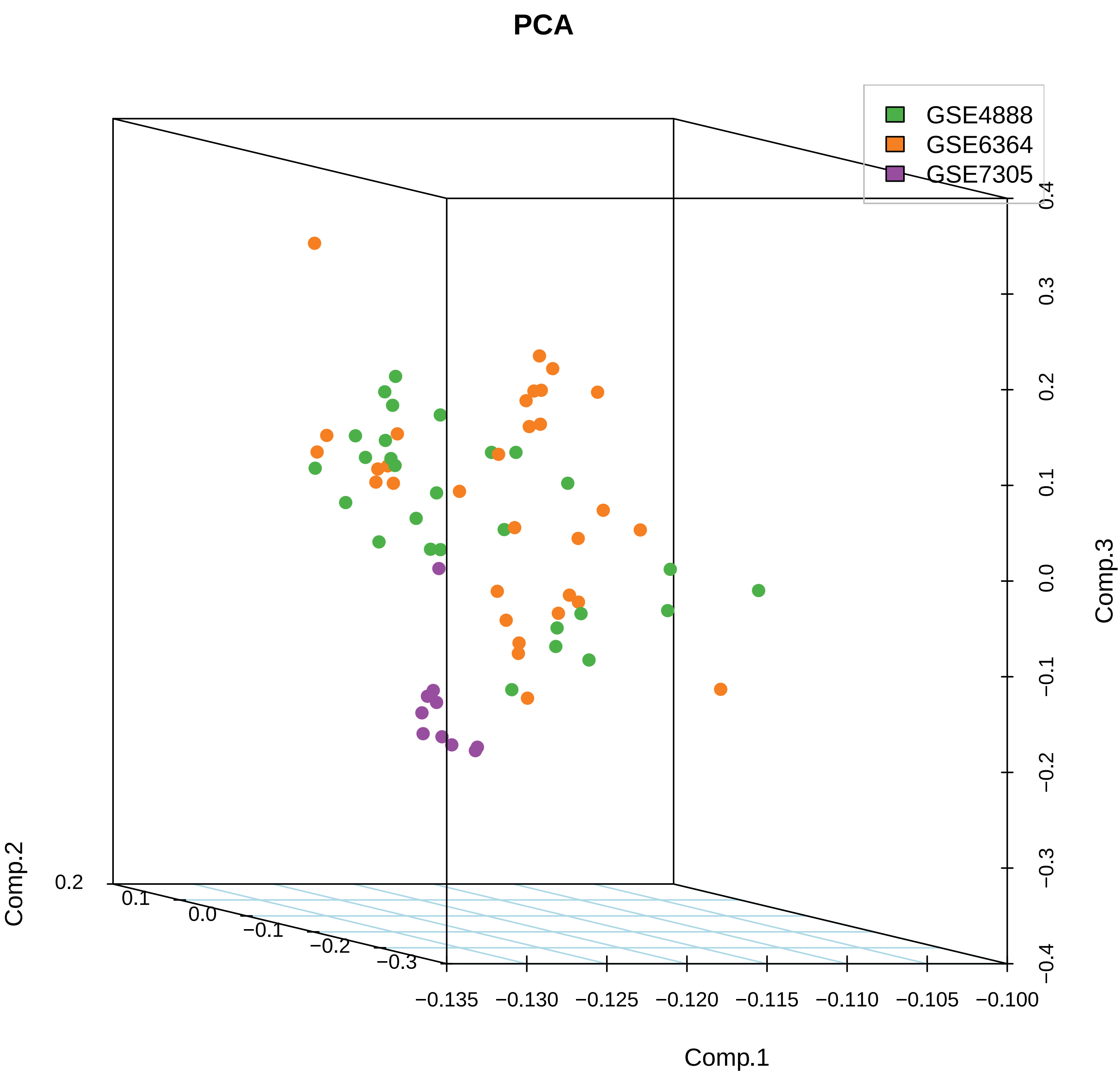
PCA
GSE4888
GSE6364
GSE7305
Comp.1
Comp.2
Comp.3
−0.135
−0.130
−0.125
−0.120
−0.115
−0.110
−0.105
−0.100
0.2
0.1
0.0
−0.1
−0.2
−0.3
0.4
0.3
0.2
0.1
0.0
−0.1
−0.2
−0.3
−0.4

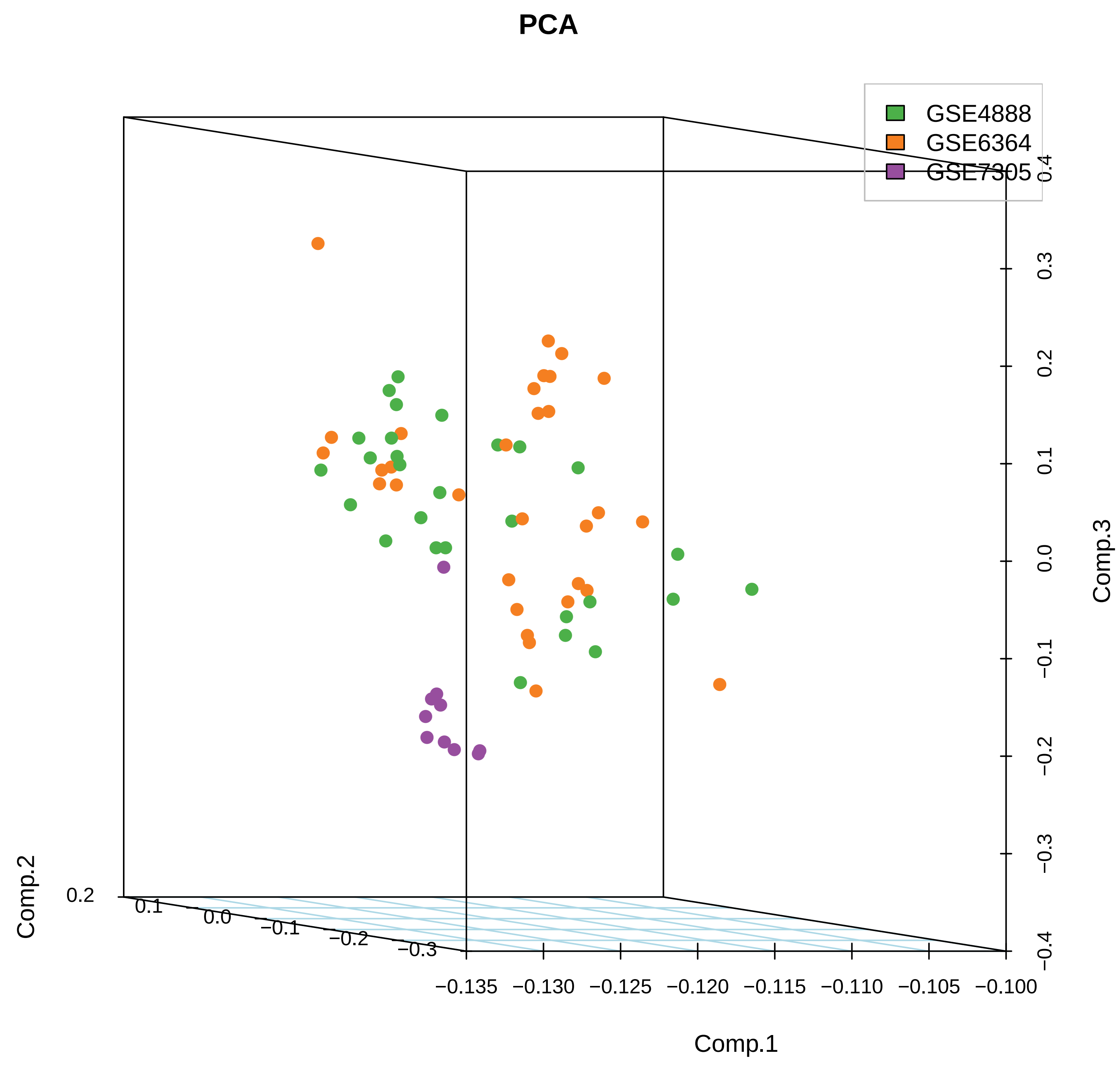

PCA
GSE4888
GSE6364
GSE7305
Comp.1
Comp.2
Comp.3
−0.135
−0.130
−0.125
−0.120
−0.115
−0.110
−0.105
−0.100
0.2
0.1
0.0
−0.1
−0.2
−0.3
0.4
0.3
0.2
0.1
0.0
−0.1
−0.2
−0.3
−0.4

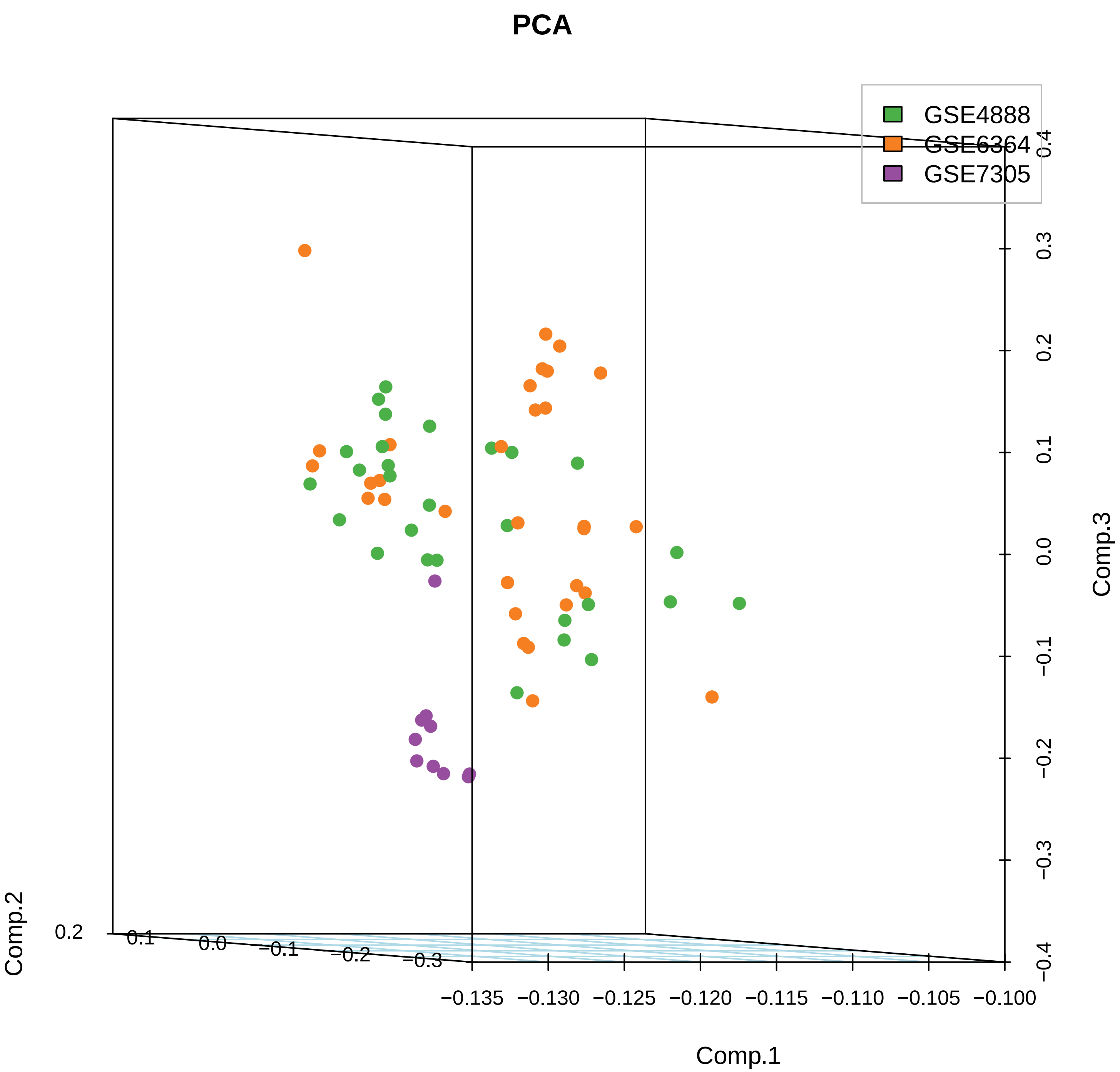
PCA
GSE4888
GSE6364
GSE7305
Comp.1
Comp.2
Comp.3
−0.135 −0.130 −0.125 −0.120 −0.115 −0.110 −0.105 −0.100
0.2 0.1 0.0 −0.1 −0.2 −0.3
−0.4 −0.3 −0.2 −0.1 0.0 0.1 0.2 0.3 0.4

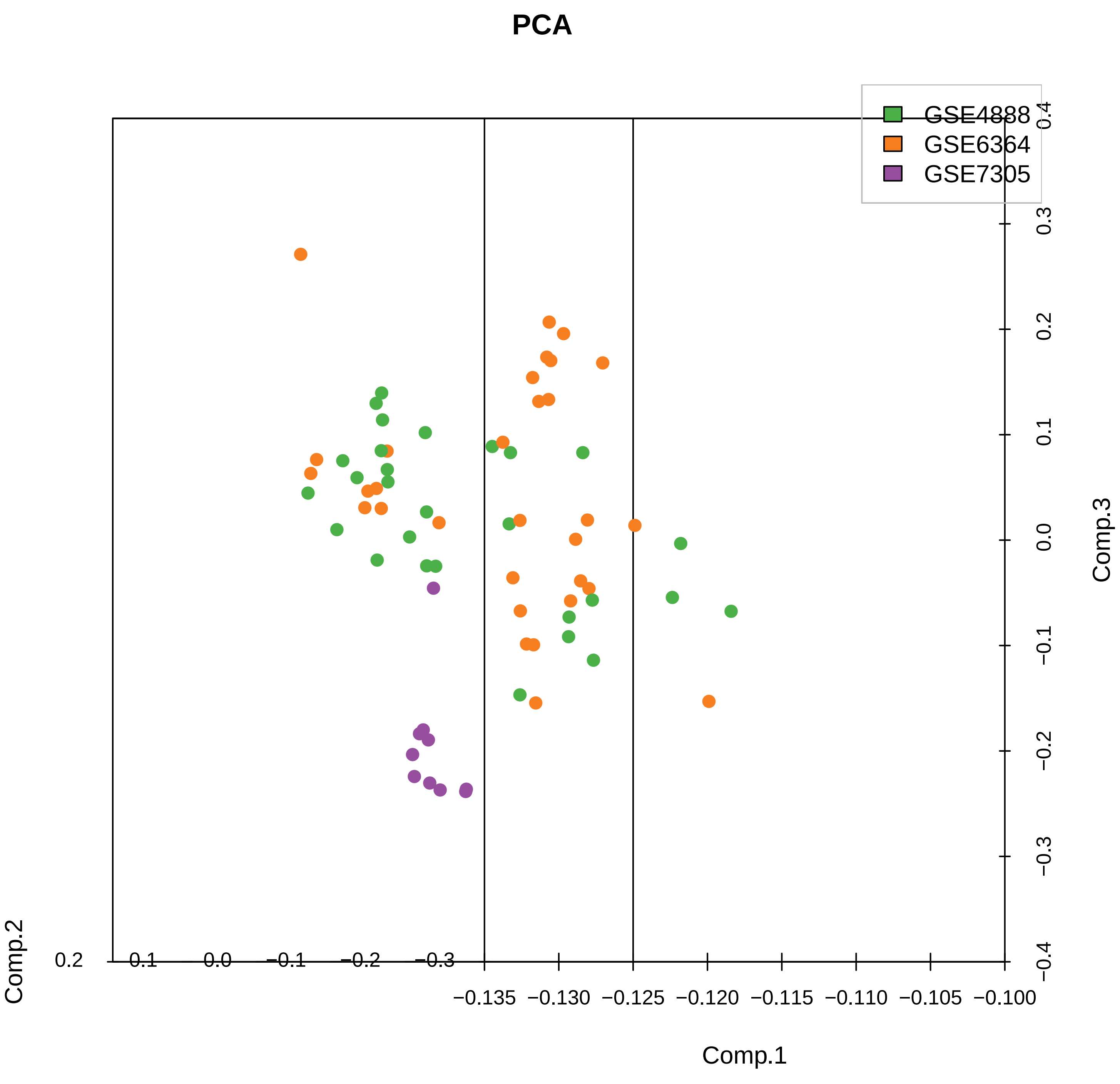

PCA
GSE4888
GSE6364
GSE7305
Comp.1
Comp.2
Comp.3
−0.135
−0.130
−0.125
−0.120
−0.115
−0.110
−0.105
−0.100
0.2
0.1
0.0
−0.1
−0.2
−0.3
−0.4
−0.3
−0.2
−0.1
0.0
0.1
0.2
0.3
0.4

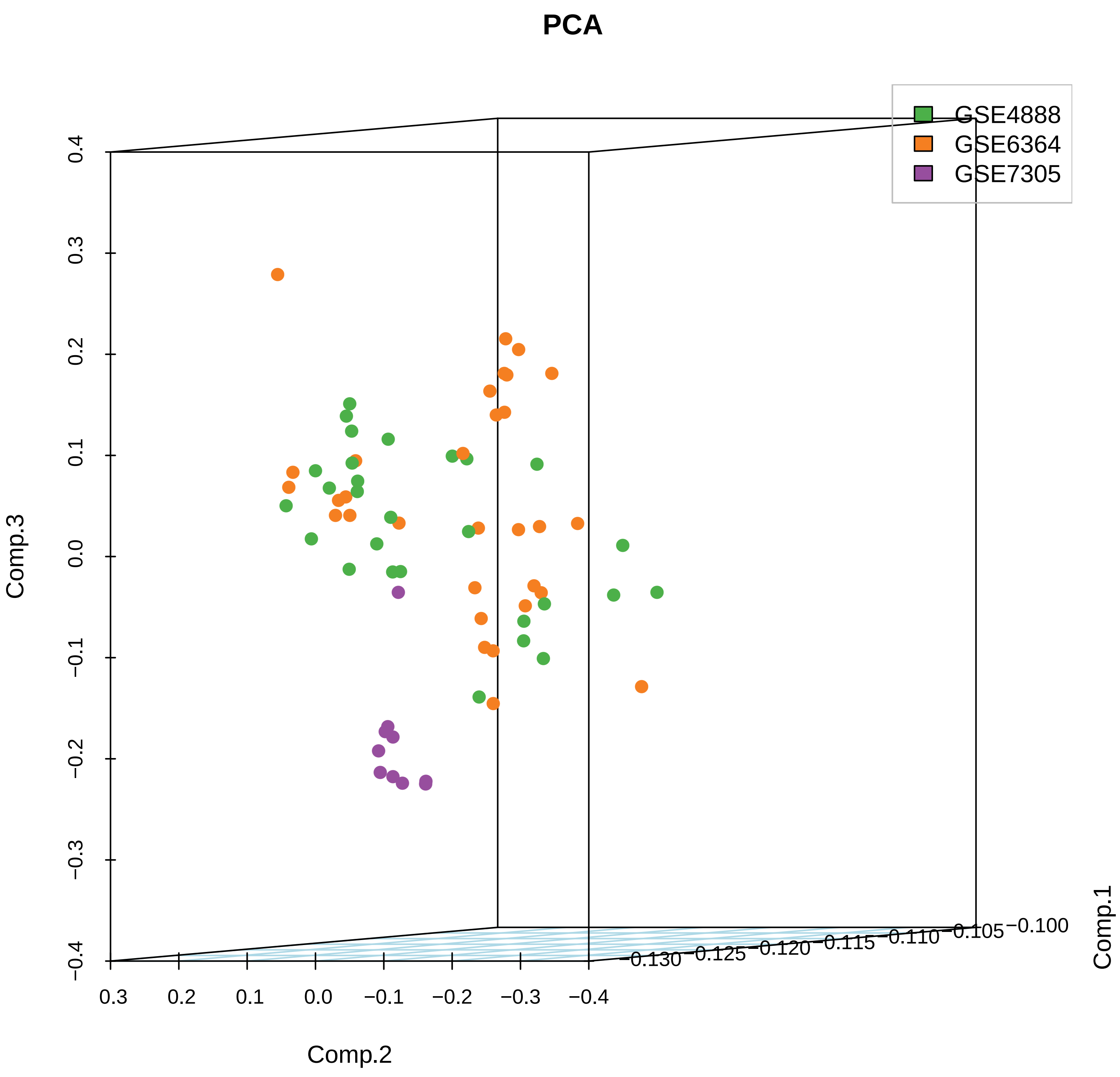
PCA
GSE4888
GSE6364
GSE7305
Comp.3
0.4
0.3
0.2
0.1
0.0
−0.1
−0.2
−0.3
−0.4
0.3
0.2
0.1
0.0
−0.1
−0.2
−0.3
−0.4
Comp.2
−0.130
−0.125
−0.120
−0.115
−0.110
−0.105
−0.100
Comp.1

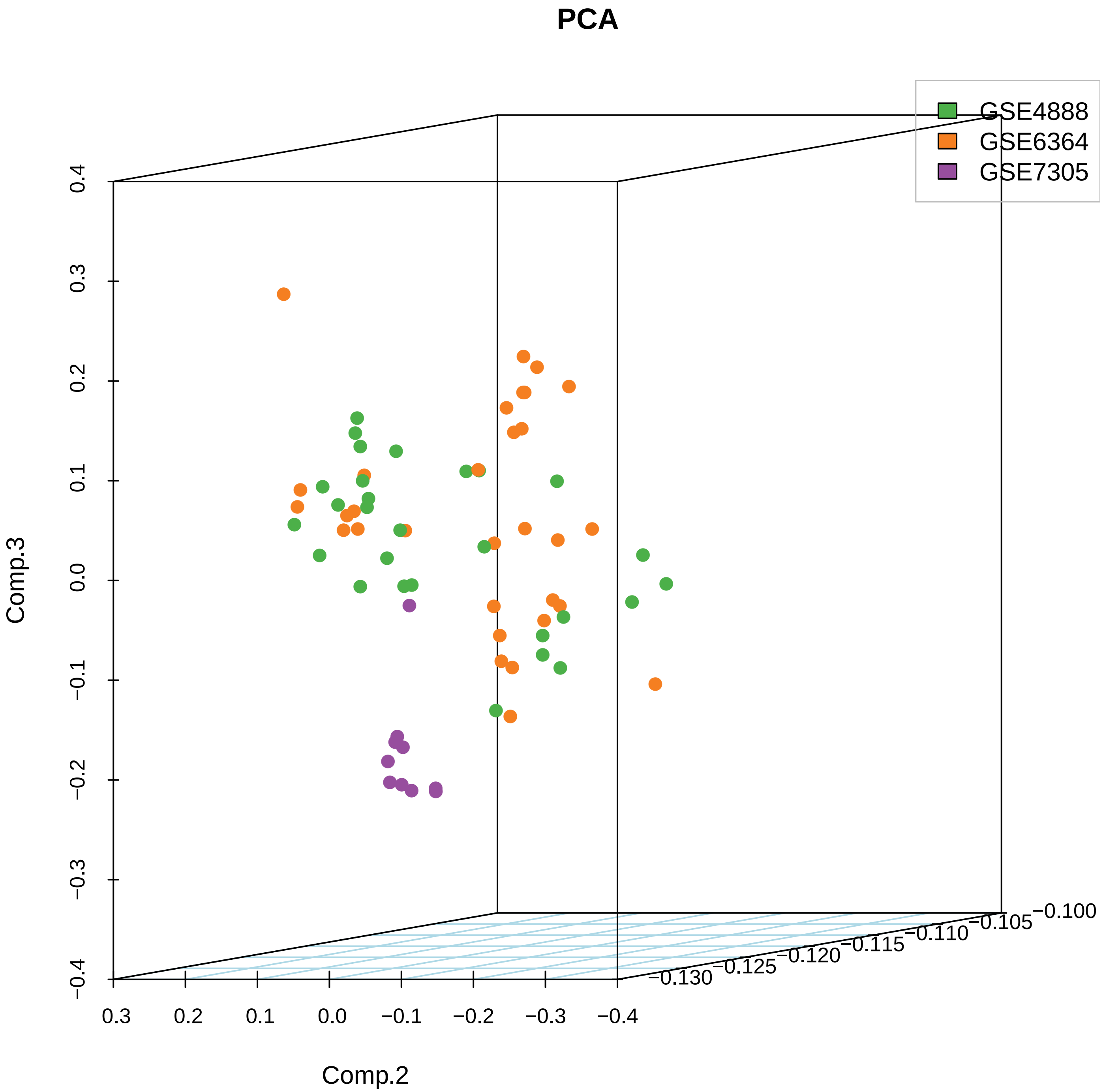
PCA
GSE4888
GSE6364
GSE7305
Comp.3
0.4
0.3
0.2
0.1
0.0
−0.1
−0.2
−0.3
−0.4
0.3
0.2
0.1
0.0
−0.1
−0.2
−0.3
−0.4
Comp.2
−0.130
−0.125
−0.120
−0.115
−0.110
−0.105
−0.100
Comp.1

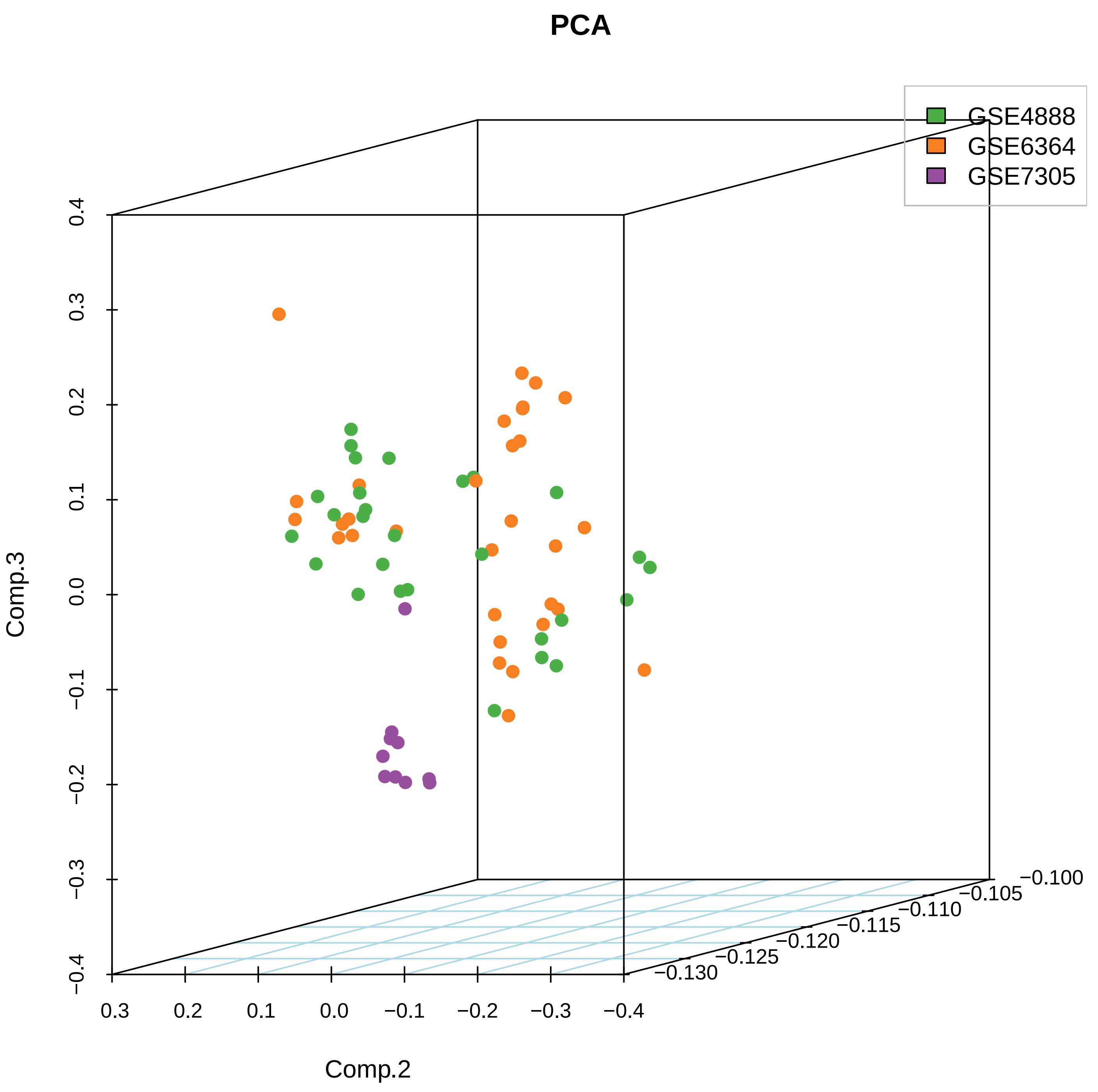
PCA
GSE4888
GSE6364
GSE7305
Comp.3
0.4
0.3
0.2
0.1
0.0
−0.1
−0.2
−0.3
−0.4
0.3
0.2
0.1
0.0
−0.1
−0.2
−0.3
−0.4
Comp.2
−0.100
−0.105
−0.110
−0.115
−0.120
−0.125
−0.130
Comp.1

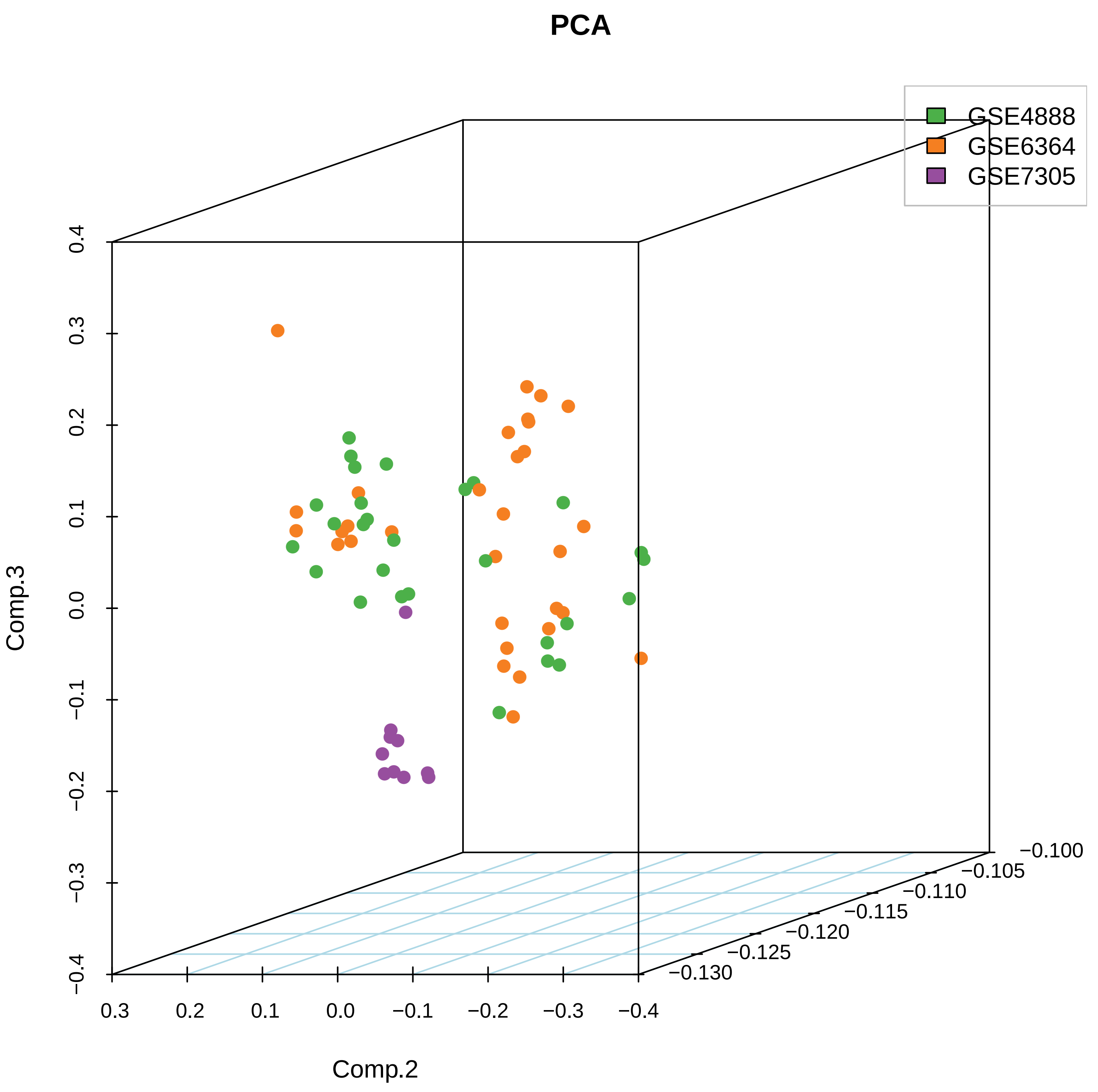
PCA
GSE4888
GSE6364
GSE7305
Comp.3
0.4
0.3
0.2
0.1
0.0
−0.1
−0.2
−0.3
−0.4
Comp.2
0.3
0.2
0.1
0.0
−0.1
−0.2
−0.3
−0.4
Comp.1
−0.100
−0.105
−0.110
−0.115
−0.120
−0.125
−0.130

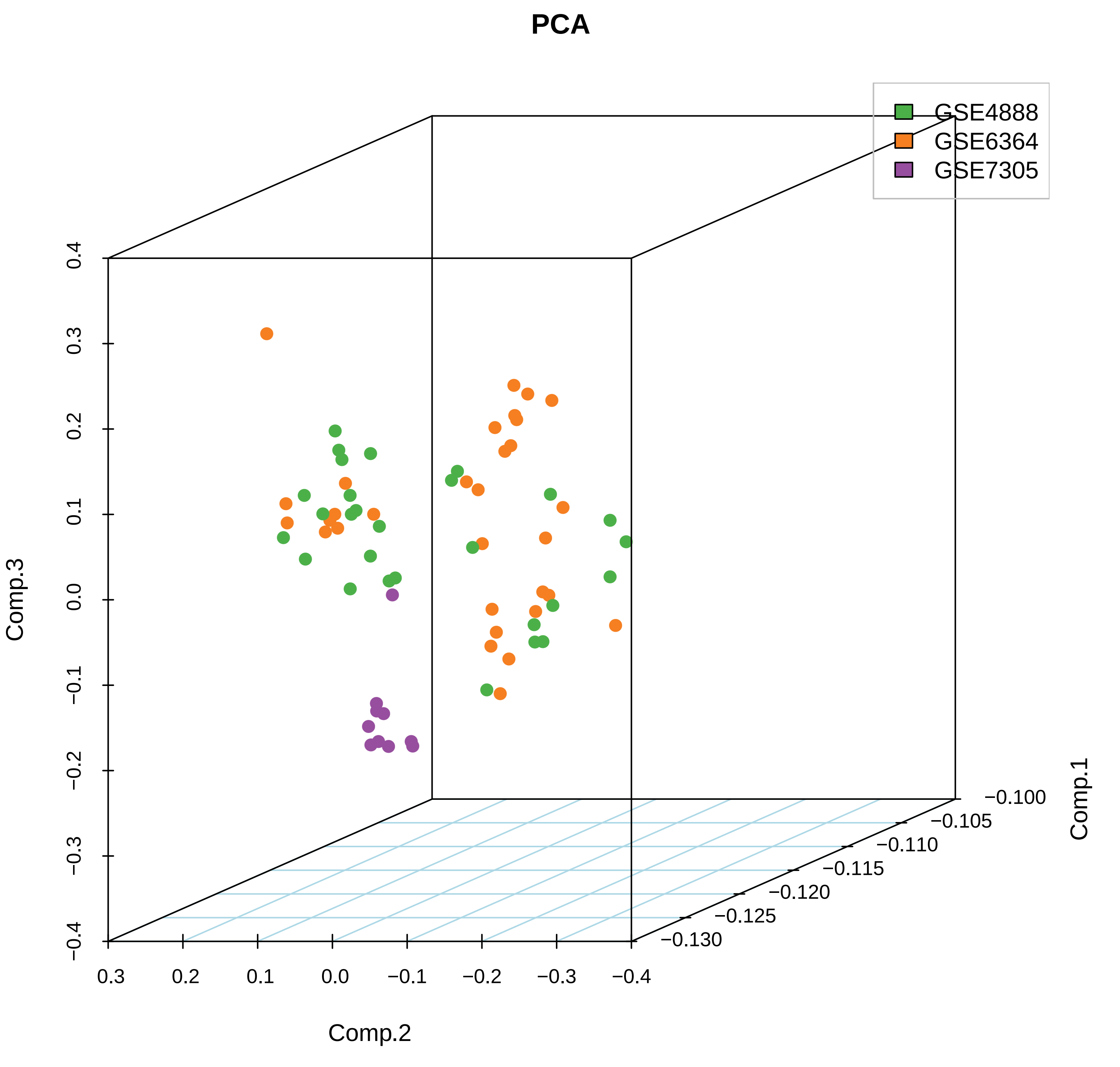
PCA
GSE4888
GSE6364
GSE7305
Comp.1
Comp.2
Comp.3
0.4
0.3
0.2
0.1
0.0
−0.1
−0.2
−0.3
−0.4
0.3
0.2
0.1
0.0
−0.1
−0.2
−0.3
−0.4
−0.100
−0.105
−0.110
−0.115
−0.120
−0.125
−0.130

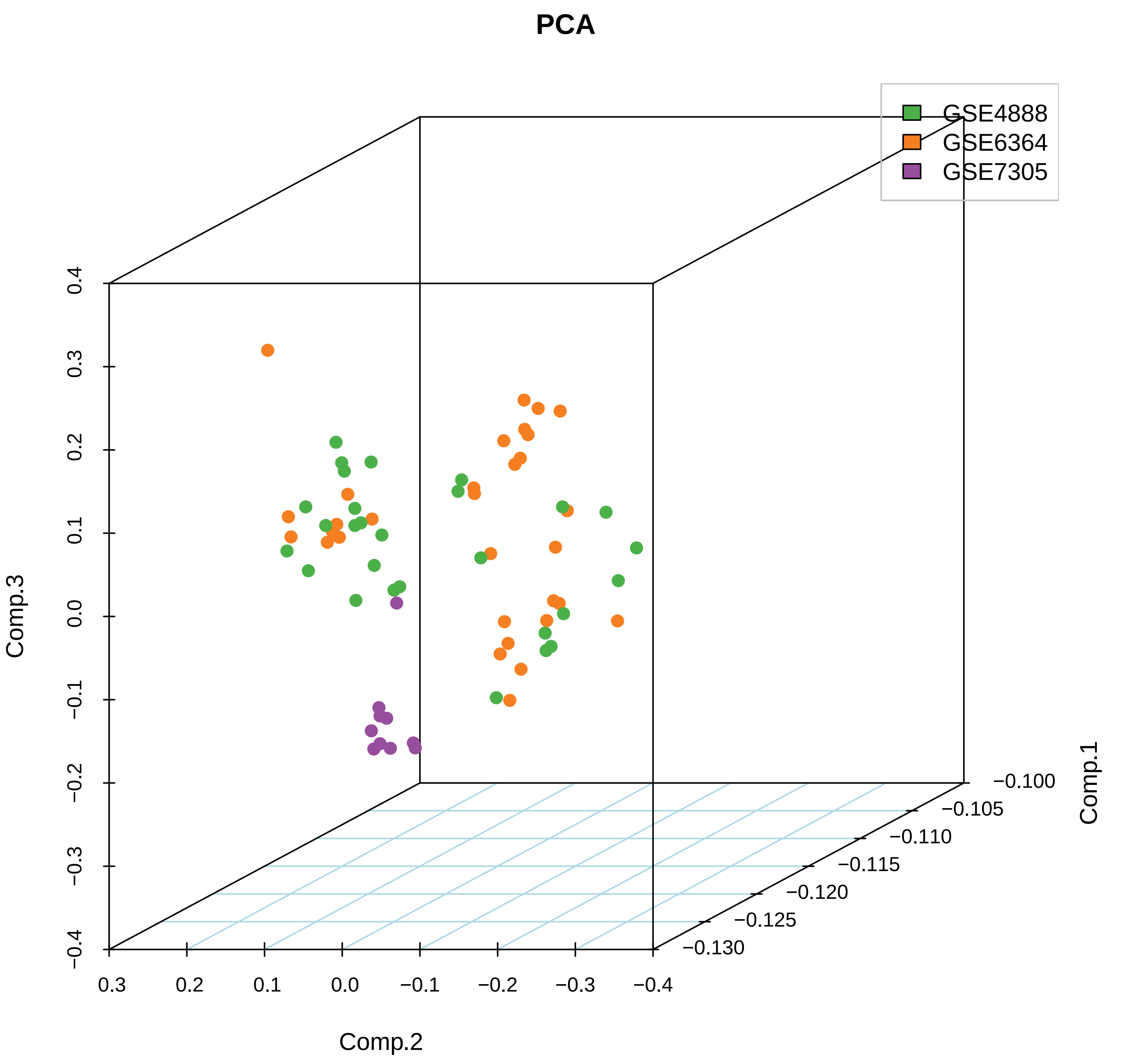
PCA
GSE4888
GSE6364
GSE7305
Comp.1
Comp.2
Comp.3
0.4
0.3
0.2
0.1
0.0
−0.1
−0.2
−0.3
−0.4
0.3
0.2
0.1
0.0
−0.1
−0.2
−0.3
−0.4
−0.100
−0.105
−0.110
−0.115
−0.120
−0.125
−0.130

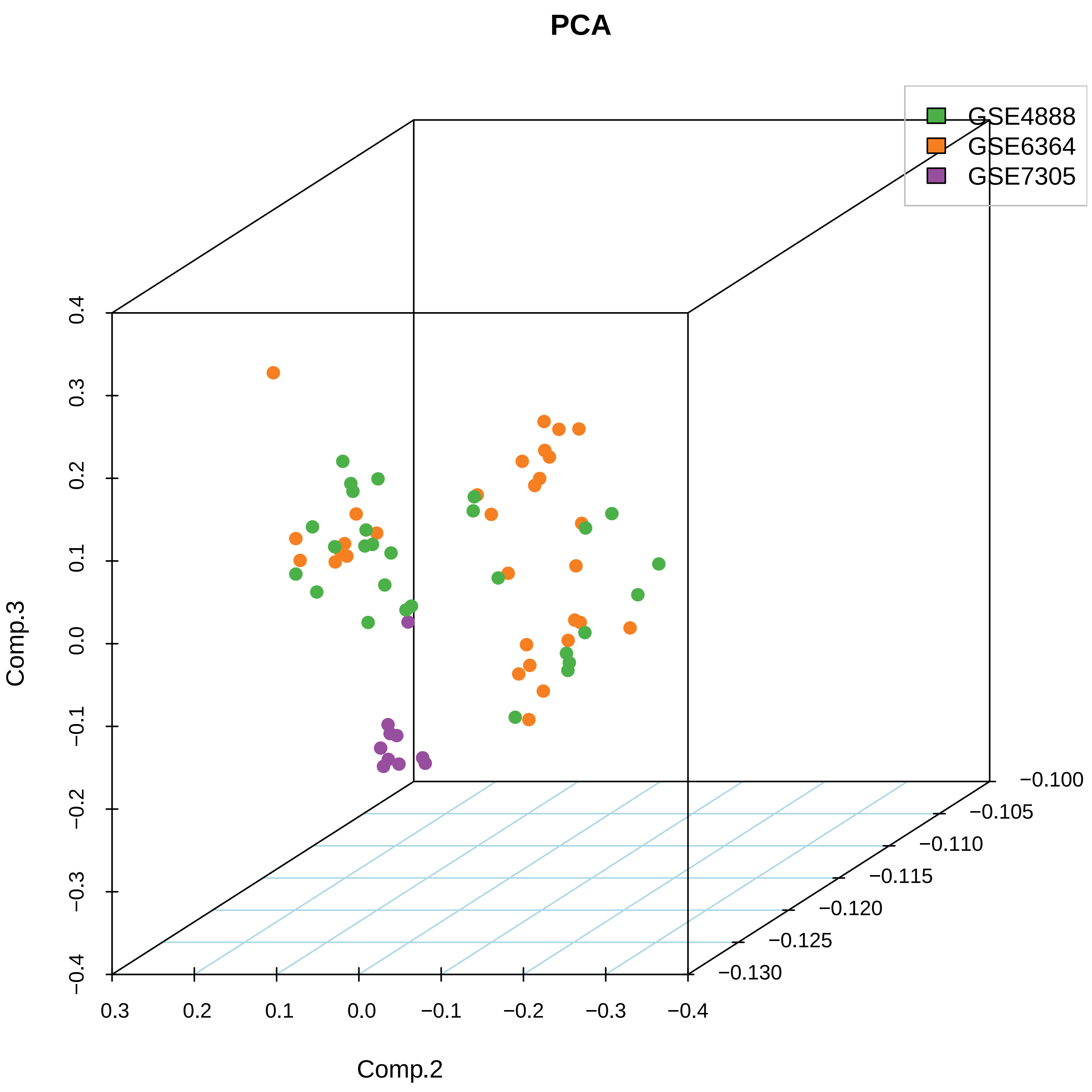

PCA
GSE4888
GSE6364
GSE7305
Comp.3
0.4
0.3
0.2
0.1
0.0
−0.1
−0.2
−0.3
−0.4
0.3
0.2
0.1
0.0
−0.1
−0.2
−0.3
−0.4
Comp.2
Comp.1
−0.100
−0.105
−0.110
−0.115
−0.120
−0.125
−0.130

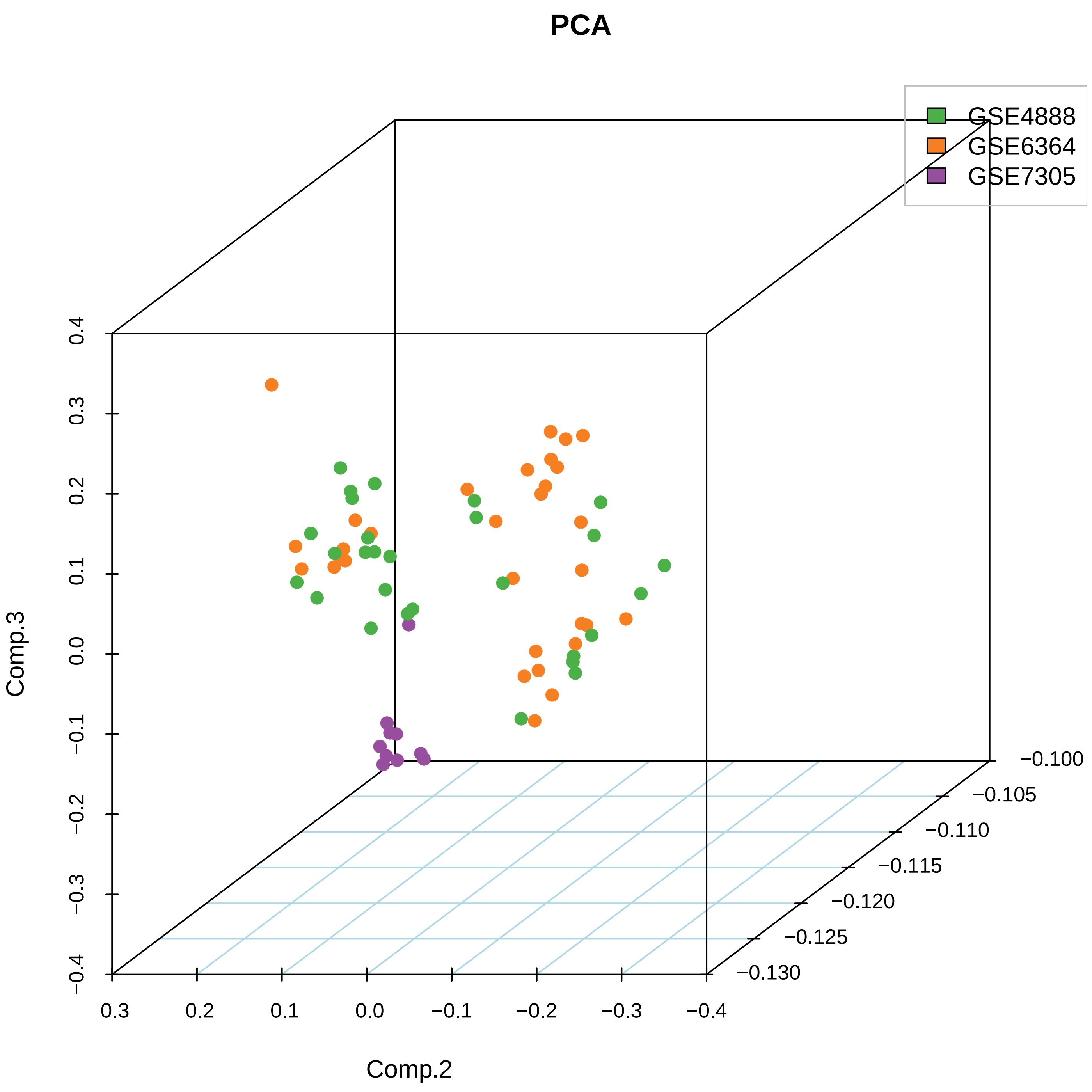

PCA
GSE4888
GSE6364
GSE7305
Comp.3
0.4
0.3
0.2
0.1
0.0
−0.1
−0.2
−0.3
−0.4
Comp.2
0.3
0.2
0.1
0.0
−0.1
−0.2
−0.3
−0.4
Comp.1
−0.100
−0.105
−0.110
−0.115
−0.120
−0.125
−0.130

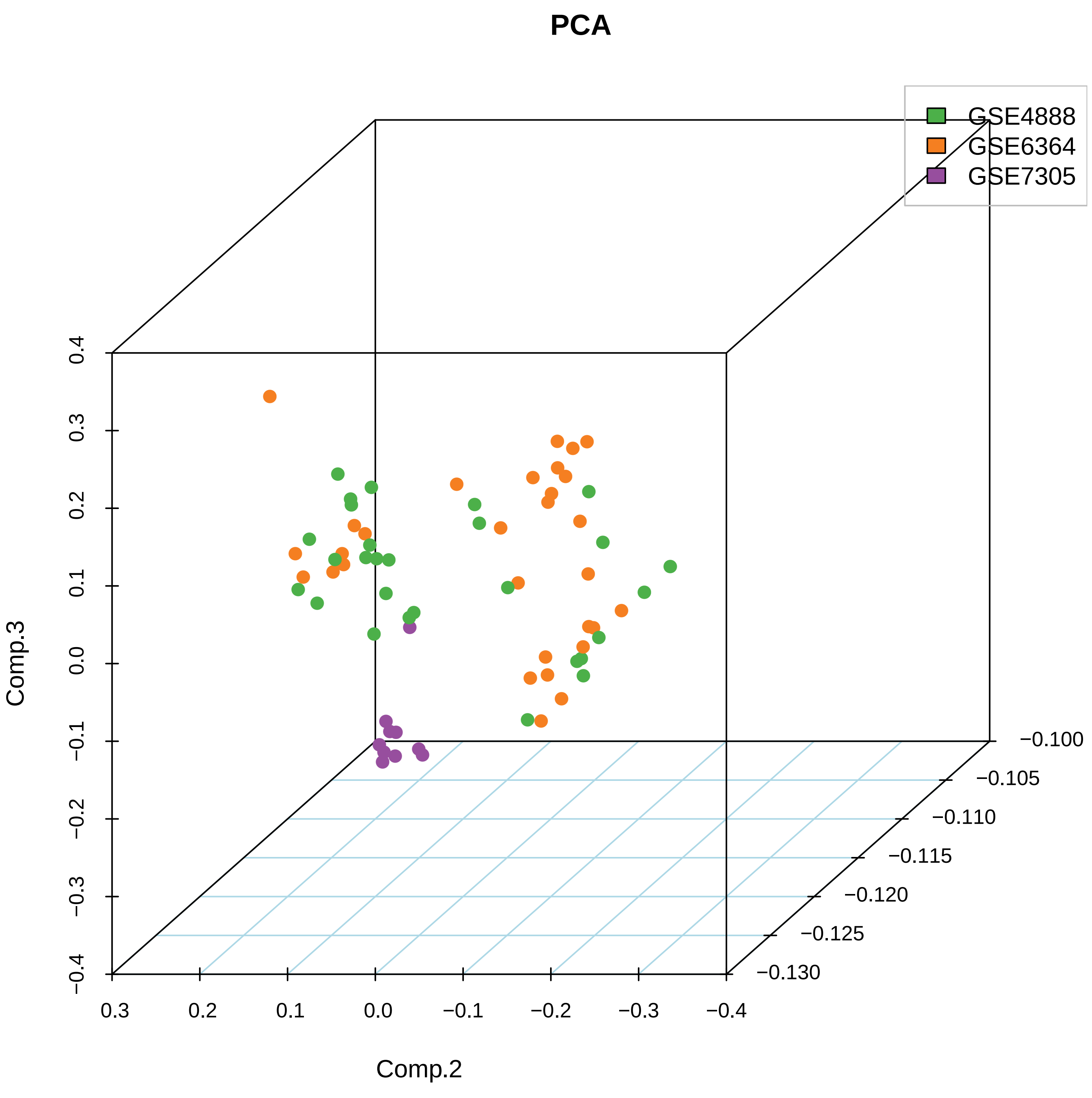
PCA
GSE4888
GSE6364
GSE7305
Comp.3
0.4
0.3
0.2
0.1
0.0
−0.1
−0.2
−0.3
−0.4
Comp.2
0.3
0.2
0.1
0.0
−0.1
−0.2
−0.3
−0.4
Comp.1
−0.100
−0.105
−0.110
−0.115
−0.120
−0.125
−0.130

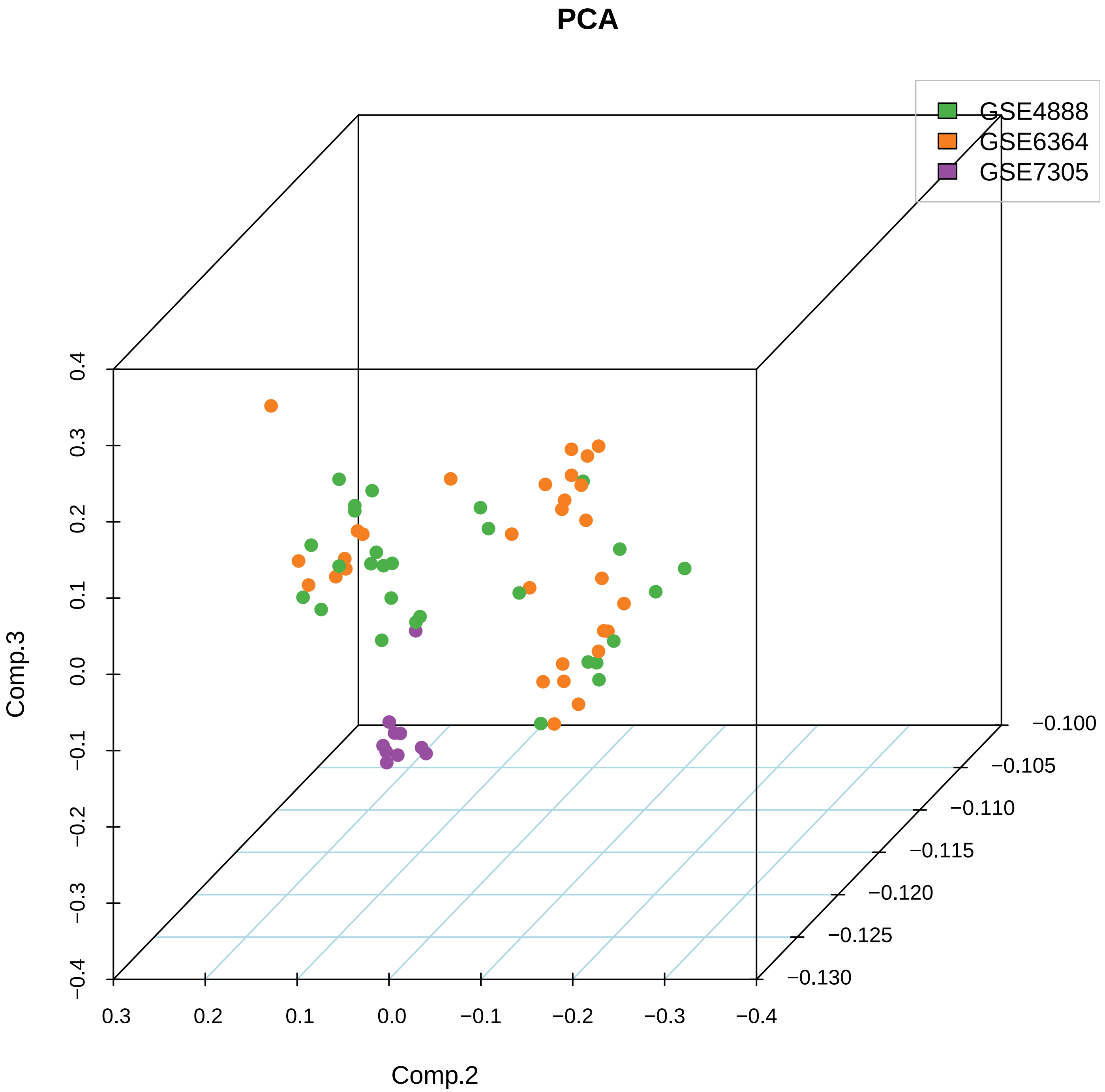
PCA
GSE4888
GSE6364
GSE7305
Comp.3
0.4
0.3
0.2
0.1
0.0
−0.1
−0.2
−0.3
−0.4
Comp.2
0.3
0.2
0.1
0.0
−0.1
−0.2
−0.3
−0.4
Comp.1
−0.100
−0.105
−0.110
−0.115
−0.120
−0.125
−0.130

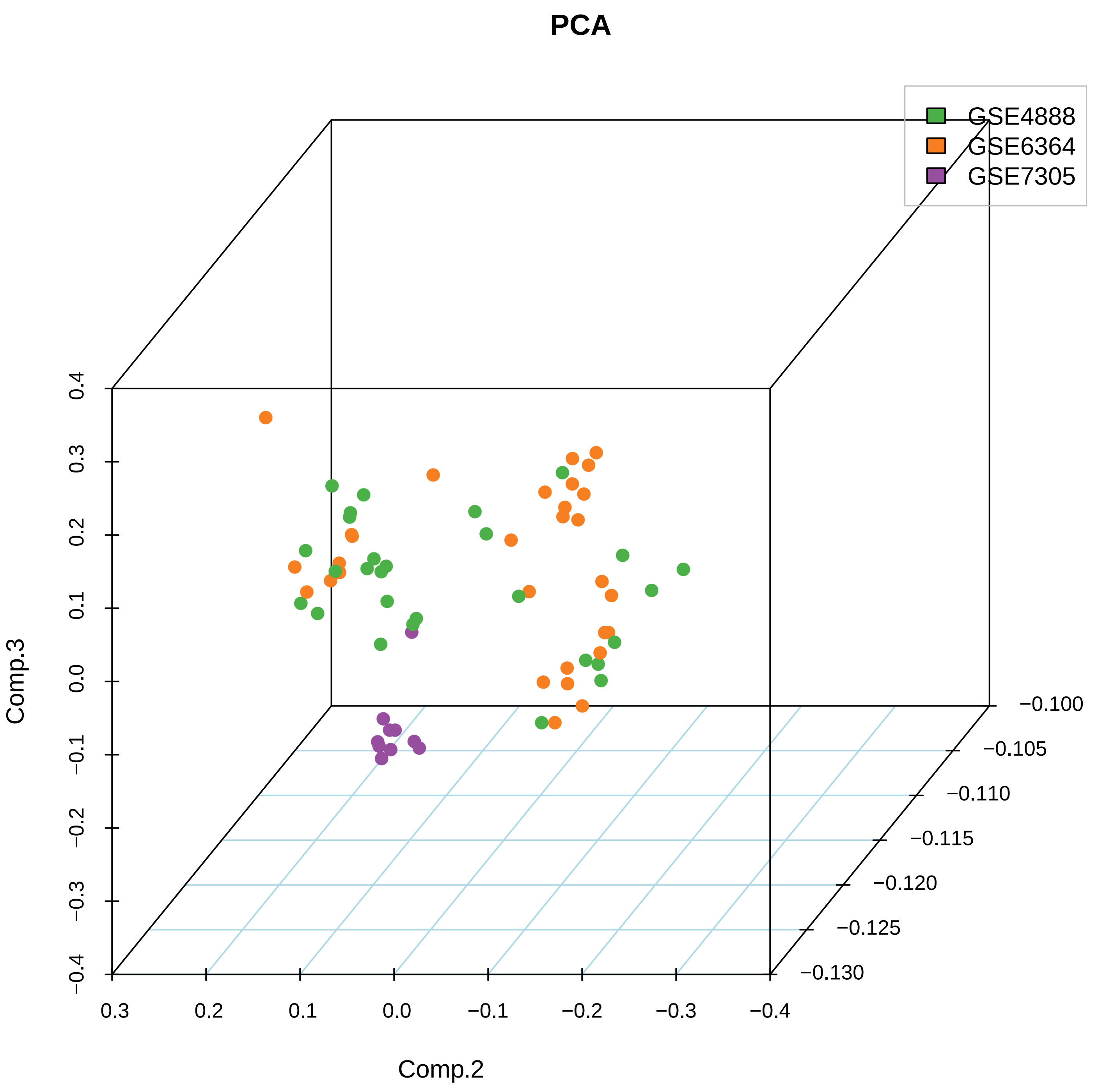
PCA
GSE4888
GSE6364
GSE7305
Comp.1
Comp.2
Comp.3
0.4
0.3
0.2
0.1
0.0
−0.1
−0.2
−0.3
−0.4
0.3
0.2
0.1
0.0
−0.1
−0.2
−0.3
−0.4
−0.100
−0.105
−0.110
−0.115
−0.120
−0.125
−0.130

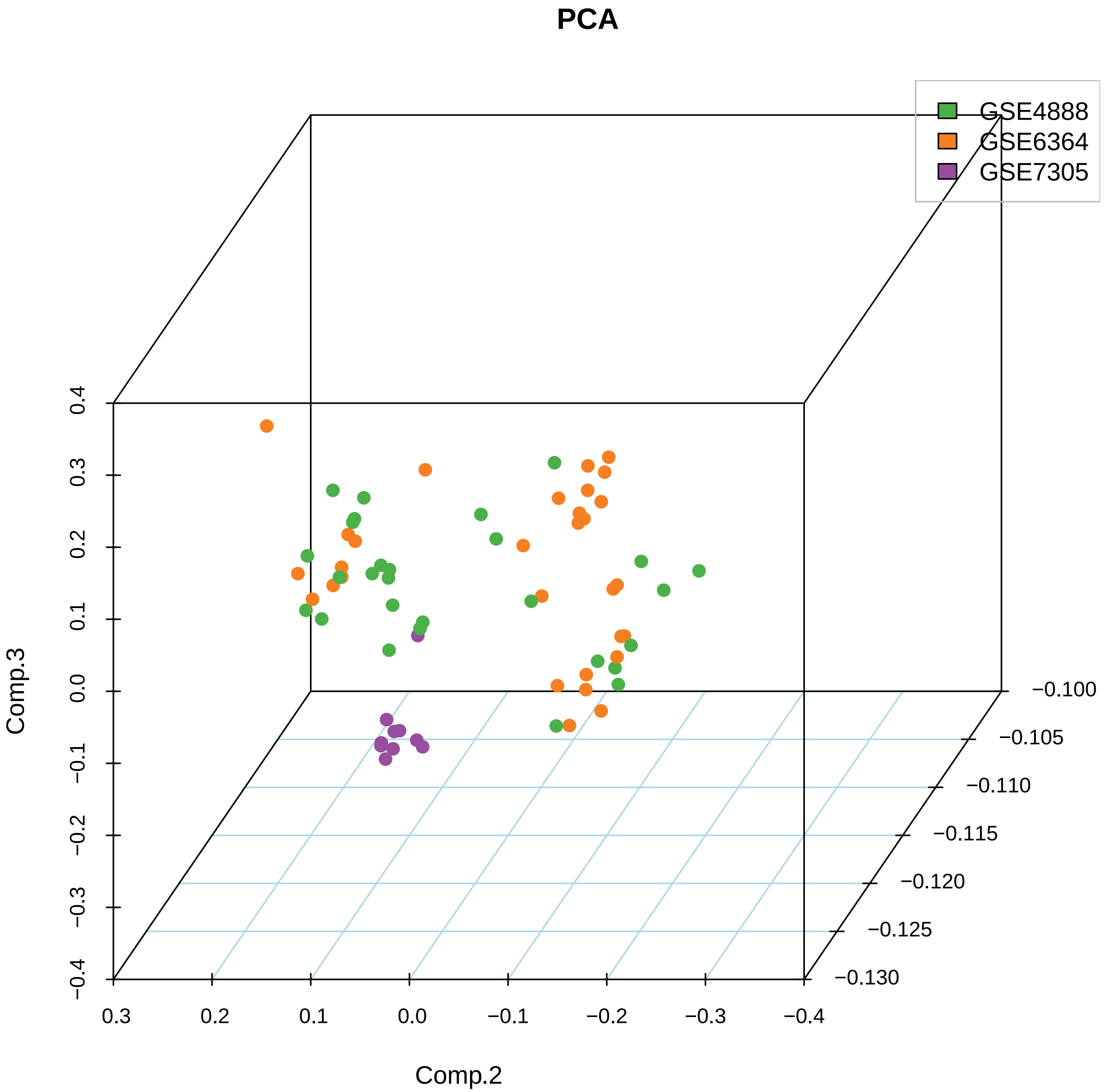

PCA
GSE4888
GSE6364
GSE7305
Comp.3
Comp.2
Comp.1
0.4
0.3
0.2
0.1
0.0
−0.1
−0.2
−0.3
−0.4
0.3
0.2
0.1
0.0
−0.1
−0.2
−0.3
−0.4
−0.100
−0.105
−0.110
−0.115
−0.120
−0.125
−0.130

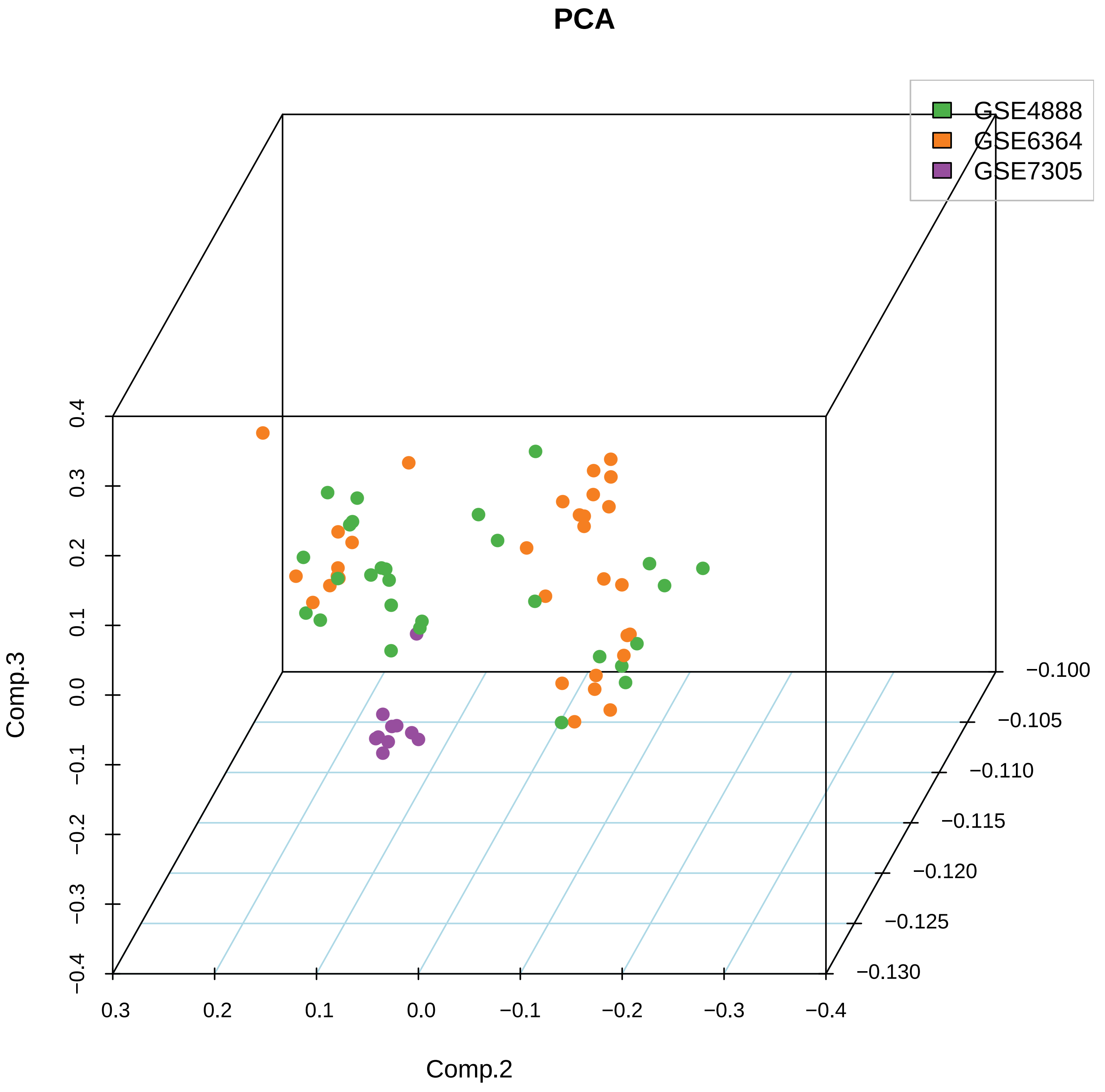

PCA
GSE4888
GSE6364
GSE7305
Comp.3
Comp.2
Comp.1
0.4
0.3
0.2
0.1
0.0
−0.1
−0.2
−0.3
−0.4
0.3
0.2
0.1
0.0
−0.1
−0.2
−0.3
−0.4
−0.100
−0.105
−0.110
−0.115
−0.120
−0.125
−0.130

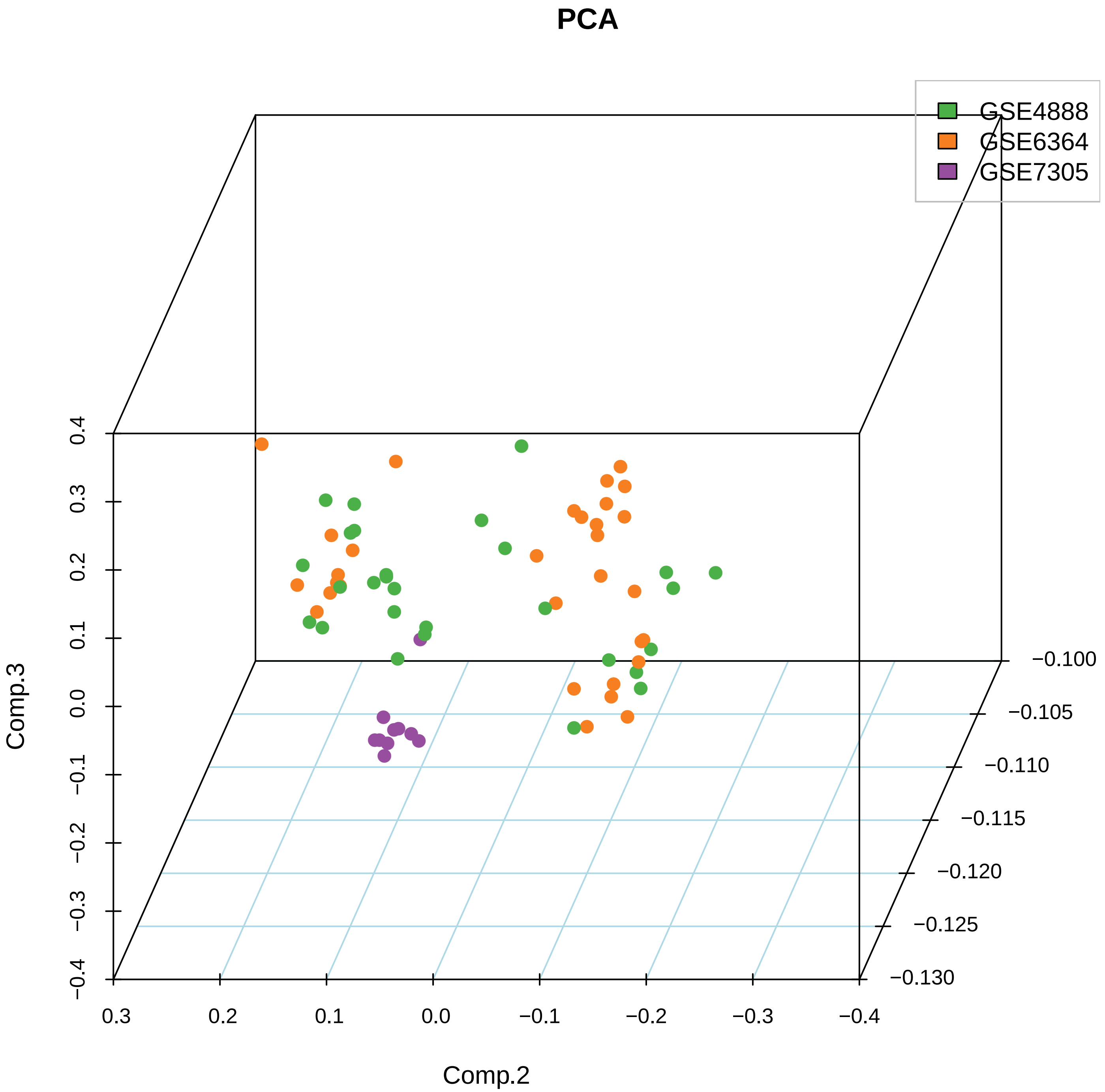
PCA
GSE4888
GSE6364
GSE7305
Comp.3
Comp.2
Comp.1
0.4
0.3
0.2
0.1
0.0
−0.1
−0.2
−0.3
−0.4
0.3
0.2
0.1
0.0
−0.1
−0.2
−0.3
−0.4
−0.100
−0.105
−0.110
−0.115
−0.120
−0.125
−0.130

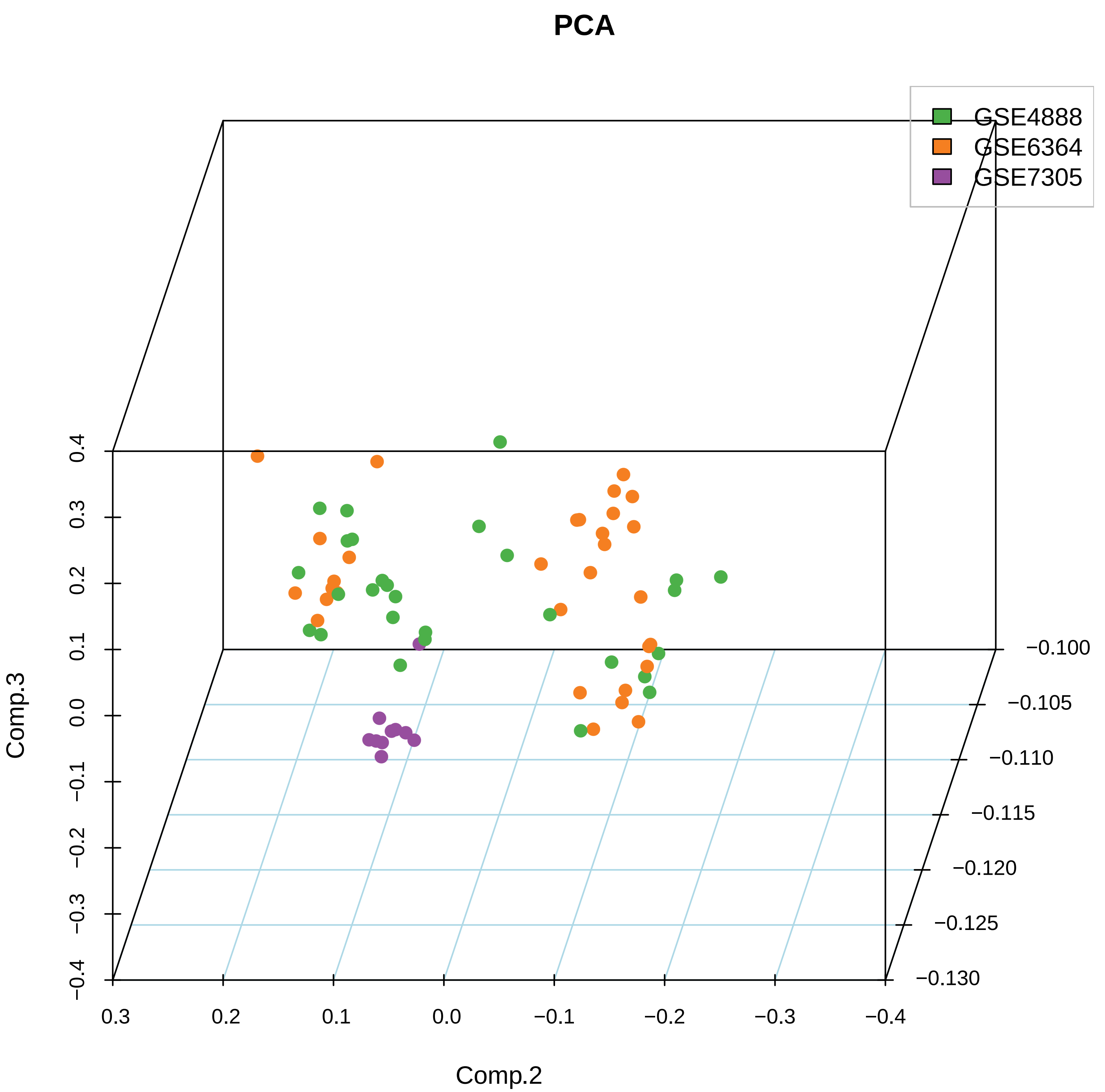

PCA
GSE4888
GSE6364
GSE7305
Comp.3
0.4
0.3
0.2
0.1
0.0
−0.1
−0.2
−0.3
−0.4
0.3
0.2
0.1
0.0
−0.1
−0.2
−0.3
−0.4
Comp.2
Comp.1
−0.100
−0.105
−0.110
−0.115
−0.120
−0.125
−0.130

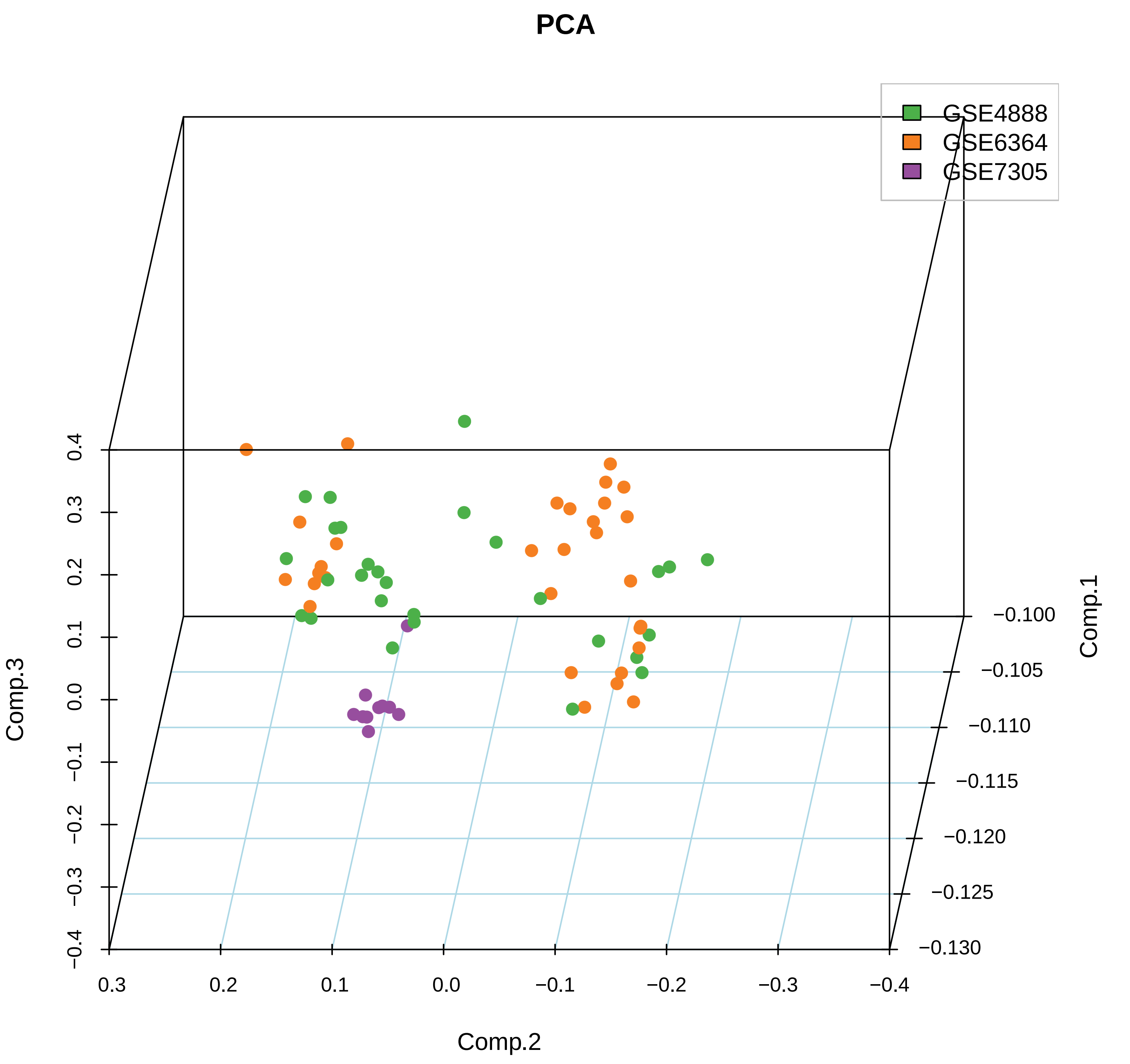
PCA
GSE4888
GSE6364
GSE7305
Comp.1
Comp.2
Comp.3
0.4
0.3
0.2
0.1
0.0
−0.1
−0.2
−0.3
−0.4
0.3
0.2
0.1
0.0
−0.1
−0.2
−0.3
−0.4
−0.100
−0.105
−0.110
−0.115
−0.120
−0.125
−0.130

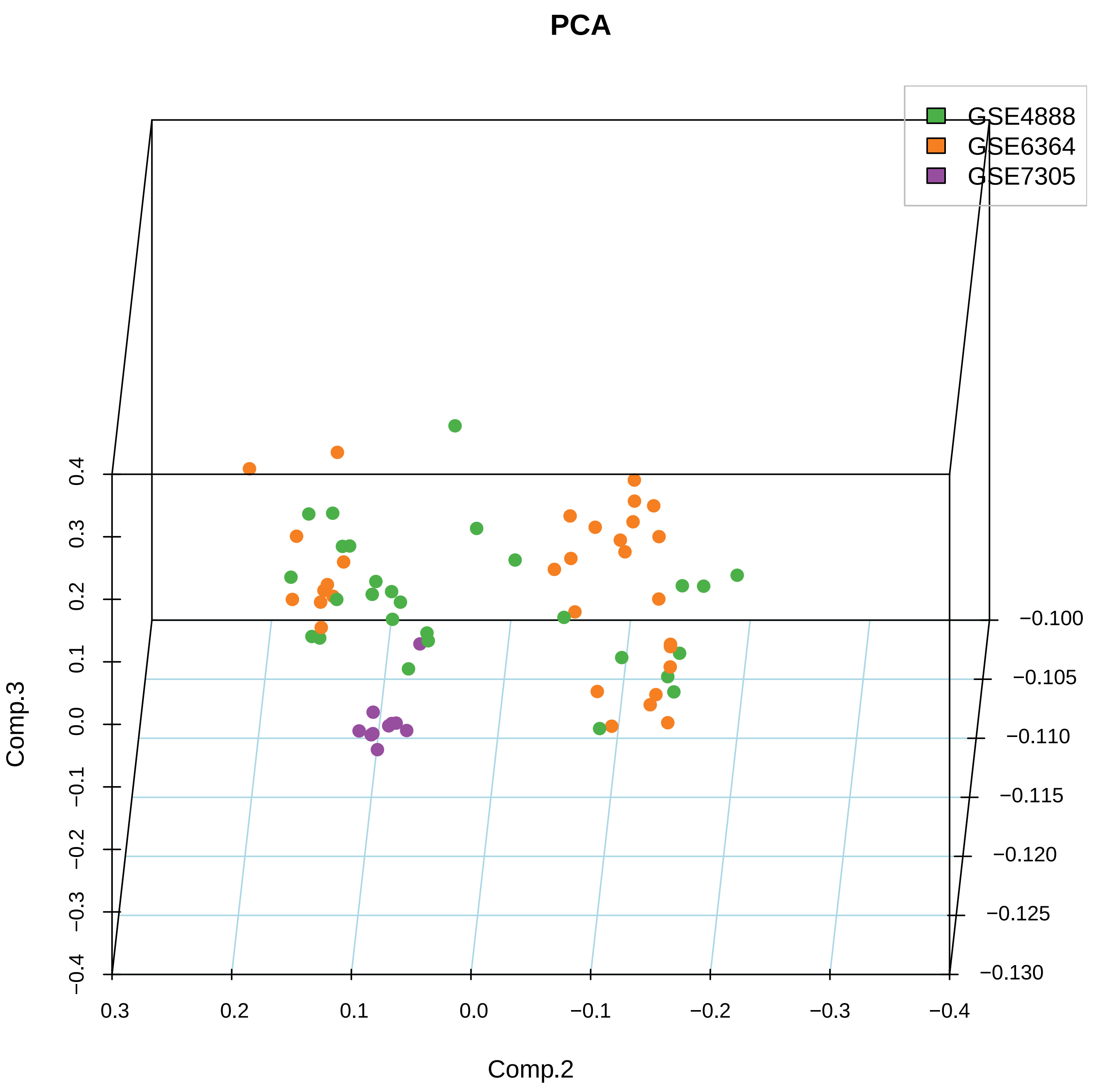
PCA
GSE4888
GSE6364
GSE7305
Comp.3
0.4
0.3
0.2
0.1
0.0
−0.1
−0.2
−0.3
−0.4
Comp.2
0.3
0.2
0.1
0.0
−0.1
−0.2
−0.3
−0.4
Comp.1
−0.100
−0.105
−0.110
−0.115
−0.120
−0.125
−0.130

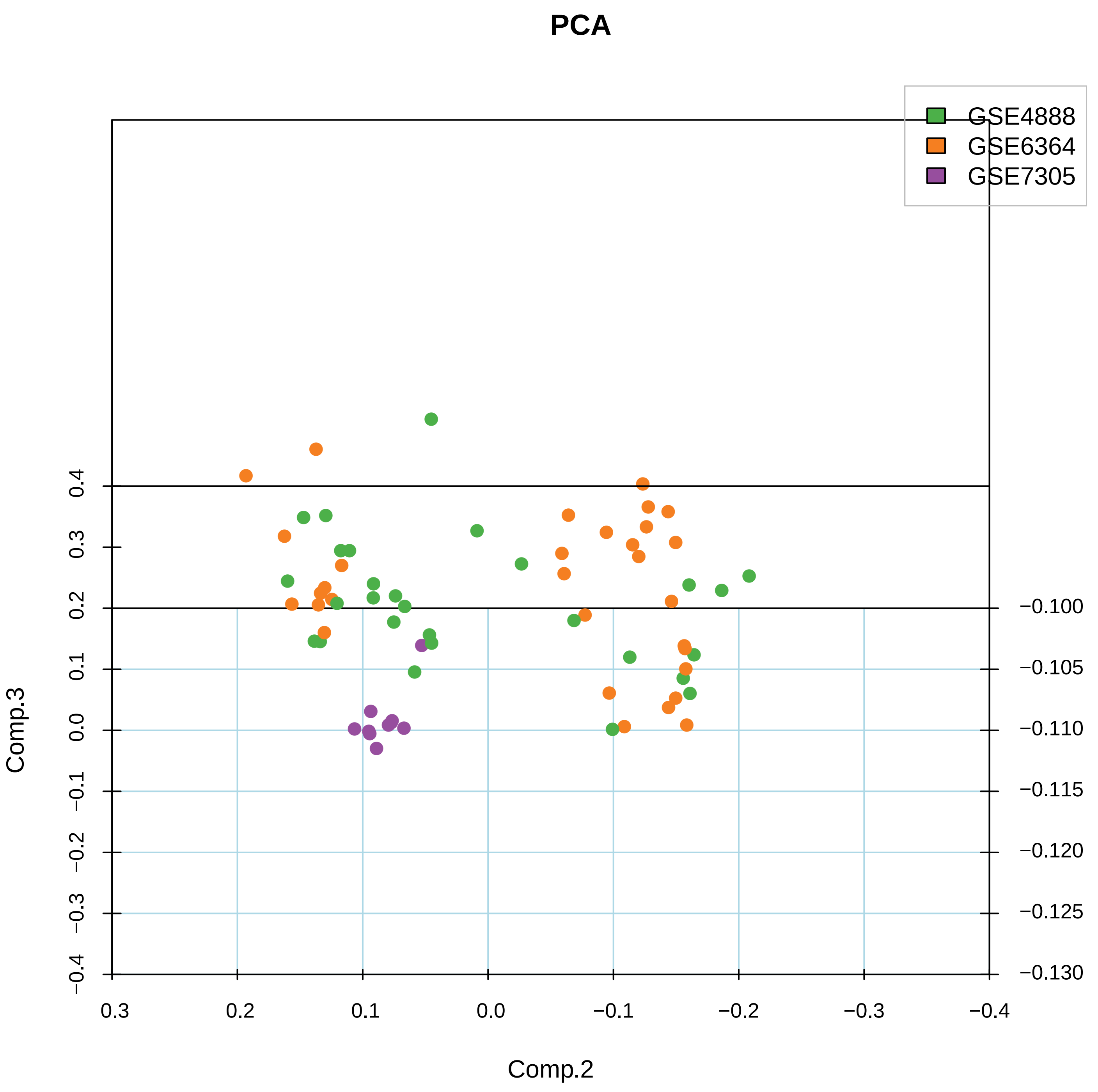
PCA
GSE4888
GSE6364
GSE7305
Comp.3
Comp.2
Comp.1
0.4
0.3
0.2
0.1
0.0
−0.1
−0.2
−0.3
−0.4
0.3
0.2
0.1
0.0
−0.1
−0.2
−0.3
−0.4
−0.100
−0.105
−0.110
−0.115
−0.120
−0.125
−0.130

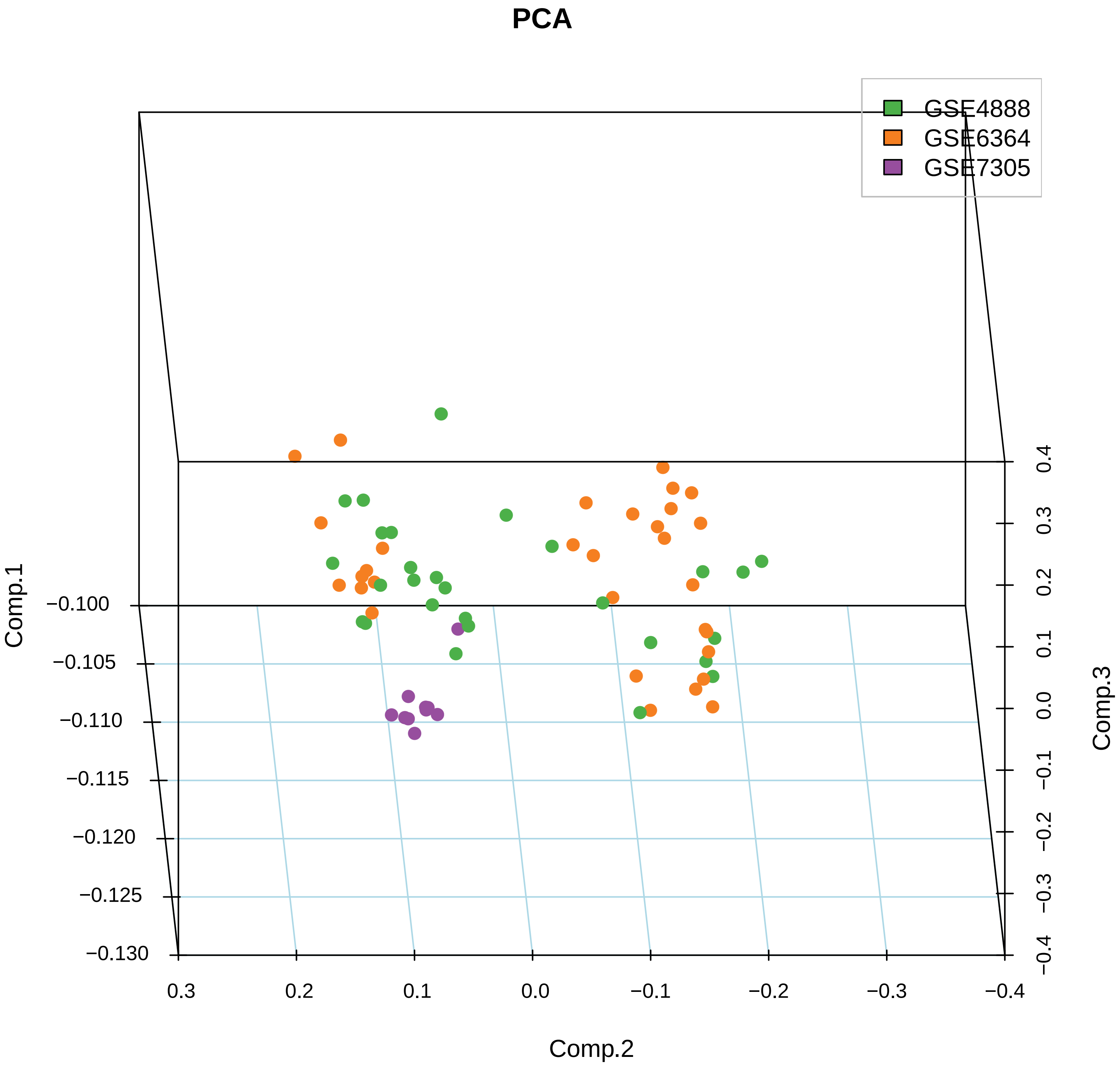

PCA
GSE4888
GSE6364
GSE7305
Comp.1
−0.100
−0.105
−0.110
−0.115
−0.120
−0.125
−0.130
0.3
0.2
0.1
0.0
−0.1
−0.2
−0.3
−0.4
Comp.2
0.4
0.3
0.2
0.1
0.0
−0.1
−0.2
−0.3
−0.4
Comp.3

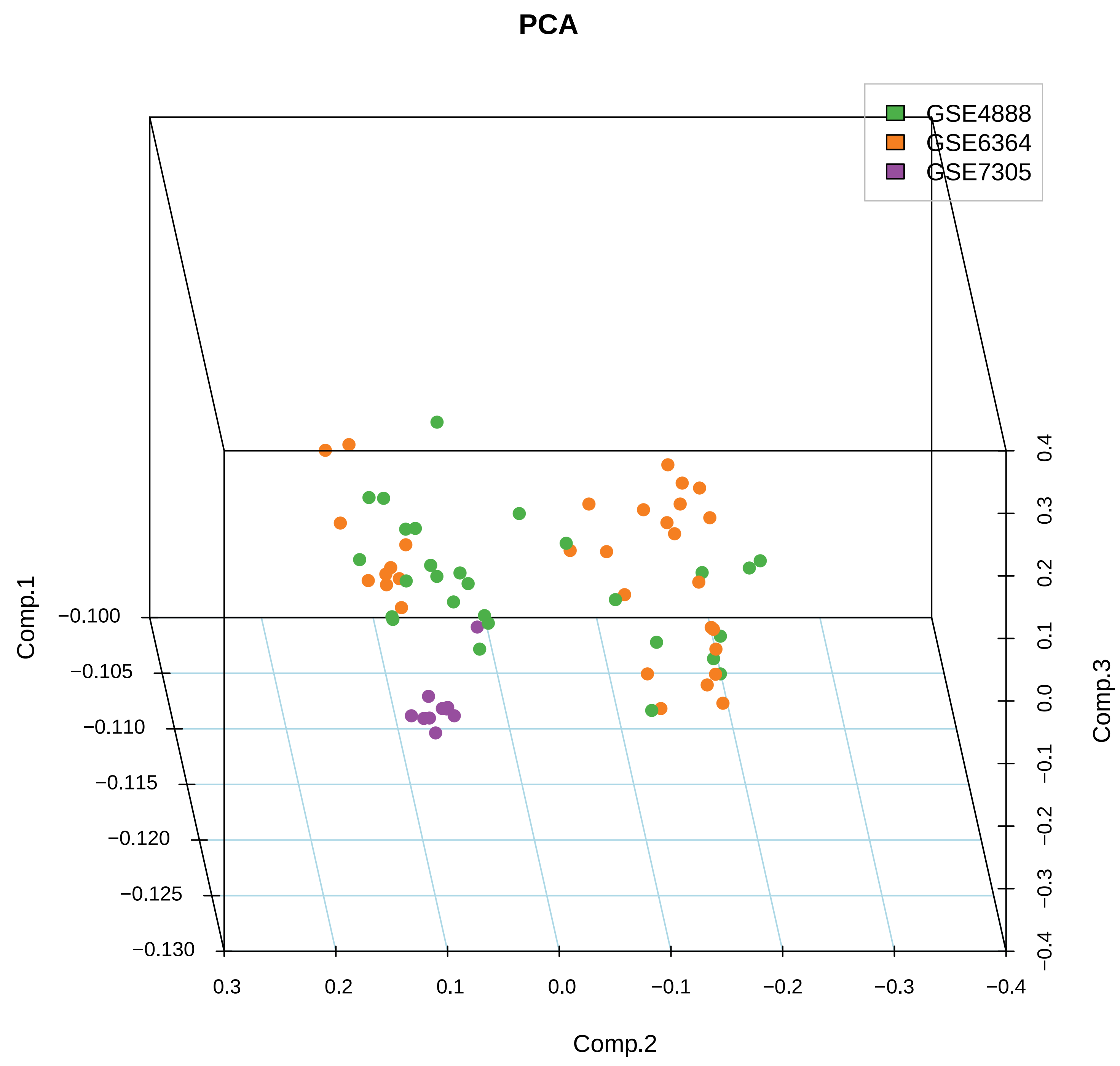
PCA
GSE4888
GSE6364
GSE7305
Comp.1
−0.100
−0.105
−0.110
−0.115
−0.120
−0.125
−0.130
Comp.2
0.3
0.2
0.1
0.0
−0.1
−0.2
−0.3
−0.4
Comp.3
0.4
0.3
0.2
0.1
0.0
−0.1
−0.2
−0.3
−0.4

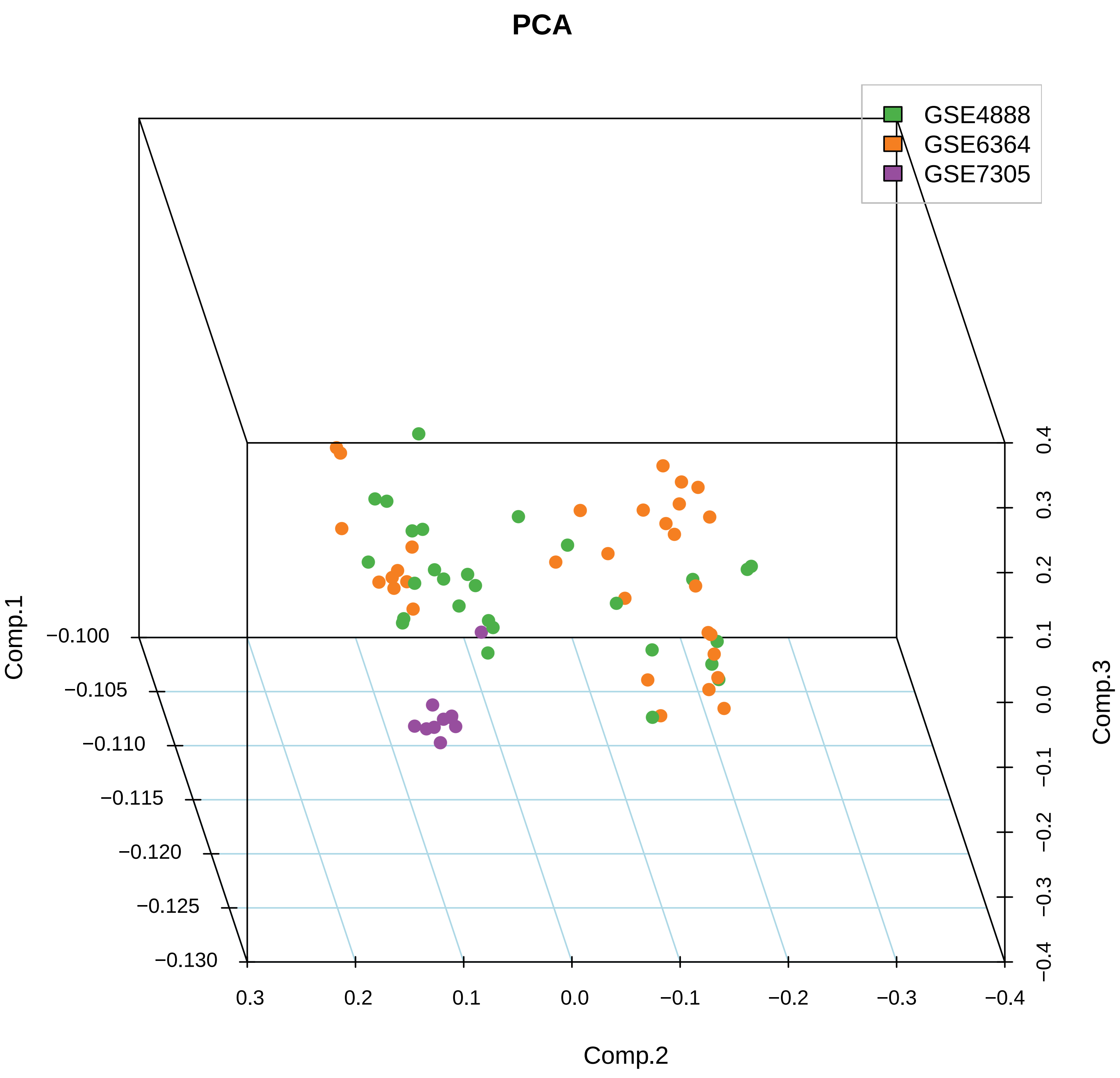

PCA
GSE4888
GSE6364
GSE7305
Comp.1
−0.100
−0.105
−0.110
−0.115
−0.120
−0.125
−0.130
Comp.2
0.3
0.2
0.1
0.0
−0.1
−0.2
−0.3
−0.4
Comp.3
0.4
0.3
0.2
0.1
0.0
−0.1
−0.2
−0.3
−0.4

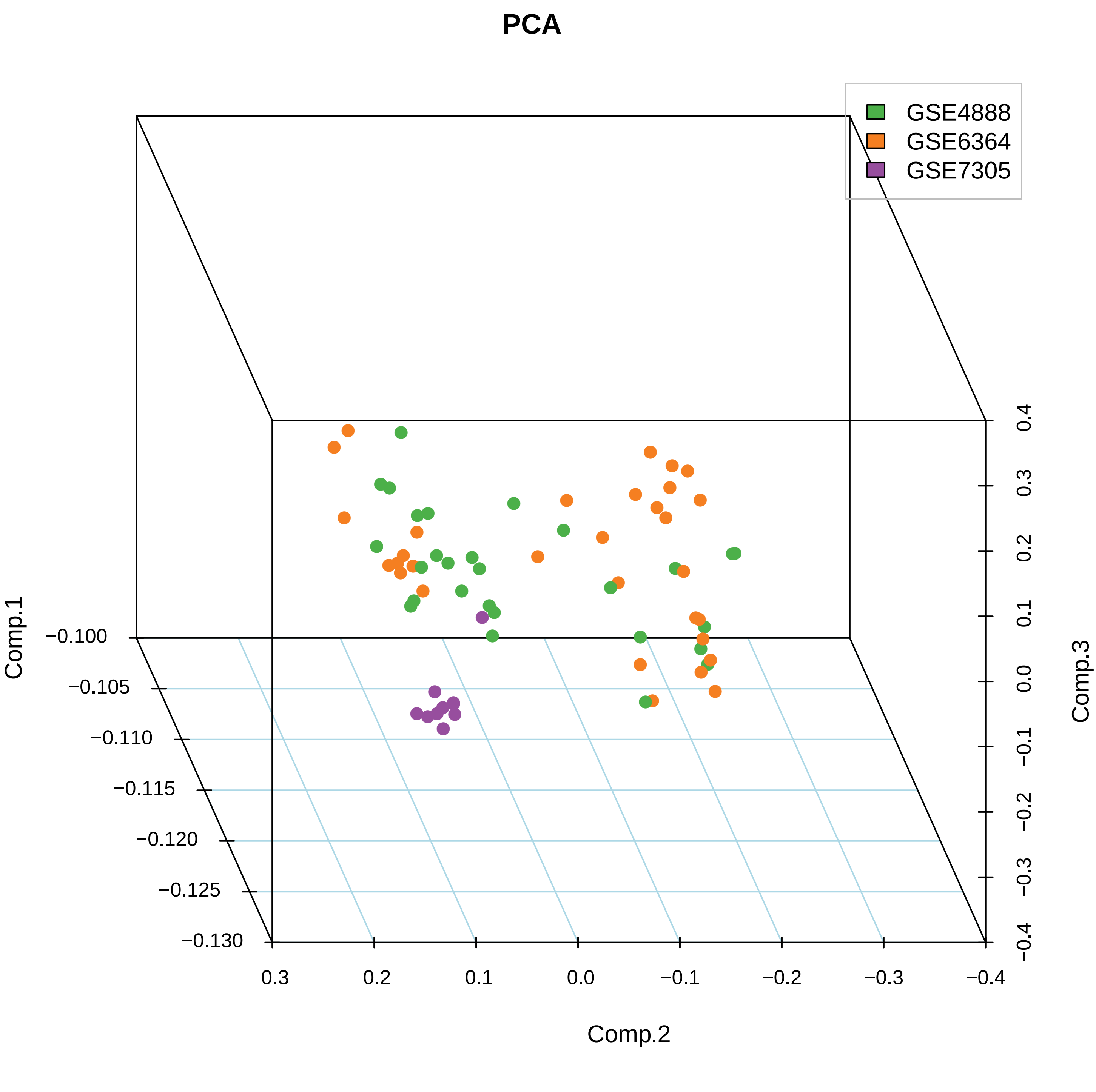

PCA
GSE4888
GSE6364
GSE7305
Comp.1
Comp.2
Comp.3
−0.100
−0.105
−0.110
−0.115
−0.120
−0.125
−0.130
0.3
0.2
0.1
0.0
−0.1
−0.2
−0.3
−0.4
0.4
0.3
0.2
0.1
0.0
−0.1
−0.2
−0.3
−0.4

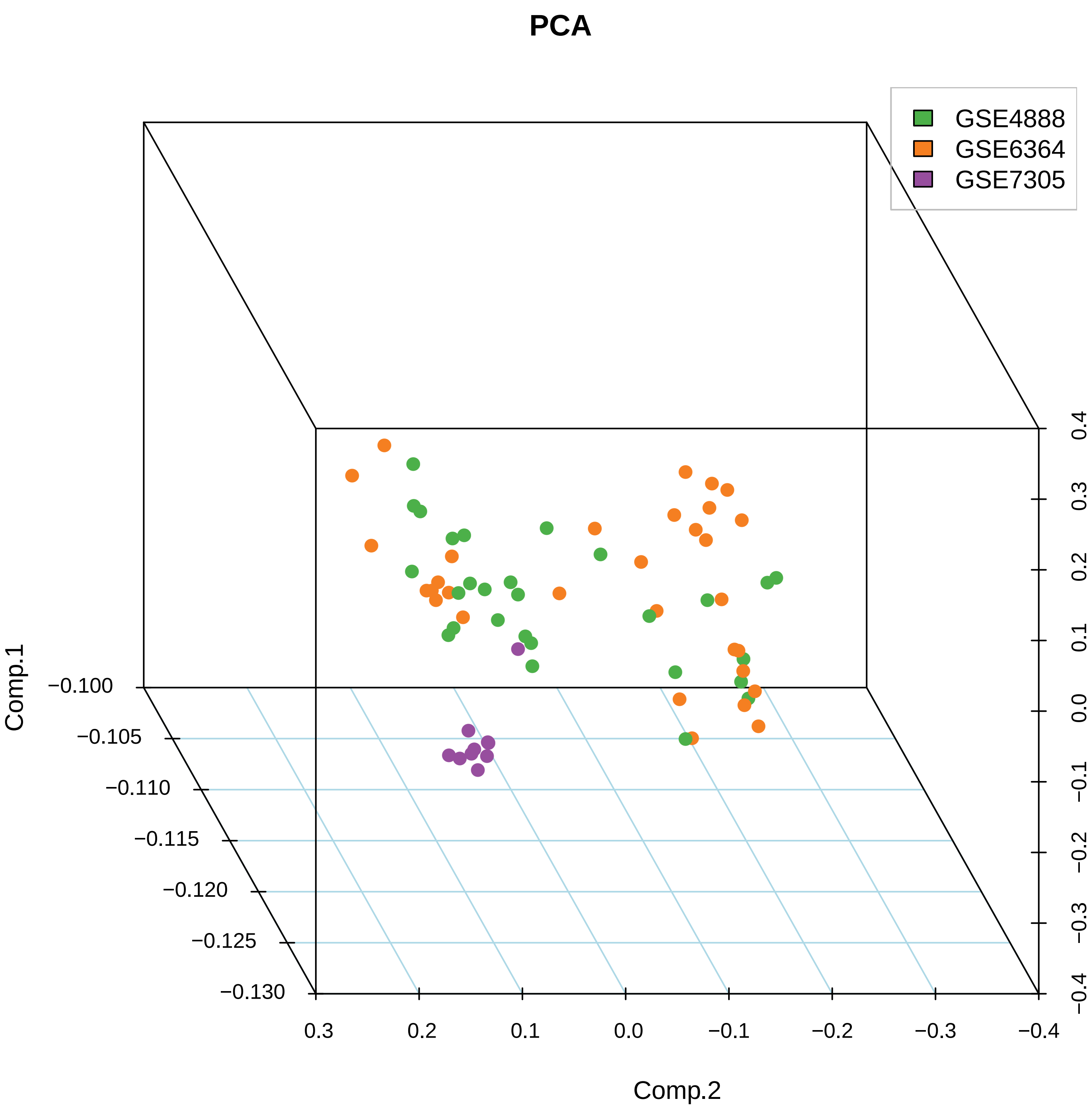
PCA
GSE4888
GSE6364
GSE7305
Comp.1
Comp.2
Comp.3
−0.100
−0.105
−0.110
−0.115
−0.120
−0.125
−0.130
0.3
0.2
0.1
0.0
−0.1
−0.2
−0.3
−0.4
0.4
0.3
0.2
0.1
0.0
−0.1
−0.2
−0.3
−0.4

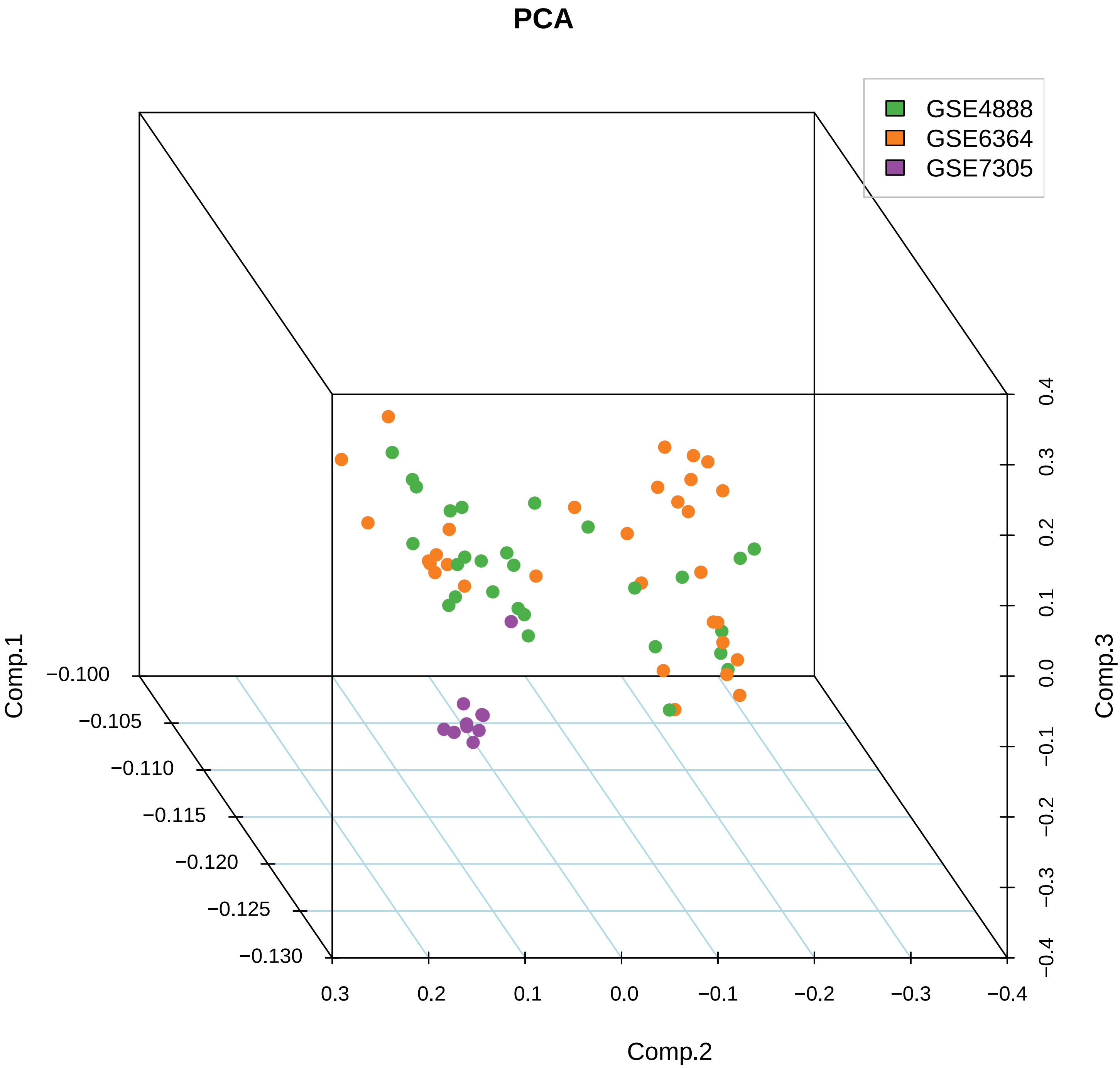

PCA
GSE4888
GSE6364
GSE7305
Comp.1
−0.100
−0.105
−0.110
−0.115
−0.120
−0.125
−0.130
0.3
0.2
0.1
0.0
−0.1
−0.2
−0.3
−0.4
Comp.2
0.4
0.3
0.2
0.1
0.0
−0.1
−0.2
−0.3
−0.4
Comp.3

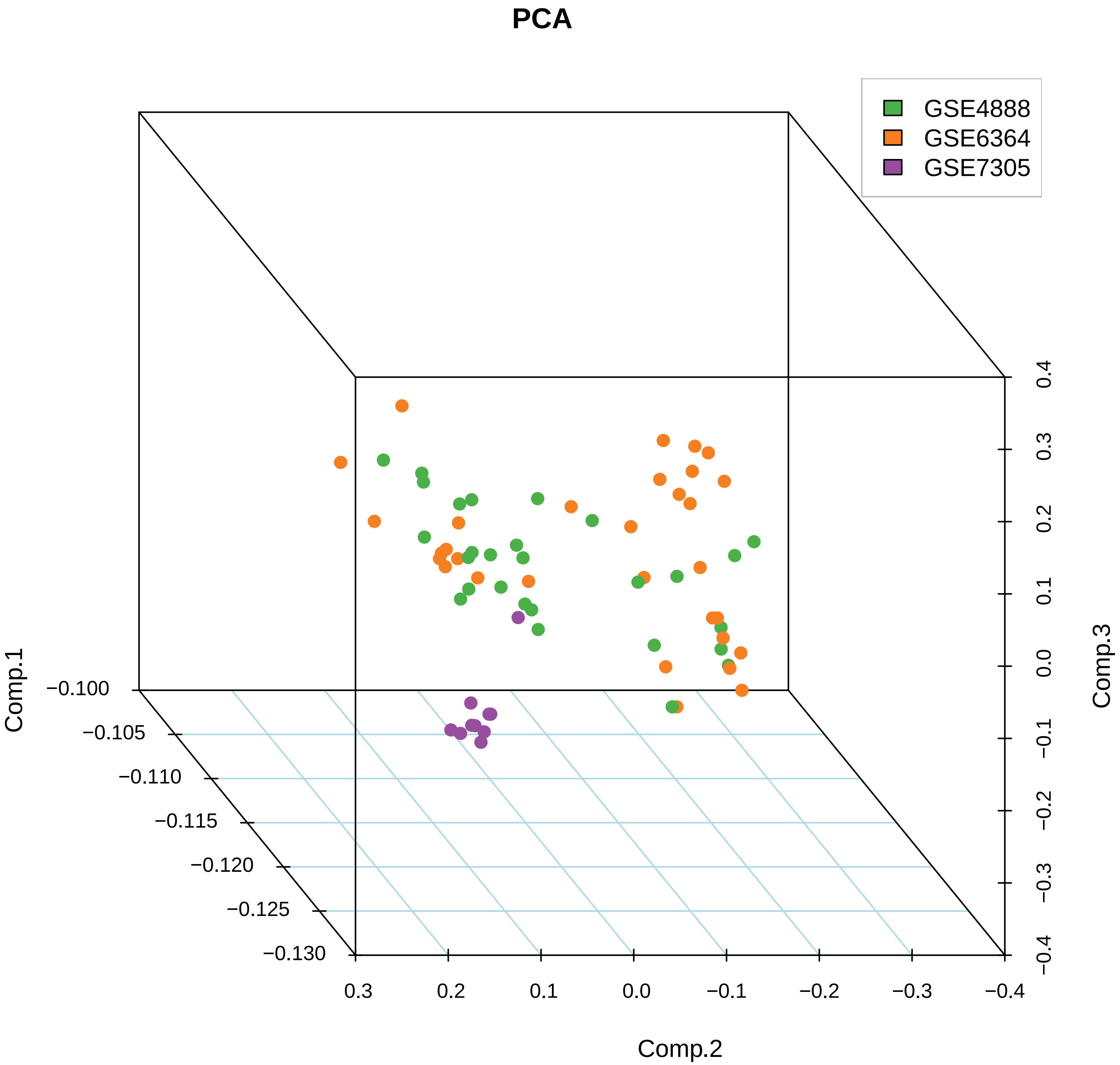
PCA
GSE4888
GSE6364
GSE7305
Comp.1
−0.100
−0.105
−0.110
−0.115
−0.120
−0.125
−0.130
Comp.2
0.3
0.2
0.1
0.0
−0.1
−0.2
−0.3
−0.4
Comp.3
0.4
0.3
0.2
0.1
0.0
−0.1
−0.2
−0.3
−0.4

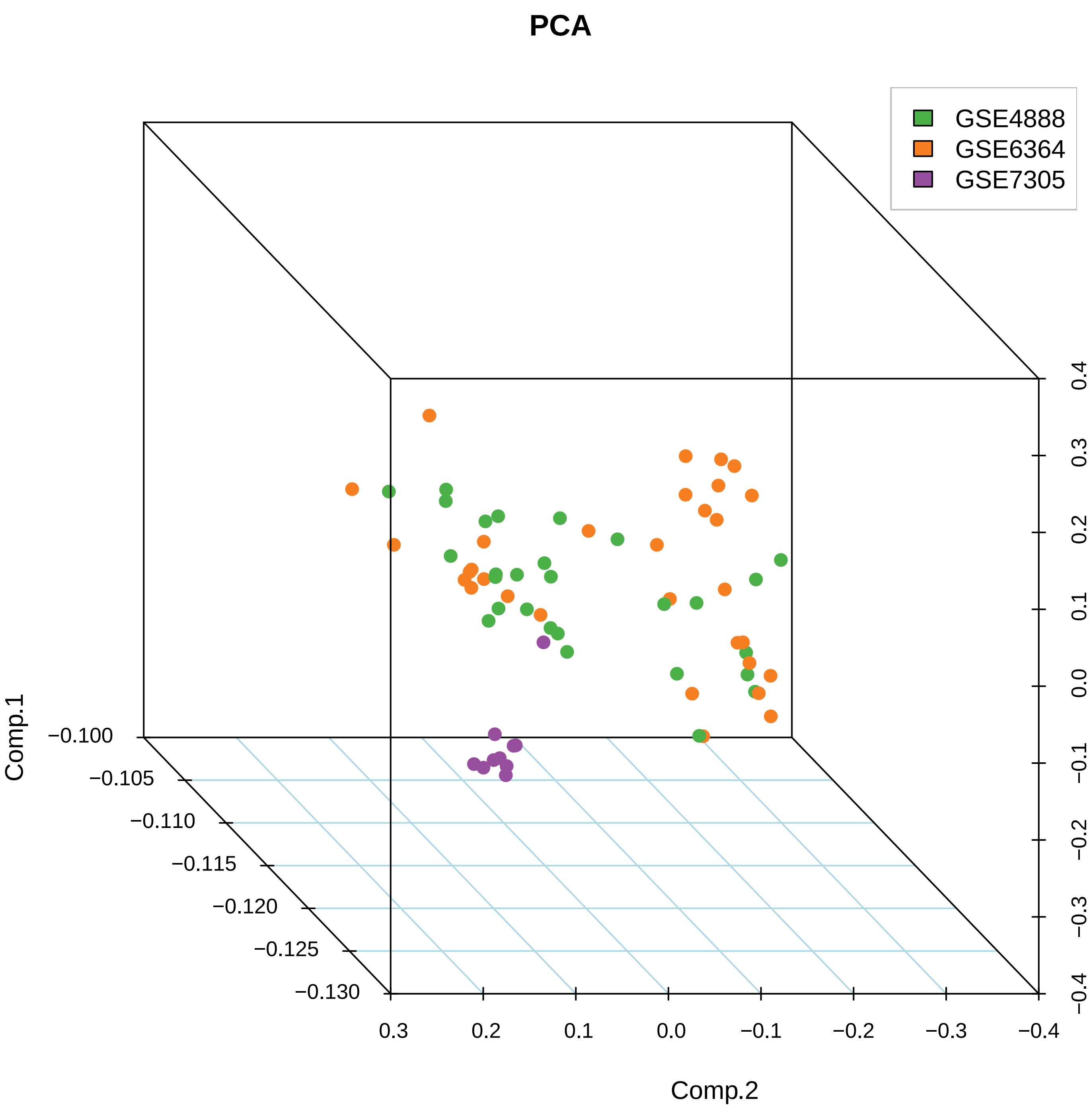

PCA
GSE4888
GSE6364
GSE7305
Comp.1
−0.100
−0.105
−0.110
−0.115
−0.120
−0.125
−0.130
Comp.2
0.3
0.2
0.1
0.0
−0.1
−0.2
−0.3
−0.4
Comp.3
0.4
0.3
0.2
0.1
0.0
−0.1
−0.2
−0.3
−0.4

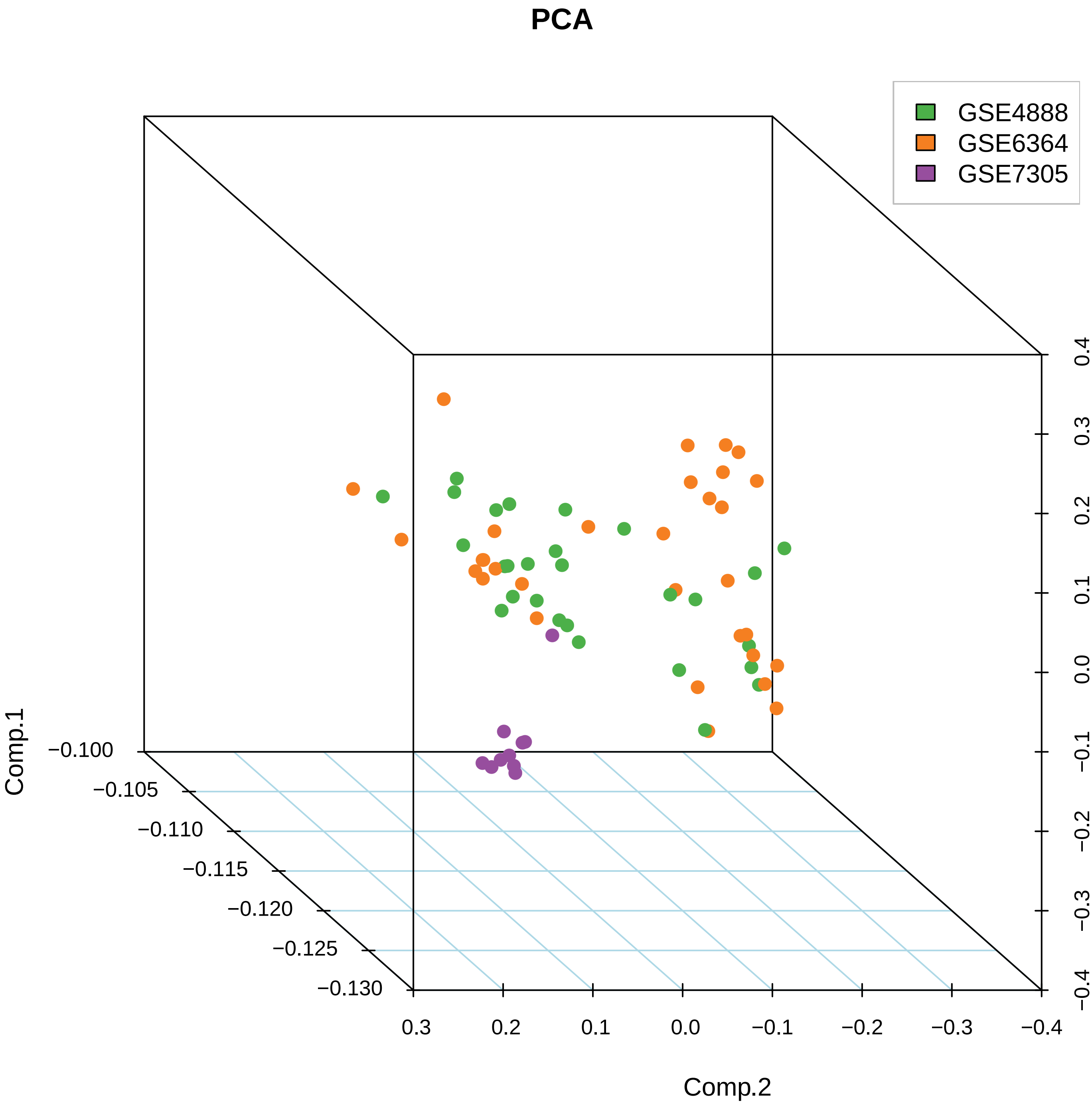

PCA
GSE4888
GSE6364
GSE7305
Comp.1
−0.100
−0.105
−0.110
−0.115
−0.120
−0.125
−0.130
Comp.2
0.3
0.2
0.1
0.0
−0.1
−0.2
−0.3
−0.4
Comp.3
0.4
0.3
0.2
0.1
0.0
−0.1
−0.2
−0.3
−0.4

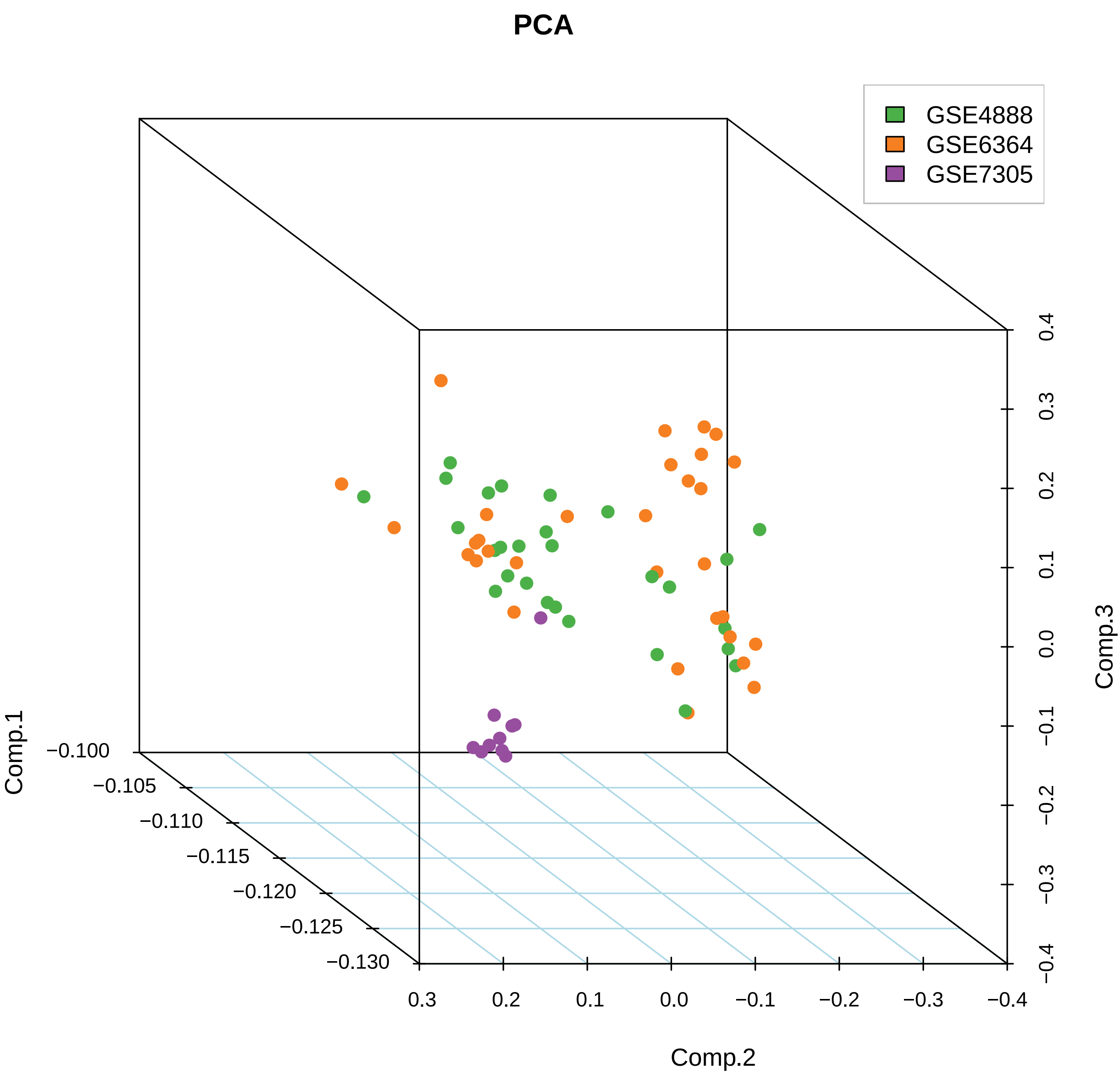
PCA
GSE4888
GSE6364
GSE7305
Comp.1
−0.100
−0.105
−0.110
−0.115
−0.120
−0.125
−0.130
Comp.2
0.3
0.2
0.1
0.0
−0.1
−0.2
−0.3
−0.4
Comp.3
0.4
0.3
0.2
0.1
0.0
−0.1
−0.2
−0.3
−0.4

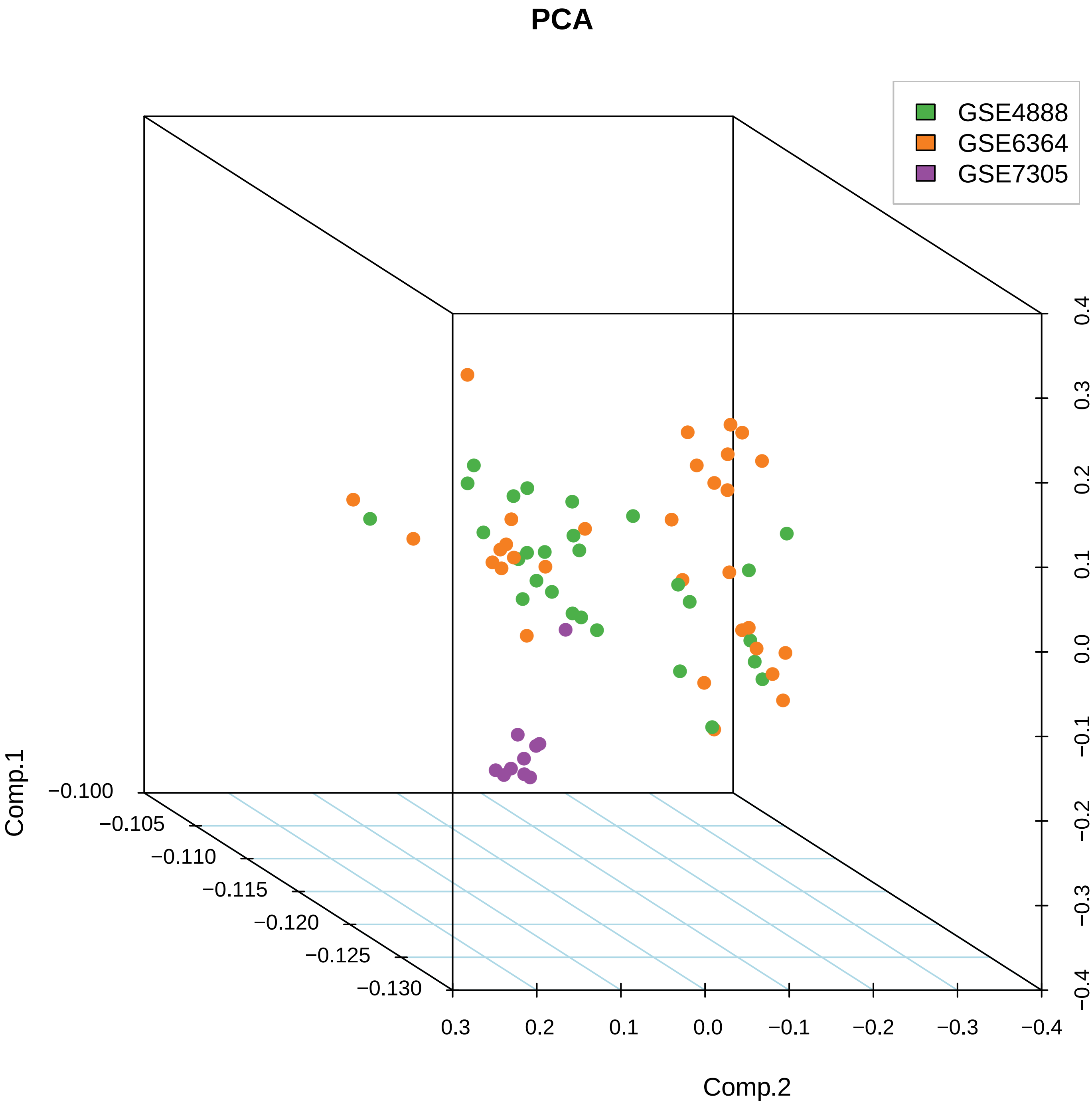

PCA
GSE4888
GSE6364
GSE7305
Comp.1
−0.100
−0.105
−0.110
−0.115
−0.120
−0.125
−0.130
Comp.2
0.3
0.2
0.1
0.0
−0.1
−0.2
−0.3
−0.4
Comp.3
0.4
0.3
0.2
0.1
0.0
−0.1
−0.2
−0.3
−0.4

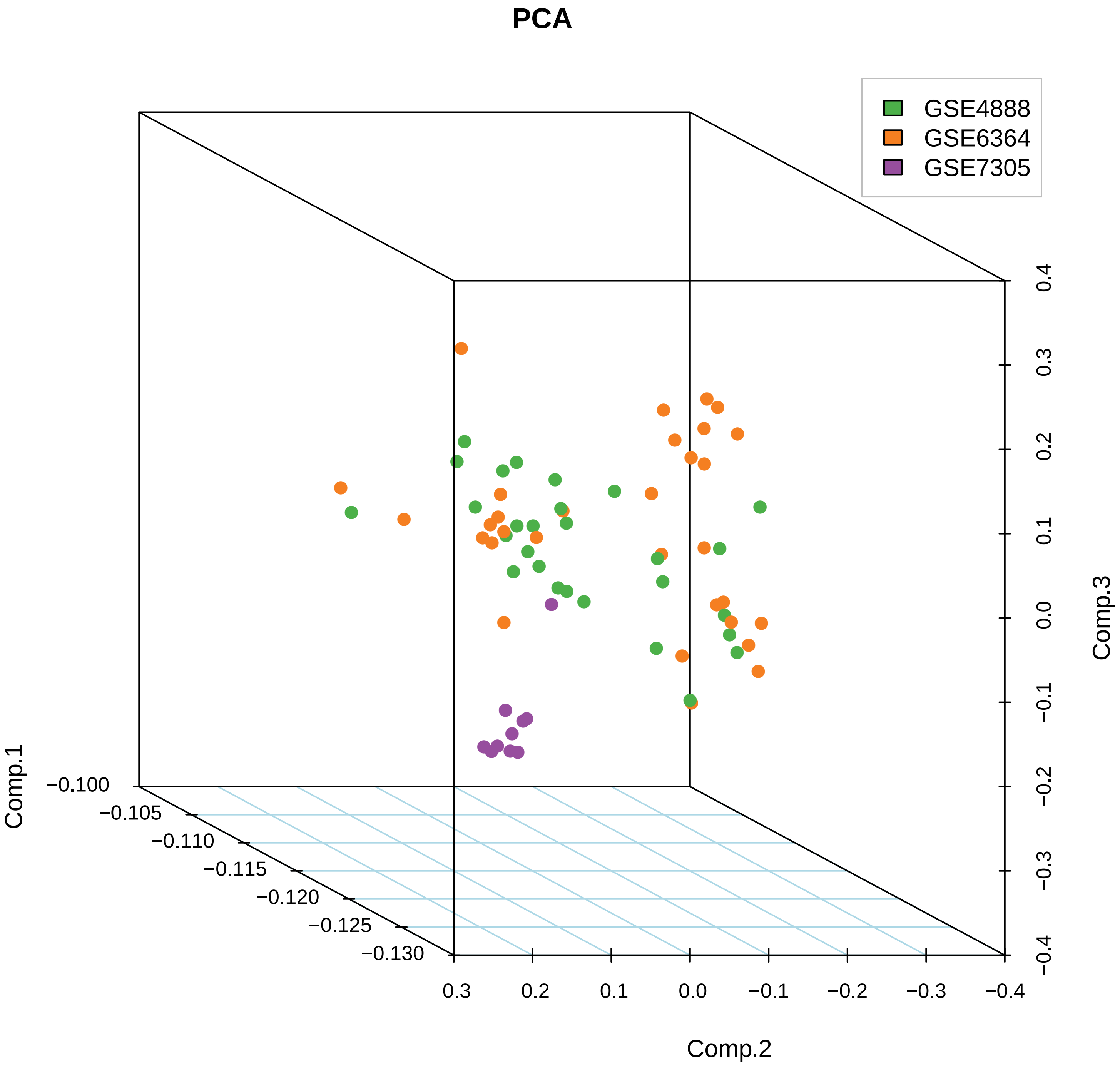

PCA
GSE4888
GSE6364
GSE7305
Comp.1
Comp.2
Comp.3
−0.100
−0.105
−0.110
−0.115
−0.120
−0.125
−0.130
0.3
0.2
0.1
0.0
−0.1
−0.2
−0.3
−0.4
0.4

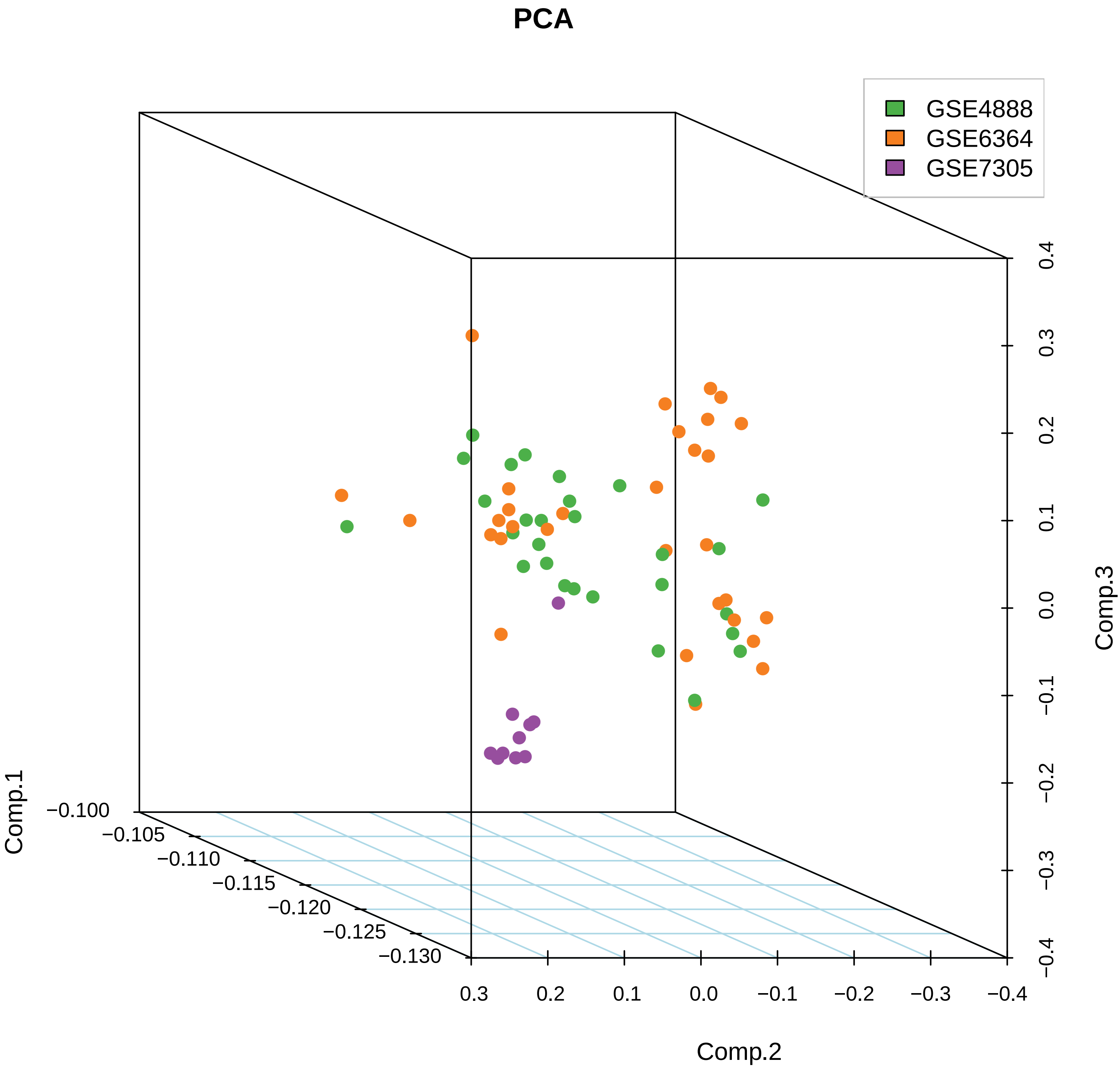

PCA
GSE4888
GSE6364
GSE7305
Comp.1
−0.100
−0.105
−0.110
−0.115
−0.120
−0.125
−0.130
Comp.2
0.3
0.2
0.1
0.0
−0.1
−0.2
−0.3
−0.4
Comp.3
0.4
0.3
0.2
0.1
0.0
−0.1
−0.2
−0.3
−0.4

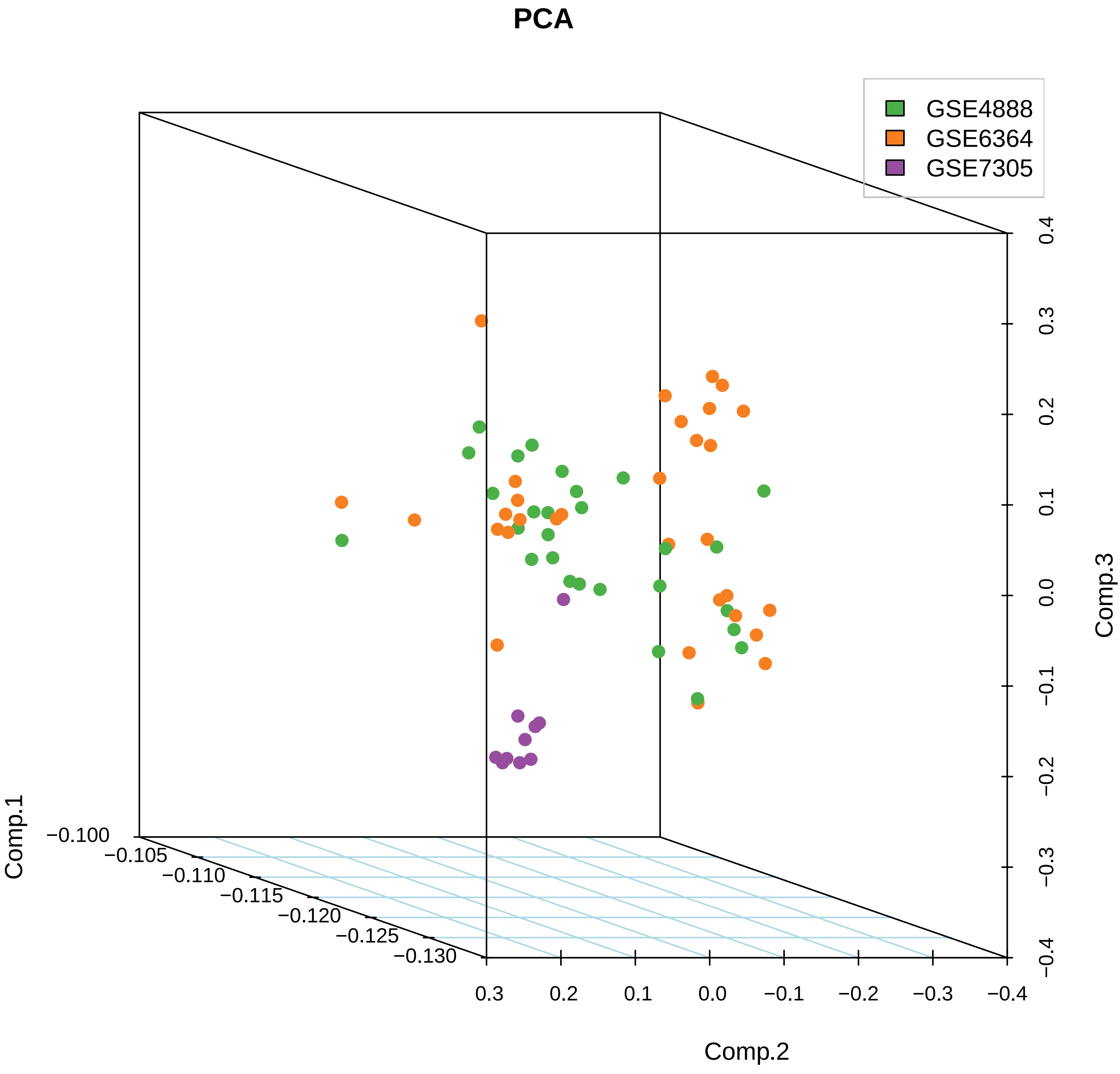

PCA
GSE4888
GSE6364
GSE7305
Comp.1
Comp.2
Comp.3
−0.100
−0.105
−0.110
−0.115
−0.120
−0.125
−0.130
0.3
0.2
0.1
0.0
−0.1
−0.2
−0.3
−0.4
0.4

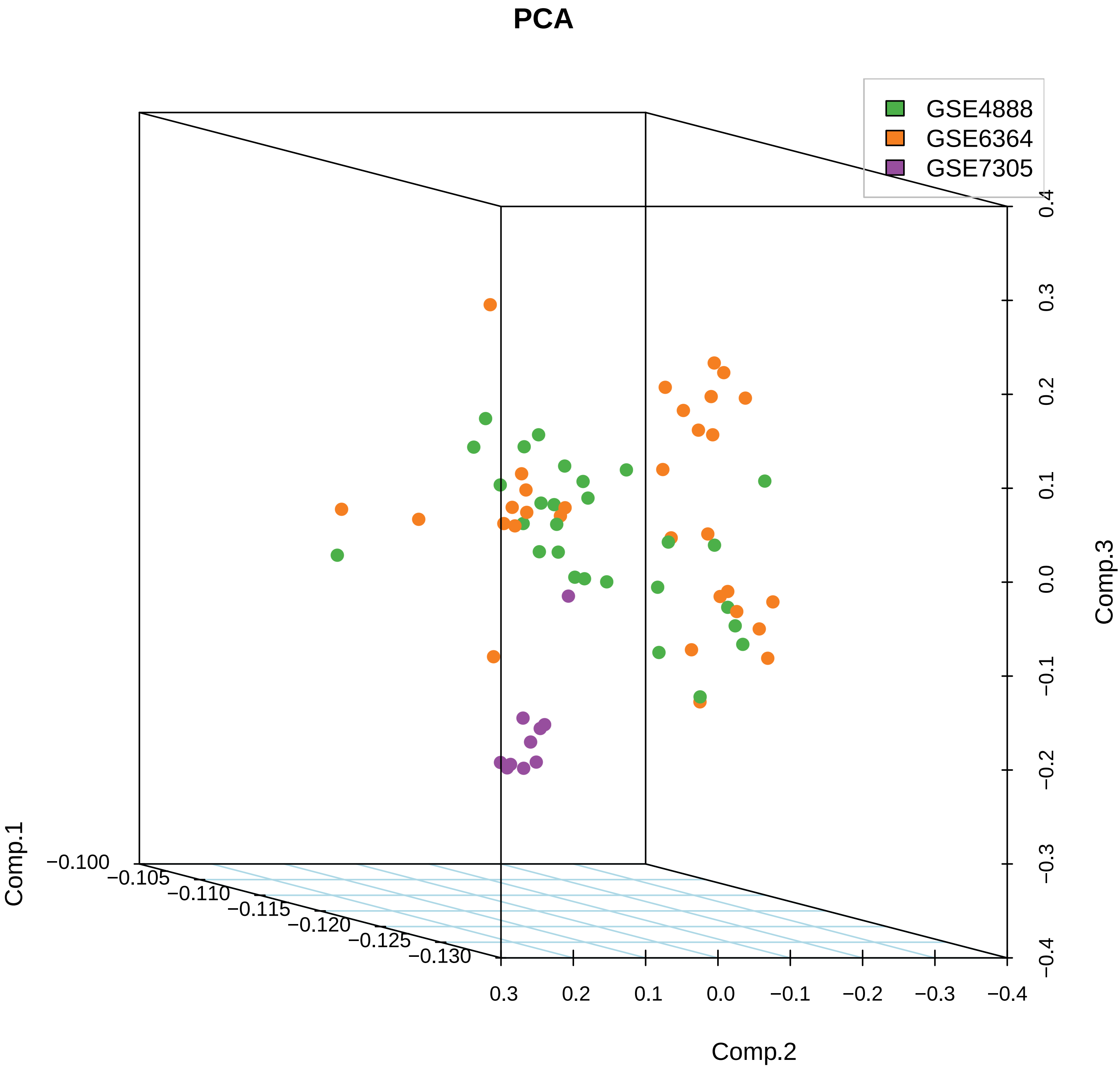
PCA
GSE4888
GSE6364
GSE7305
Comp.1
Comp.2
Comp.3
−0.100
−0.105
−0.110
−0.115
−0.120
−0.125
−0.130
0.3
0.2
0.1
0.0
−0.1
−0.2
−0.3
−0.4
0.4
0.3
0.2
0.1
0.0
−0.1
−0.2
−0.3
−0.4

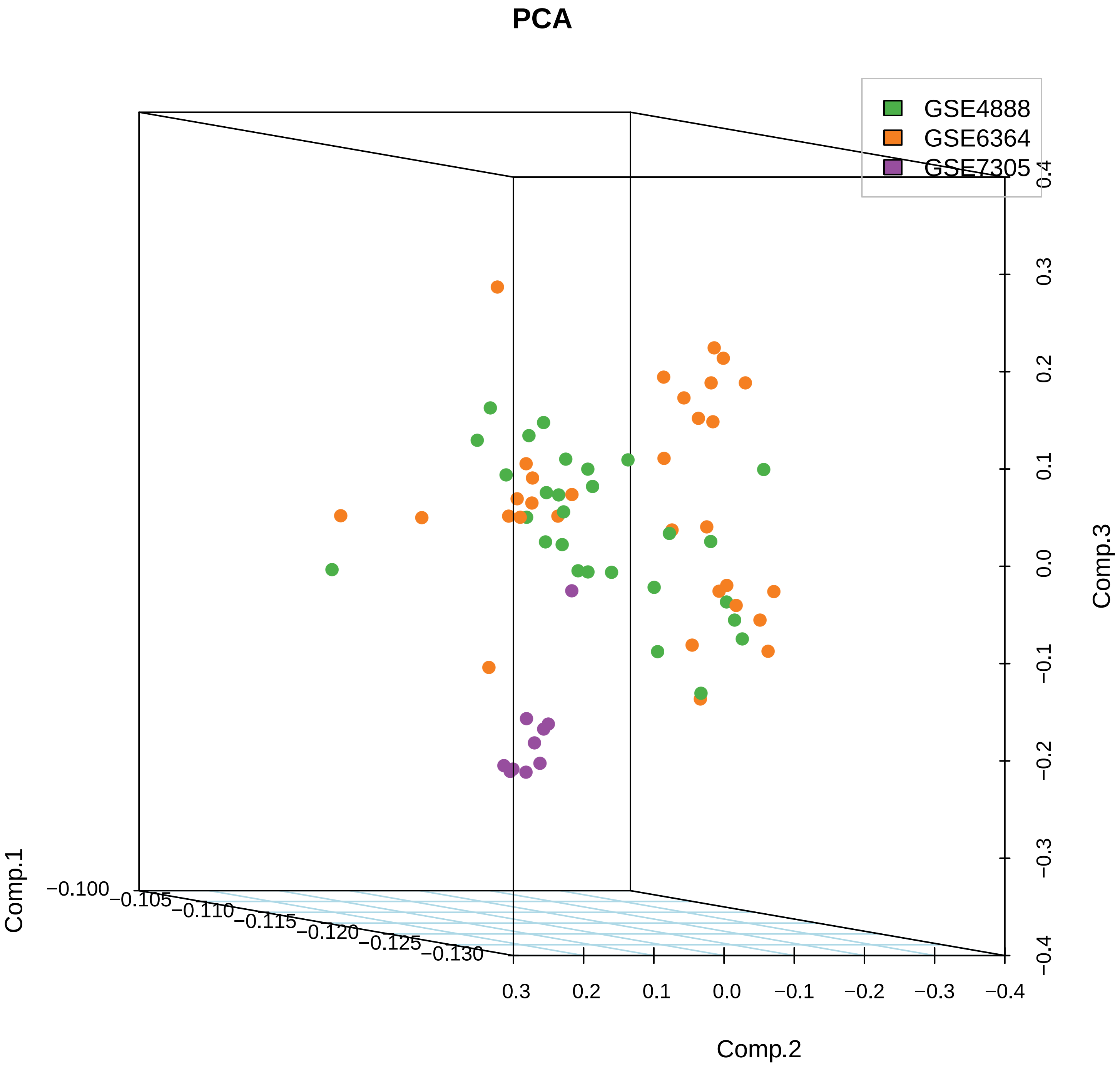
PCA
GSE4888
GSE6364
GSE7305
Comp.1
Comp.2
Comp.3
−0.100
−0.105
−0.110
−0.115
−0.120
−0.125
−0.130
0.3
0.2
0.1
0.0
−0.1
−0.2
−0.3
−0.4
0.4
0.3
0.2
0.1
0.0
−0.1
−0.2
−0.3
−0.4

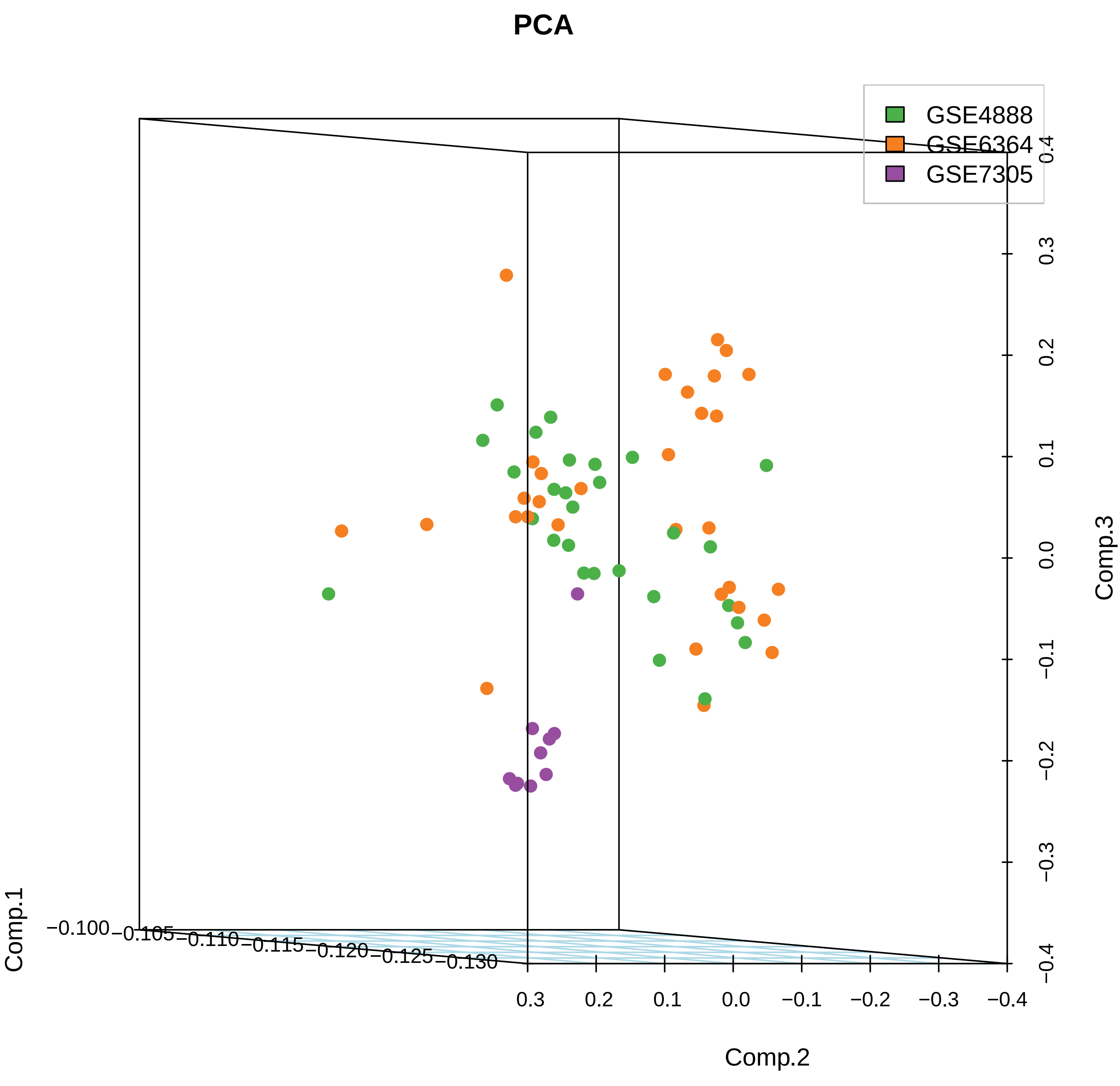
PCA
GSE4888
GSE6364
GSE7305
Comp.1
Comp.2
Comp.3
−0.100
−0.105
−0.110
−0.115
−0.120
−0.125
−0.130
0.3
0.2
0.1
0.0
−0.1
−0.2
−0.3
−0.4
0.4
0.3
0.2
0.1
0.0
−0.1
−0.2
−0.3
−0.4

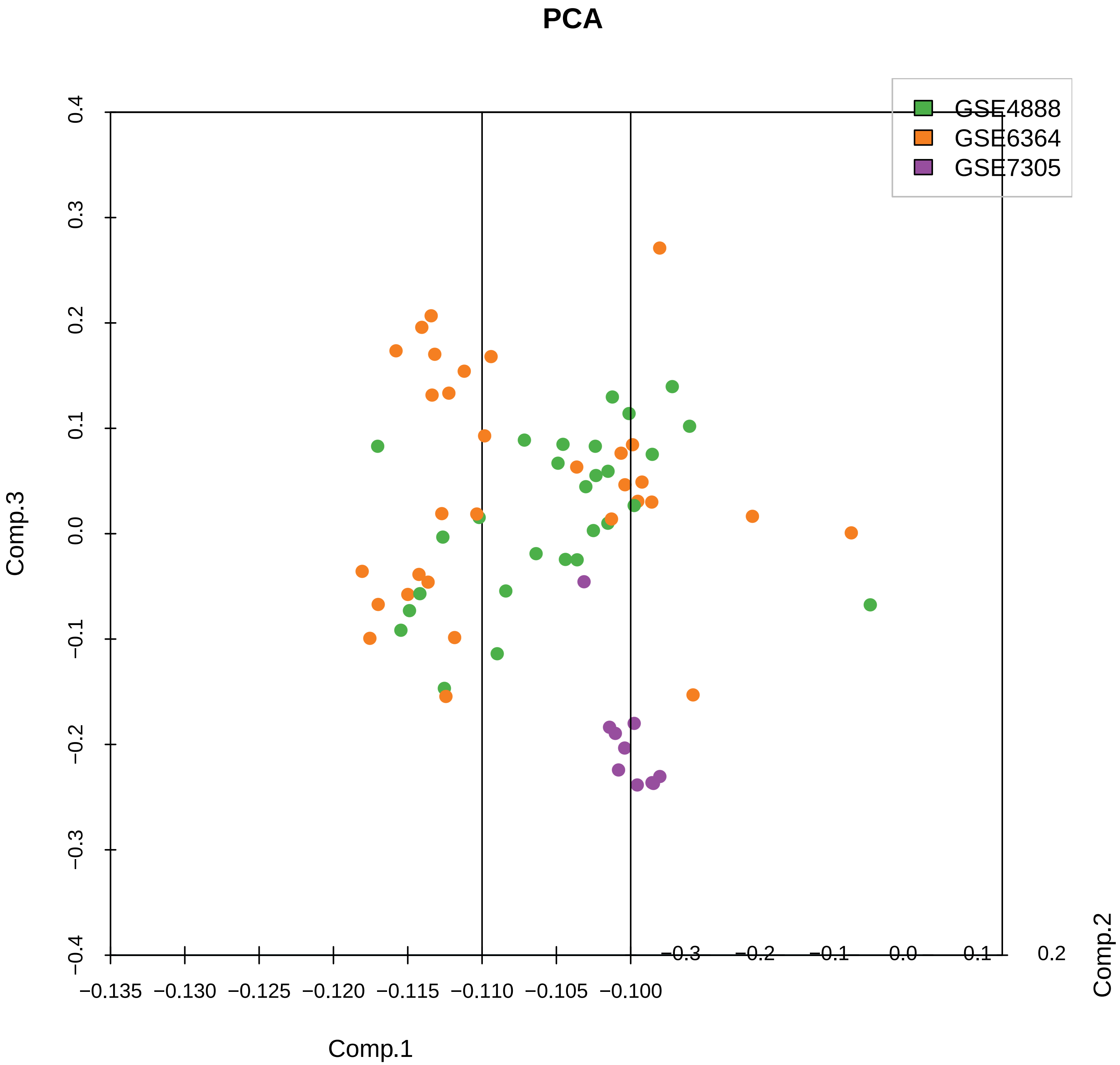
PCA
GSE4888
GSE6364
GSE7305
Comp.3
Comp.1
Comp.2
0.4
0.3
0.2
0.1
0.0
−0.1
−0.2
−0.3
−0.4
−0.135 −0.130 −0.125 −0.120 −0.115 −0.110 −0.105 −0.100
−0.3 −0.2 −0.1 0.0 0.1 0.2

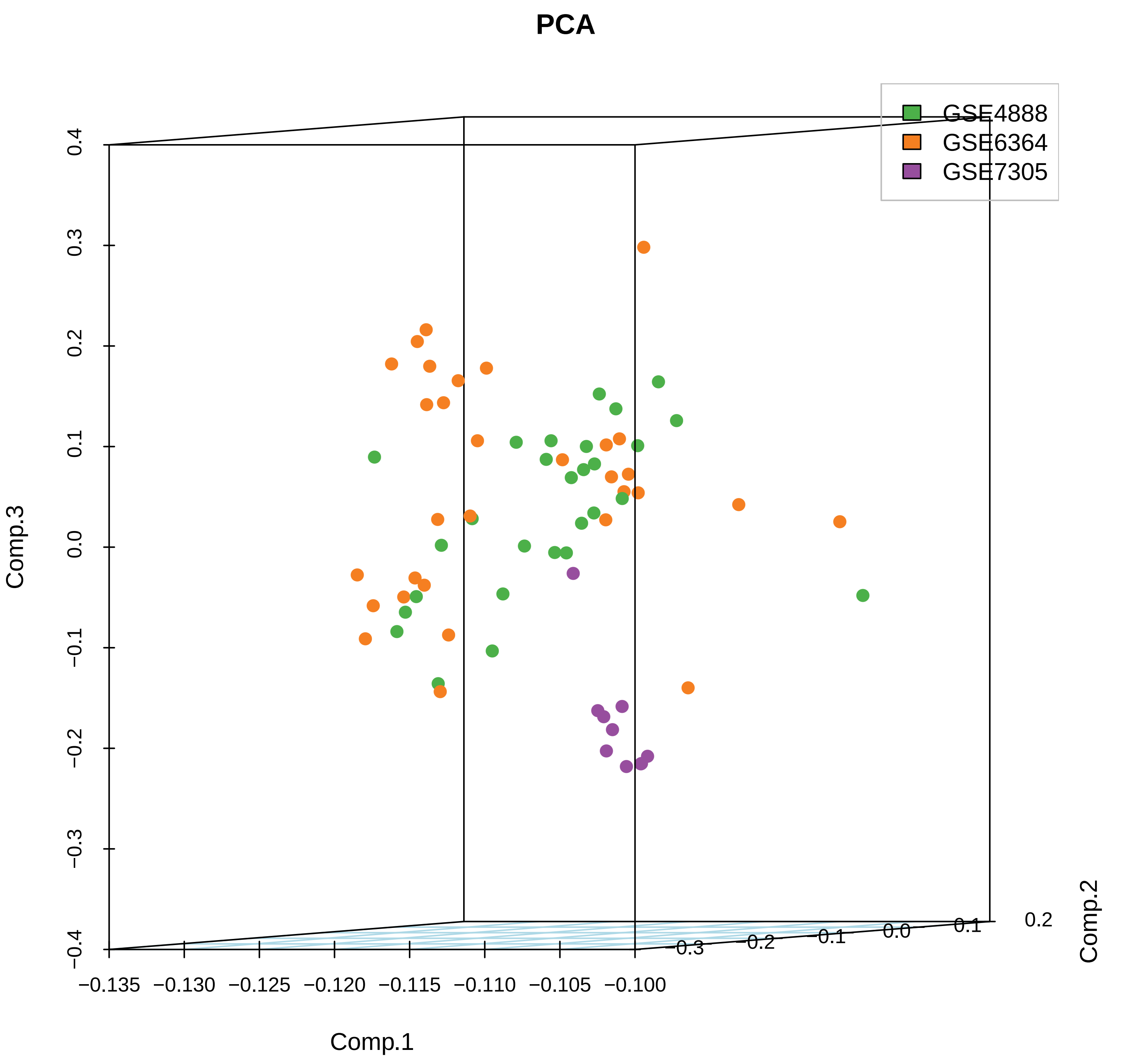

PCA
GSE4888
GSE6364
GSE7305
Comp.3
Comp.1
Comp.2
0.4
0.3
0.2
0.1
0.0
−0.1
−0.2
−0.3
−0.4
−0.135
−0.130
−0.125
−0.120
−0.115
−0.110
−0.105
−0.100
−0.3
−0.2
−0.1
0.0
0.1
0.2

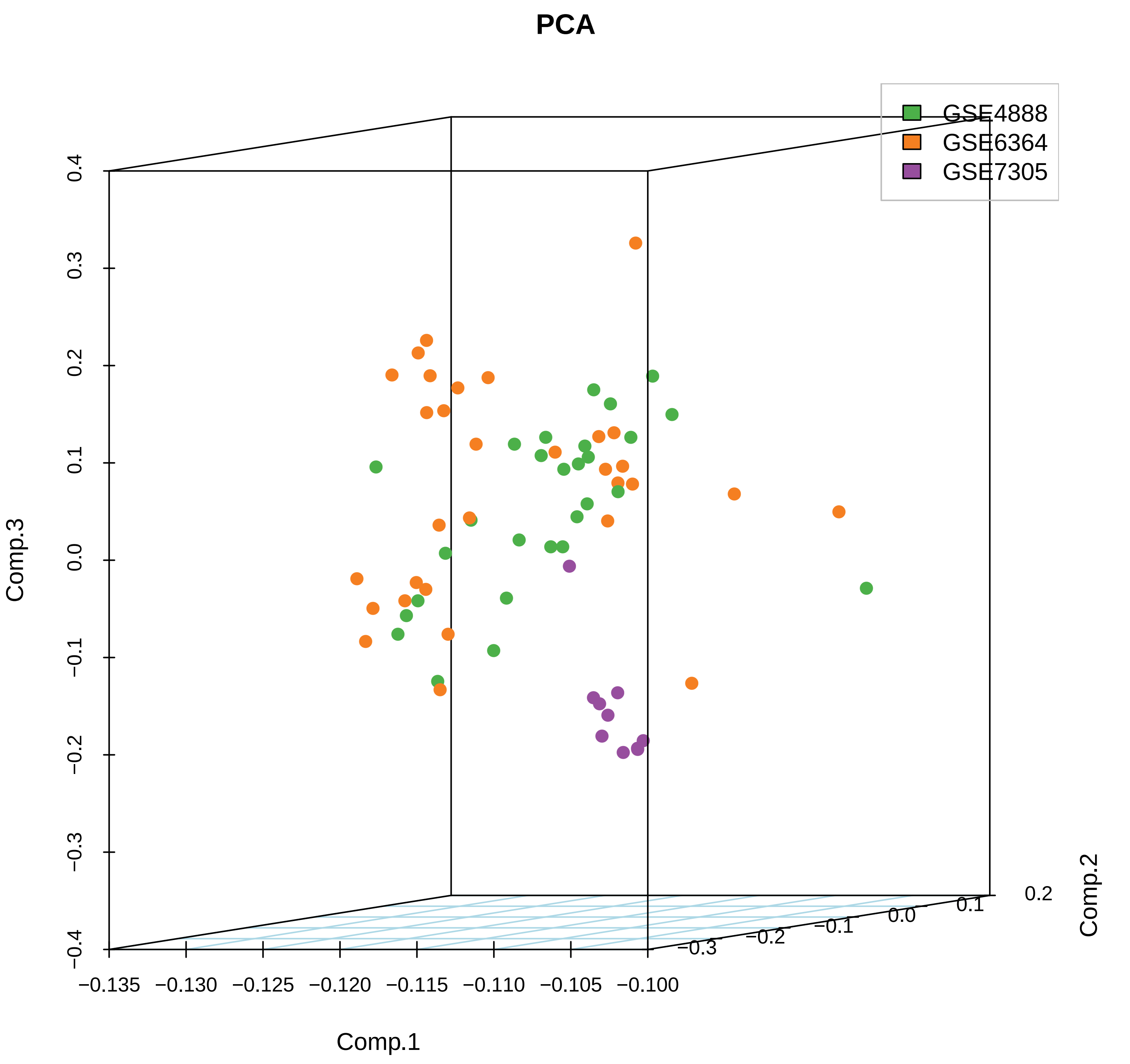
PCA
GSE4888
GSE6364
GSE7305
Comp.3
0.4
0.3
0.2
0.1
0.0
−0.1
−0.2
−0.3
−0.4
Comp.1
−0.135
−0.130
−0.125
−0.120
−0.115
−0.110
−0.105
−0.100
Comp.2
−0.3
−0.2
−0.1
0.0
0.1
0.2

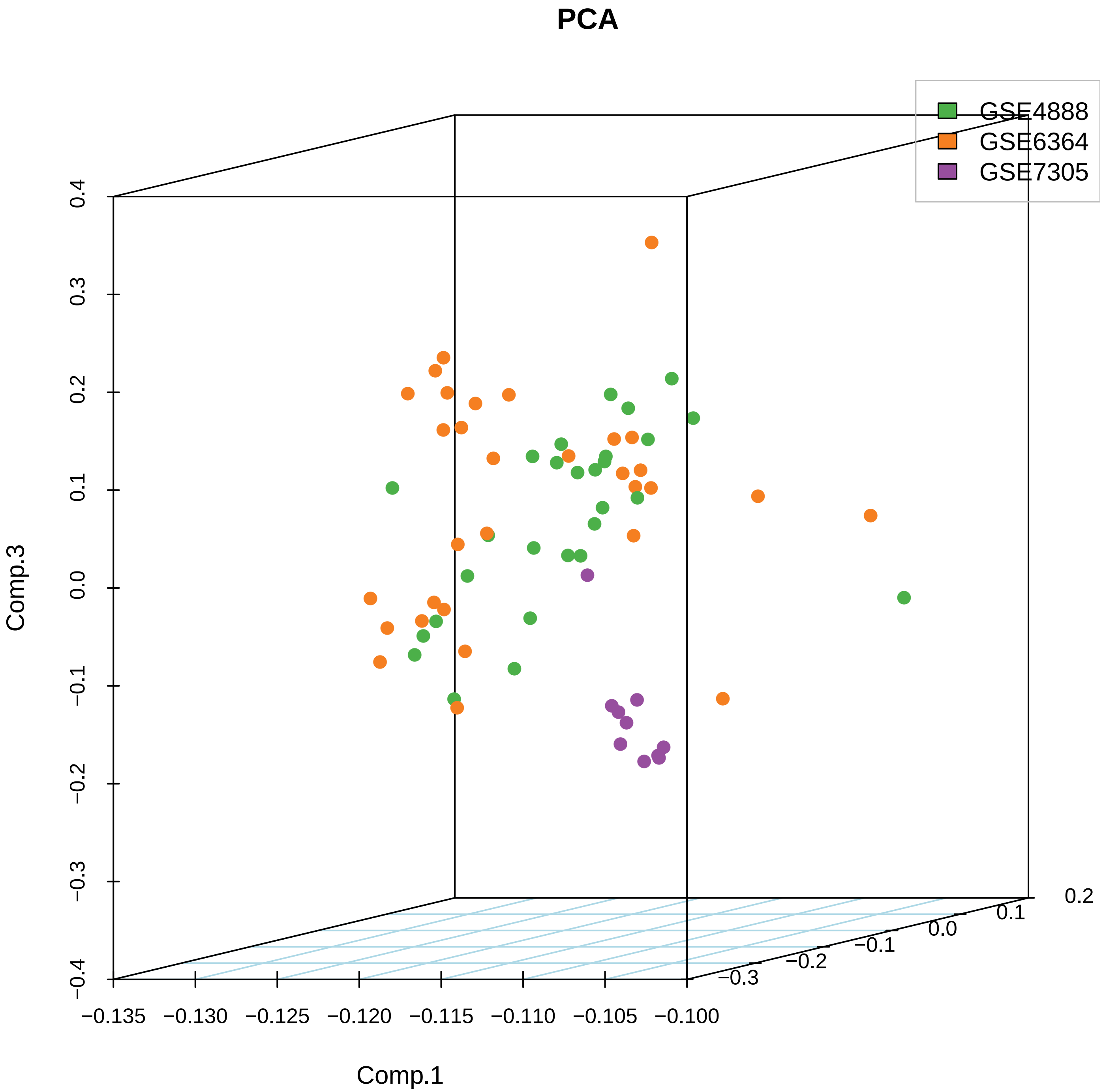
PCA
GSE4888
GSE6364
GSE7305
Comp.1
Comp.2
Comp.3
−0.135
−0.130
−0.125
−0.120
−0.115
−0.110
−0.105
−0.100
−0.3
−0.2
−0.1
0.0
0.1
0.2
−0.4
−0.3
−0.2
−0.1
0.0
0.1
0.2
0.3
0.4

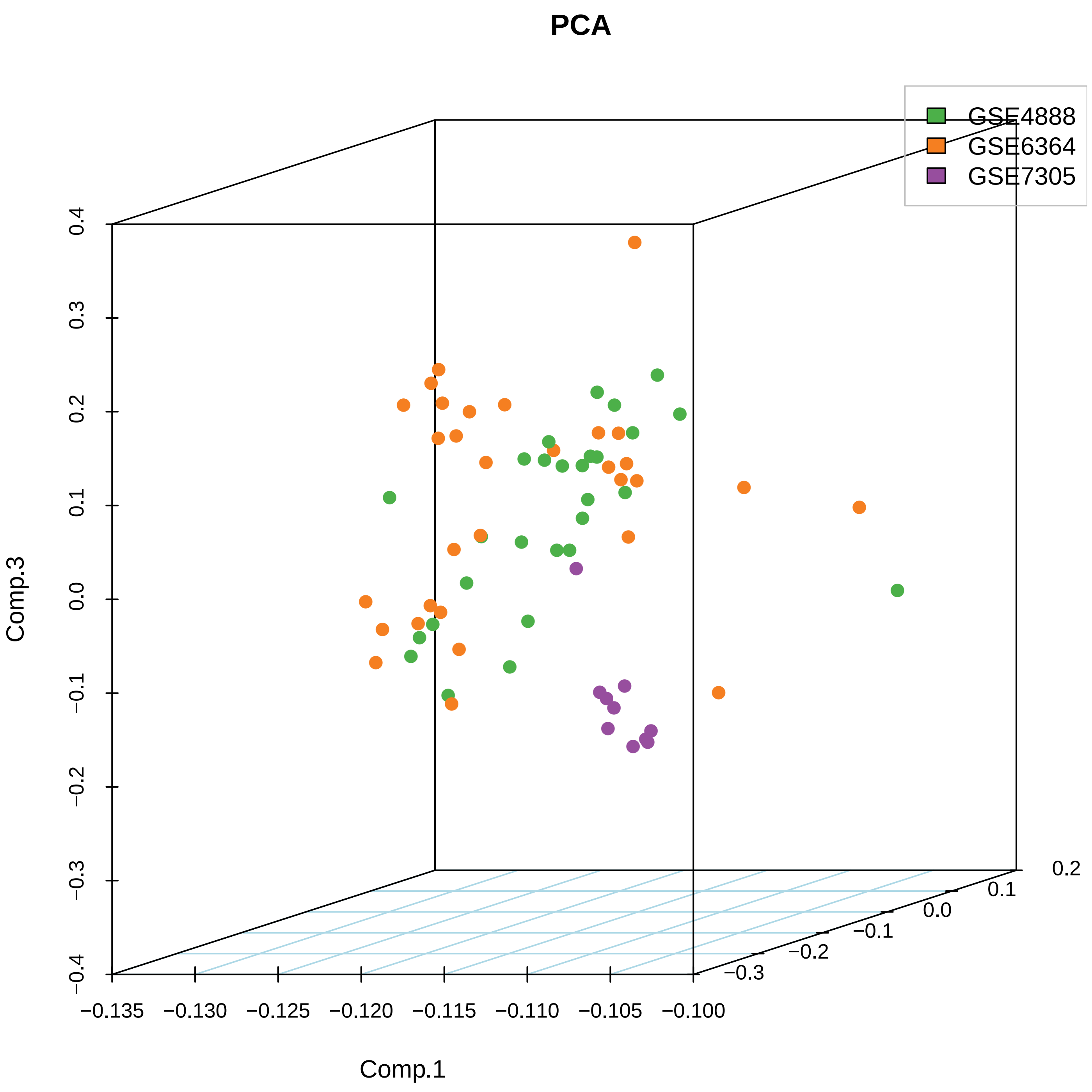
PCA
GSE4888
GSE6364
GSE7305
Comp.3
0.4
0.3
0.2
0.1
0.0
−0.1
−0.2
−0.3
−0.4
Comp.1
−0.135
−0.130
−0.125
−0.120
−0.115
−0.110
−0.105
−0.100
Comp.2
0.2
0.1
0.0
−0.1
−0.2
−0.3

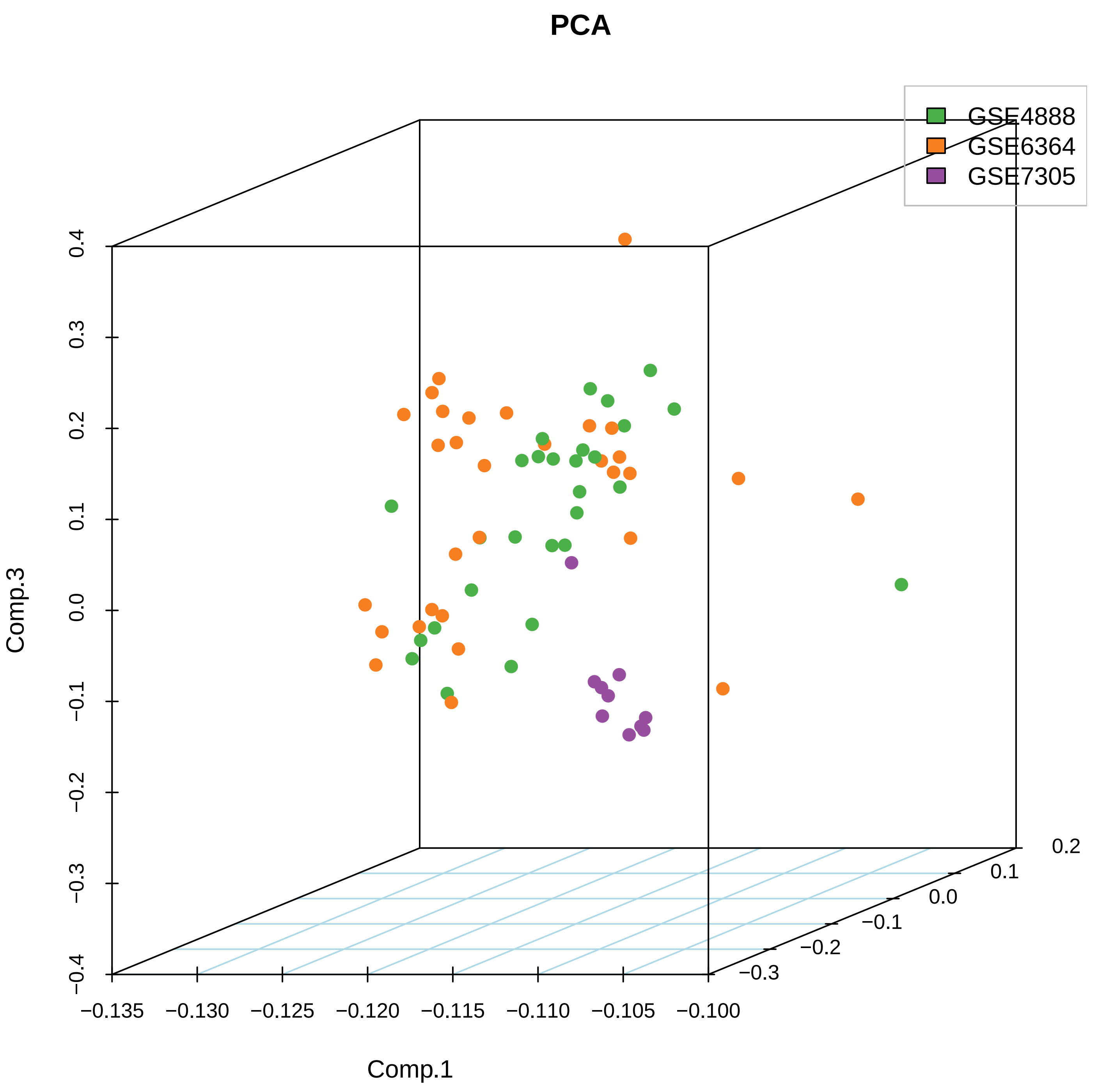
PCA
GSE4888
GSE6364
GSE7305
Comp.1
Comp.2
Comp.3
−0.135
−0.130
−0.125
−0.120
−0.115
−0.110
−0.105
−0.100
−0.3
−0.2
−0.1
0.0
0.1
0.2
−0.4
−0.3
−0.2
−0.1
0.0
0.1
0.2
0.3
0.4

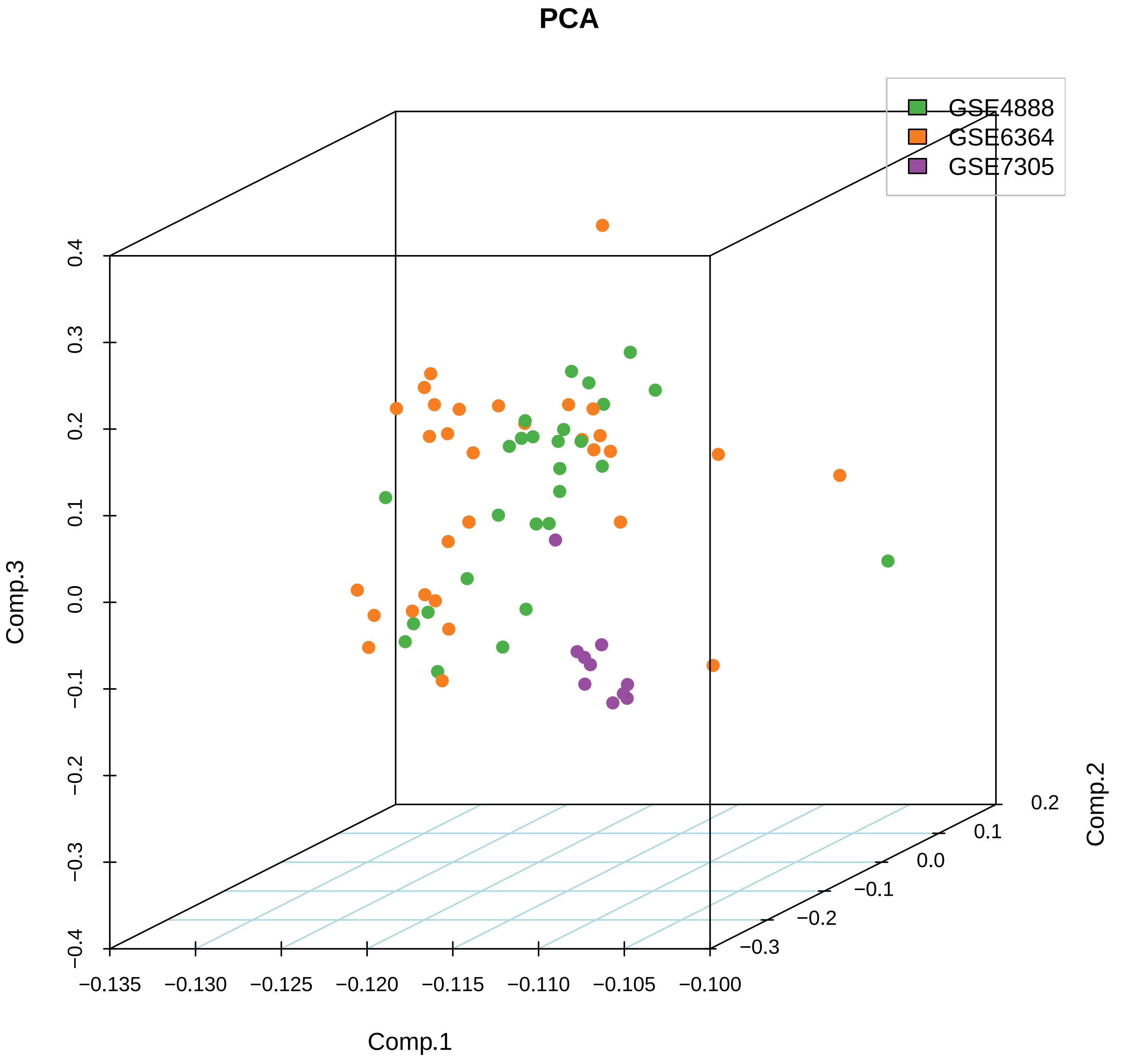

PCA
GSE4888
GSE6364
GSE7305
Comp.1
Comp.2
Comp.3
−0.135
−0.130
−0.125
−0.120
−0.115
−0.110
−0.105
−0.100
−0.3
−0.2
−0.1
0.0
0.1
0.2
−0.4
−0.3
−0.2
−0.1
0.0
0.1
0.2
0.3
0.4

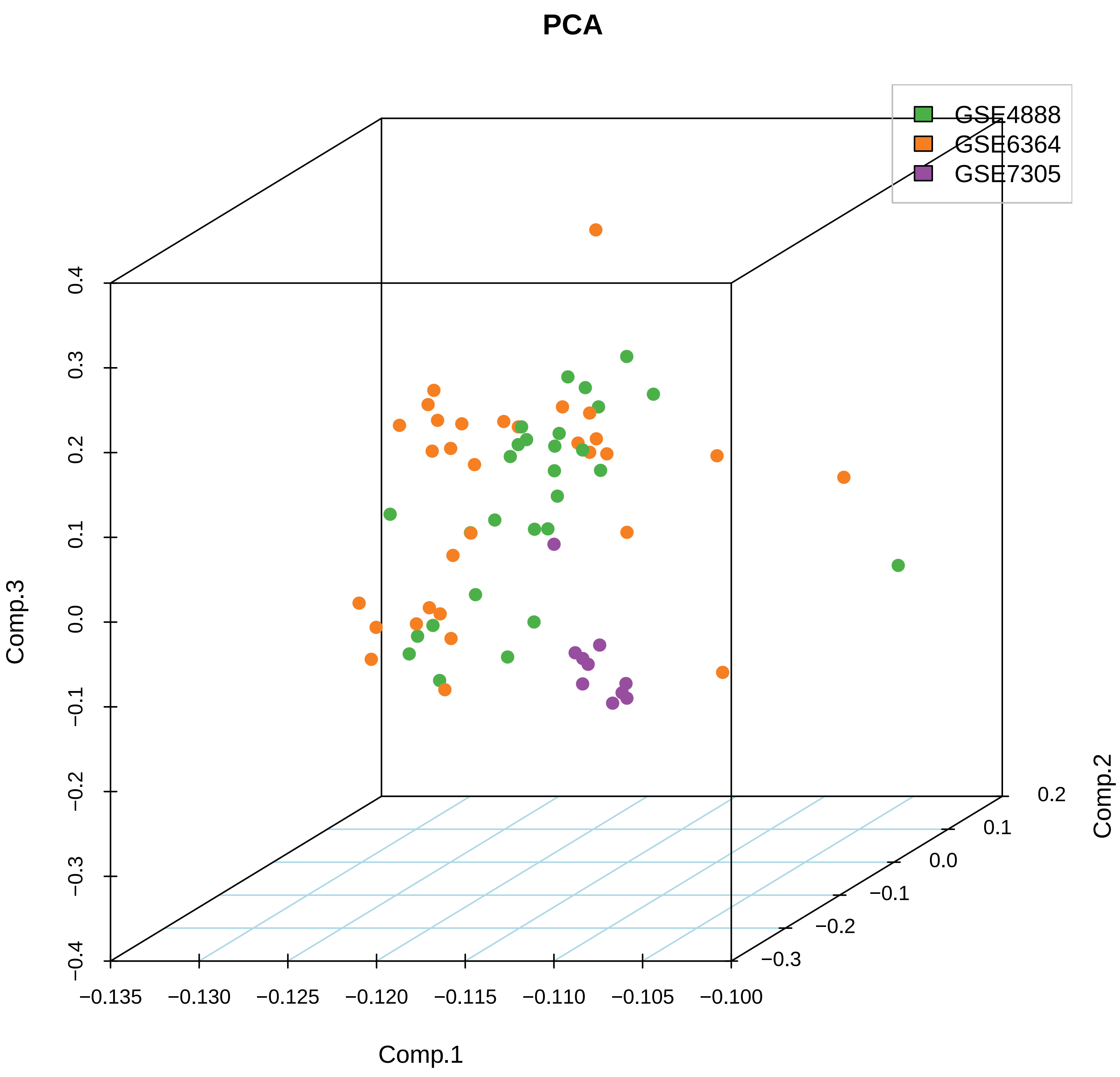
PCA
GSE4888
GSE6364
GSE7305
Comp.1
Comp.2
Comp.3
−0.135
−0.130
−0.125
−0.120
−0.115
−0.110
−0.105
−0.100
0.2
0.1
0.0
−0.1
−0.2
−0.3
0.4
0.3
−0.4

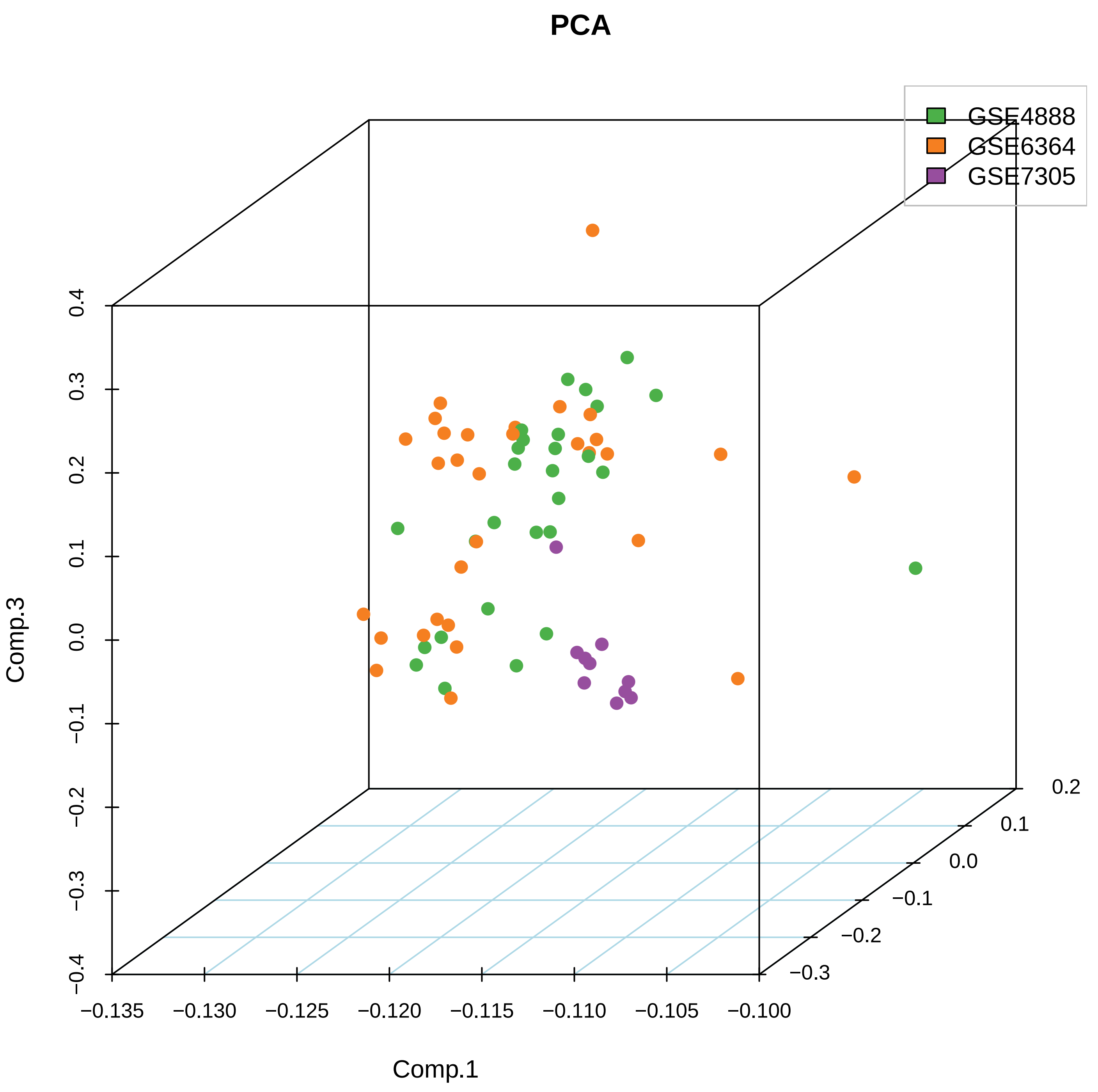
PCA
GSE4888
GSE6364
GSE7305
Comp.3
0.4
0.3
0.2
0.1
0.0
−0.1
−0.2
−0.3
−0.4
Comp.2
0.2
0.1
0.0
−0.1
−0.2
−0.3
−0.135
−0.130
−0.125
−0.120
−0.115
−0.110
−0.105
−0.100
Comp.1

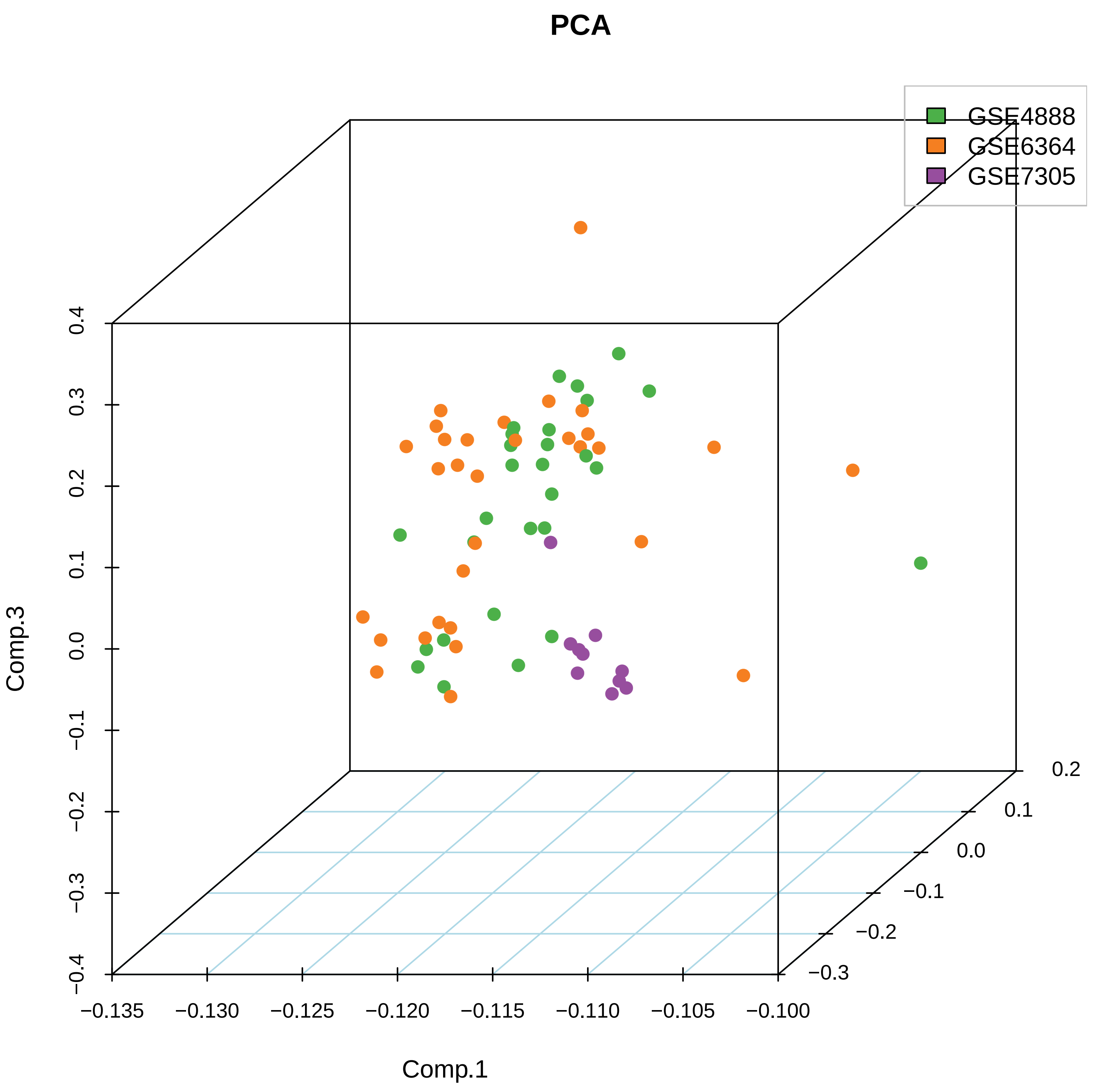
PCA
GSE4888
GSE6364
GSE7305
Comp.1
Comp.2
Comp.3
−0.135
−0.130
−0.125
−0.120
−0.115
−0.110
−0.105
−0.100
−0.3
−0.2
−0.1
0.0
0.1
0.2
−0.4
−0.3
−0.2
−0.1
0.0
0.1
0.2
0.3
0.4

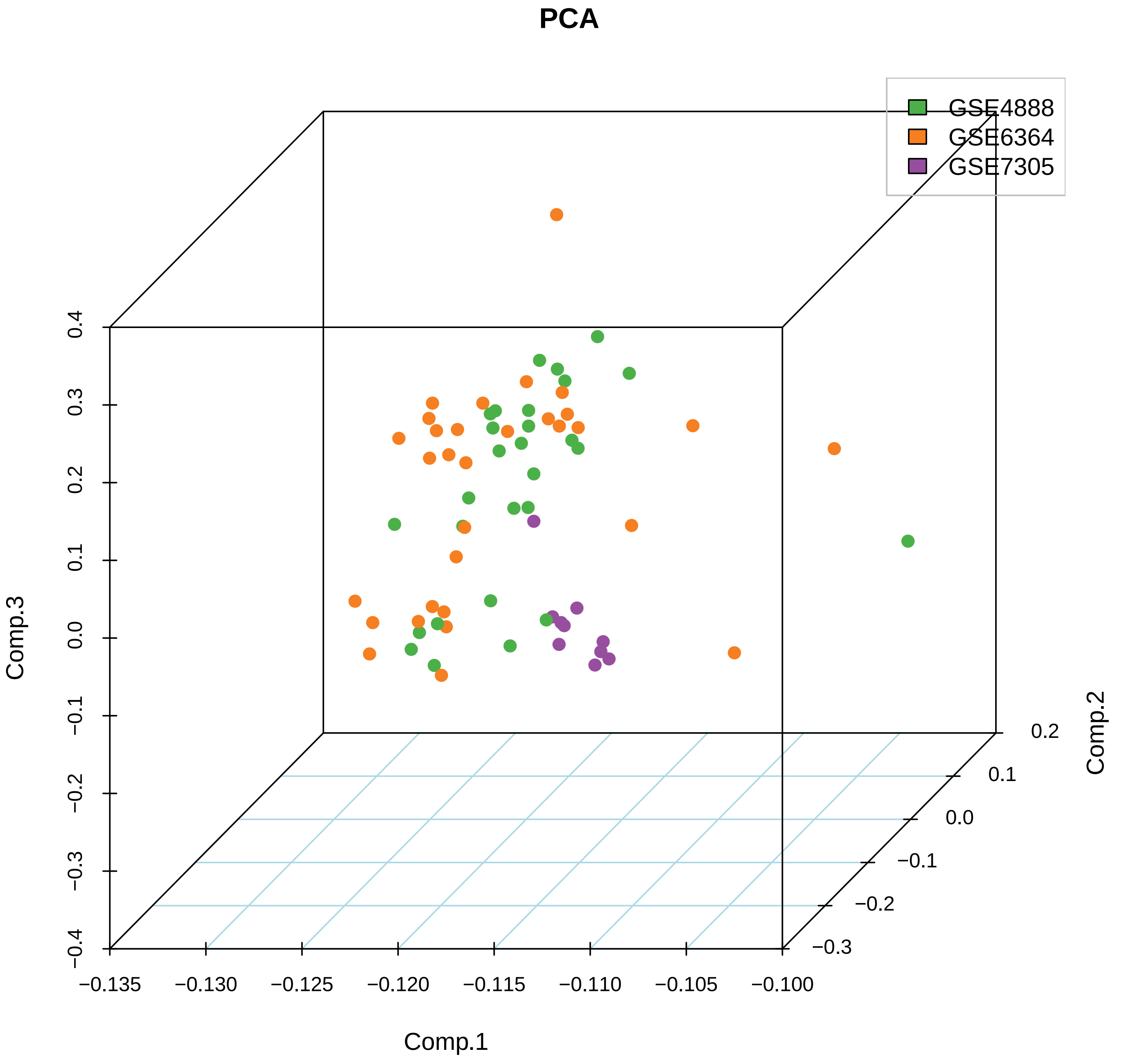

PCA
GSE4888
GSE6364
GSE7305
Comp.3
0.4
0.3
0.2
0.1
0.0
−0.1
−0.2
−0.3
−0.4
Comp.2
0.2
0.1
0.0
−0.1
−0.2
−0.3
Comp.1
−0.135
−0.130
−0.125
−0.120
−0.115
−0.110
−0.105
−0.100

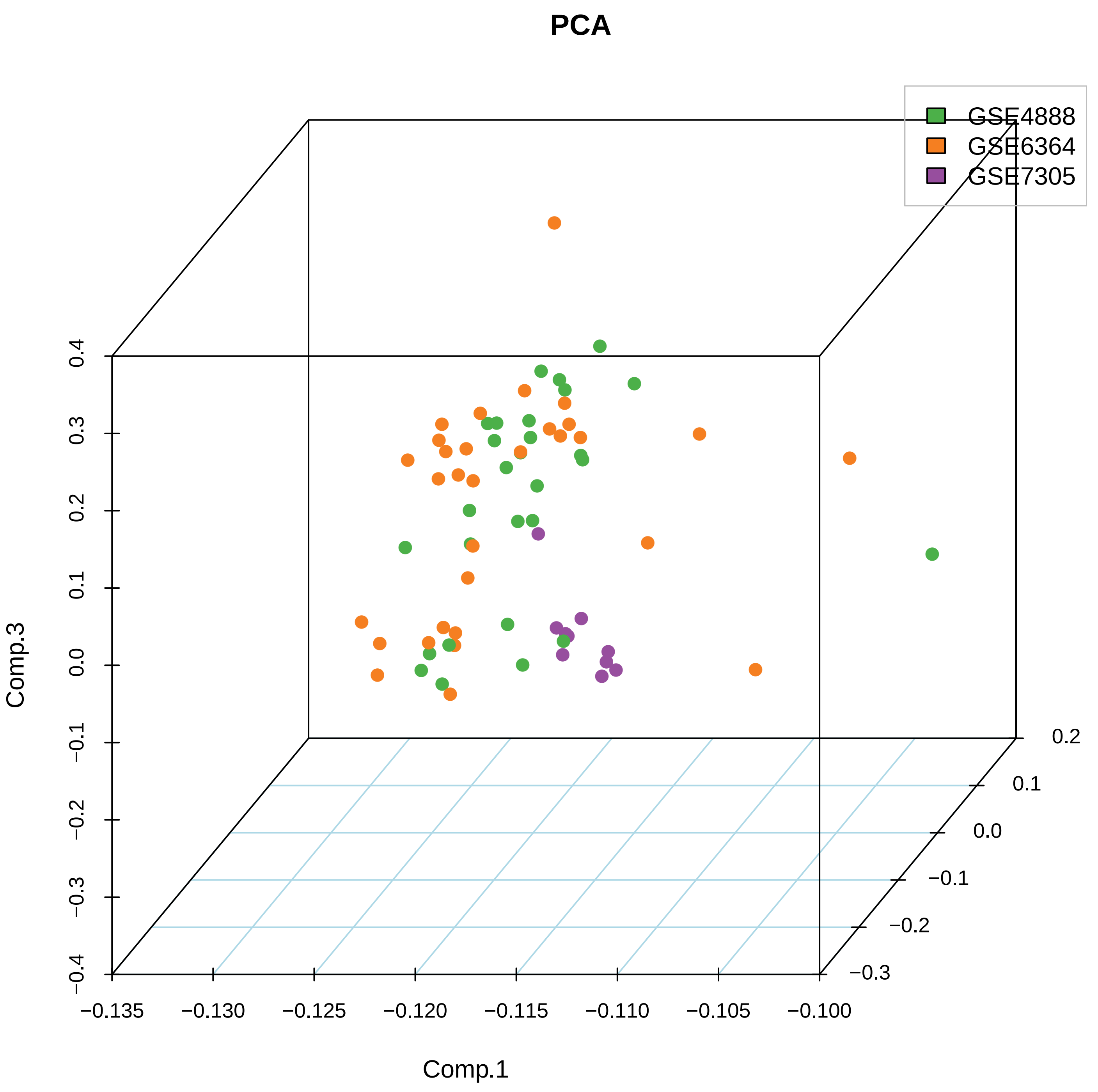
PCA
GSE4888
GSE6364
GSE7305
Comp.3
Comp.2
Comp.1
0.4
0.3
0.2
0.1
0.0
−0.1
−0.2
−0.3
−0.4
−0.135
−0.130
−0.125
−0.120
−0.115
−0.110
−0.105
−0.100
0.2
0.1
0.0
−0.1
−0.2
−0.3

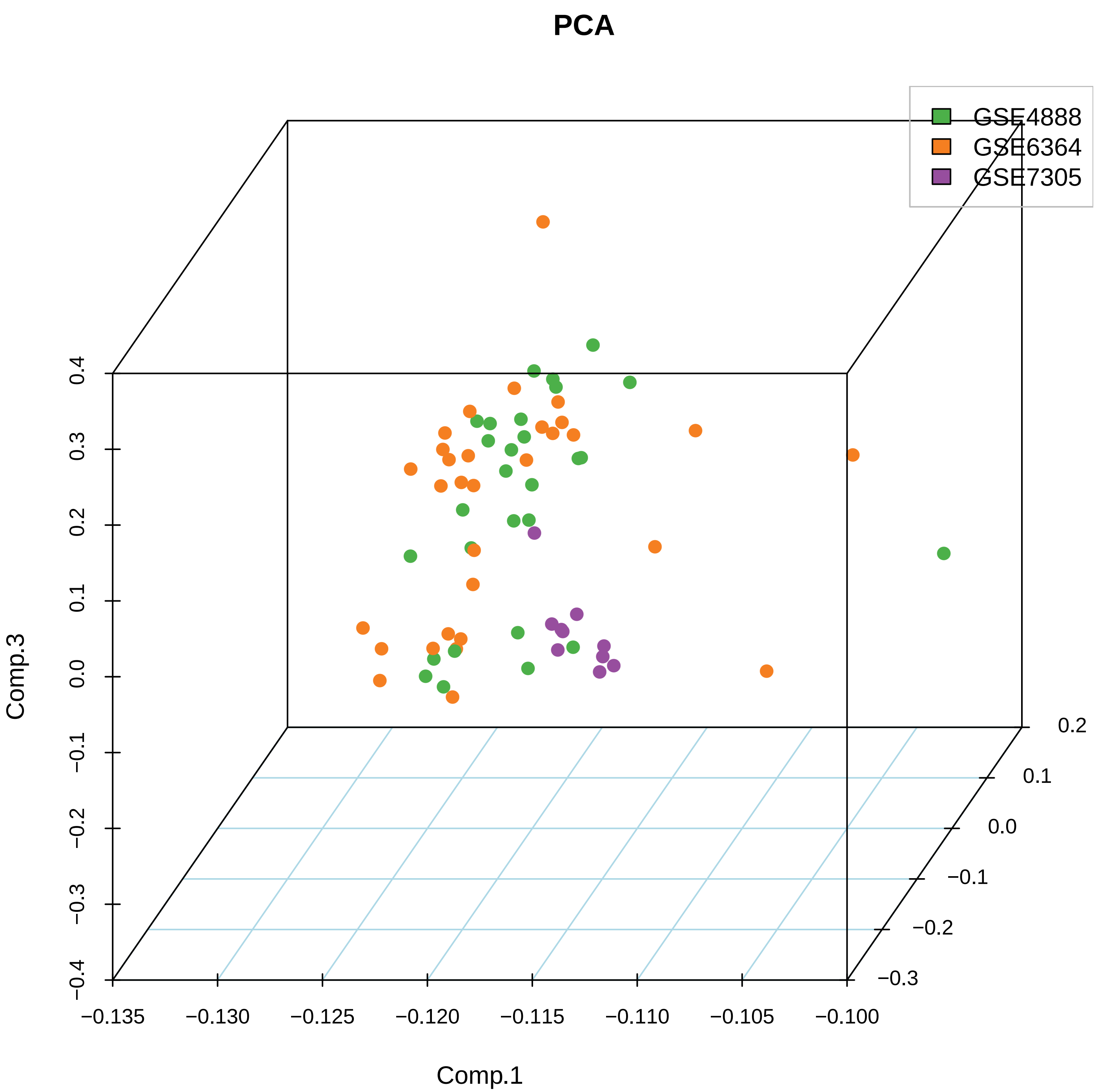

PCA
GSE4888
GSE6364
GSE7305
Comp.1
Comp.2
Comp.3
−0.135
−0.130
−0.125
−0.120
−0.115
−0.110
−0.105
−0.100
−0.3
−0.2
−0.1
0.0
0.1
0.2
−0.4
−0.3
−0.2
−0.1
0.0
0.1
0.2
0.3
0.4

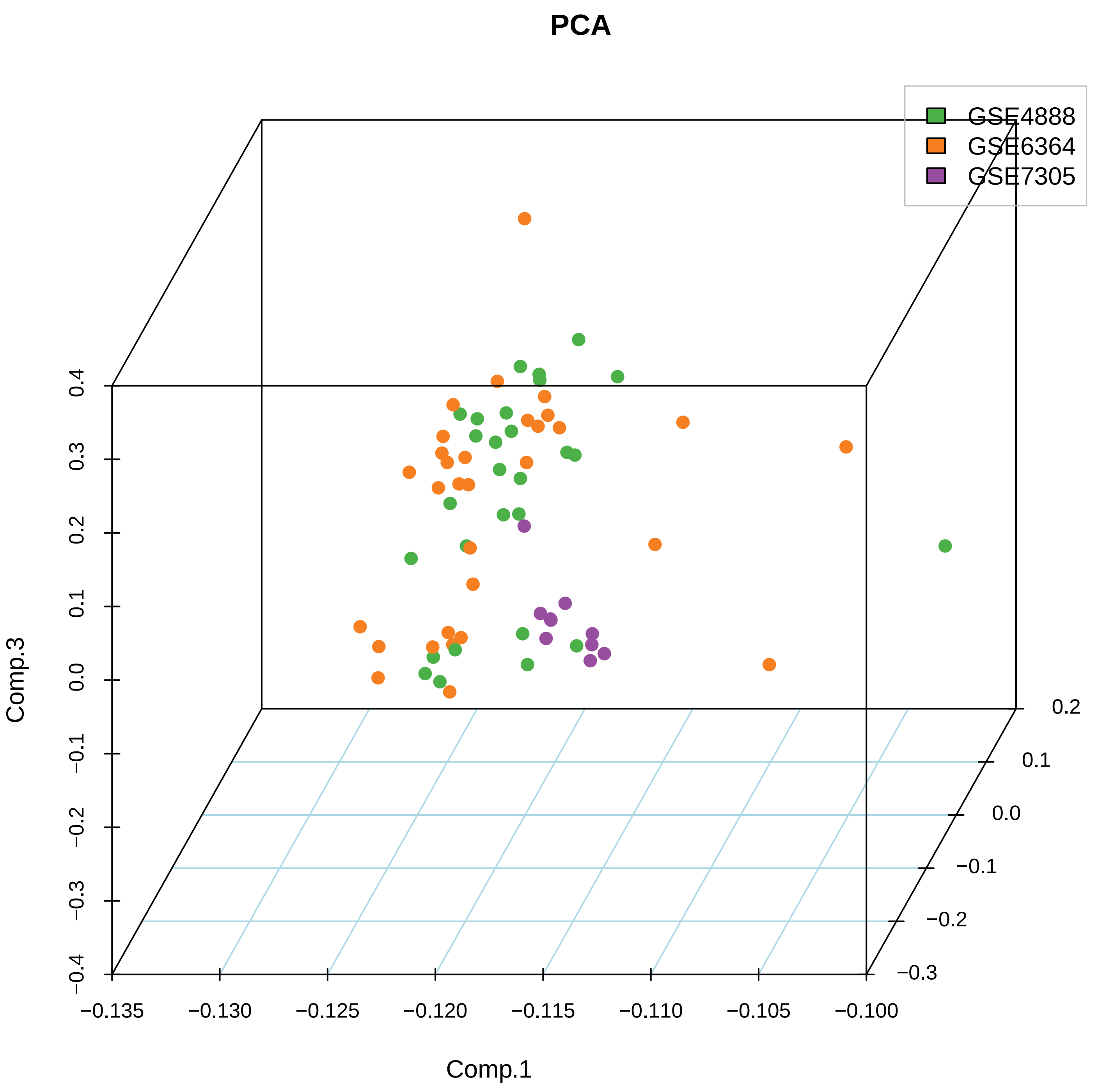
PCA
GSE4888
GSE6364
GSE7305
Comp.1
Comp.2
Comp.3
−0.135
−0.130
−0.125
−0.120
−0.115
−0.110
−0.105
−0.100
−0.3
−0.2
−0.1
0.0
0.1
0.2
−0.4
−0.3
−0.2
−0.1
0.0
0.1
0.2
0.3
0.4

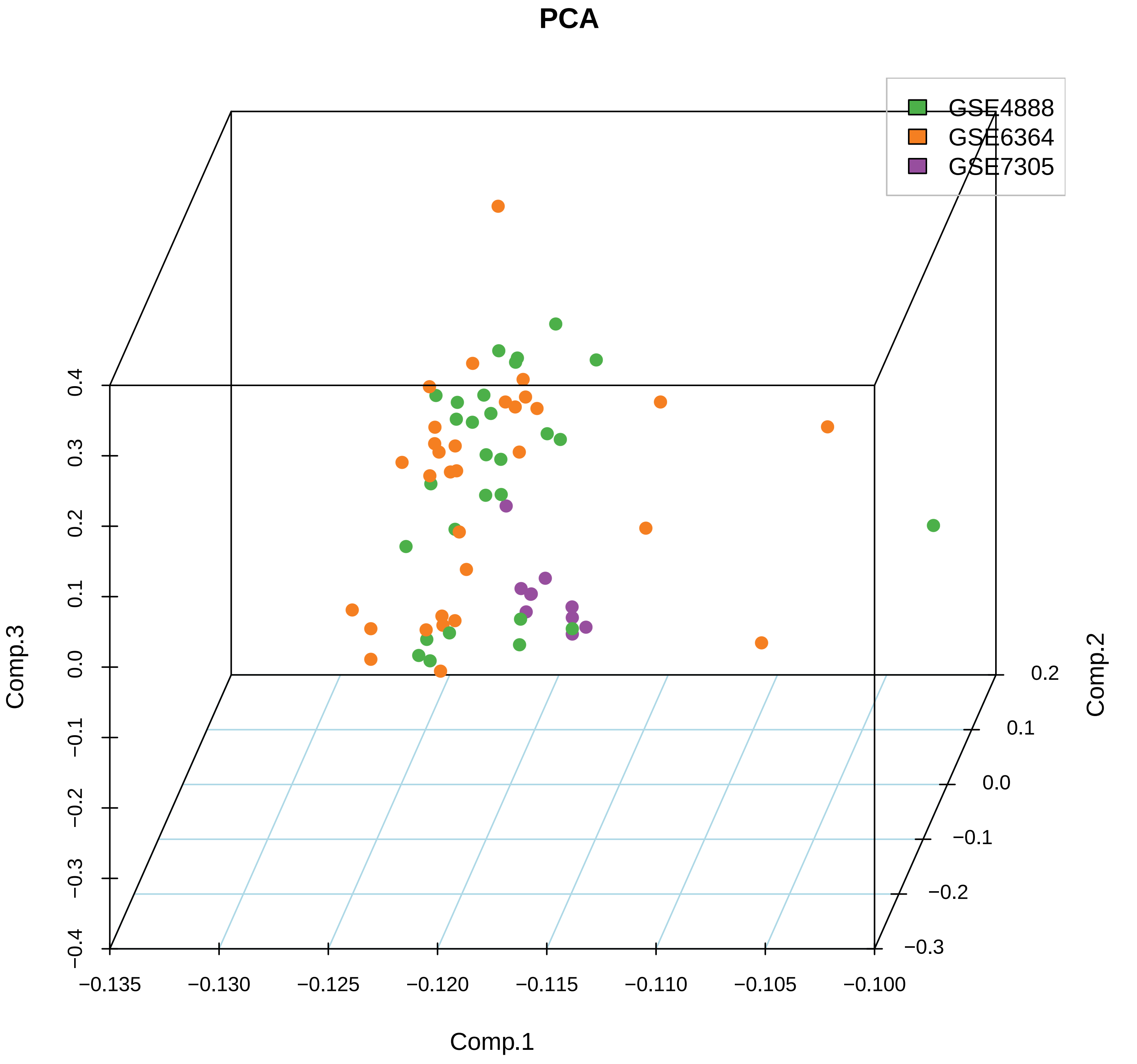

PCA
GSE4888
GSE6364
GSE7305
Comp.1
Comp.2
Comp.3
−0.135
−0.130
−0.125
−0.120
−0.115
−0.110
−0.105
−0.100
−0.3
−0.2
−0.1
0.0
0.1
0.2
−0.4
−0.3
−0.2
−0.1
0.0
0.1
0.2
0.3
0.4

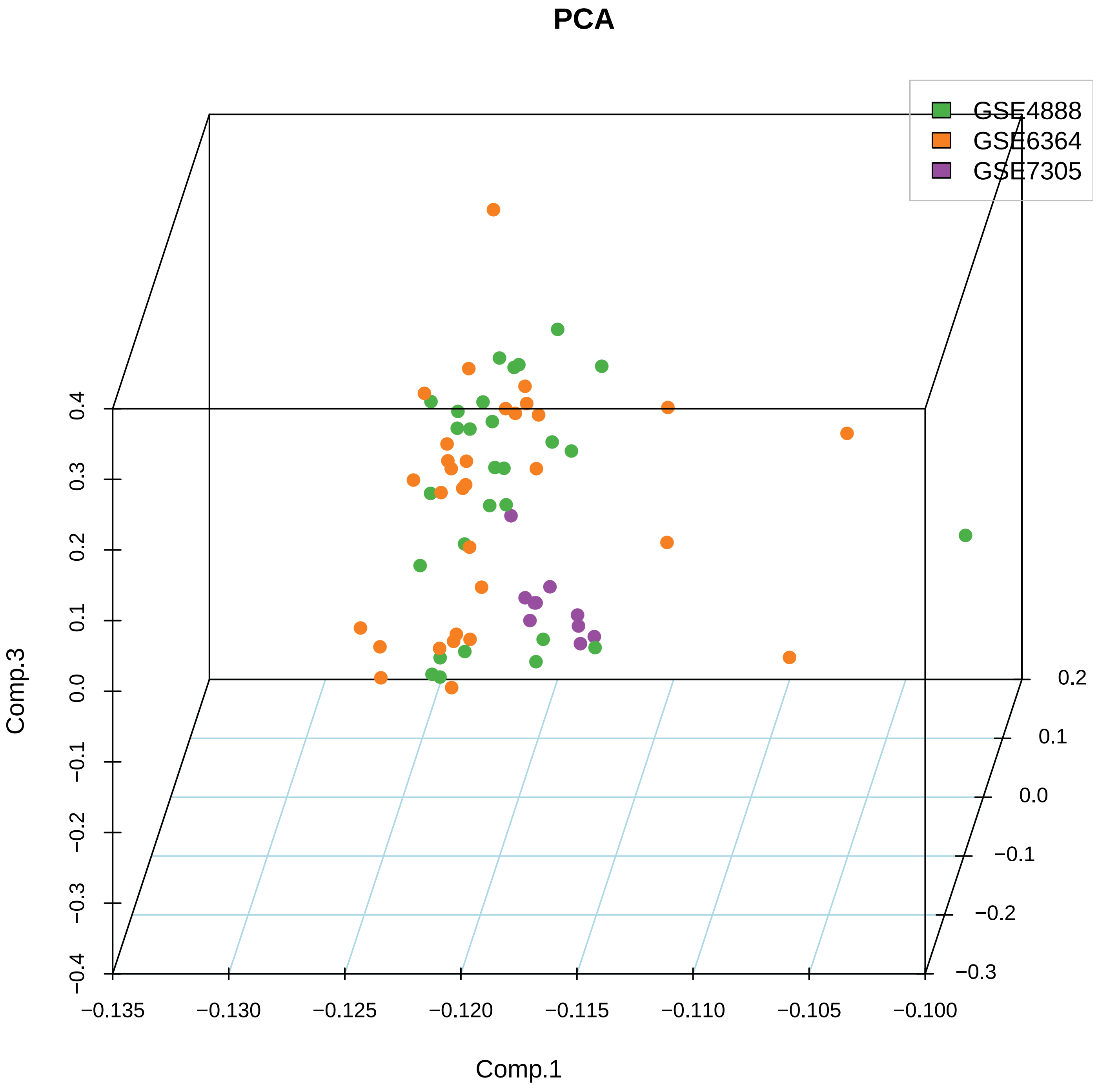

PCA
GSE4888
GSE6364
GSE7305
Comp.3
Comp.2
Comp.1
0.4
0.3
0.2
0.1
0.0
−0.1
−0.2
−0.3
−0.4
−0.135
−0.130
−0.125
−0.120
−0.115
−0.110
−0.105
−0.100
0.2
0.1
0.0
−0.1
−0.2
−0.3

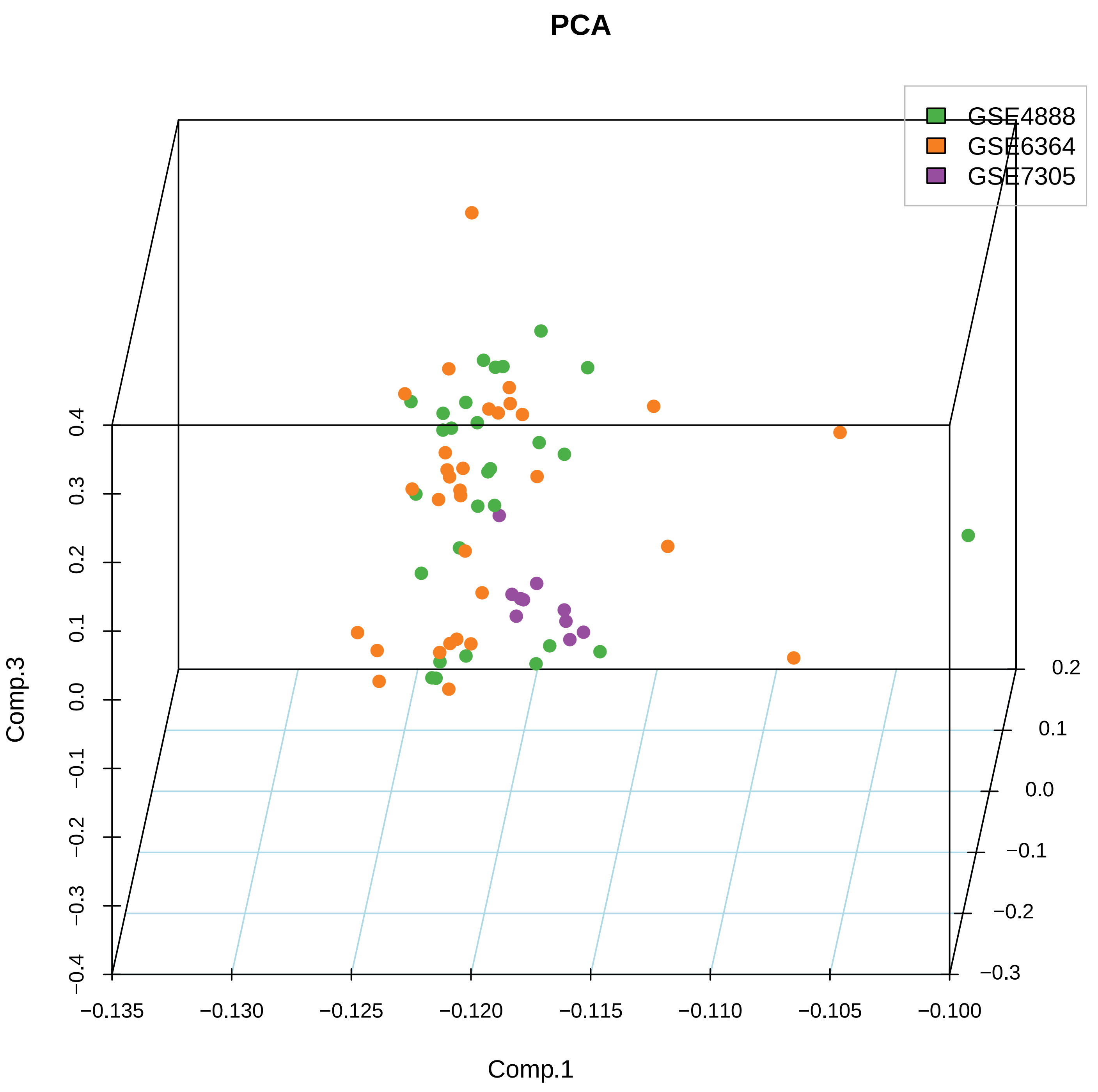

PCA
GSE4888
GSE6364
GSE7305
Comp.1
Comp.2
Comp.3
−0.135
−0.130
−0.125
−0.120
−0.115
−0.110
−0.105
−0.100
−0.3
−0.2
−0.1
0.0
0.1
0.2
−0.4
−0.3
−0.2
−0.1
0.0
0.1
0.2
0.3
0.4

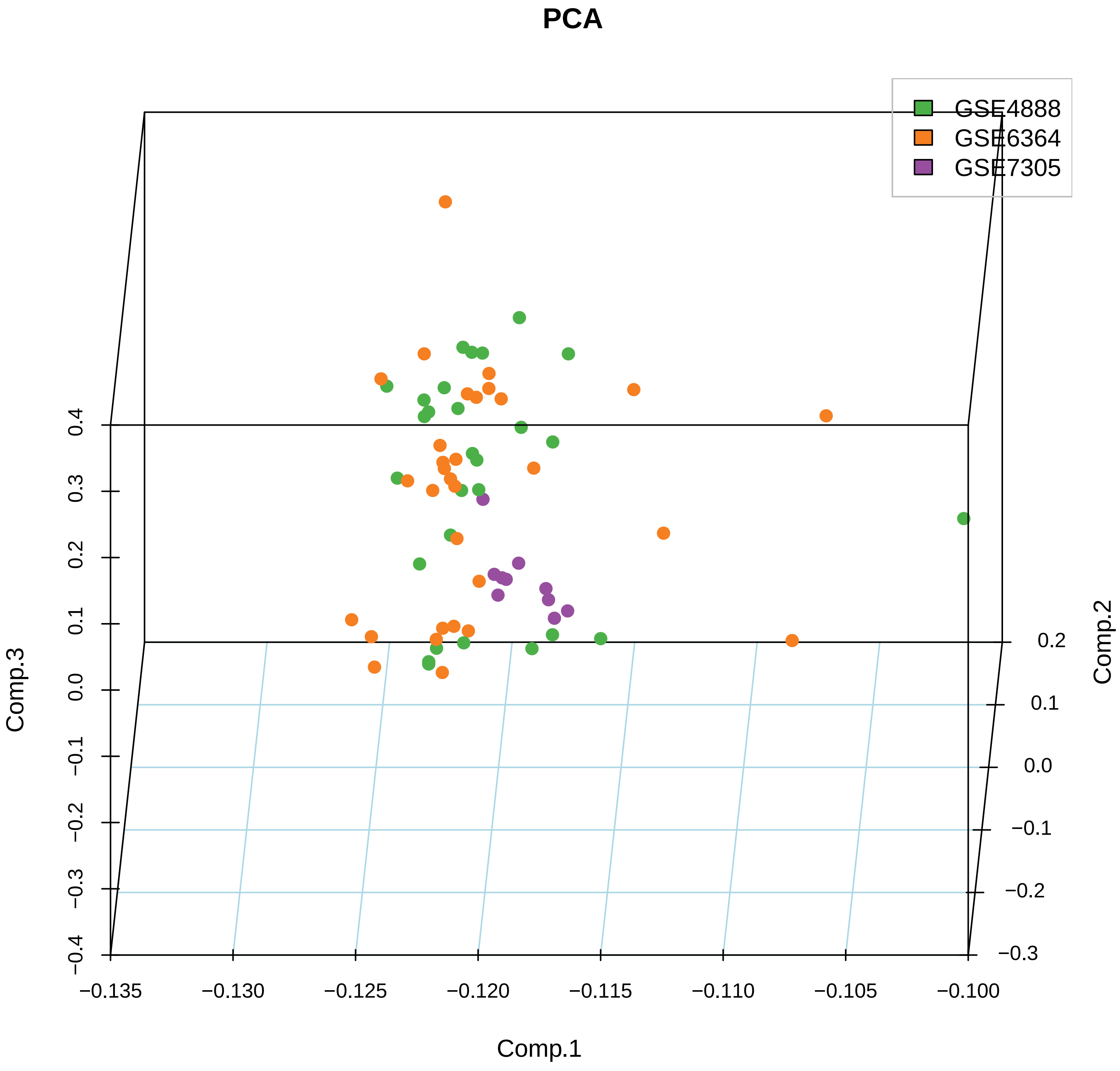

PCA
GSE4888
GSE6364
GSE7305
Comp.1
Comp.2
Comp.3
−0.135
−0.130
−0.125
−0.120
−0.115
−0.110
−0.105
−0.100
−0.4
−0.3
−0.2
−0.1
0.0
0.1
0.2
0.3
0.4
−0.3
−0.2
−0.1
0.0
0.1
0.2

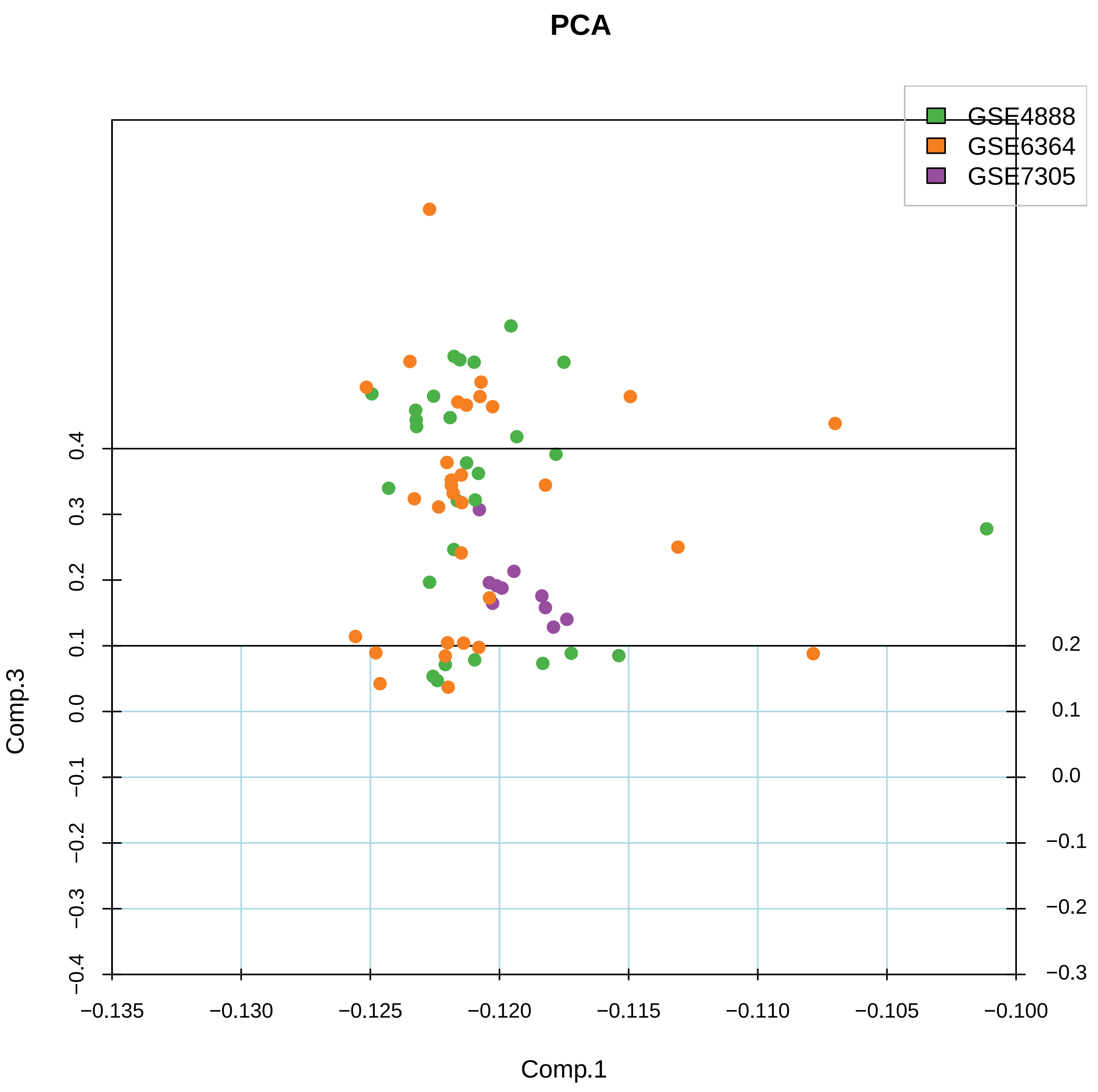

PCA
GSE4888
GSE6364
GSE7305
Comp.1
Comp.2
Comp.3
−0.135
−0.130
−0.125
−0.120
−0.115
−0.110
−0.105
−0.100
−0.4
−0.3
−0.2
−0.1
0.0
0.1
0.2
0.3
0.4
−0.3
−0.2
−0.1
0.0
0.1
0.2

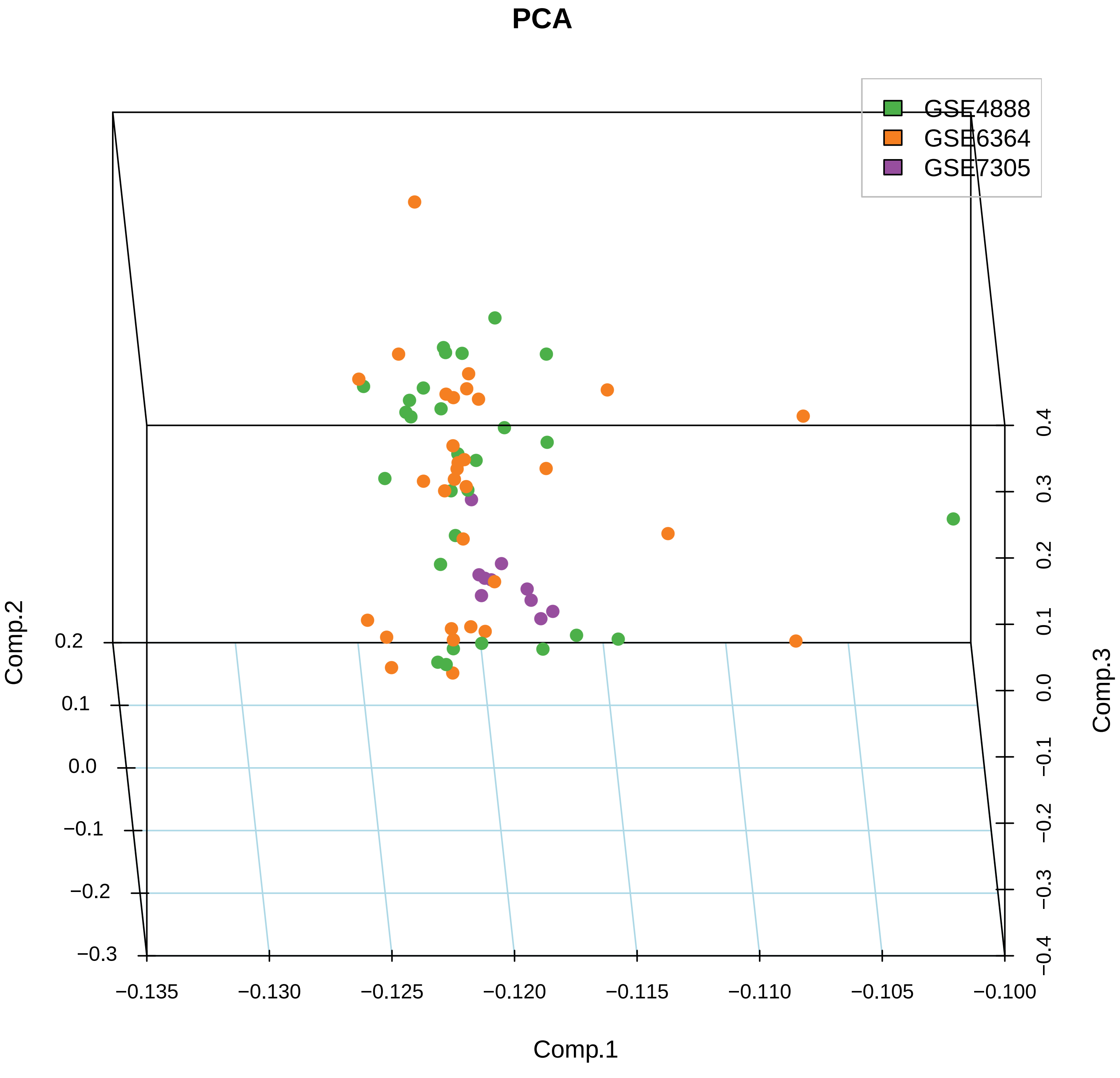

PCA
GSE4888
GSE6364
GSE7305
Comp.1
Comp.2
Comp.3
−0.135
−0.130
−0.125
−0.120
−0.115
−0.110
−0.105
−0.100
0.2
0.1
0.0
−0.1
−0.2
−0.3
0.4
0.3
0.2
0.1
0.0
−0.1
−0.2
−0.3
−0.4

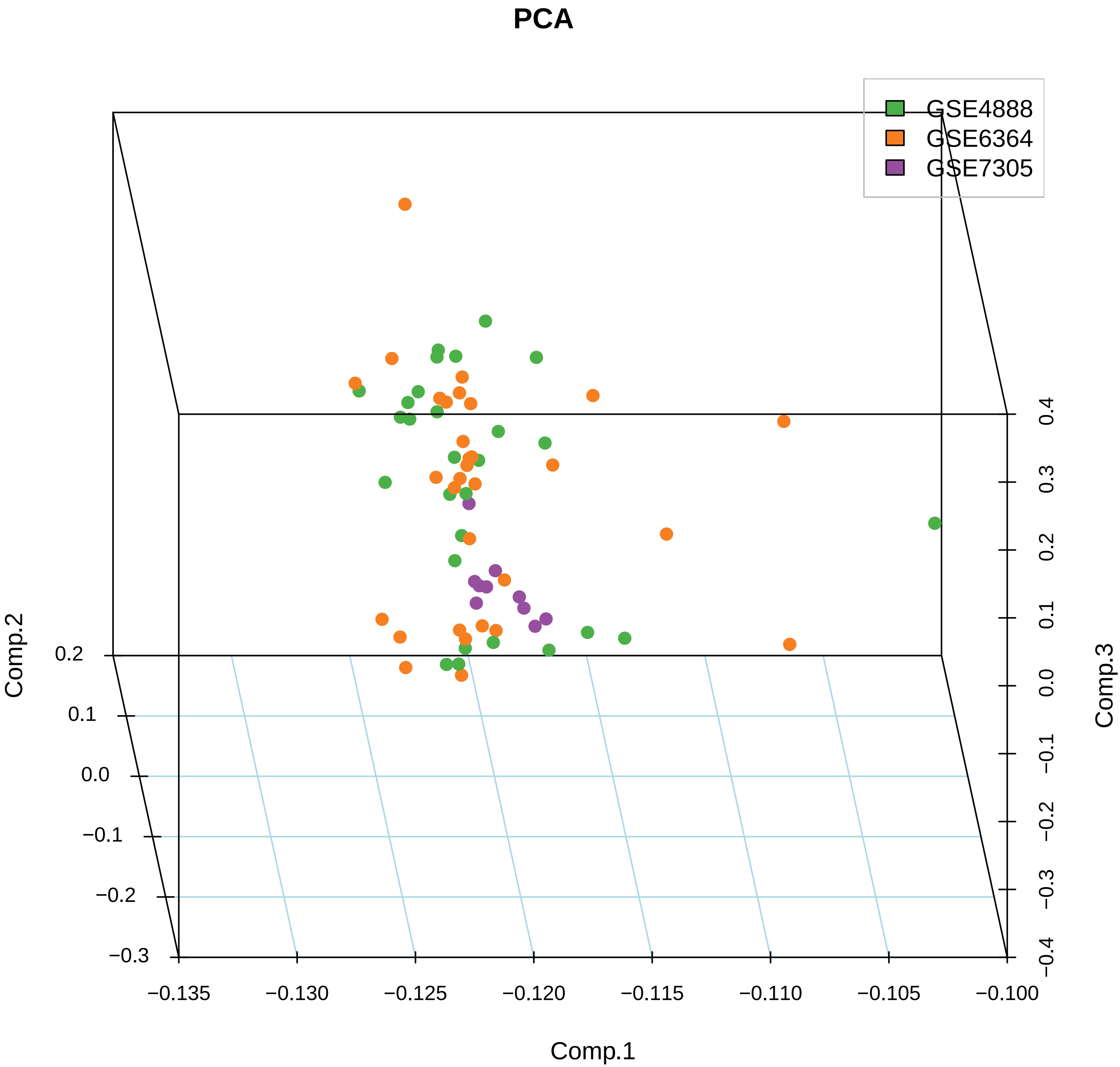

PCA
GSE4888
GSE6364
GSE7305
Comp.1
Comp.2
Comp.3
−0.135
−0.130
−0.125
−0.120
−0.115
−0.110
−0.105
−0.100
0.2
0.1
0.0
−0.1
−0.2
−0.3
0.4
0.3
0.2
0.1
0.0
−0.1
−0.2
−0.3
−0.4

PCA

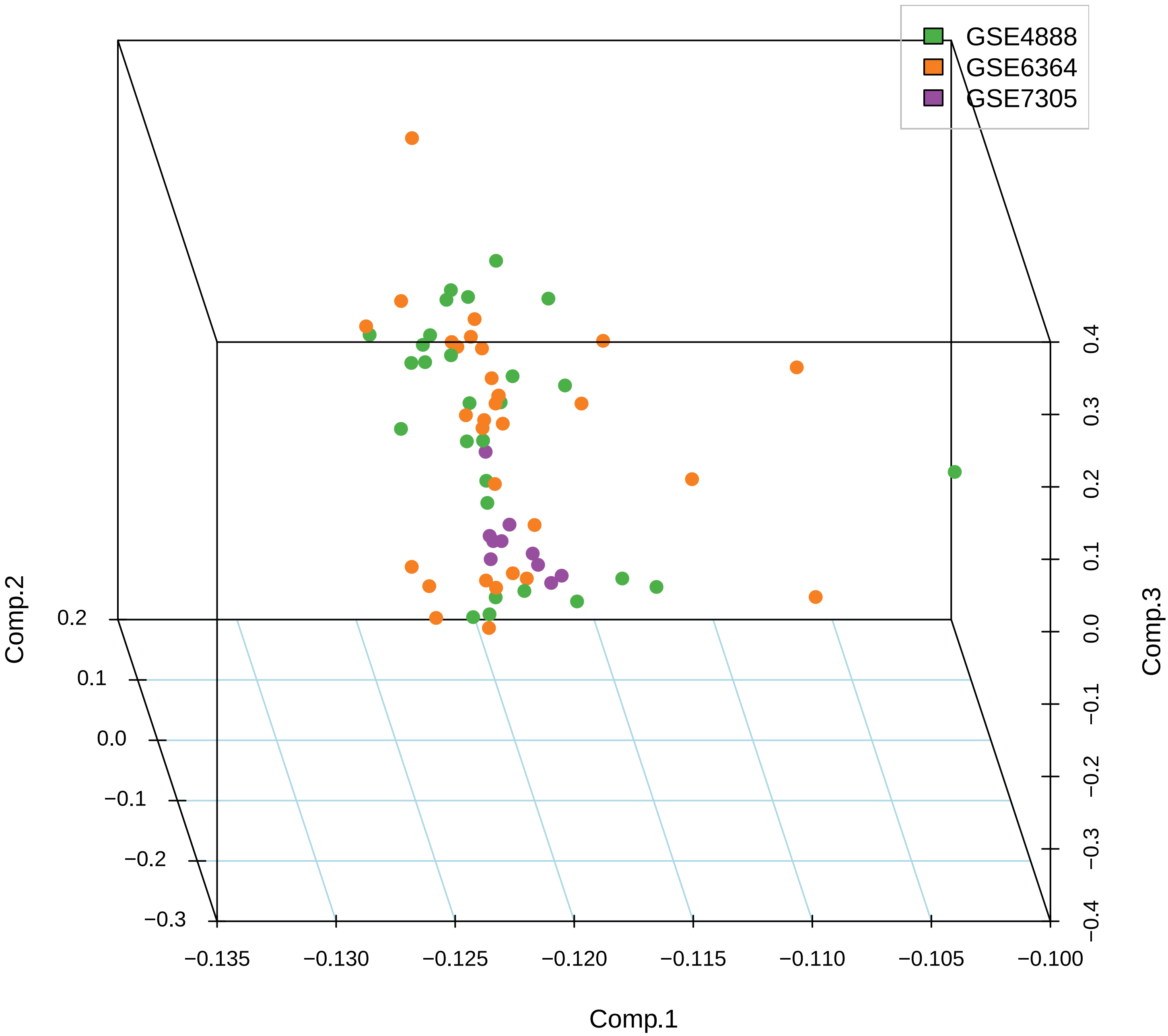

GSE4888
GSE6364
GSE7305
Comp.1
Comp.2
Comp.3
−0.135
−0.130
−0.125
−0.120
−0.115
−0.110
−0.105
−0.100
0.2
0.1
0.0
−0.1
−0.2
−0.3
0.4
0.3
0.2
0.1
0.0
−0.1
−0.2
−0.3
−0.4

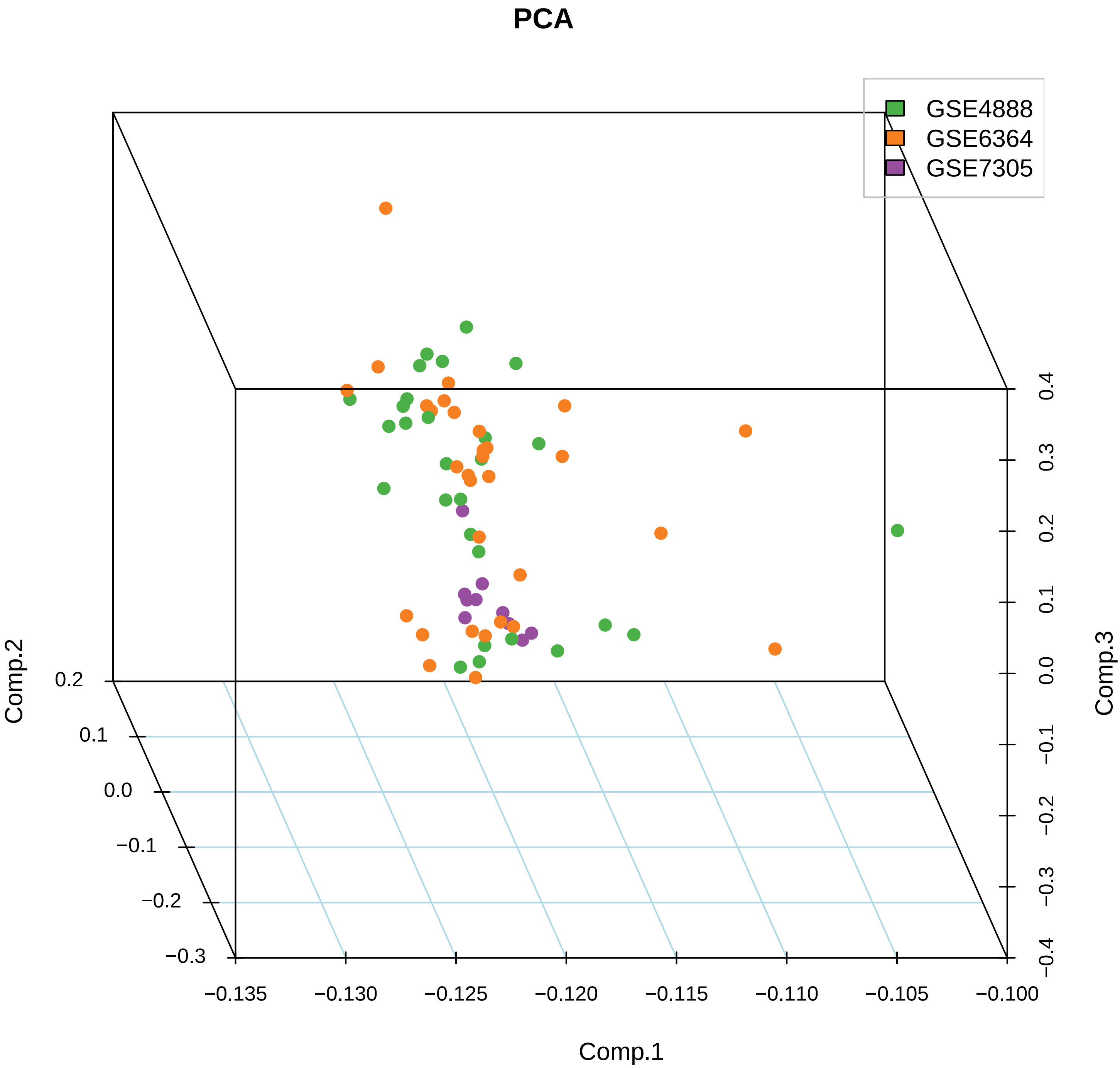
PCA
GSE4888
GSE6364
GSE7305
Comp.1
Comp.2
Comp.3
−0.135
−0.130
−0.125
−0.120
−0.115
−0.110
−0.105
−0.100
0.2
0.1
0.0
−0.1
−0.2
−0.3
0.4
0.3
0.2
0.1
0.0
−0.1
−0.2
−0.3
−0.4

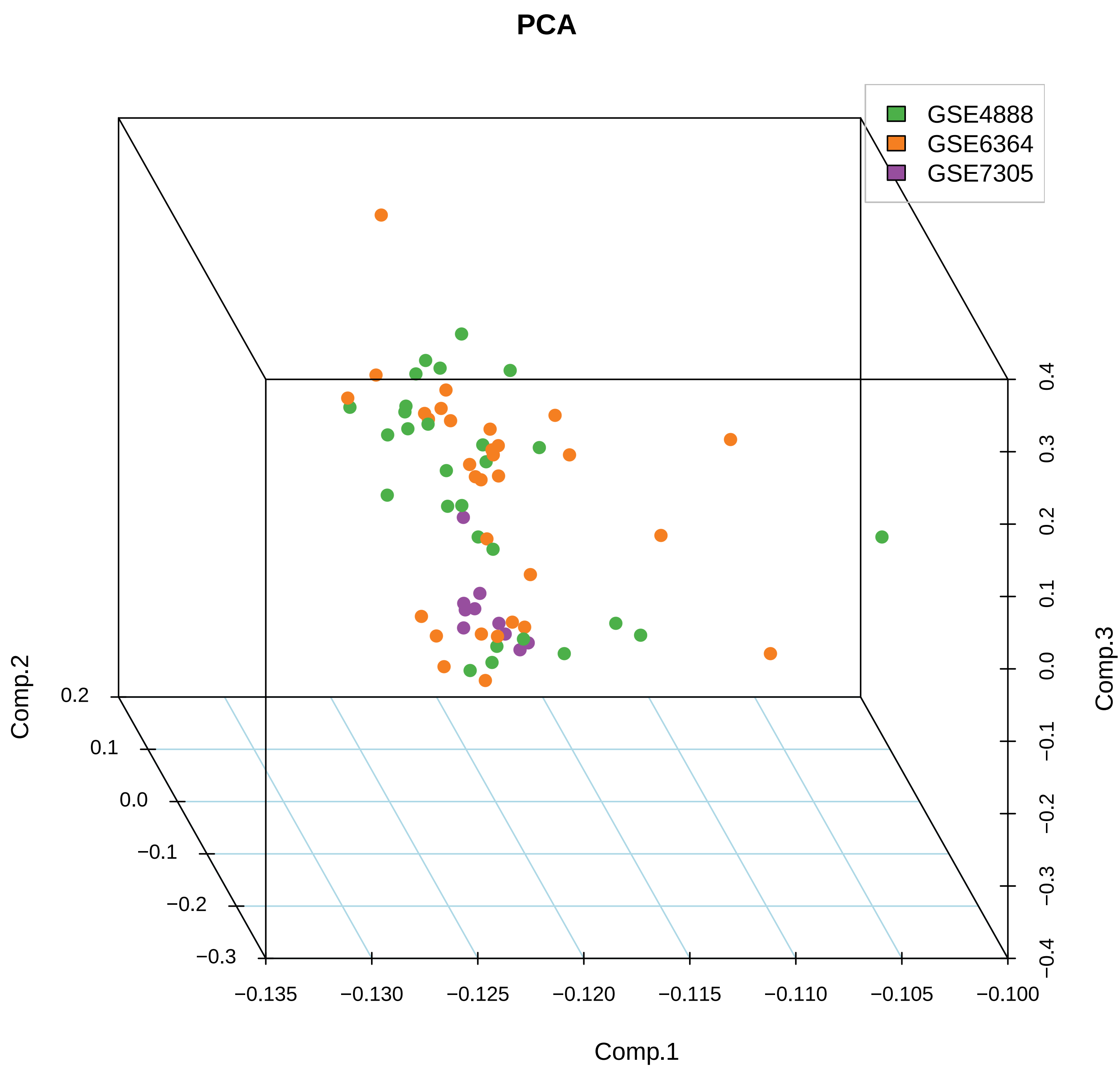

PCA
GSE4888
GSE6364
GSE7305
Comp.1
Comp.2
Comp.3
−0.135
−0.130
−0.125
−0.120
−0.115
−0.110
−0.105
−0.100
0.2
0.1
0.0
−0.1
−0.2
−0.3
0.4
0.3
0.2
0.1
0.0
−0.1
−0.2
−0.3
−0.4

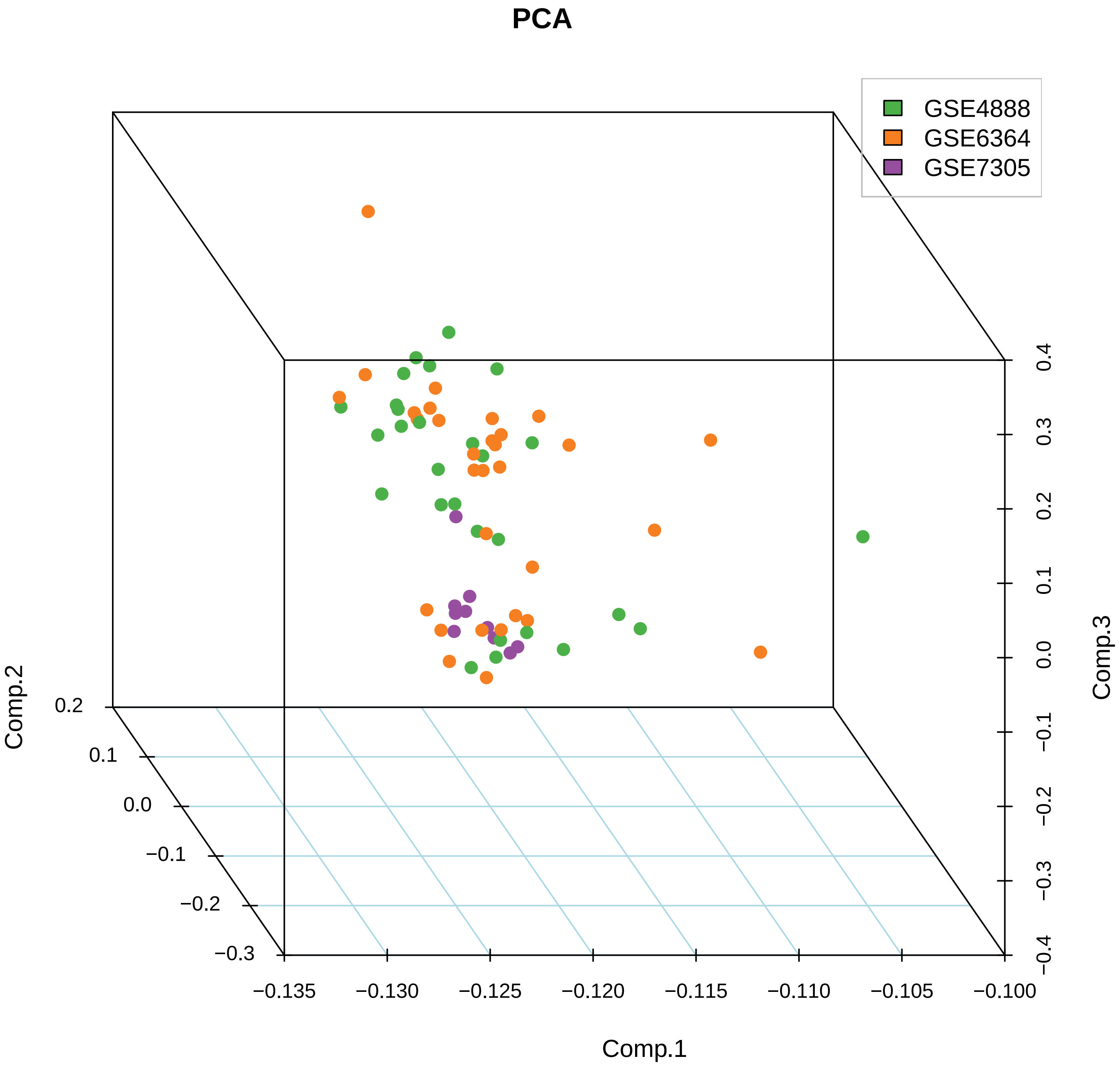

PCA
GSE4888
GSE6364
GSE7305
Comp.1
Comp.2
Comp.3
−0.135
−0.130
−0.125
−0.120
−0.115
−0.110
−0.105
−0.100
0.2
0.1
0.0
−0.1
−0.2
−0.3
0.4
0.3
0.2
0.1
0.0
−0.1
−0.2
−0.3
−0.4

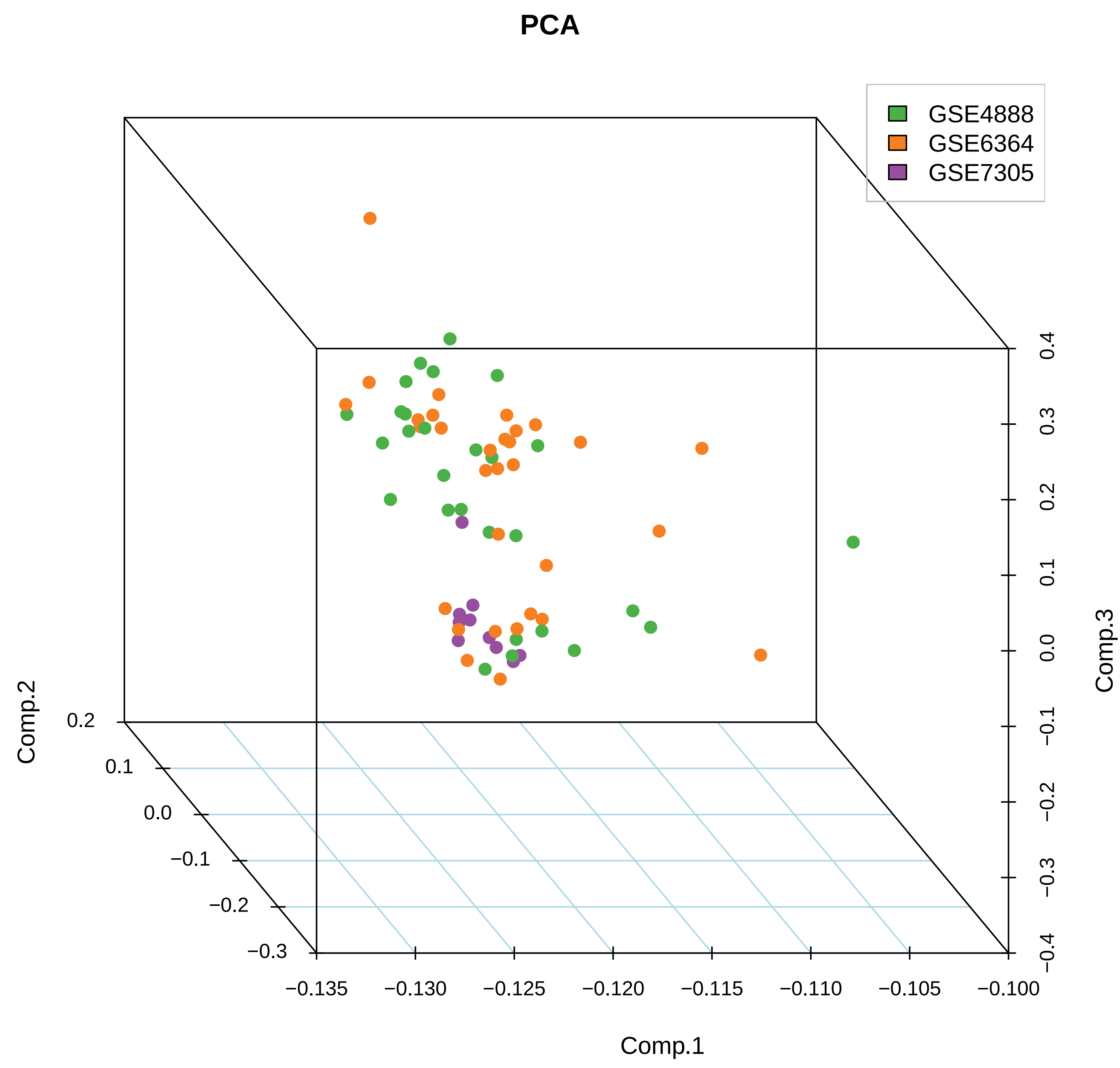
PCA
GSE4888
GSE6364
GSE7305
Comp.1
Comp.2
Comp.3
−0.135
−0.130
−0.125
−0.120
−0.115
−0.110
−0.105
−0.100
0.2
0.1
0.0
−0.1
−0.2
−0.3
0.4
0.3
0.2
0.1
0.0
−0.1
−0.2
−0.3
−0.4

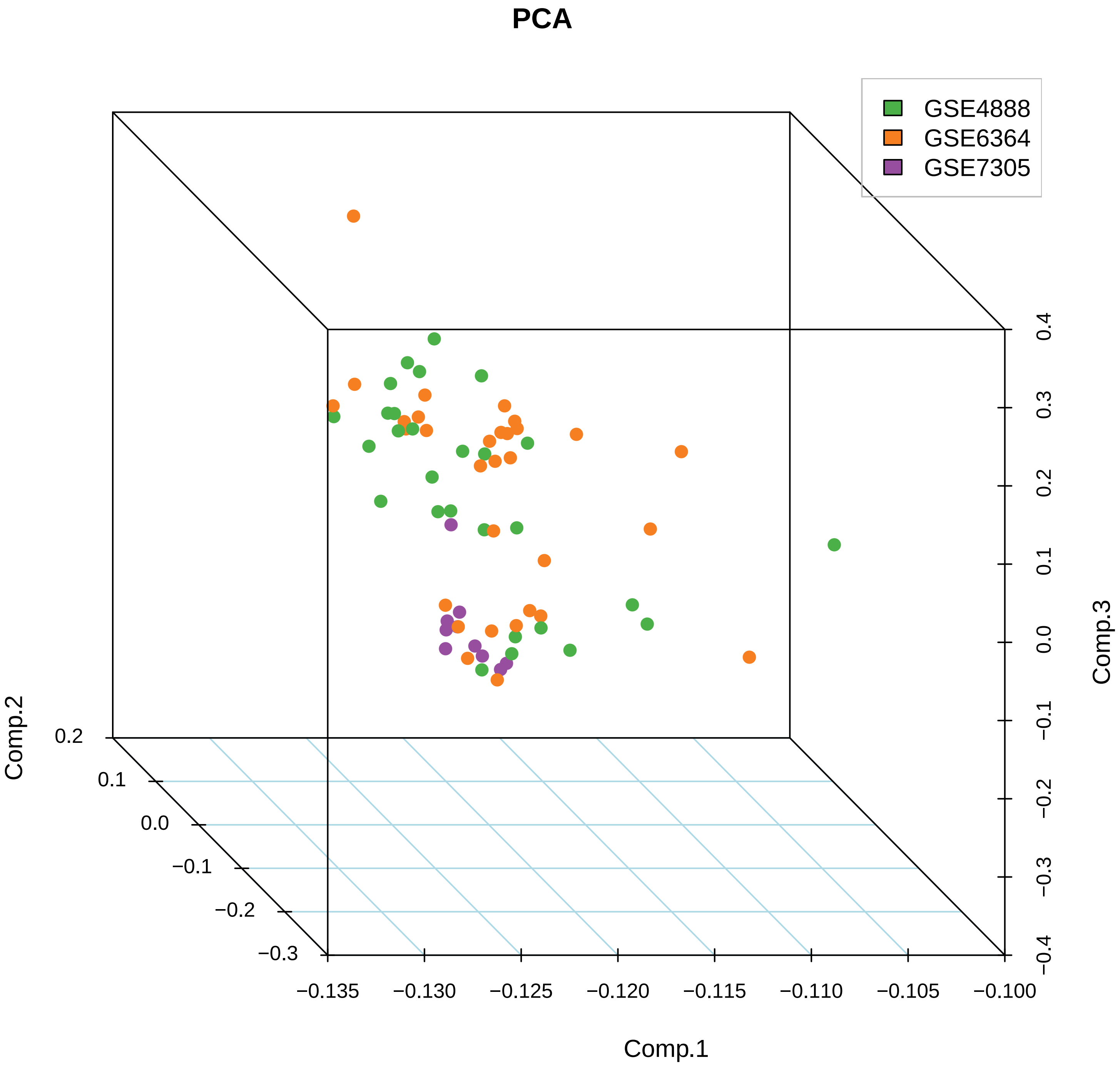

PCA
GSE4888
GSE6364
GSE7305
Comp.1
Comp.2
Comp.3
−0.135
−0.130
−0.125
−0.120
−0.115
−0.110
−0.105
−0.100
0.2
0.1
0.0
−0.1
−0.2
−0.3
0.4
0.3
0.2
0.1
0.0
−0.1
−0.2
−0.3
−0.4

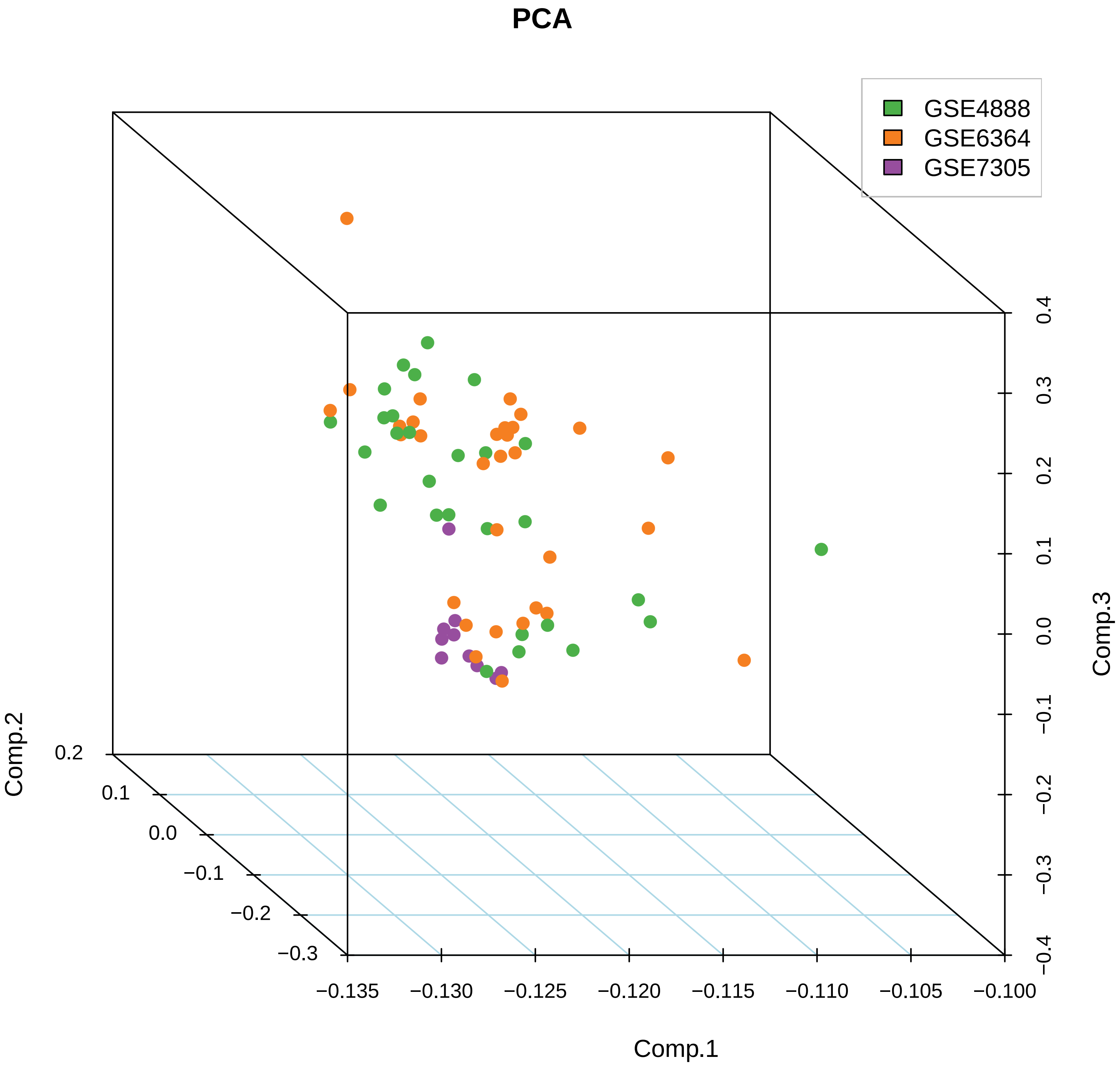

PCA
GSE4888
GSE6364
GSE7305
Comp.1
Comp.2
Comp.3
−0.135
−0.130
−0.125
−0.120
−0.115
−0.110
−0.105
−0.100
0.2
0.1
0.0
−0.1
−0.2
−0.3
0.4
0.3
0.2
0.1
0.0
−0.1
−0.2
−0.3
−0.4

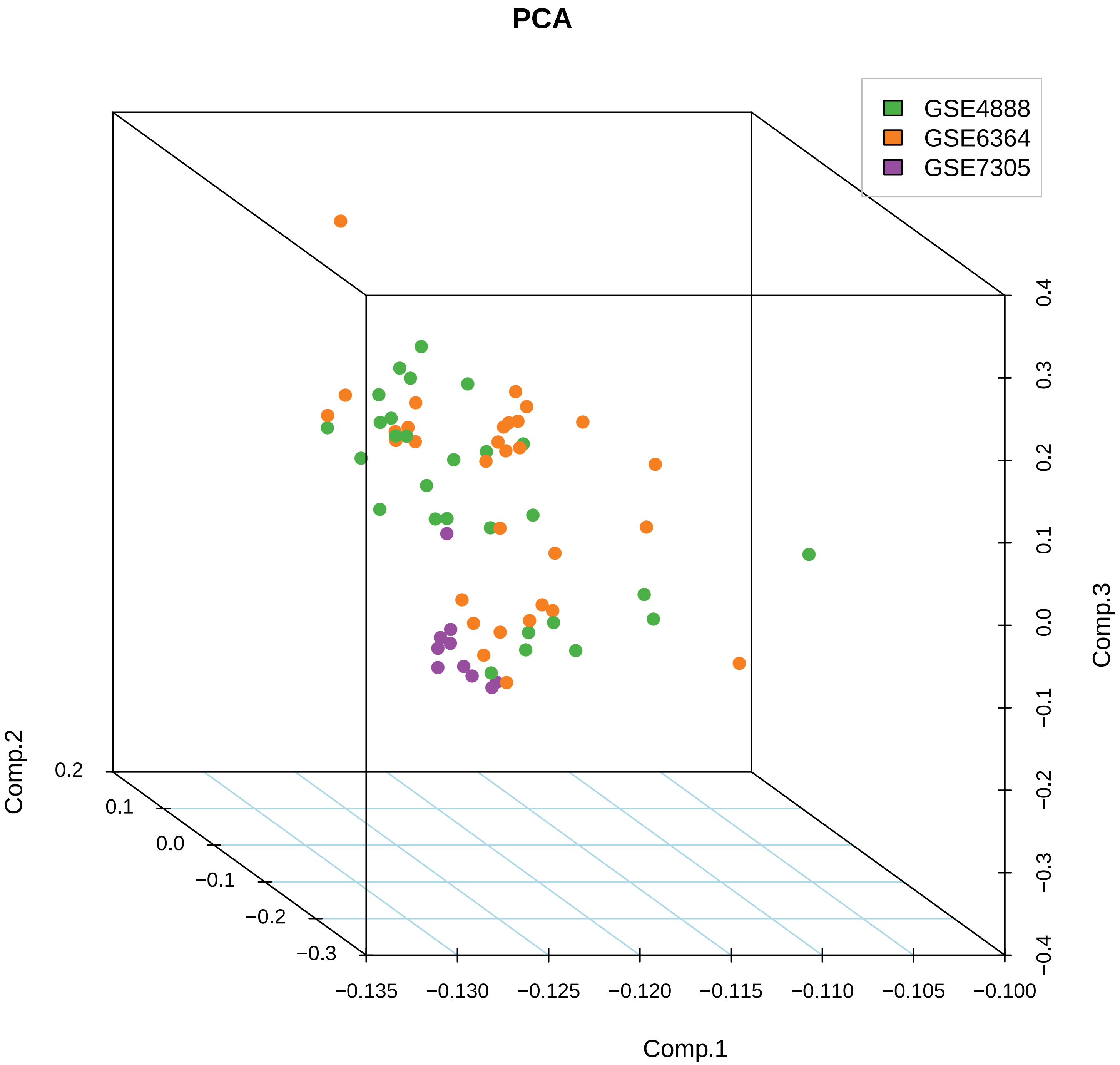
PCA
GSE4888
GSE6364
GSE7305
Comp.1
Comp.2
Comp.3
−0.135
−0.130
−0.125
−0.120
−0.115
−0.110
−0.105
−0.100
0.2
0.1
0.0
−0.1
−0.2
−0.3
0.4
0.3
0.2
0.1
0.0
−0.1
−0.2
−0.3
−0.4

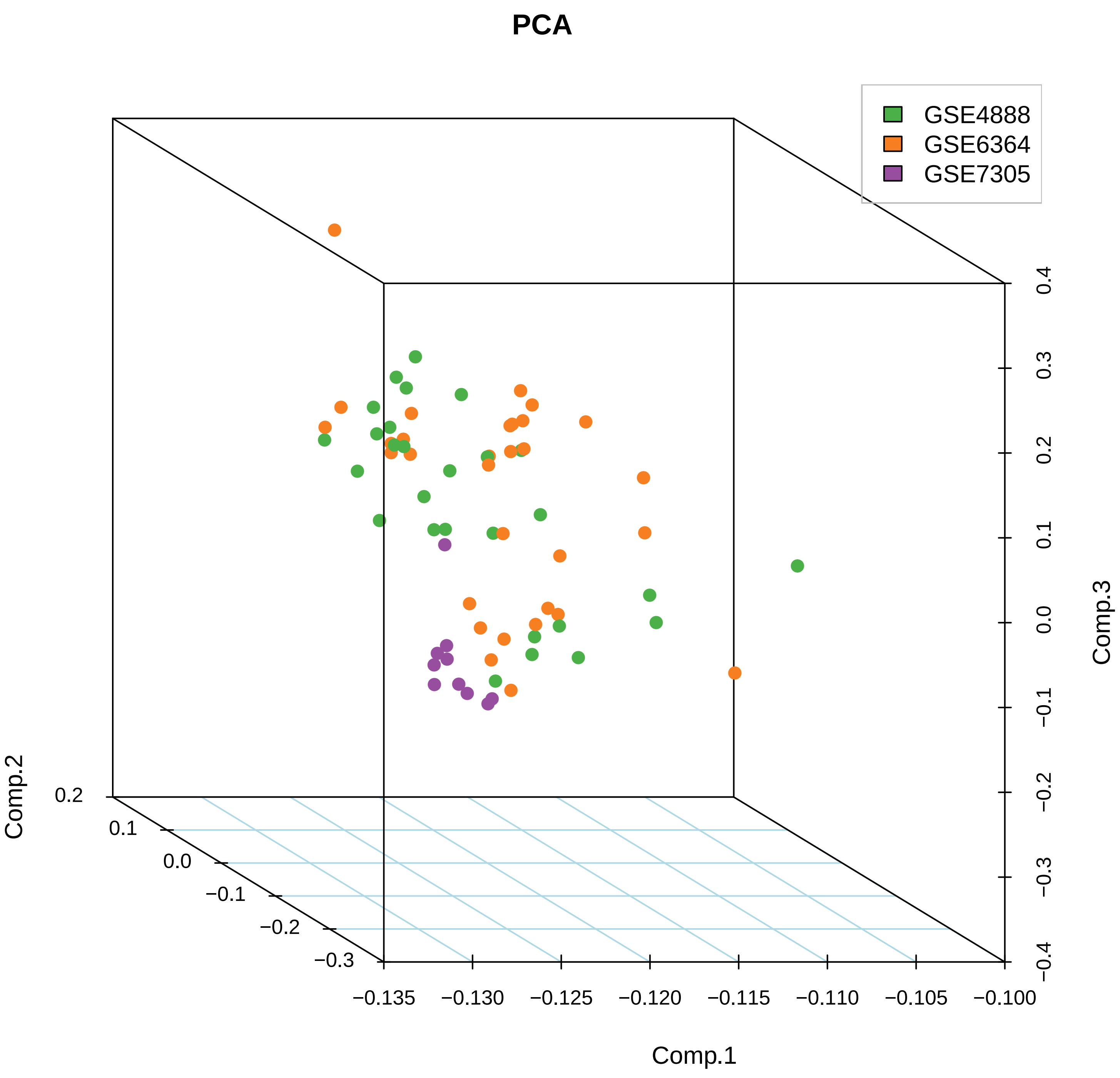
PCA
GSE4888
GSE6364
GSE7305
Comp.1
Comp.2
Comp.3
−0.135
−0.130
−0.125
−0.120
−0.115
−0.110
−0.105
−0.100
0.2
0.1
0.0
−0.1
−0.2
−0.3
0.4
0.3
0.2
0.1
0.0
−0.1
−0.2
−0.3
−0.4

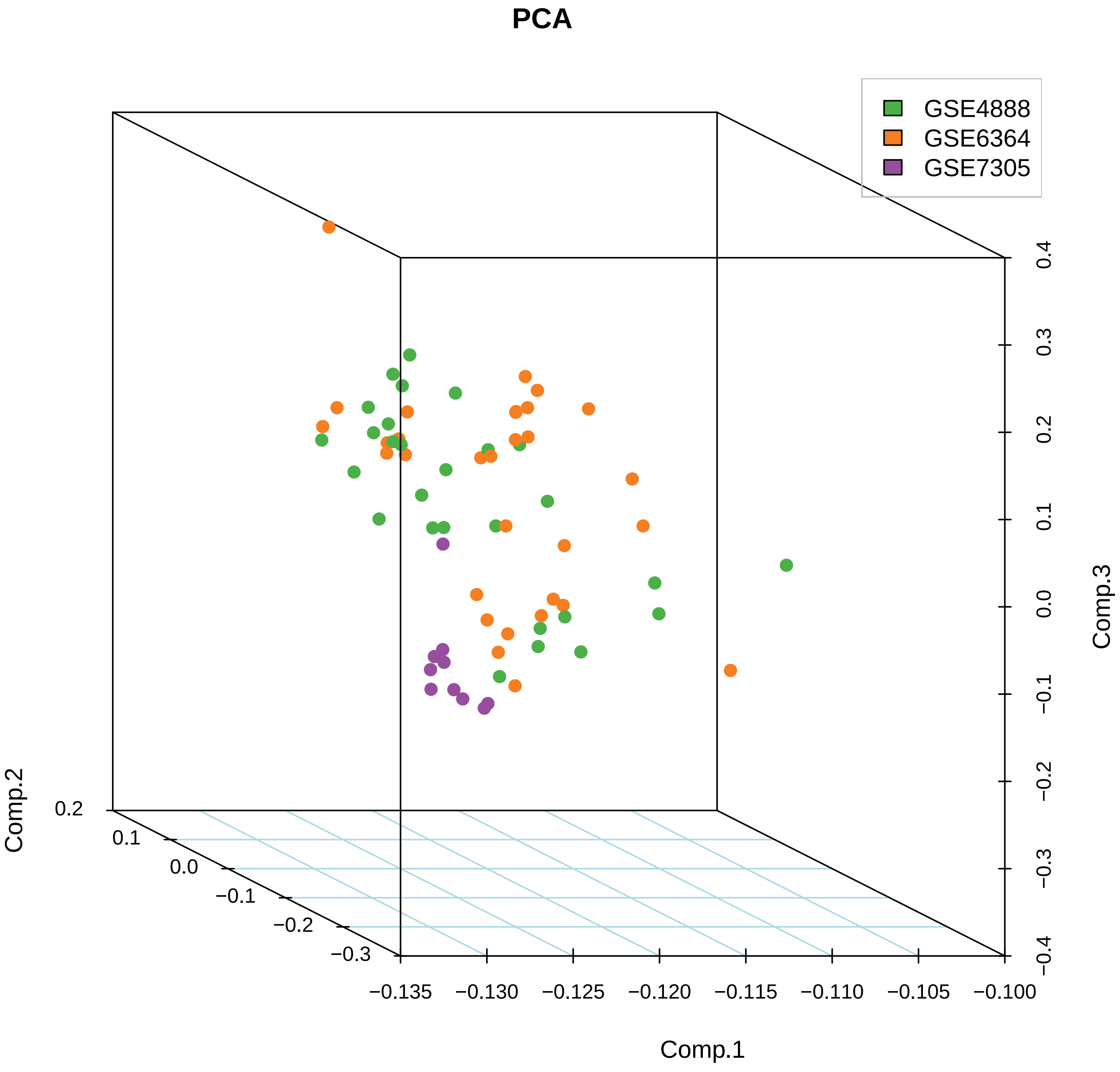

PCA
GSE4888
GSE6364
GSE7305
Comp.1
Comp.2
Comp.3
−0.135
−0.130
−0.125
−0.120
−0.115
−0.110
−0.105
−0.100
0.2
0.1
0.0
−0.1
−0.2
−0.3
0.4
0.3
0.2
0.1
0.0
−0.1
−0.2
−0.3
−0.4

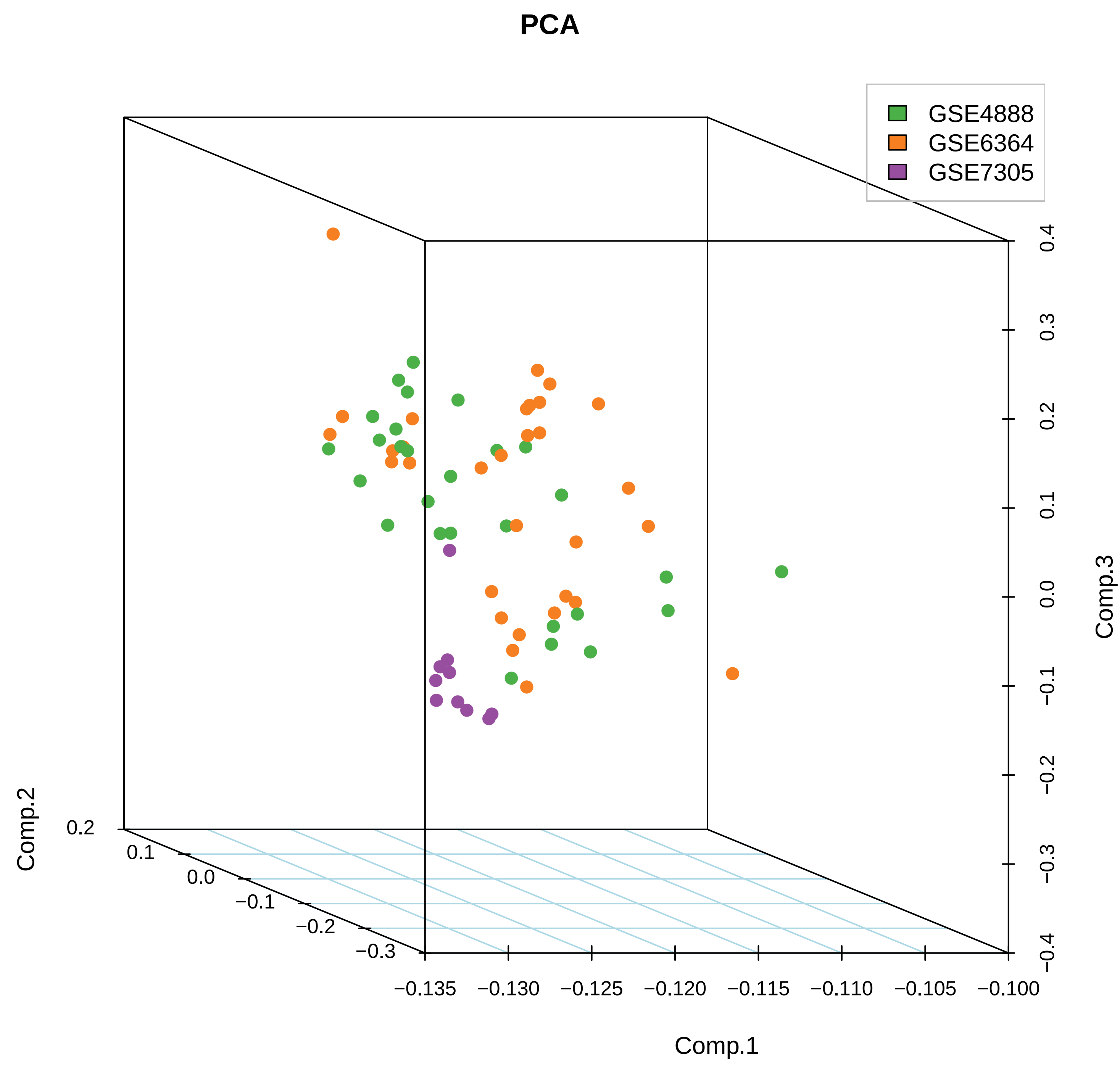
PCA
GSE4888
GSE6364
GSE7305
Comp.1
Comp.2
Comp.3
−0.135
−0.130
−0.125
−0.120
−0.115
−0.110
−0.105
−0.100
0.2
0.1
0.0
−0.1
−0.2
−0.3
0.4
0.3
0.2
0.1
0.0
−0.1
−0.2
−0.3
−0.4

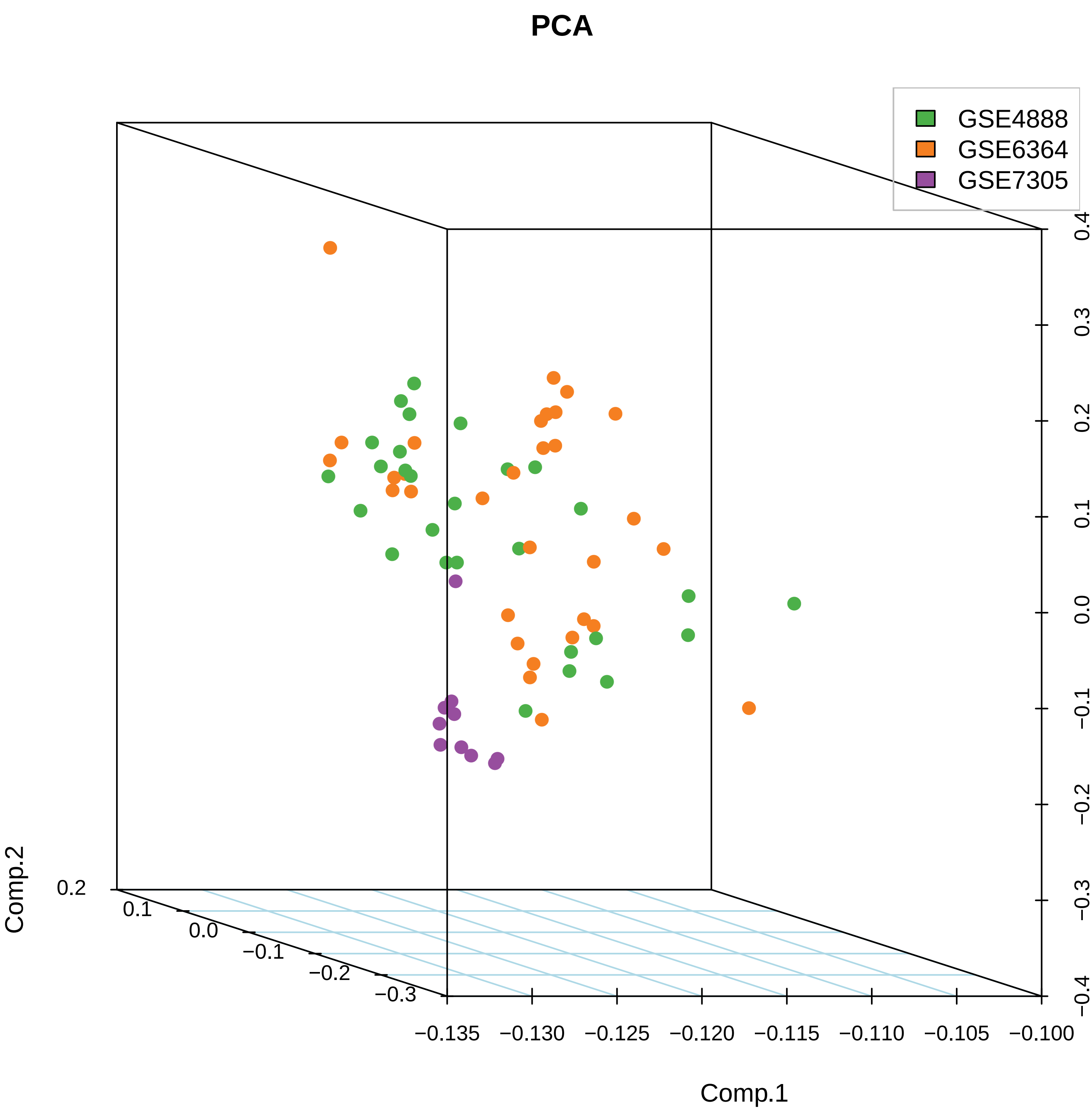
PCA
GSE4888
GSE6364
GSE7305
Comp.1
Comp.2
Comp.3
−0.135
−0.130
−0.125
−0.120
−0.115
−0.110
−0.105
−0.100
0.2
0.1
0.0
−0.1
−0.2
−0.3
−0.4
0.3
0.4

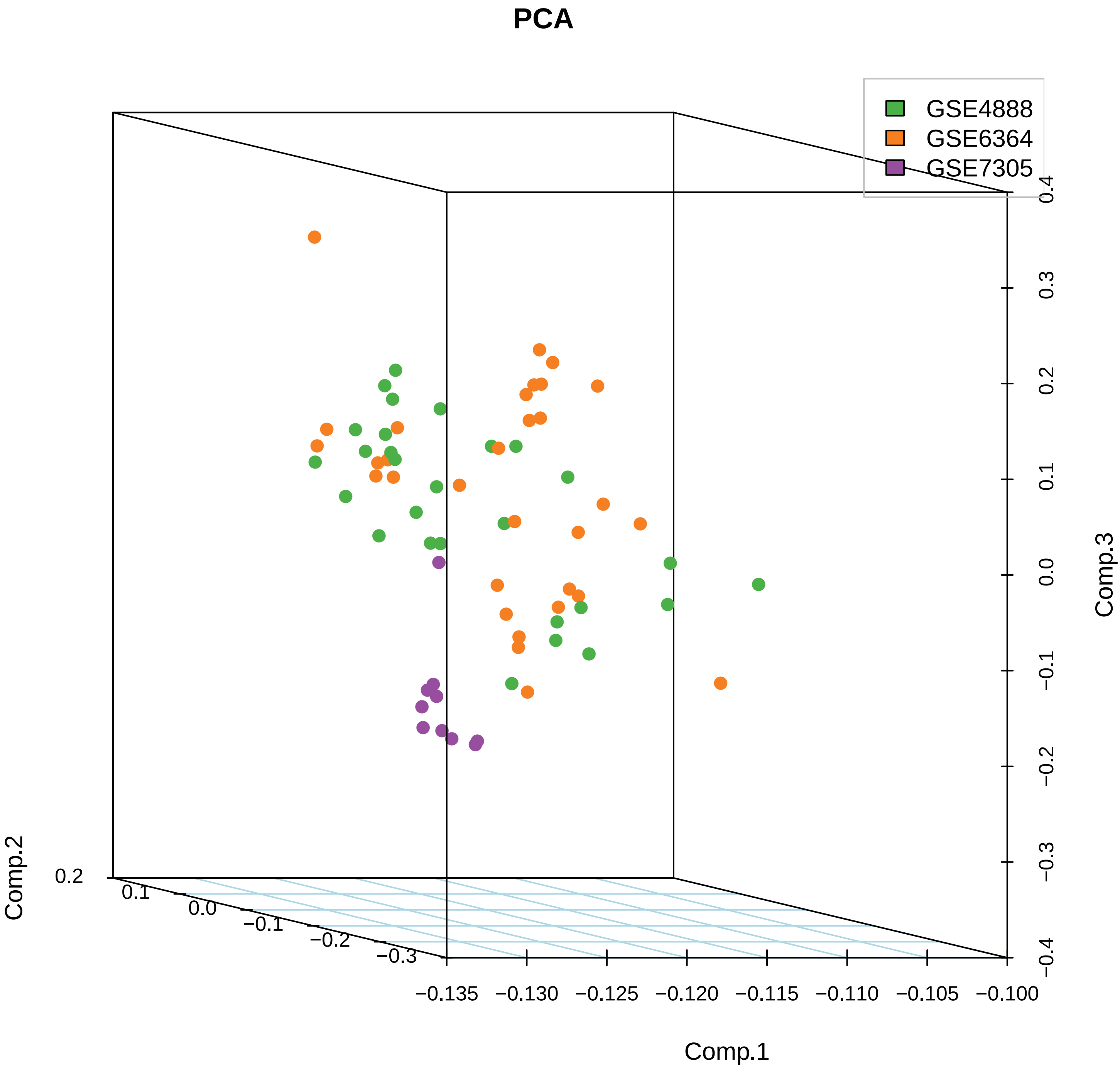
PCA
GSE4888
GSE6364
GSE7305
Comp.1
Comp.2
Comp.3
−0.135
−0.130
−0.125
−0.120
−0.115
−0.110
−0.105
−0.100
0.2
0.1
0.0
−0.1
−0.2
−0.3
−0.4
0.3
0.4

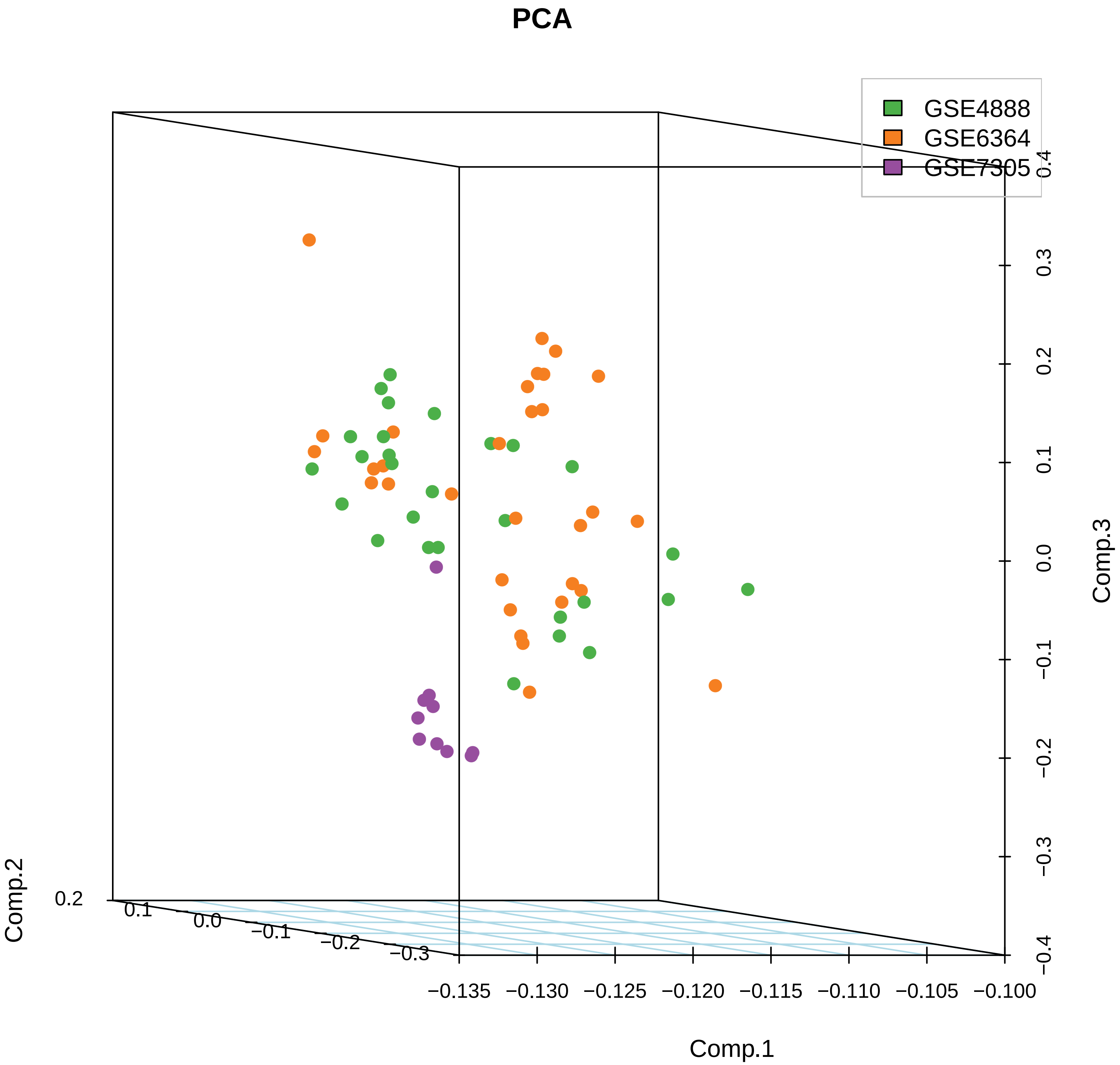
PCA
GSE4888
GSE6364
GSE7305
Comp.1
Comp.2
Comp.3
−0.135 −0.130 −0.125 −0.120 −0.115 −0.110 −0.105 −0.100
0.2 0.1 0.0 −0.1 −0.2 −0.3
−0.4 −0.3 −0.2 −0.1 0.0 0.1 0.2 0.3 0.4

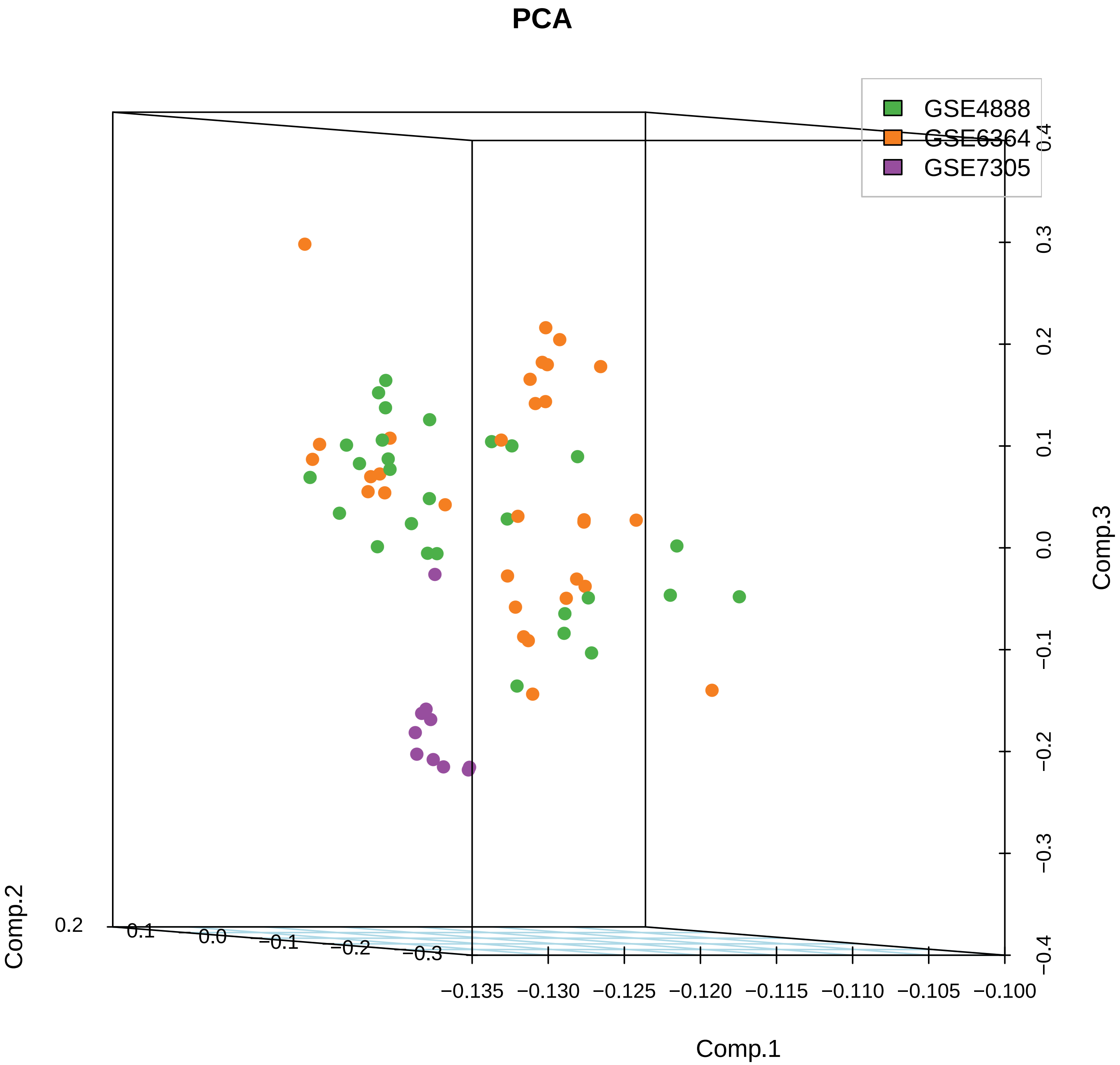
PCA
GSE4888
GSE6364
GSE7305
Comp.1
Comp.2
Comp.3
−0.135
−0.130
−0.125
−0.120
−0.115
−0.110
−0.105
−0.100
0.2
0.1
0.0
−0.1
−0.2
−0.3
−0.4
0.3
0.4

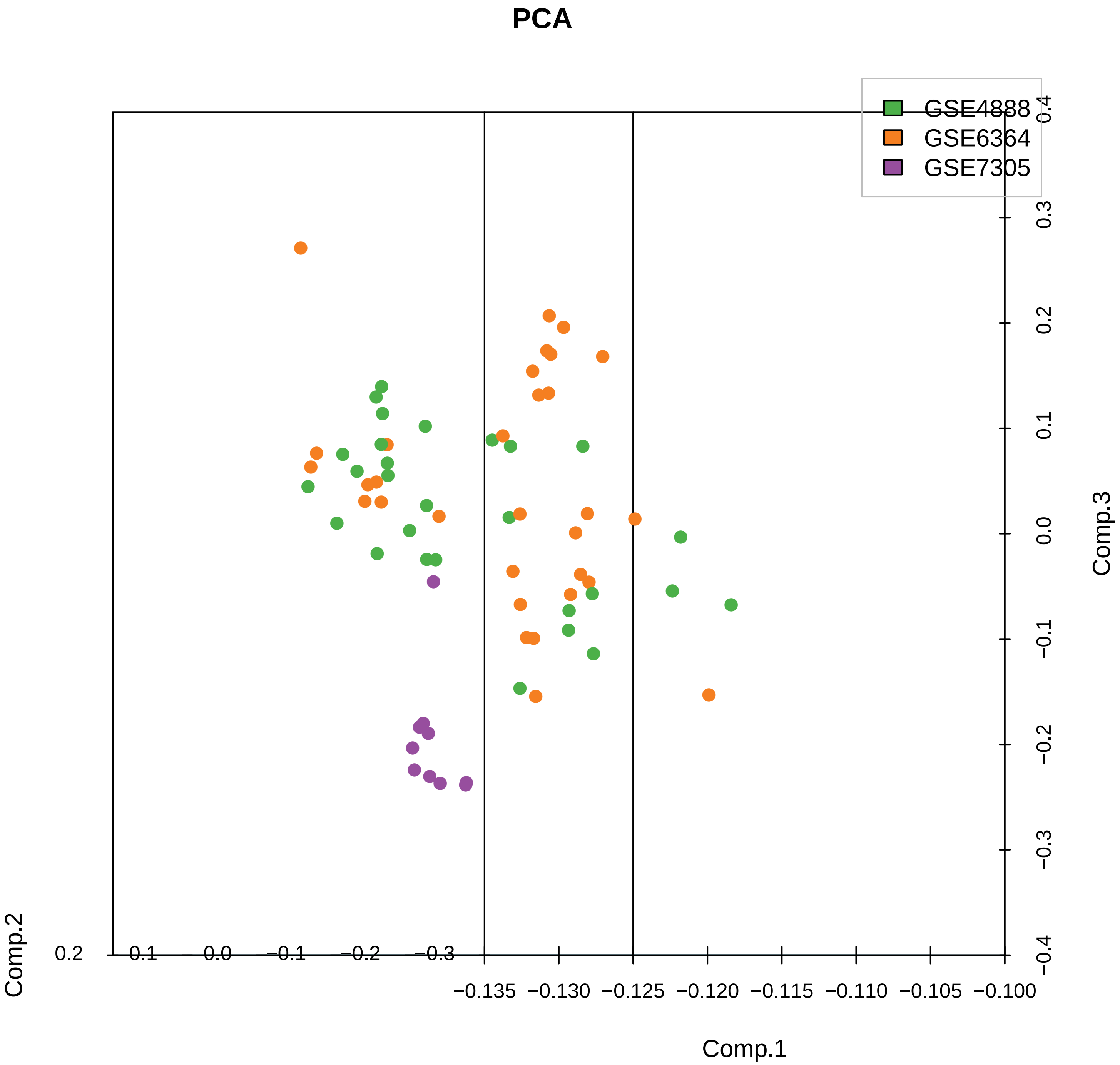

PCA
GSE4888
GSE6364
GSE7305
Comp.1
Comp.2
Comp.3
−0.135 −0.130 −0.125 −0.120 −0.115 −0.110 −0.105 −0.100
0.2 0.1 0.0 −0.1 −0.2 −0.3
−0.4 −0.3 −0.2 −0.1 0.0 0.1 0.2 0.3 0.4

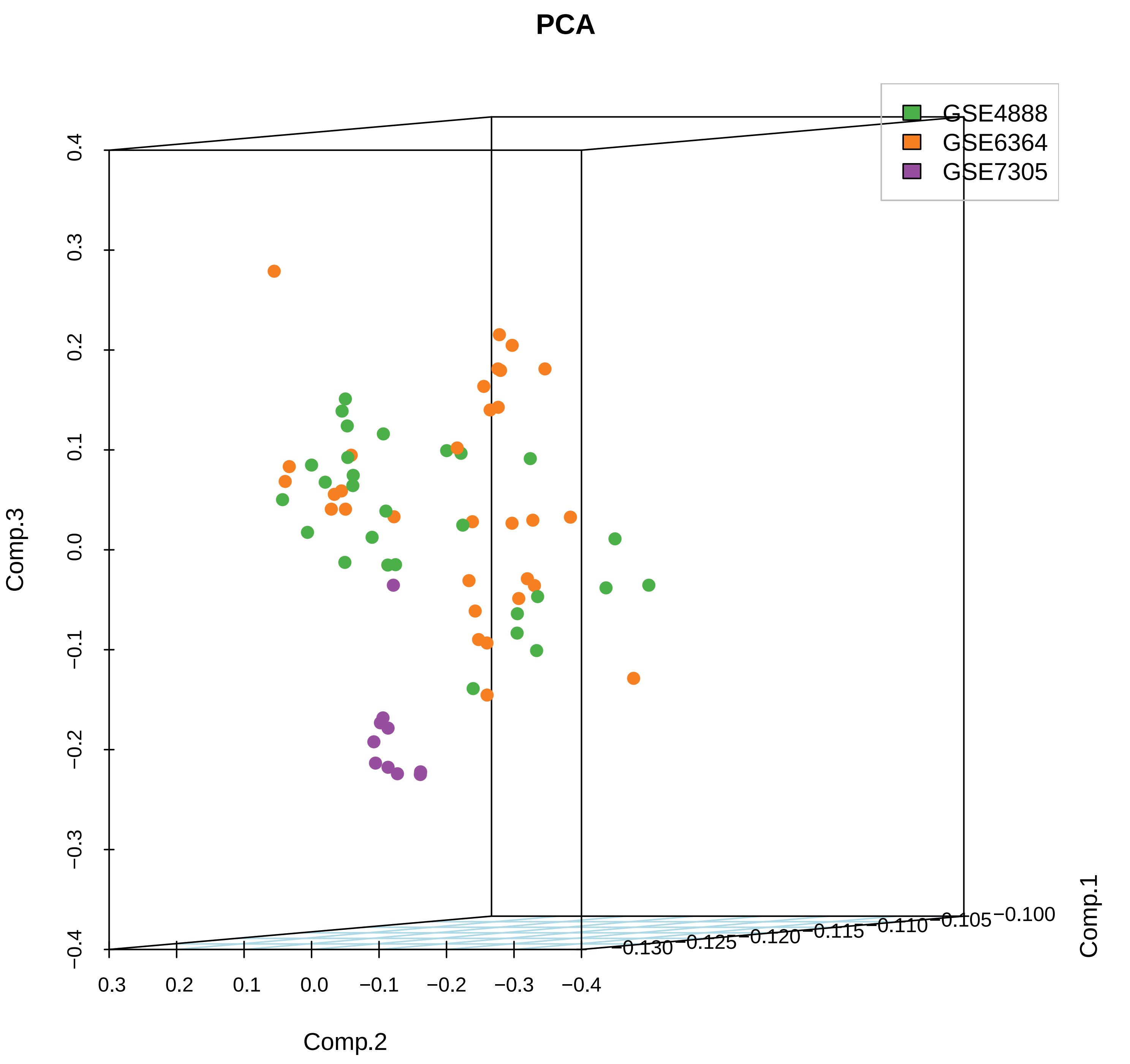

PCA
GSE4888
GSE6364
GSE7305
Comp.3
0.4
0.3
0.2
0.1
0.0
−0.1
−0.2
−0.3
−0.4
Comp.2
0.3
0.2
0.1
0.0
−0.1
−0.2
−0.3
−0.4
Comp.1
−0.130
−0.125
−0.120
−0.115
−0.110
−0.105
−0.100

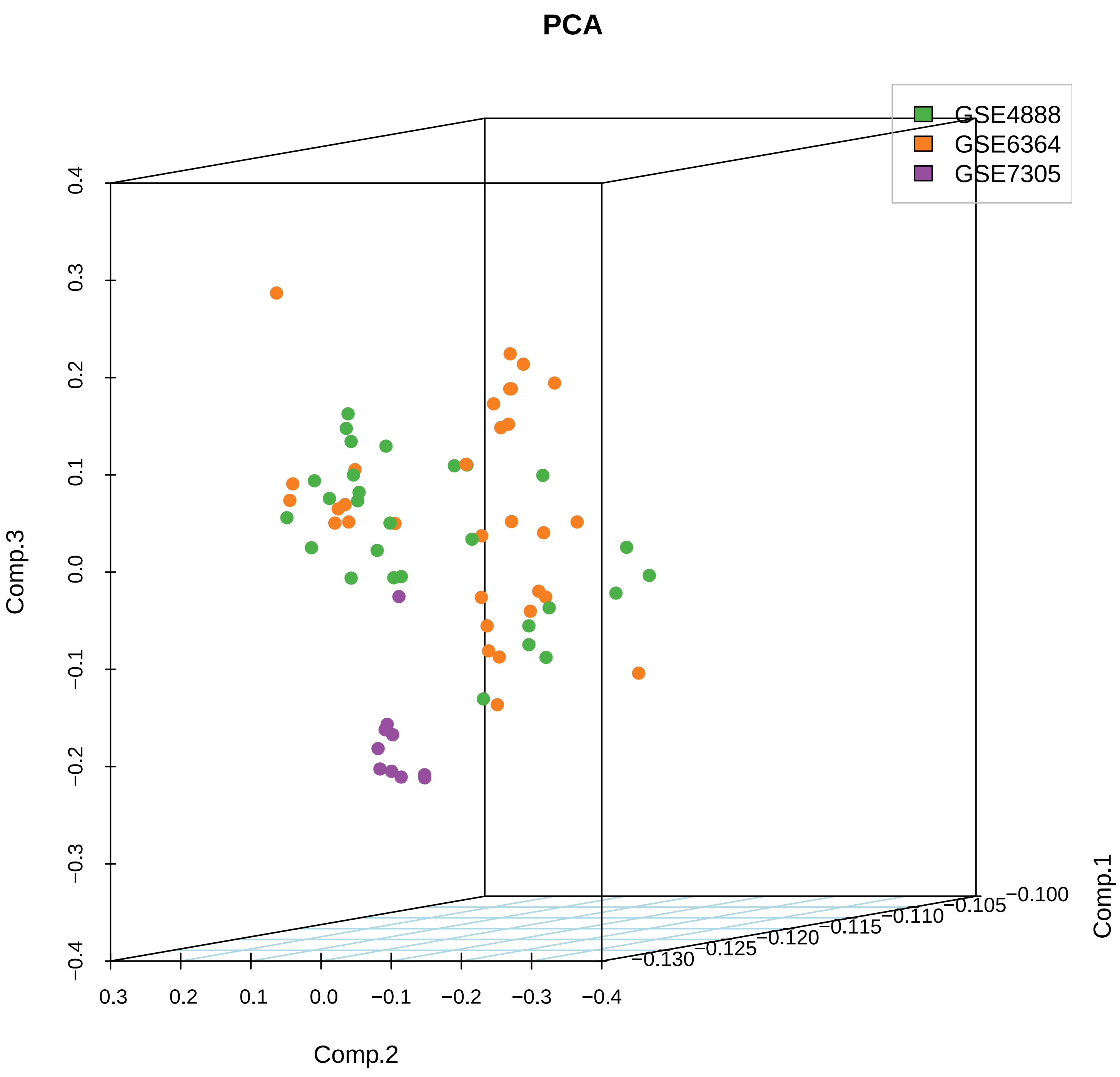

PCA
GSE4888
GSE6364
GSE7305
Comp.3
0.4
0.3
0.2
0.1
0.0
−0.1
−0.2
−0.3
−0.4
Comp.2
0.3
0.2
0.1
0.0
−0.1
−0.2
−0.3
−0.4
Comp.1
−0.130
−0.125
−0.120
−0.115
−0.110
−0.105
−0.100

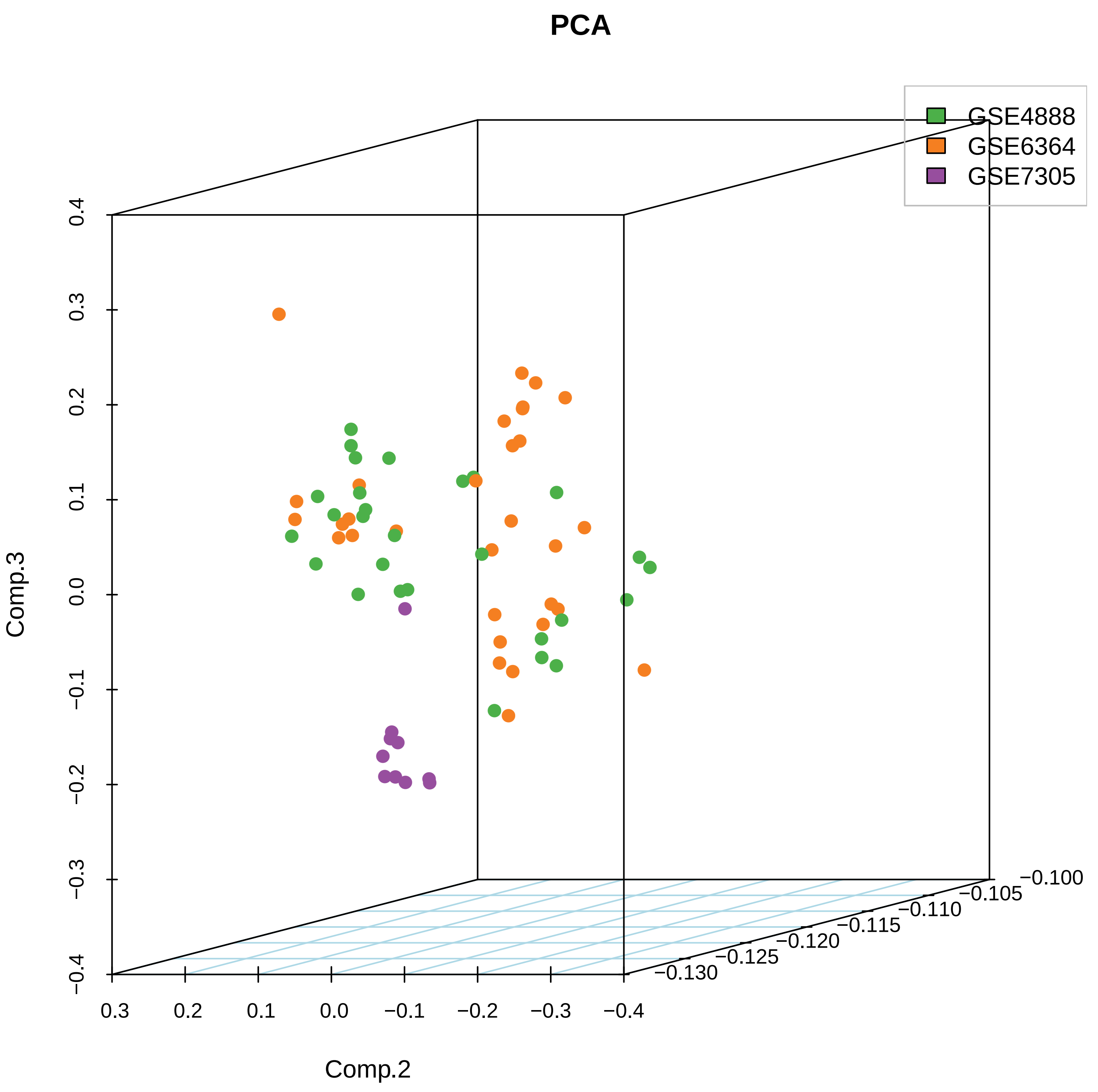

PCA
GSE4888
GSE6364
GSE7305
Comp.3
Comp.2
Comp.1
0.4
0.3
0.2
0.1
0.0
−0.1
−0.2
−0.3
−0.4
0.3
0.2
0.1
0.0
−0.1
−0.2
−0.3
−0.4
−0.100
−0.105
−0.110
−0.115
−0.120
−0.125
−0.130

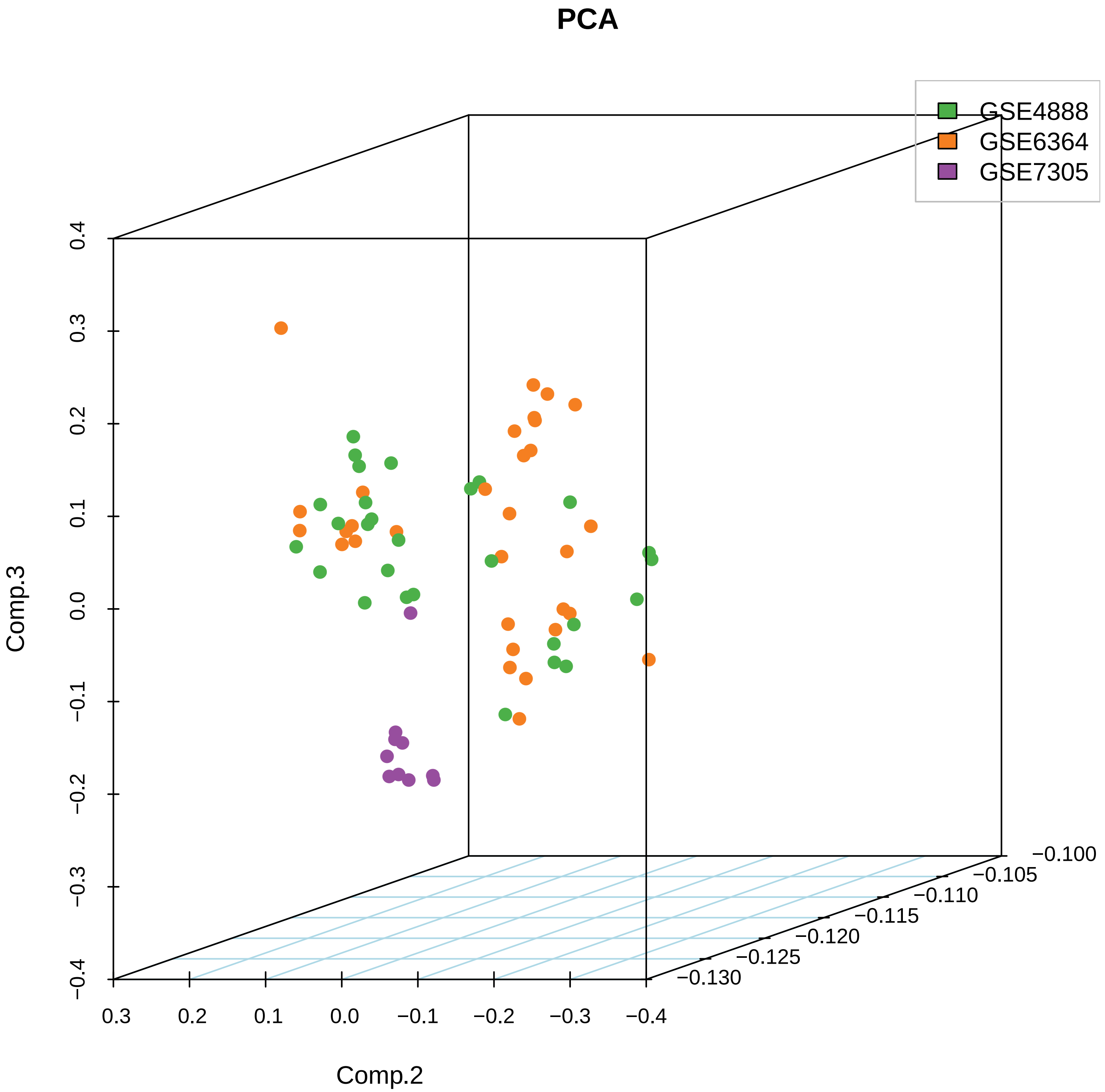

PCA
GSE4888
GSE6364
GSE7305
Comp.3
0.4
0.3
0.2
0.1
0.0
−0.1
−0.2
−0.3
−0.4
Comp.2
0.3
0.2
0.1
0.0
−0.1
−0.2
−0.3
−0.4
Comp.1
−0.100
−0.105
−0.110
−0.115
−0.120
−0.125
−0.130

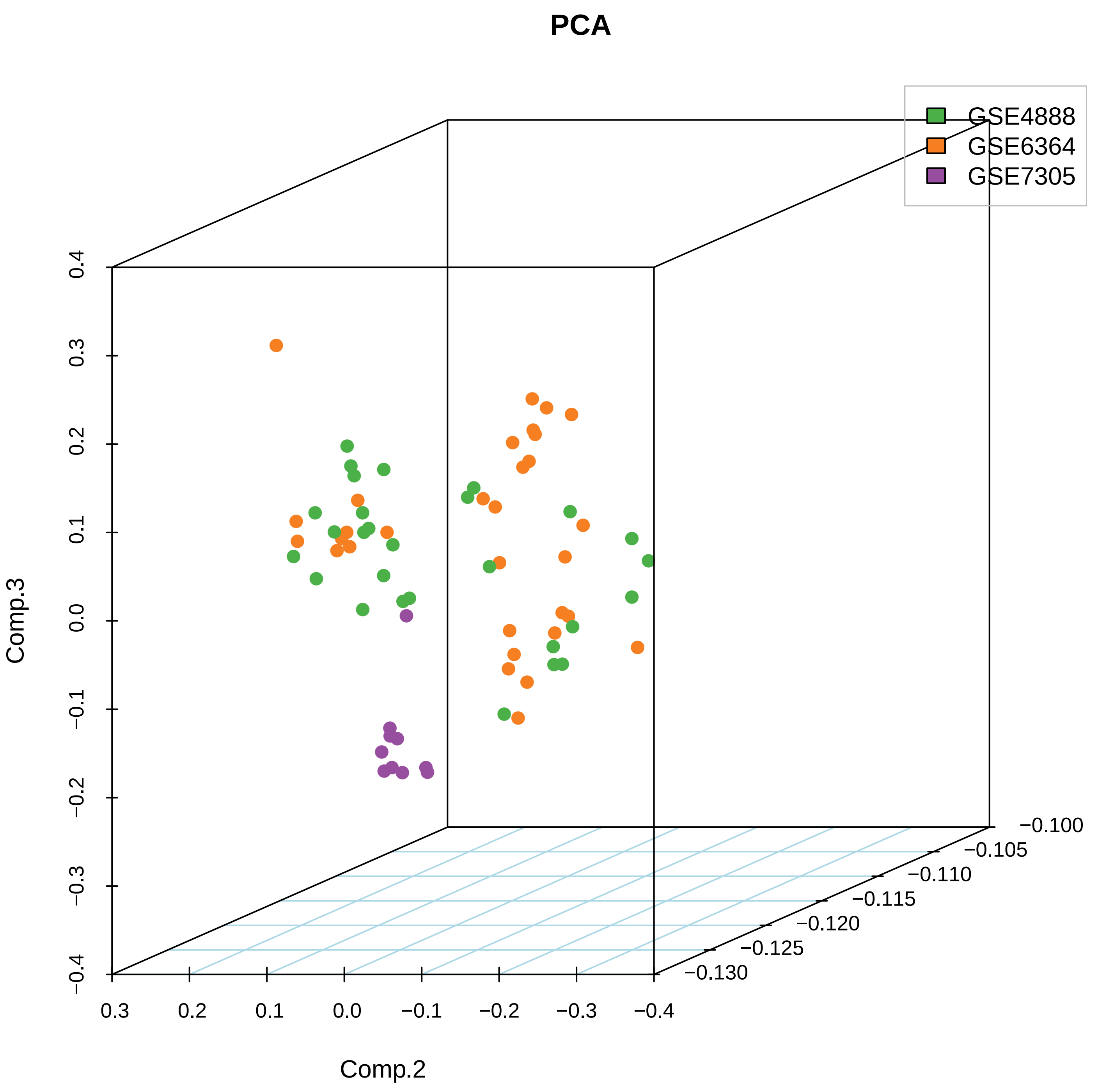
PCA
GSE4888
GSE6364
GSE7305
Comp.3
0.4
0.3
0.2
0.1
0.0
−0.1
−0.2
−0.3
−0.4
Comp.2
0.3
0.2
0.1
0.0
−0.1
−0.2
−0.3
−0.4
Comp.1
−0.100
−0.105
−0.110
−0.115
−0.120
−0.125
−0.130

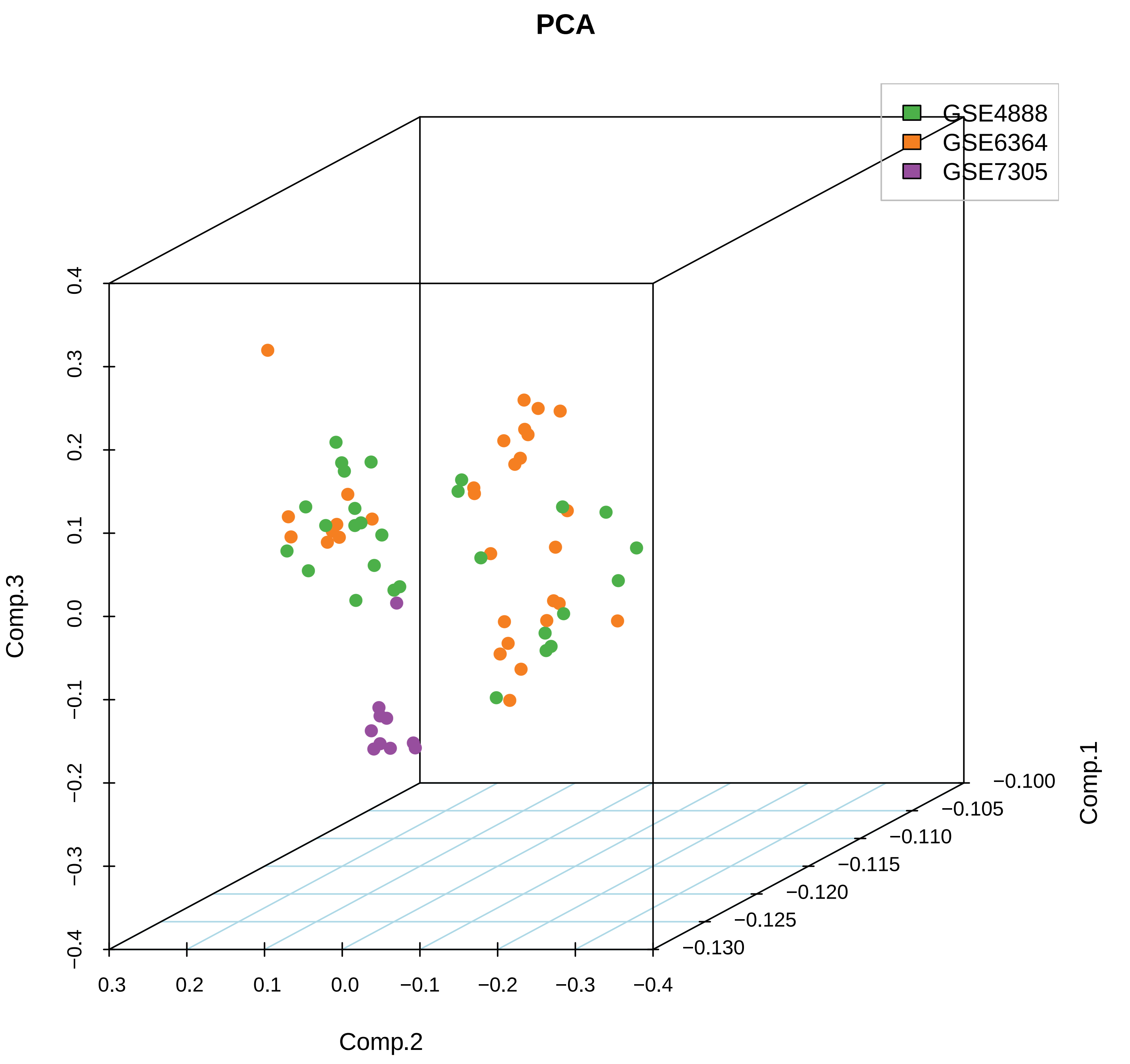
PCA
GSE4888
GSE6364
GSE7305
Comp.3
Comp.2
Comp.1
0.4
0.3
0.2
0.1
0.0
−0.1
−0.2
−0.3
−0.4
0.3
0.2
0.1
0.0
−0.1
−0.2
−0.3
−0.4
−0.100
−0.105
−0.110
−0.115
−0.120
−0.125
−0.130

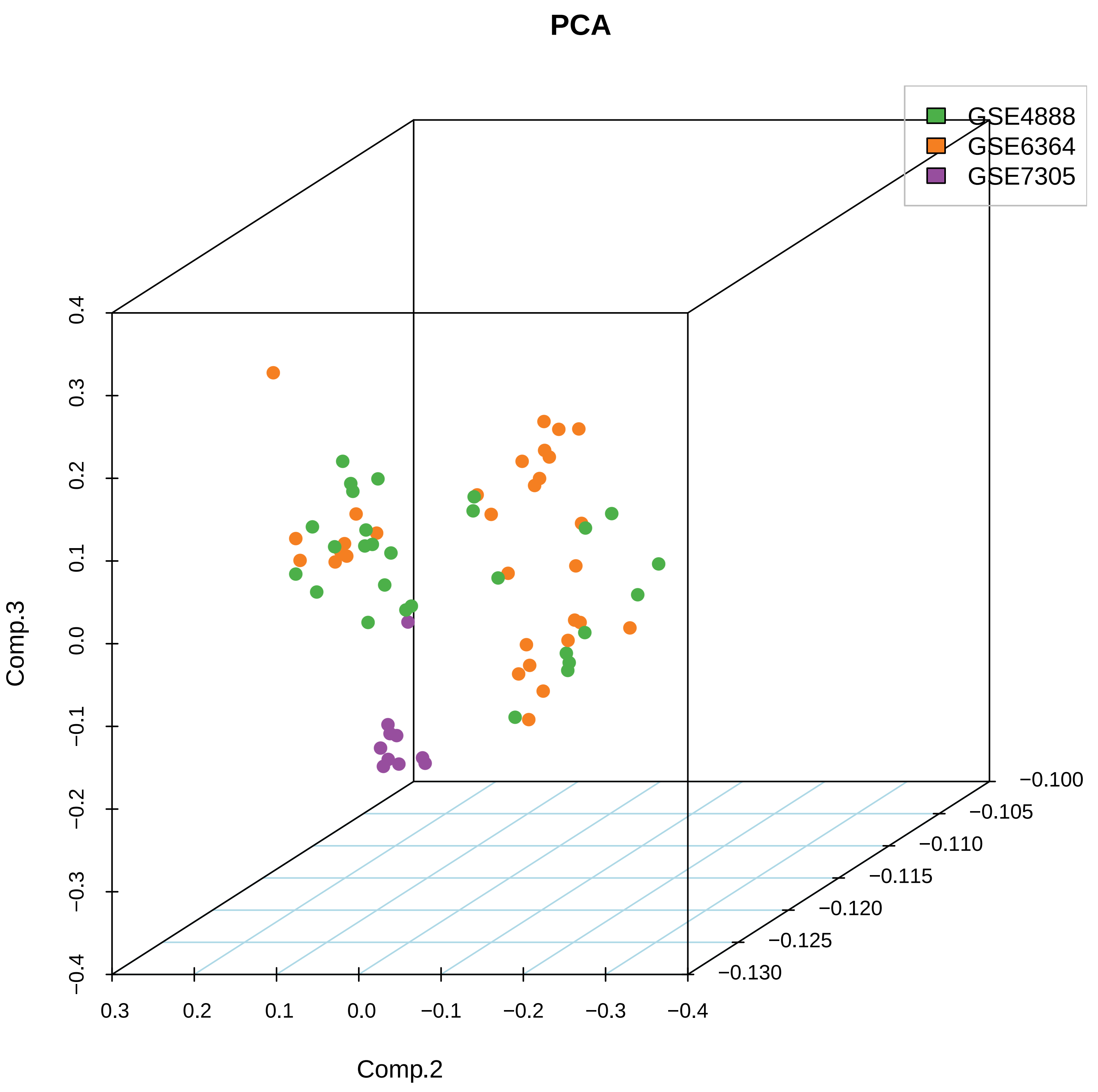
PCA
GSE4888
GSE6364
GSE7305
Comp.3
0.4
0.3
0.2
0.1
0.0
−0.1
−0.2
−0.3
−0.4
0.3
0.2
0.1
0.0
−0.1
−0.2
−0.3
−0.4
Comp.2
Comp.1
−0.100
−0.105
−0.110
−0.115
−0.120
−0.125
−0.130

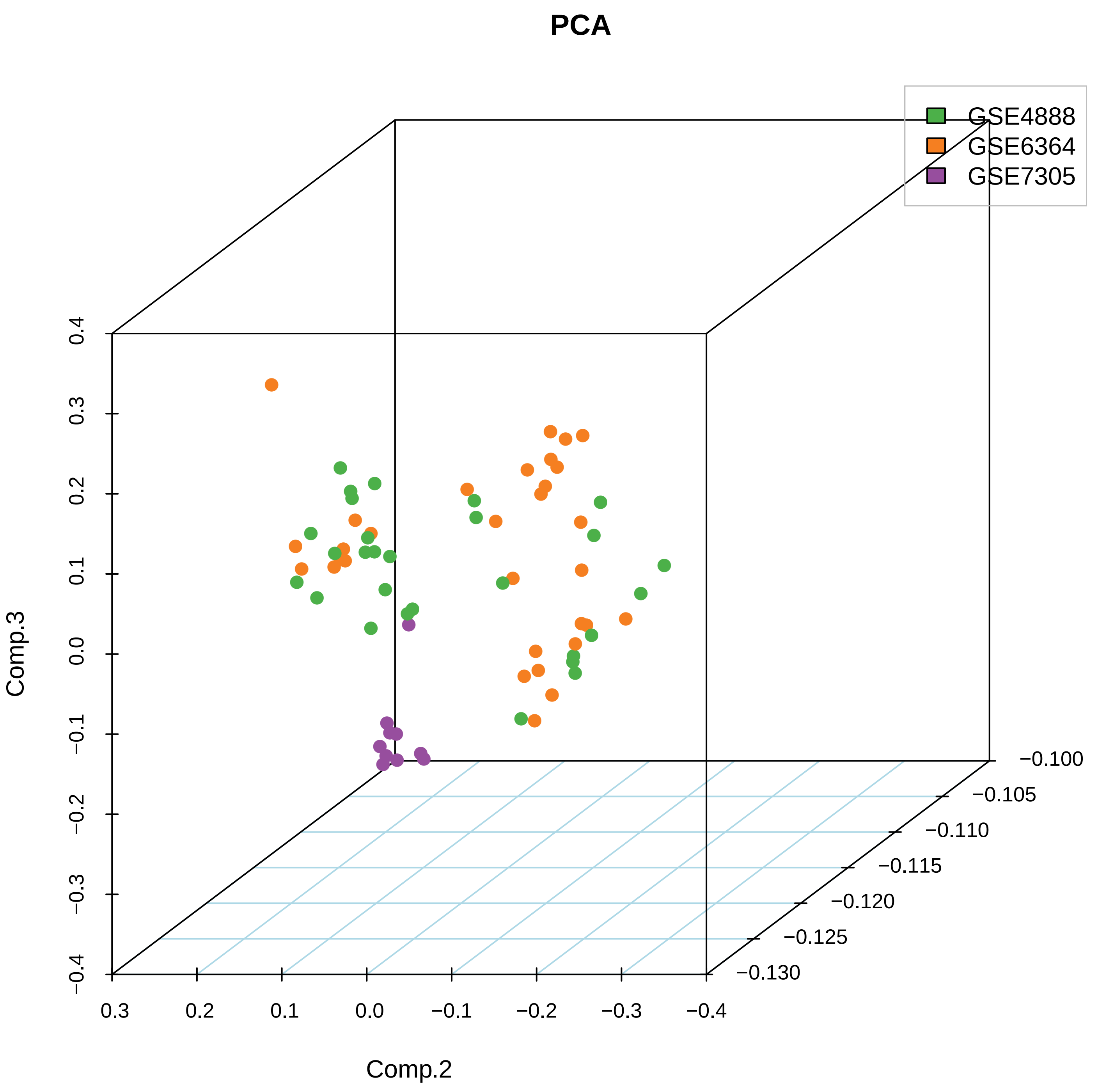
PCA
GSE4888
GSE6364
GSE7305
Comp.3
0.4
0.3
0.2
0.1
0.0
−0.1
−0.2
−0.3
−0.4
Comp.2
0.3
0.2
0.1
0.0
−0.1
−0.2
−0.3
−0.4
Comp.1
−0.100
−0.105
−0.110
−0.115
−0.120
−0.125
−0.130

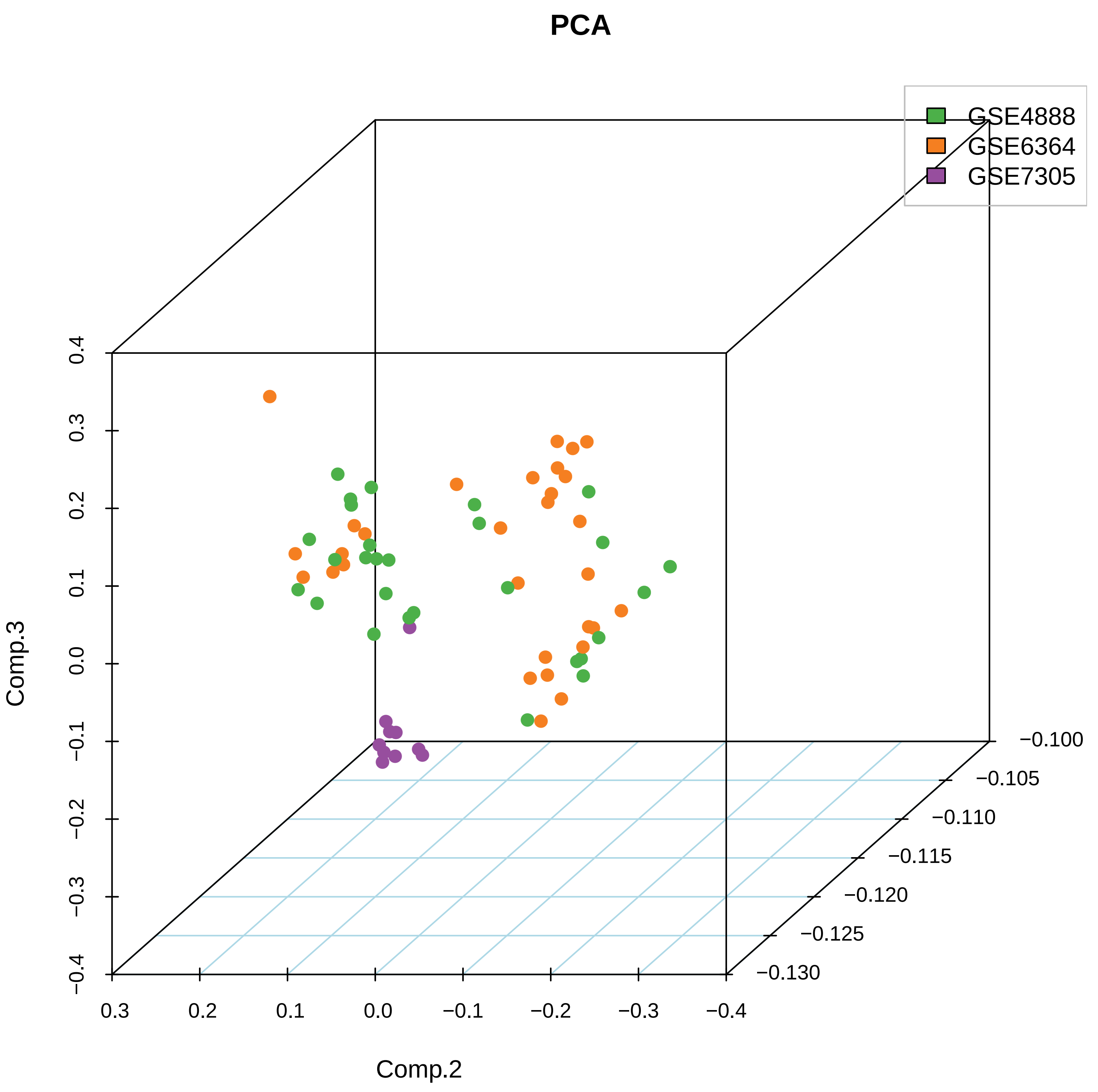

PCA
GSE4888
GSE6364
GSE7305
Comp.3
0.4
0.3
0.2
0.1
0.0
−0.1
−0.2
−0.3
−0.4
Comp.2
0.3
0.2
0.1
0.0
−0.1
−0.2
−0.3
−0.4
Comp.1
−0.100
−0.105
−0.110
−0.115
−0.120
−0.125
−0.130

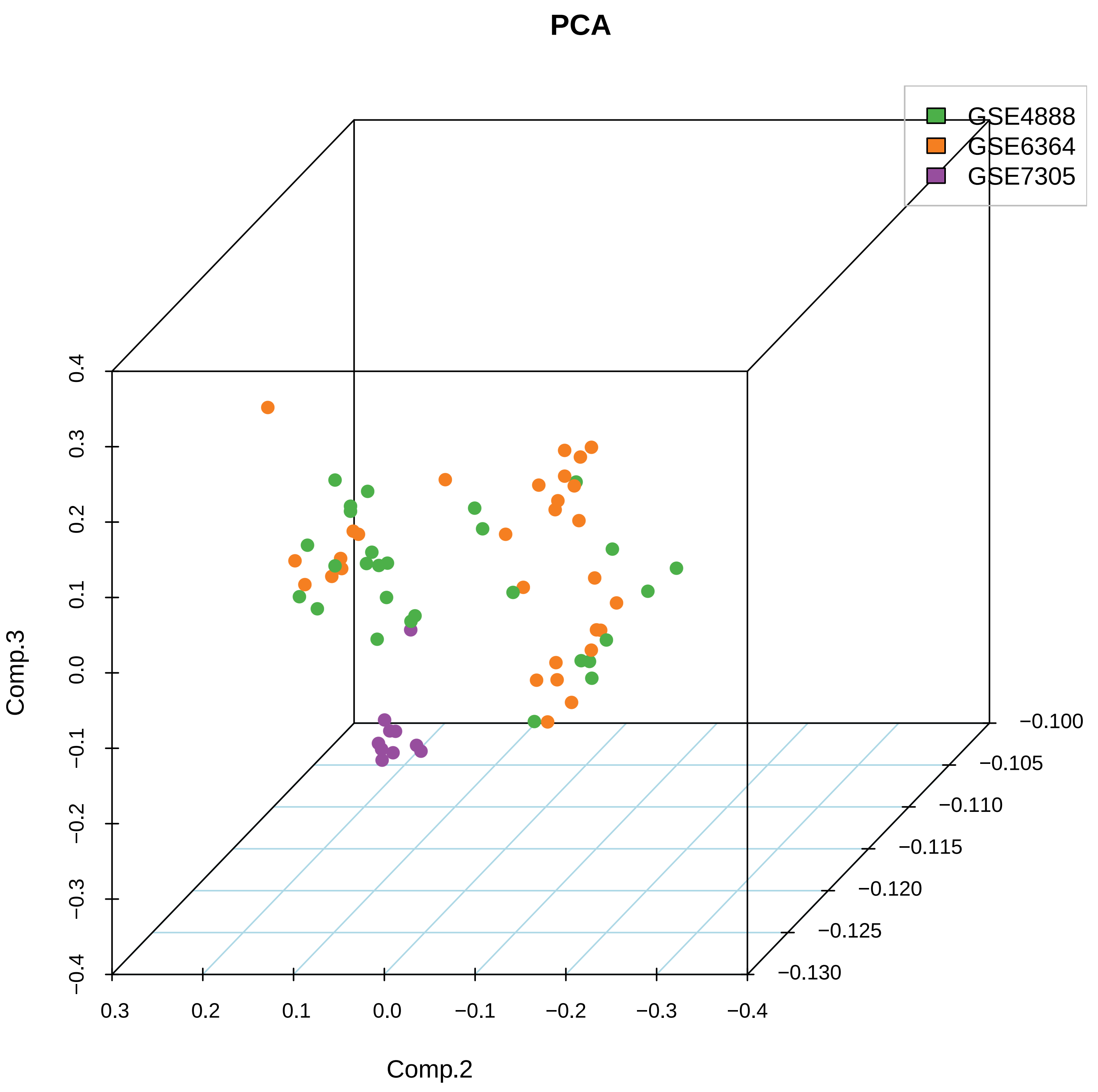

PCA
GSE4888
GSE6364
GSE7305
Comp.3
0.4
0.3
0.2
0.1
0.0
−0.1
−0.2
−0.3
−0.4
Comp.2
0.3
0.2
0.1
0.0
−0.1
−0.2
−0.3
−0.4
Comp.1
−0.100
−0.105
−0.110
−0.115
−0.120
−0.125
−0.130

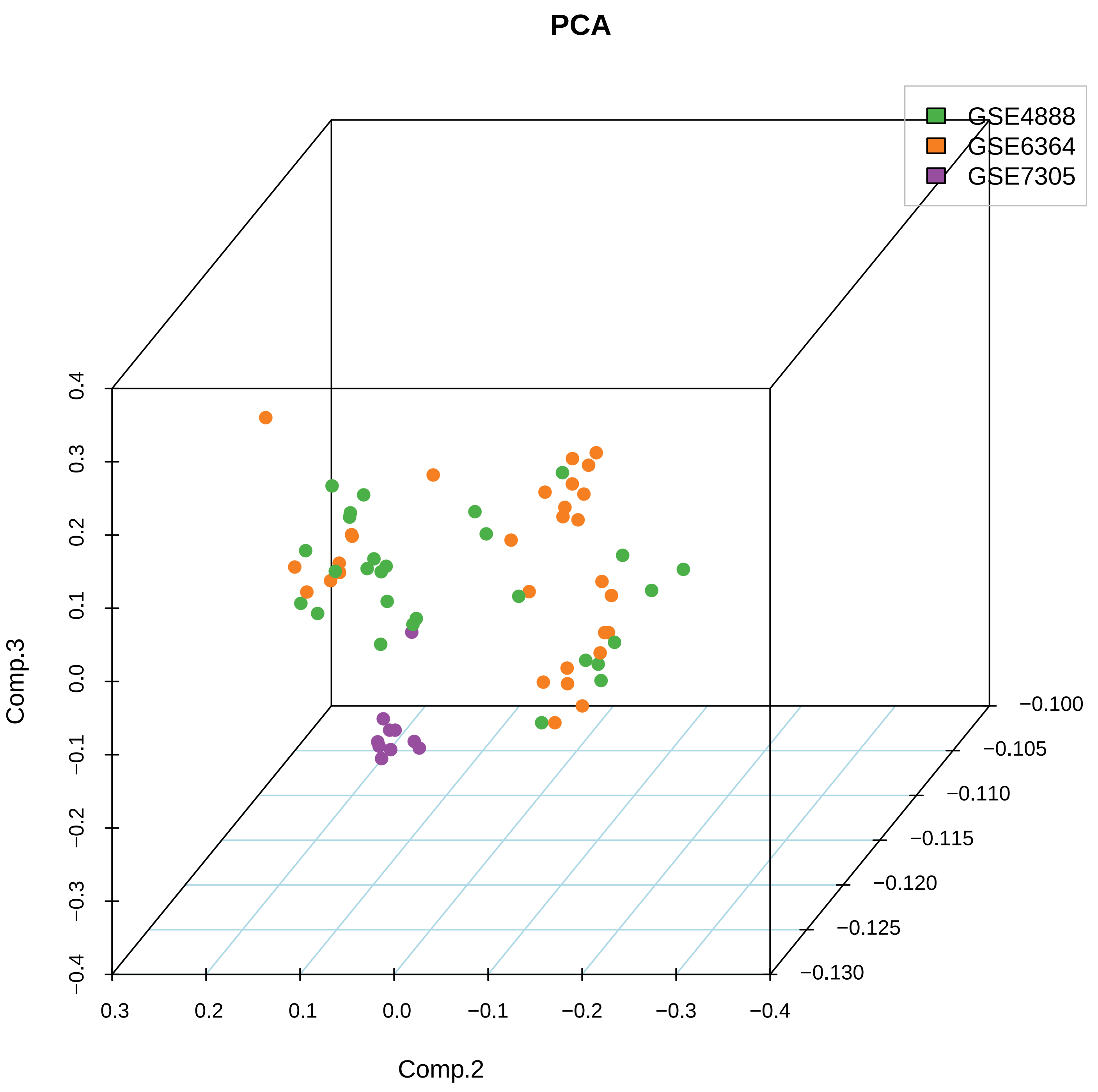
PCA
GSE4888
GSE6364
GSE7305
Comp.3
0.4
0.3
0.2
0.1
0.0
−0.1
−0.2
−0.3
−0.4
0.3
0.2
0.1
0.0
−0.1
−0.2
−0.3
−0.4
Comp.2
Comp.1
−0.100
−0.105
−0.110
−0.115
−0.120
−0.125
−0.130

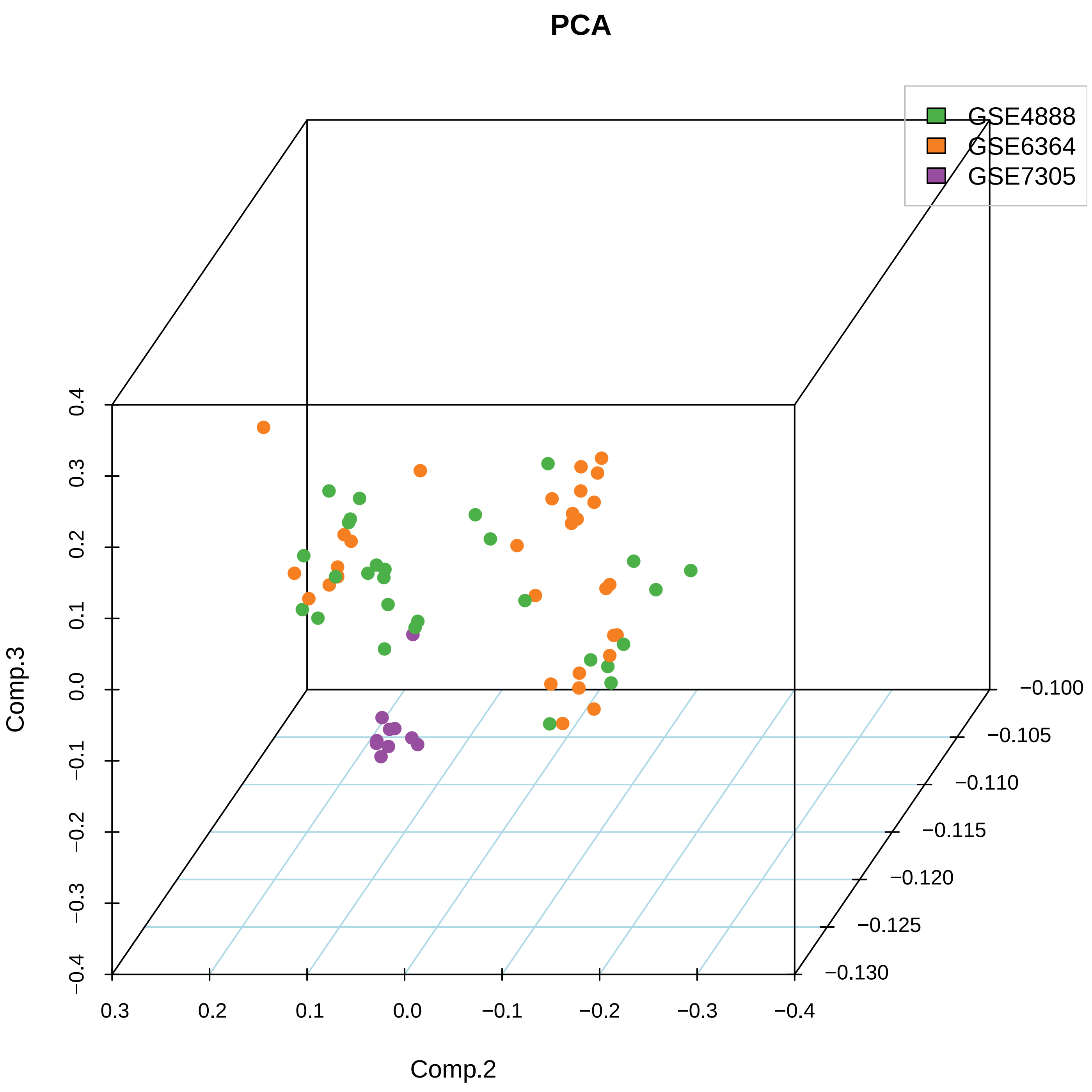

PCA
GSE4888
GSE6364
GSE7305
Comp.1
Comp.2
Comp.3
0.4
0.3
0.2
0.1
0.0
−0.1
−0.2
−0.3
−0.4
0.3
0.2
0.1
0.0
−0.1
−0.2
−0.3
−0.4
−0.100
−0.105
−0.110
−0.115
−0.120
−0.125
−0.130

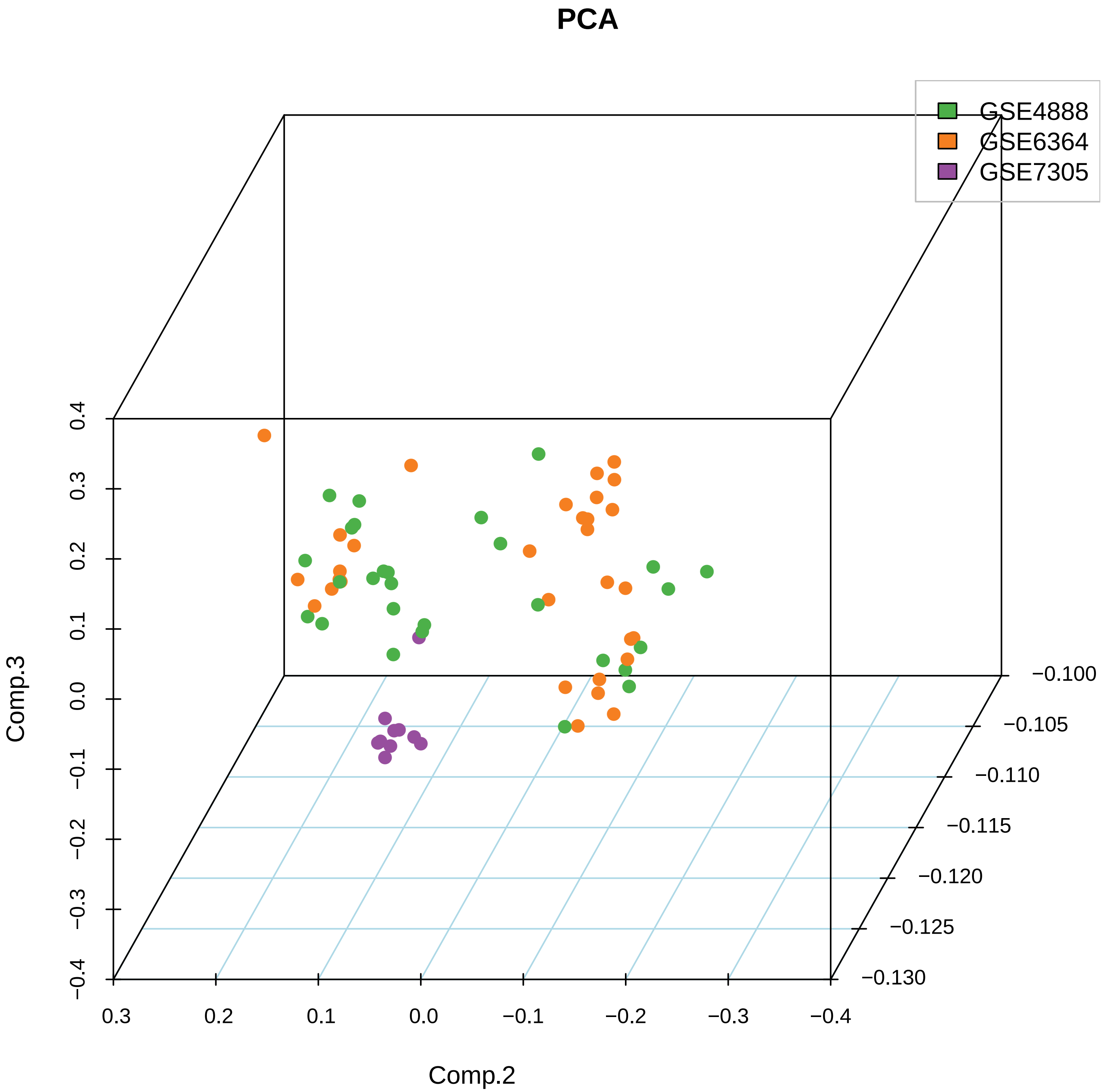
PCA
GSE4888
GSE6364
GSE7305
Comp.1
Comp.2
Comp.3
0.4
0.3
0.2
0.1
0.0
−0.1
−0.2
−0.3
−0.4
0.3
0.2
0.1
0.0
−0.1
−0.2
−0.3
−0.4
−0.100
−0.105
−0.110
−0.115
−0.120
−0.125
−0.130

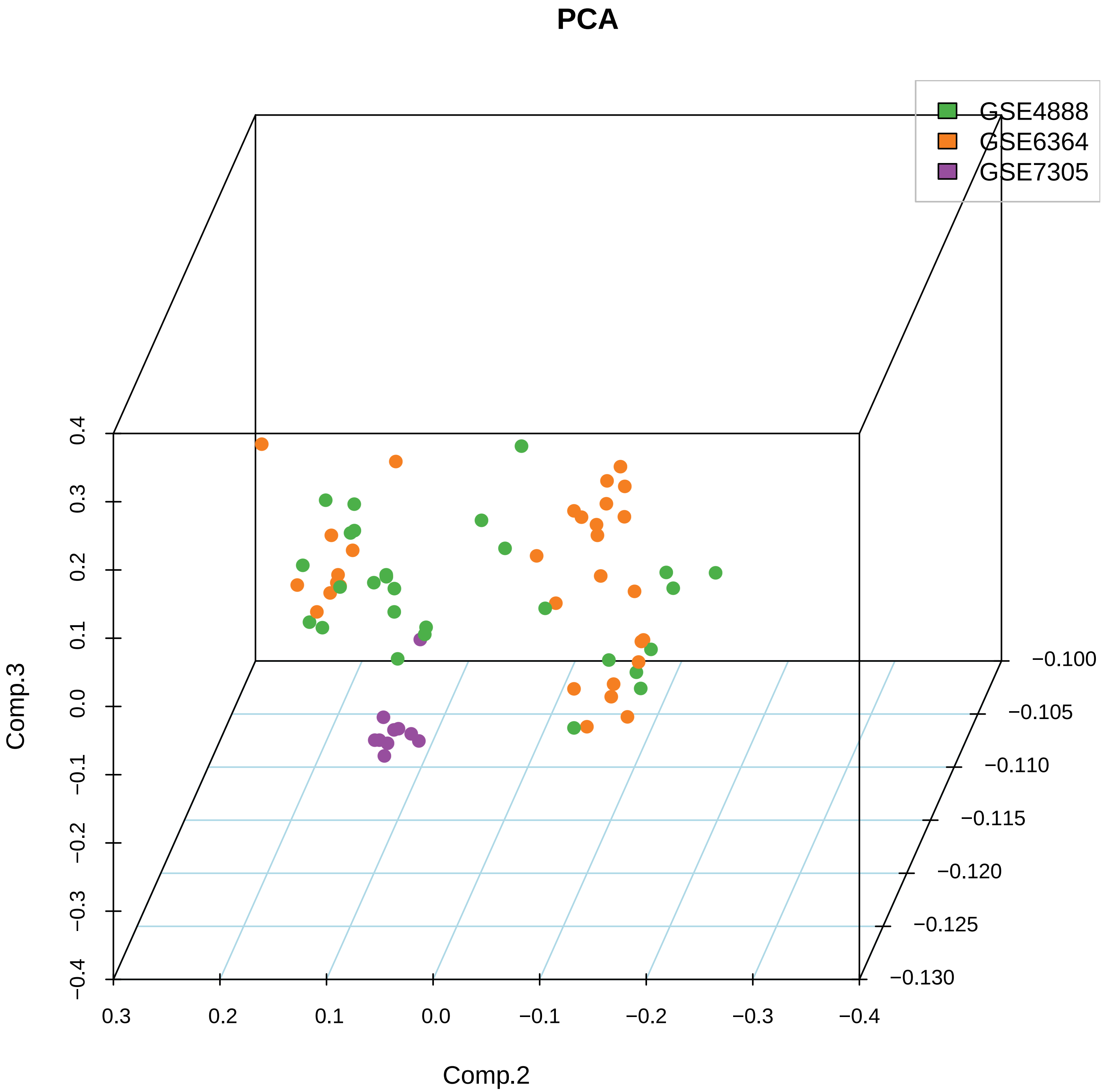

PCA
GSE4888
GSE6364
GSE7305
Comp.3
0.4
0.3
0.2
0.1
0.0
−0.1
−0.2
−0.3
−0.4
Comp.2
0.3
0.2
0.1
0.0
−0.1
−0.2
−0.3
−0.4
Comp.1
−0.100
−0.105
−0.110
−0.115
−0.120
−0.125
−0.130

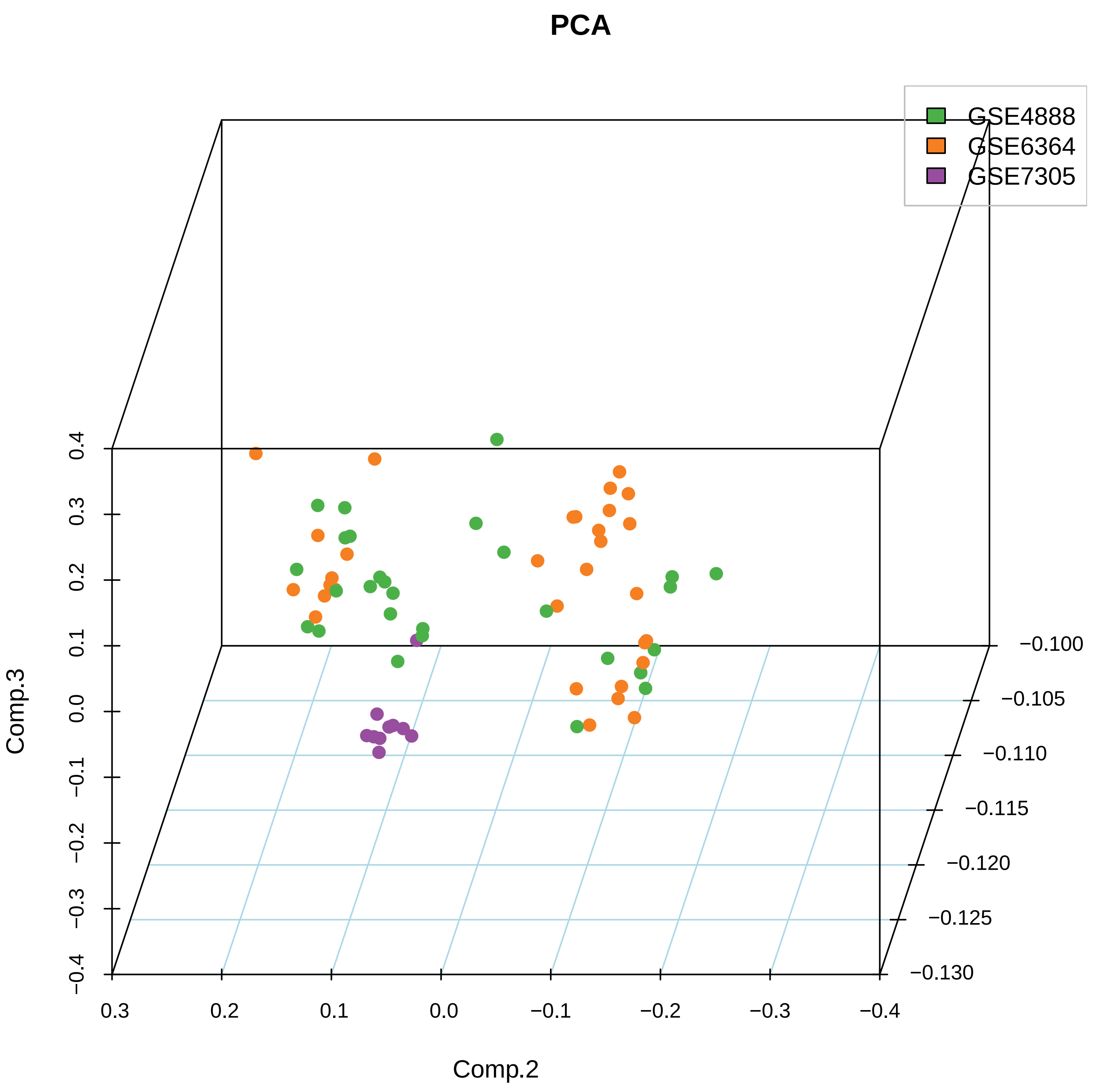
PCA
GSE4888
GSE6364
GSE7305
Comp.1
Comp.2
Comp.3
0.4
0.3
0.2
0.1
0.0
−0.1
−0.2
−0.3
−0.4
0.3
0.2
0.1
0.0
−0.1
−0.2
−0.3
−0.4
−0.100
−0.105
−0.110
−0.115
−0.120
−0.125
−0.130

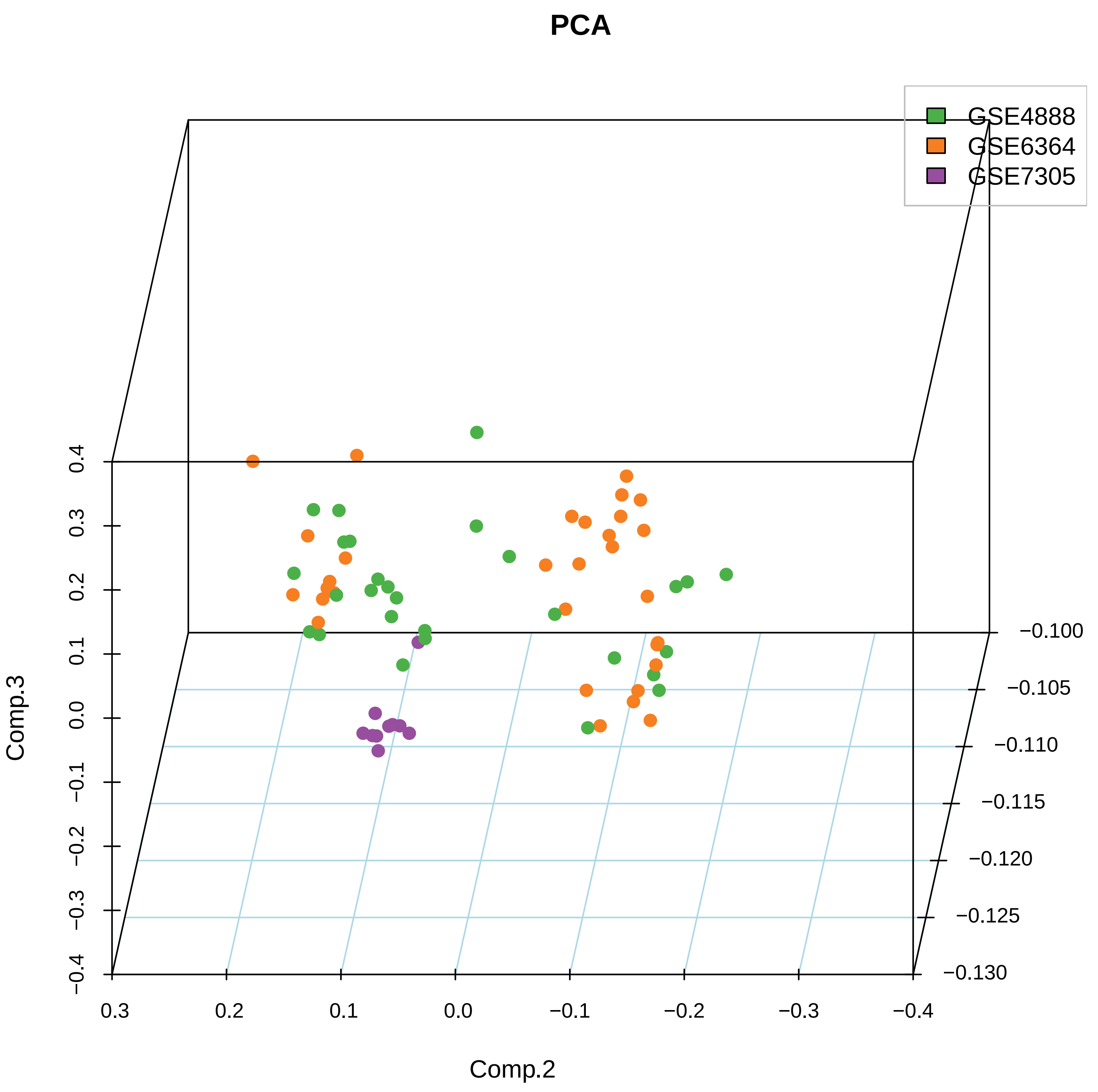
PCA
GSE4888
GSE6364
GSE7305
Comp.3
Comp.2
Comp.1
0.4
0.3
0.2
0.1
0.0
−0.1
−0.2
−0.3
−0.4
0.3
0.2
0.1
0.0
−0.1
−0.2
−0.3
−0.4
−0.100
−0.105
−0.110
−0.115
−0.120
−0.125
−0.130

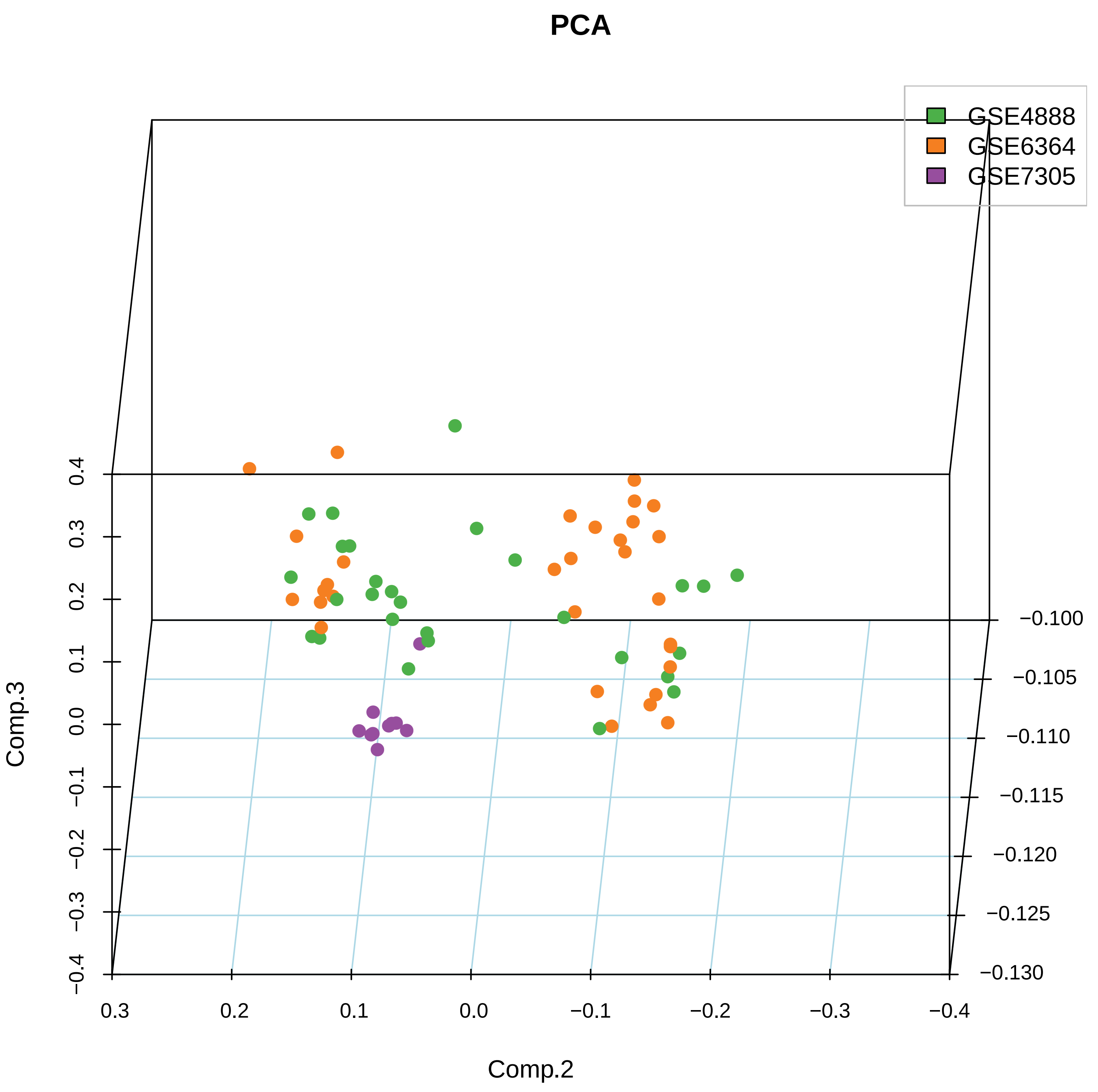
PCA
GSE4888
GSE6364
GSE7305
Comp.1
−0.100
−0.105
−0.110
−0.115
−0.120
−0.125
−0.130
Comp.2
0.3
0.2
0.1
0.0
−0.1
−0.2
−0.3
−0.4
Comp.3
0.4
0.3
0.2
0.1
0.0
−0.1
−0.2
−0.3
−0.4

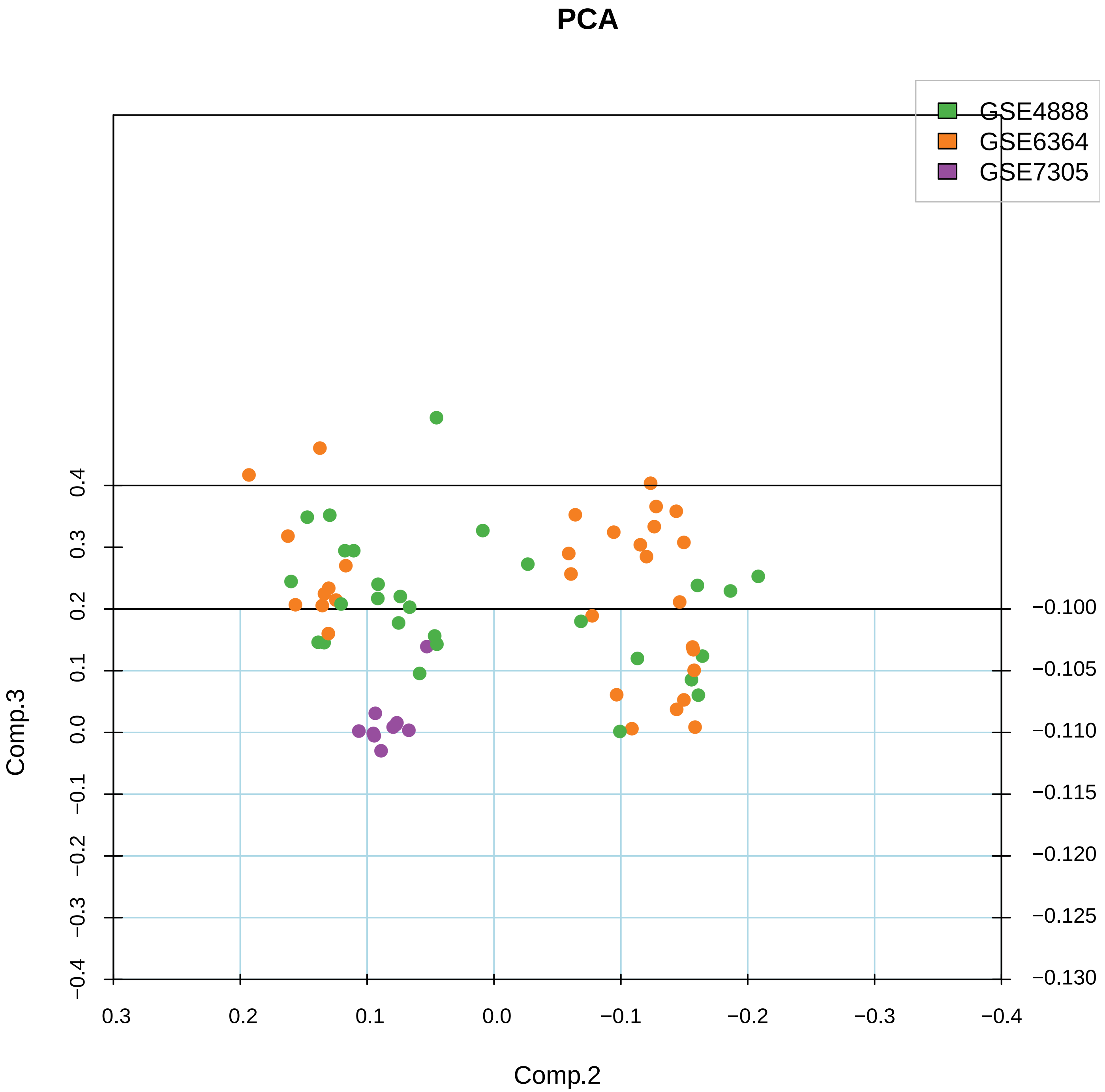
PCA
GSE4888
GSE6364
GSE7305
Comp.1
Comp.2
Comp.3
0.4
0.3
0.2
0.1
0.0
−0.1
−0.2
−0.3
−0.4
−0.100
−0.105
−0.110
−0.115
−0.120
−0.125
−0.130

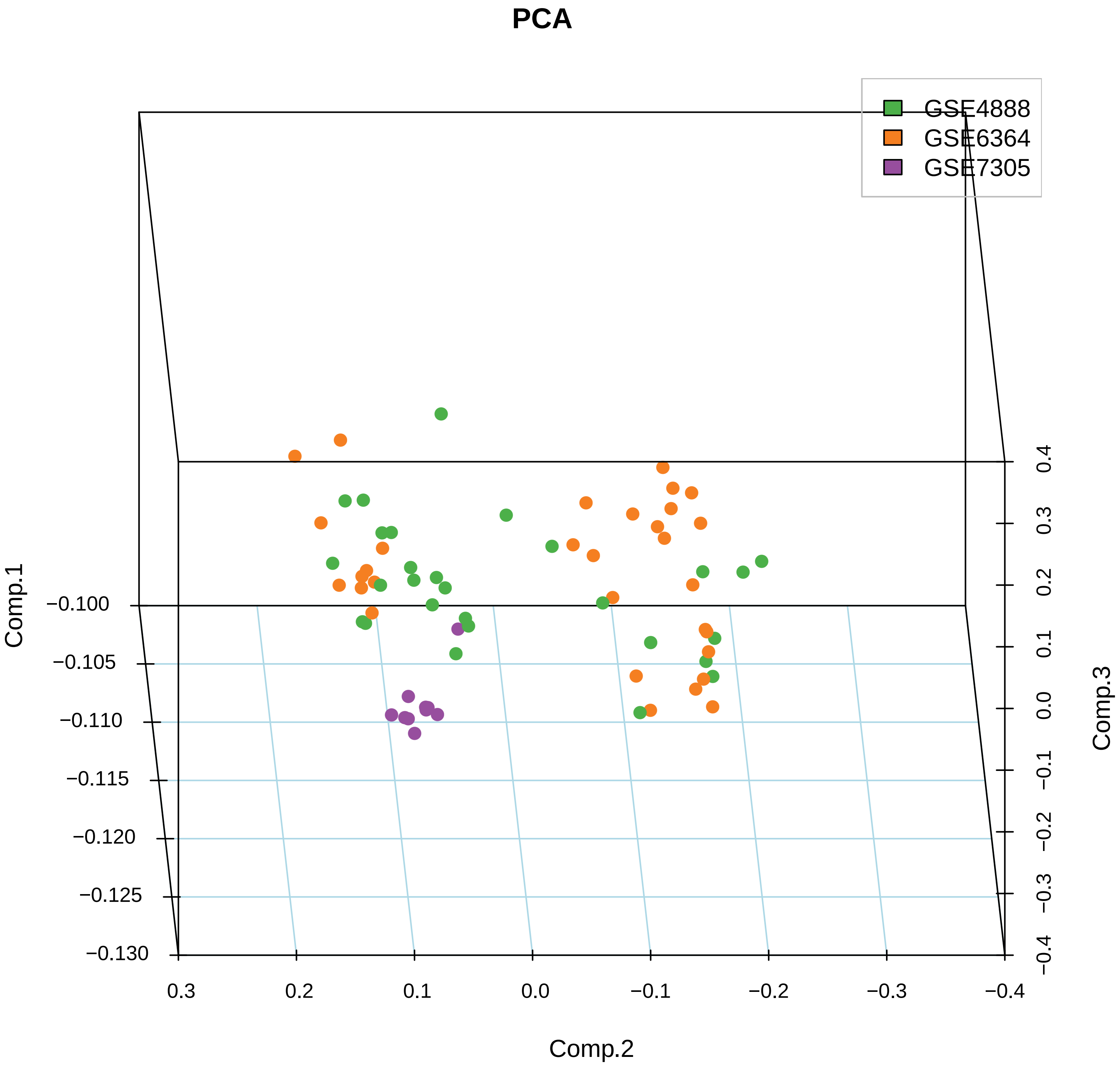
PCA
GSE4888
GSE6364
GSE7305
Comp.1
−0.100
−0.105
−0.110
−0.115
−0.120
−0.125
−0.130
Comp.2
0.3
0.2
0.1
0.0
−0.1
−0.2
−0.3
−0.4
Comp.3
0.4
0.3
0.2
0.1
0.0
−0.1
−0.2
−0.3
−0.4

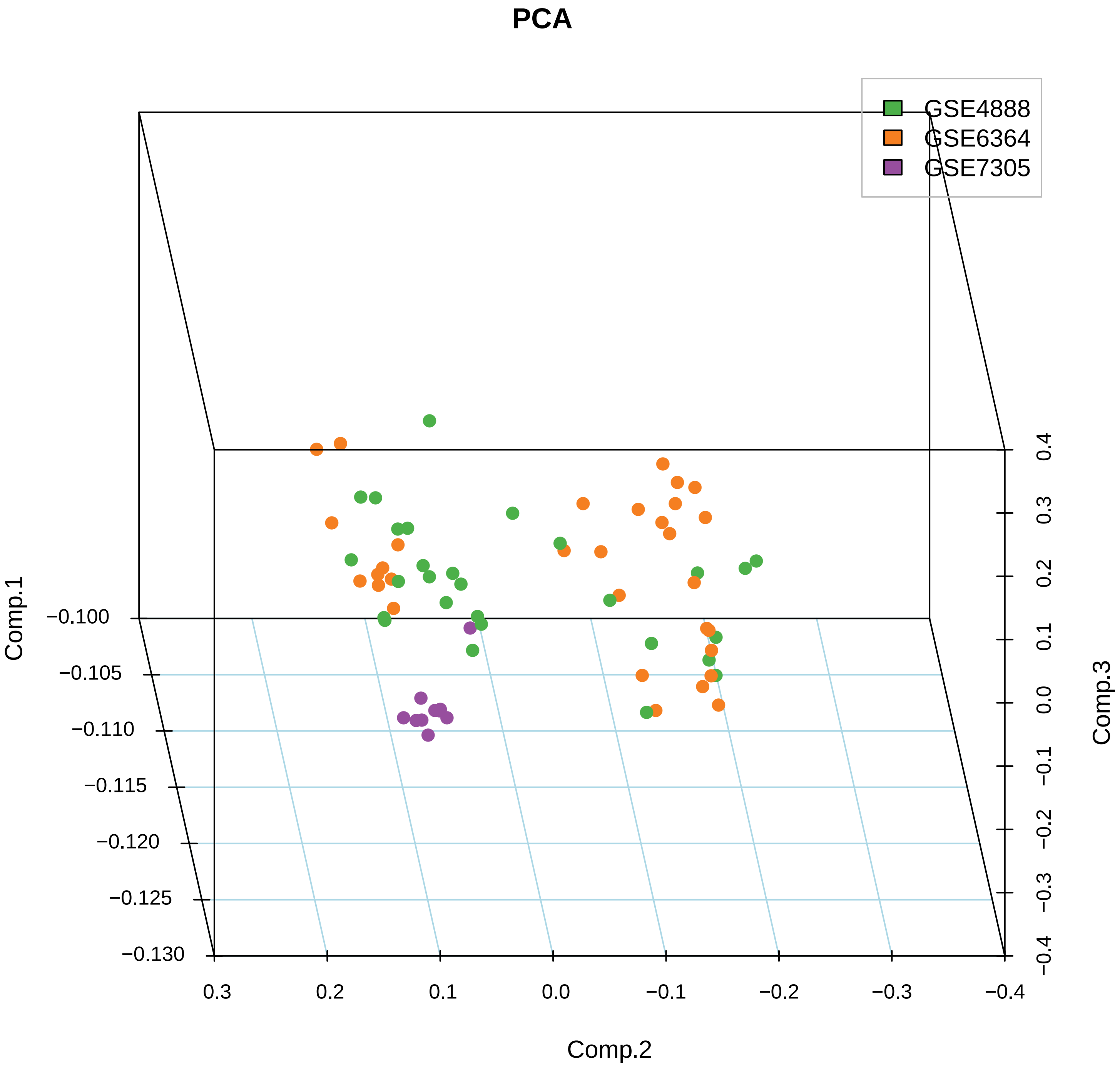
PCA
GSE4888
GSE6364
GSE7305
Comp.1
Comp.2
Comp.3
−0.100
−0.105
−0.110
−0.115
−0.120
−0.125
−0.130
0.3
0.2
0.1
0.0
−0.1
−0.2
−0.3
−0.4
0.4
0.3
0.2
0.1
0.0
−0.1
−0.2
−0.3
−0.4

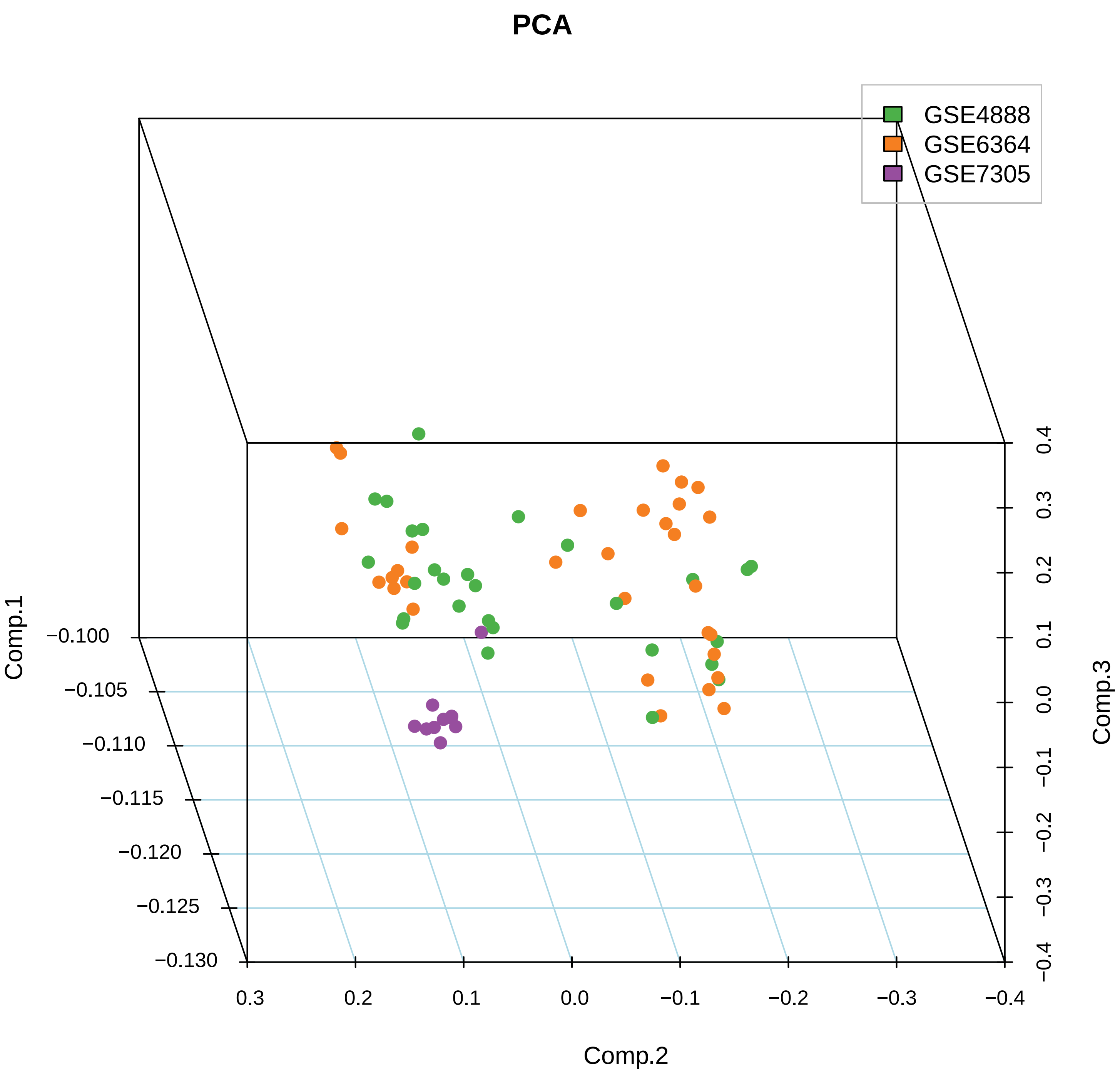
PCA
GSE4888
GSE6364
GSE7305
Comp.1
−0.100
−0.105
−0.110
−0.115
−0.120
−0.125
−0.130
0.3
0.2
0.1
0.0
−0.1
−0.2
−0.3
−0.4
Comp.2
0.4
0.3
0.2
0.1
0.0
−0.1
−0.2
−0.3
−0.4
Comp.3

PCA

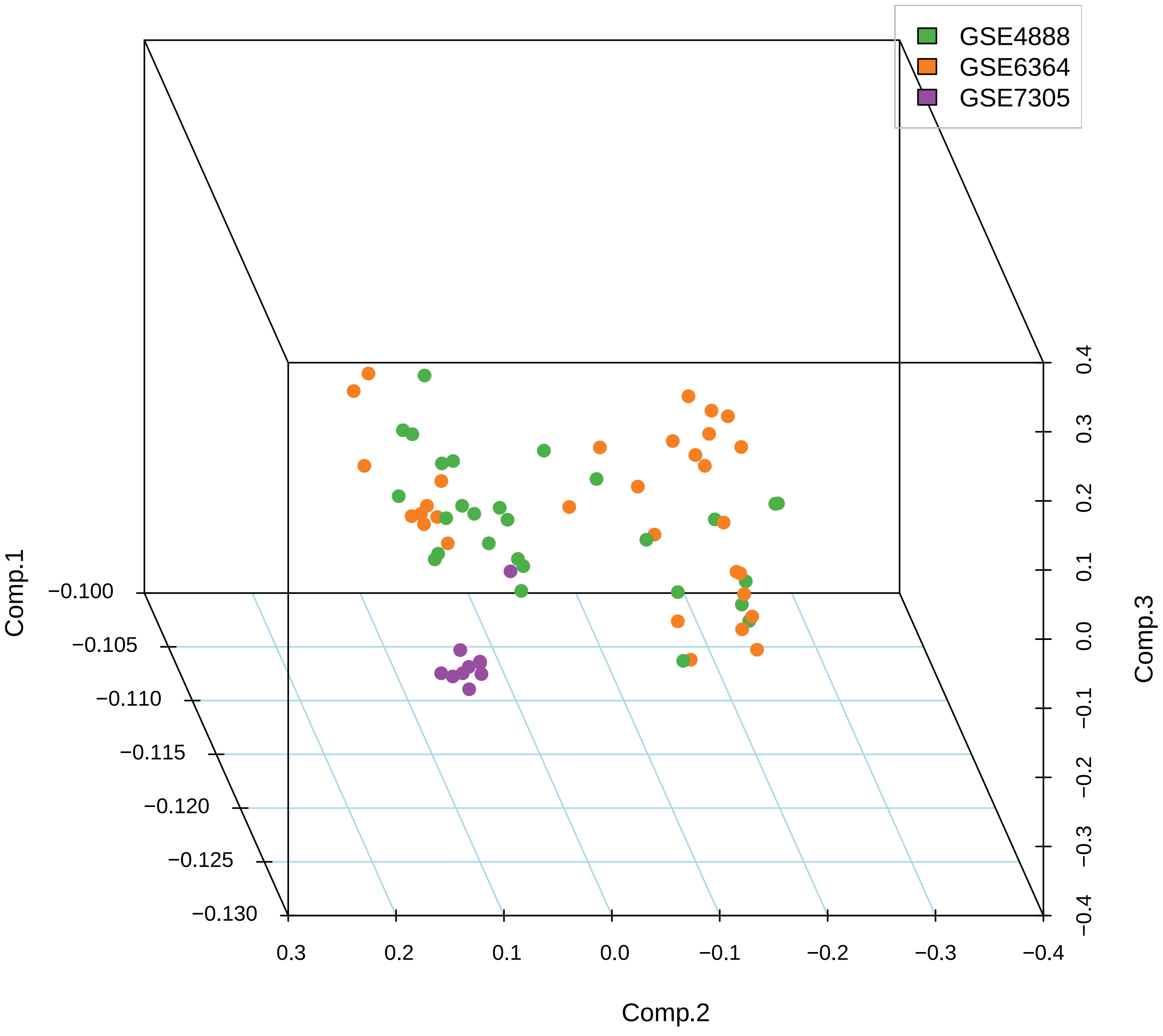

GSE4888
GSE6364
GSE7305
Comp.1
−0.100
−0.105
−0.110
−0.115
−0.120
−0.125
−0.130
0.3
0.2
0.1
0.0
−0.1
−0.2
−0.3
−0.4
Comp.2
0.4
0.3
0.2
0.1
0.0
−0.1
−0.2
−0.3
−0.4
Comp.3

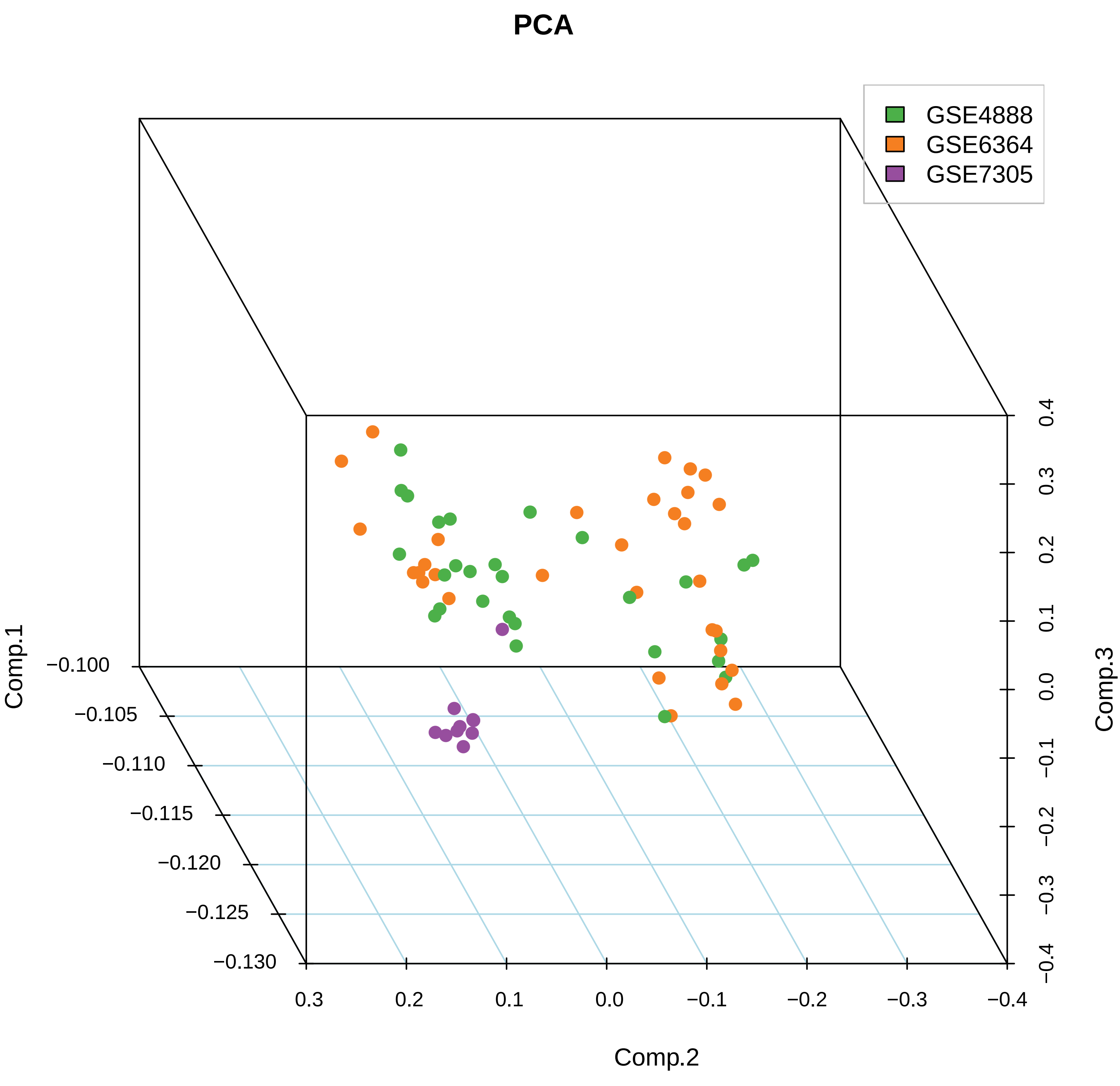
PCA
GSE4888
GSE6364
GSE7305
Comp.1
−0.100
−0.105
−0.110
−0.115
−0.120
−0.125
−0.130
Comp.2
0.3
0.2
0.1
0.0
−0.1
−0.2
−0.3
−0.4
Comp.3
0.4
0.3
0.2
0.1
0.0
−0.1
−0.2
−0.3
−0.4

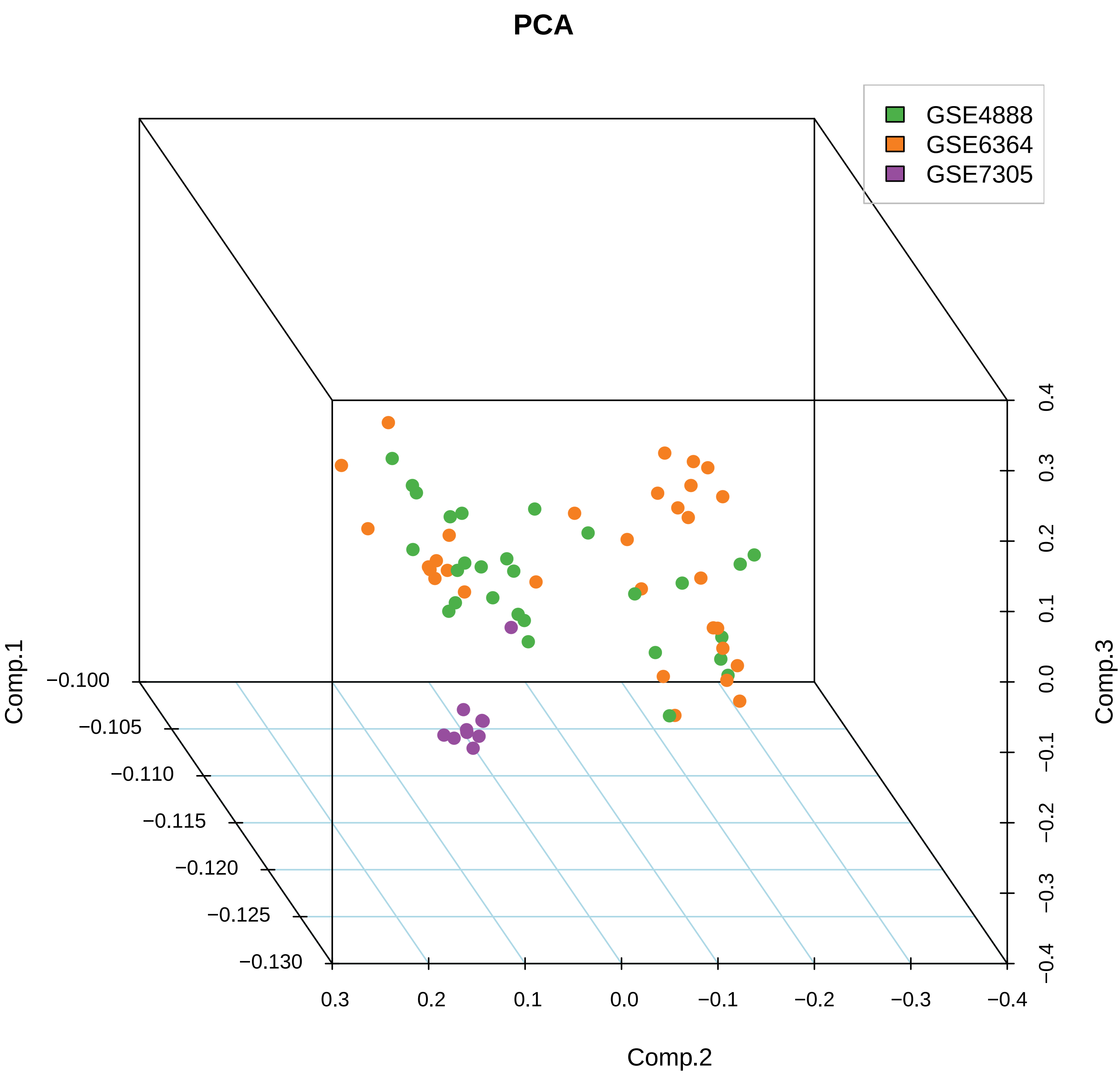

PCA
GSE4888
GSE6364
GSE7305
Comp.1
Comp.2
Comp.3
−0.100
−0.105
−0.110
−0.115
−0.120
−0.125
−0.130
0.3
0.2
0.1
0.0
−0.1
−0.2
−0.3
−0.4
0.4
0.3
0.2
0.1
0.0
−0.1
−0.2
−0.3
−0.4

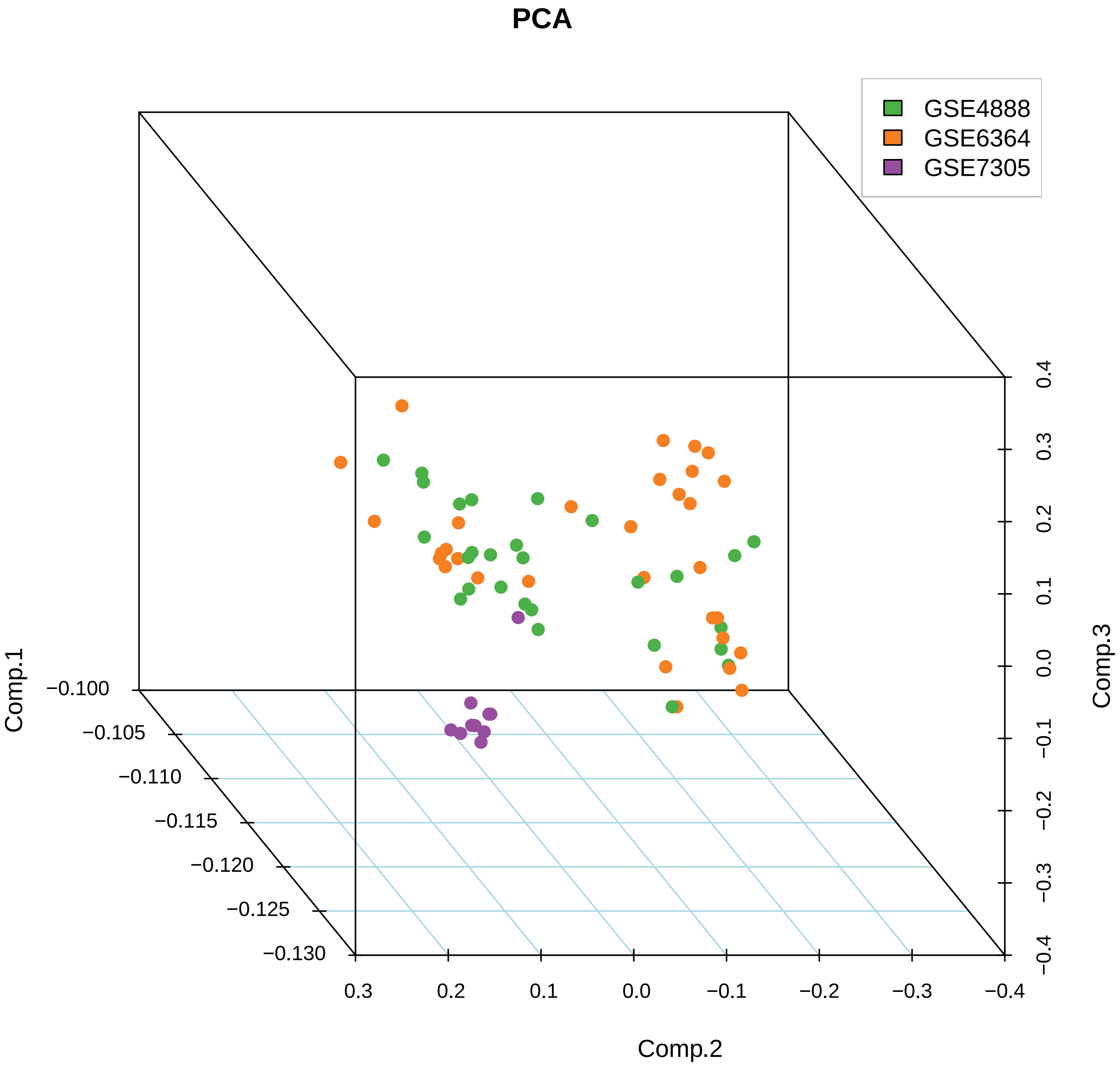

PCA
GSE4888
GSE6364
GSE7305
Comp.1
Comp.2
Comp.3
−0.100
−0.105
−0.110
−0.115
−0.120
−0.125
−0.130
0.3
0.2
0.1
0.0
−0.1
−0.2
−0.3
−0.4
0.4
0.3
0.2
0.1
0.0
−0.1
−0.2
−0.3
−0.4

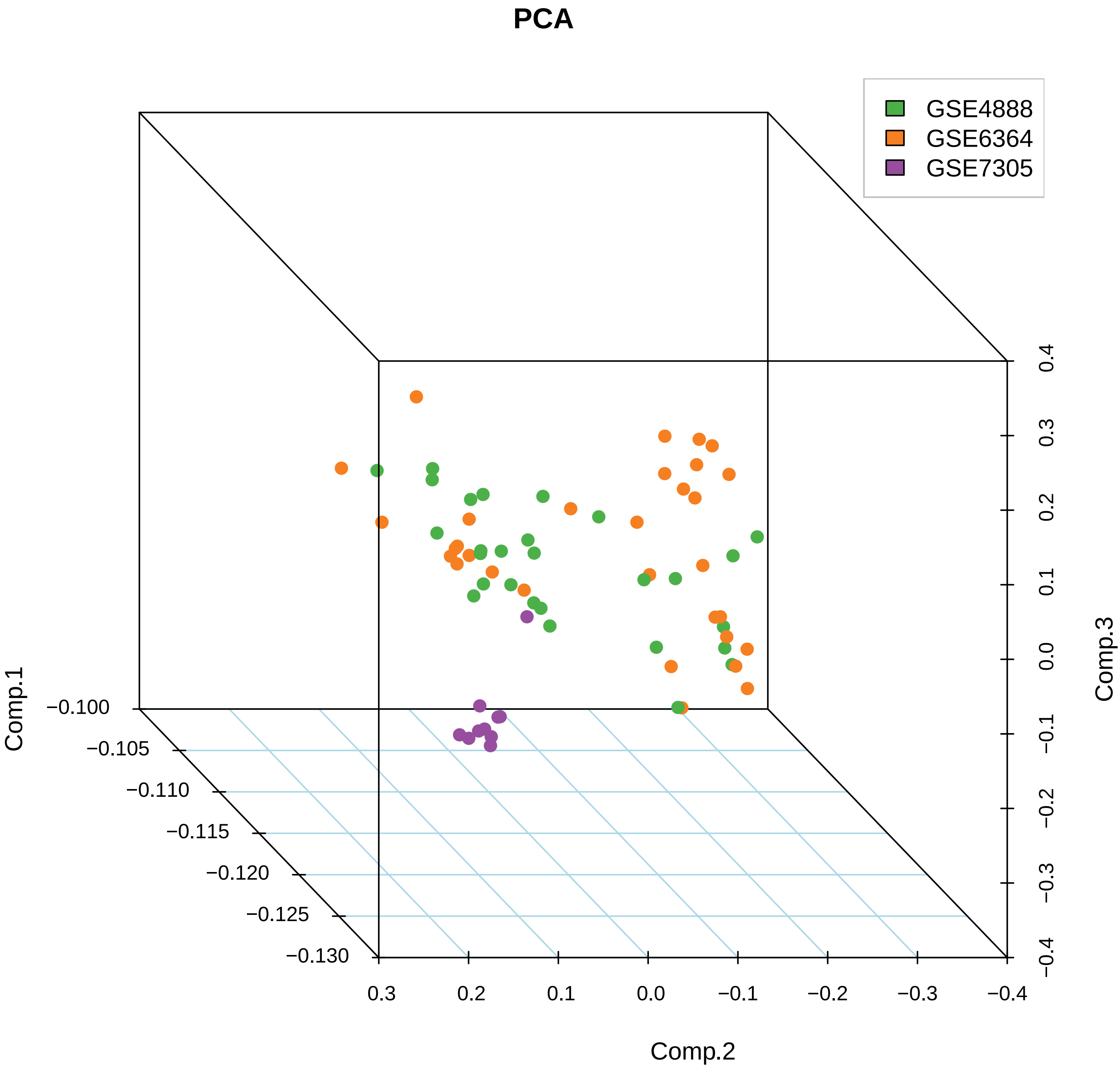
PCA
GSE4888
GSE6364
GSE7305
Comp.1
−0.100
−0.105
−0.110
−0.115
−0.120
−0.125
−0.130
0.3
0.2
0.1
0.0
−0.1
−0.2
−0.3
−0.4
Comp.2
0.4
0.3
0.2
0.1
0.0
−0.1
−0.2
−0.3
−0.4
Comp.3

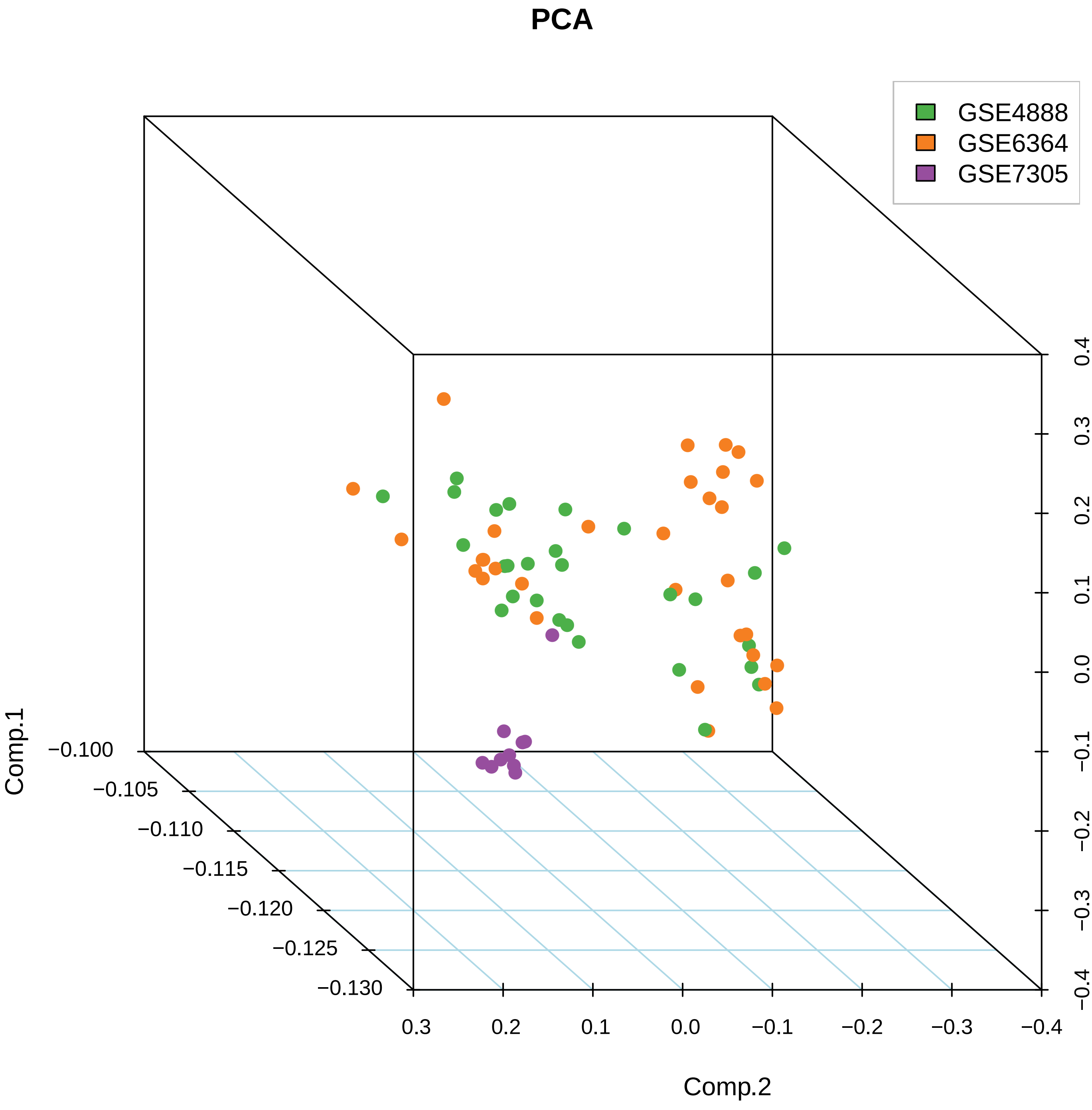
PCA
GSE4888
GSE6364
GSE7305
Comp.1
Comp.2
Comp.3
−0.100
−0.105
−0.110
−0.115
−0.120
−0.125
−0.130
0.3
0.2
0.1
0.0
−0.1
−0.2
−0.3
−0.4
0.4
0.3
0.2
0.1
0.0
−0.1
−0.2
−0.3
−0.4

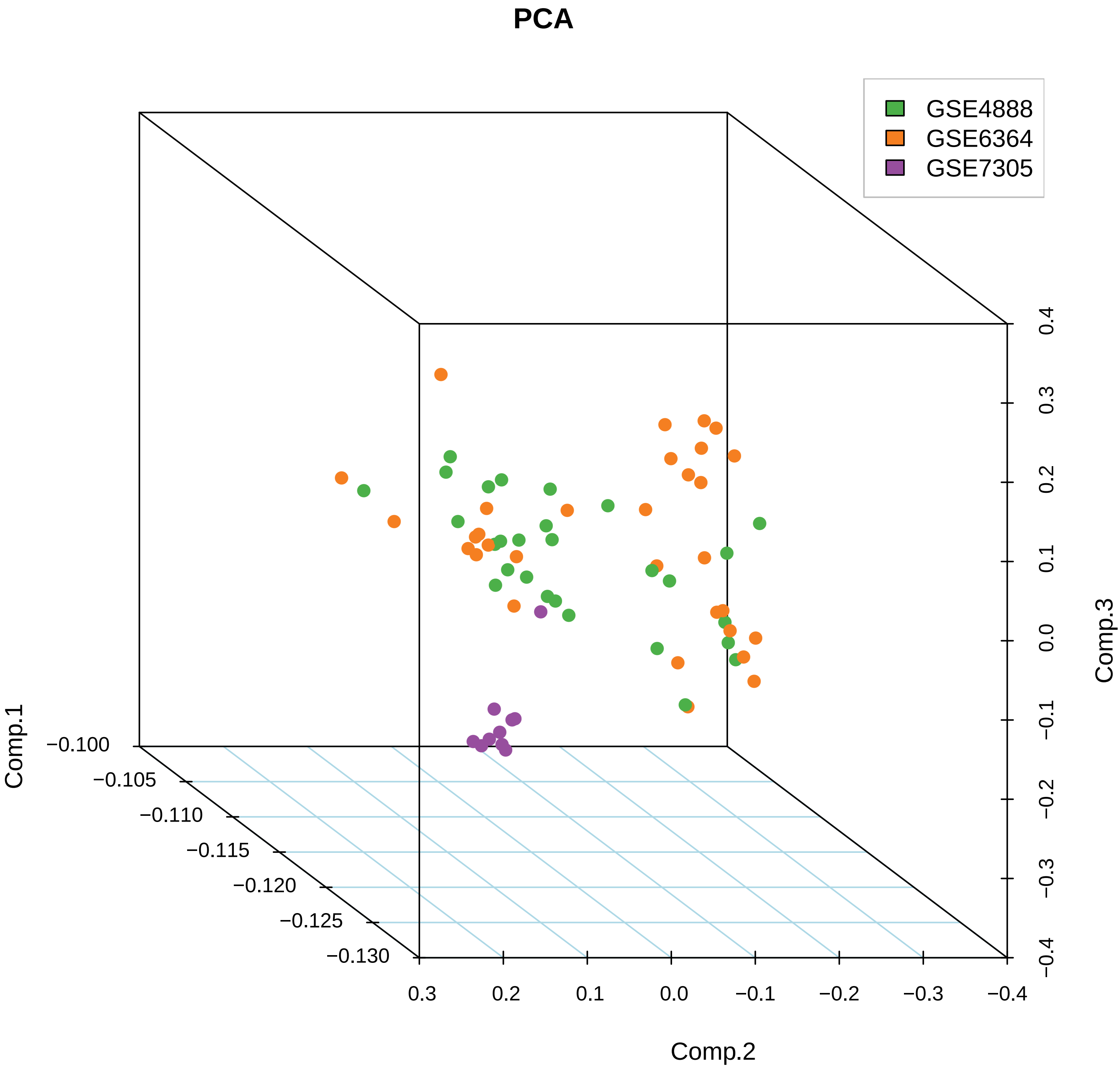
PCA
GSE4888
GSE6364
GSE7305
Comp.1
Comp.2
Comp.3
−0.100
−0.105
−0.110
−0.115
−0.120
−0.125
−0.130
0.3
0.2
0.1
0.0
−0.1
−0.2
−0.3
−0.4
0.4

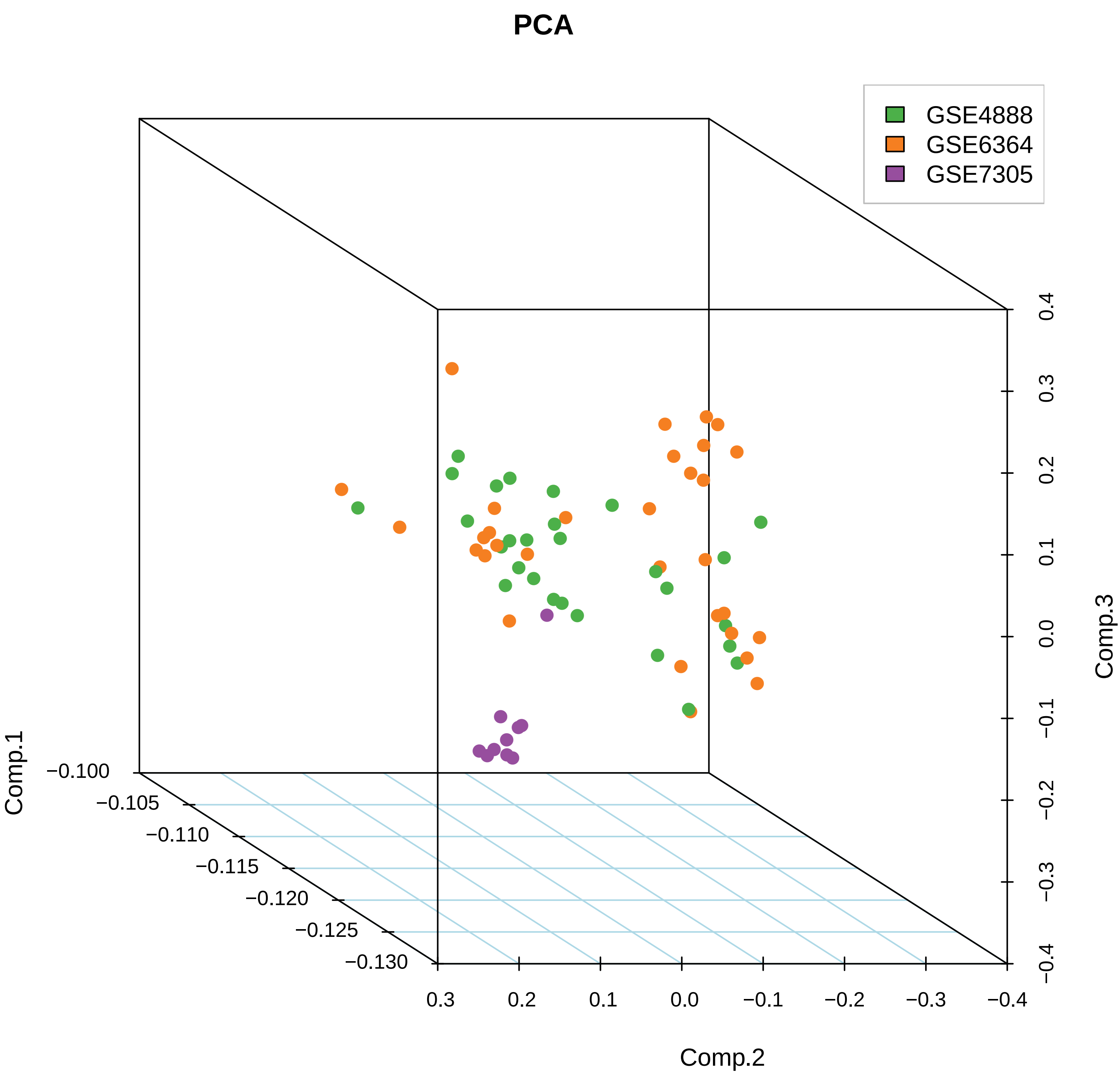
PCA
GSE4888
GSE6364
GSE7305
Comp.1
Comp.2
Comp.3
−0.100
−0.105
−0.110
−0.115
−0.120
−0.125
−0.130
0.3
0.2
0.1
0.0
−0.1
−0.2
−0.3
−0.4
0.4
0.3
0.2
0.1
0.0
−0.1
−0.2
−0.3
−0.4

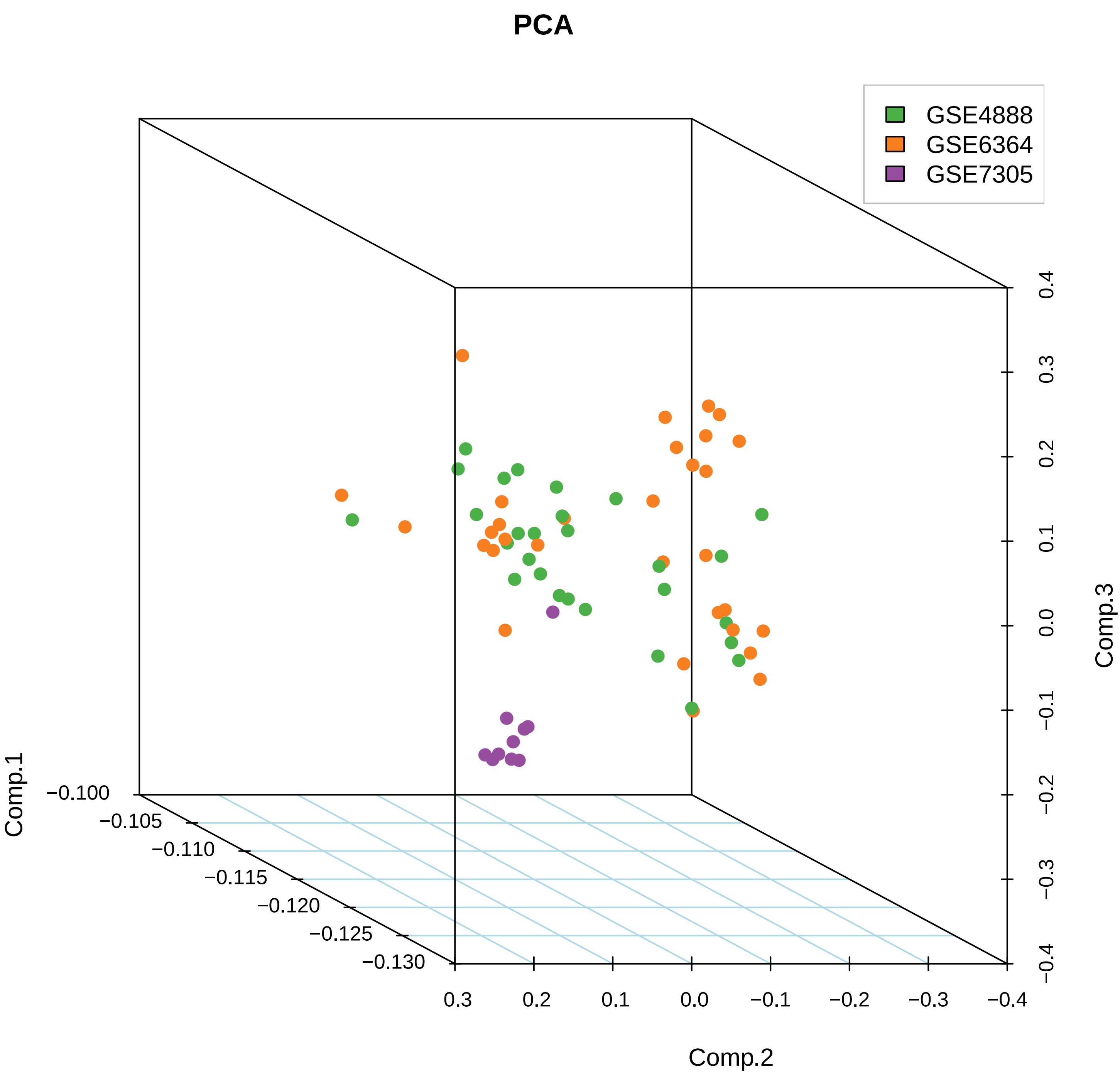

PCA
GSE4888
GSE6364
GSE7305
Comp.1
Comp.2
Comp.3
−0.100
−0.105
−0.110
−0.115
−0.120
−0.125
−0.130
0.3
0.2
0.1
0.0
−0.1
−0.2
−0.3
−0.4
0.4
0.3
0.2
0.1
0.0
−0.1
−0.2
−0.3
−0.4

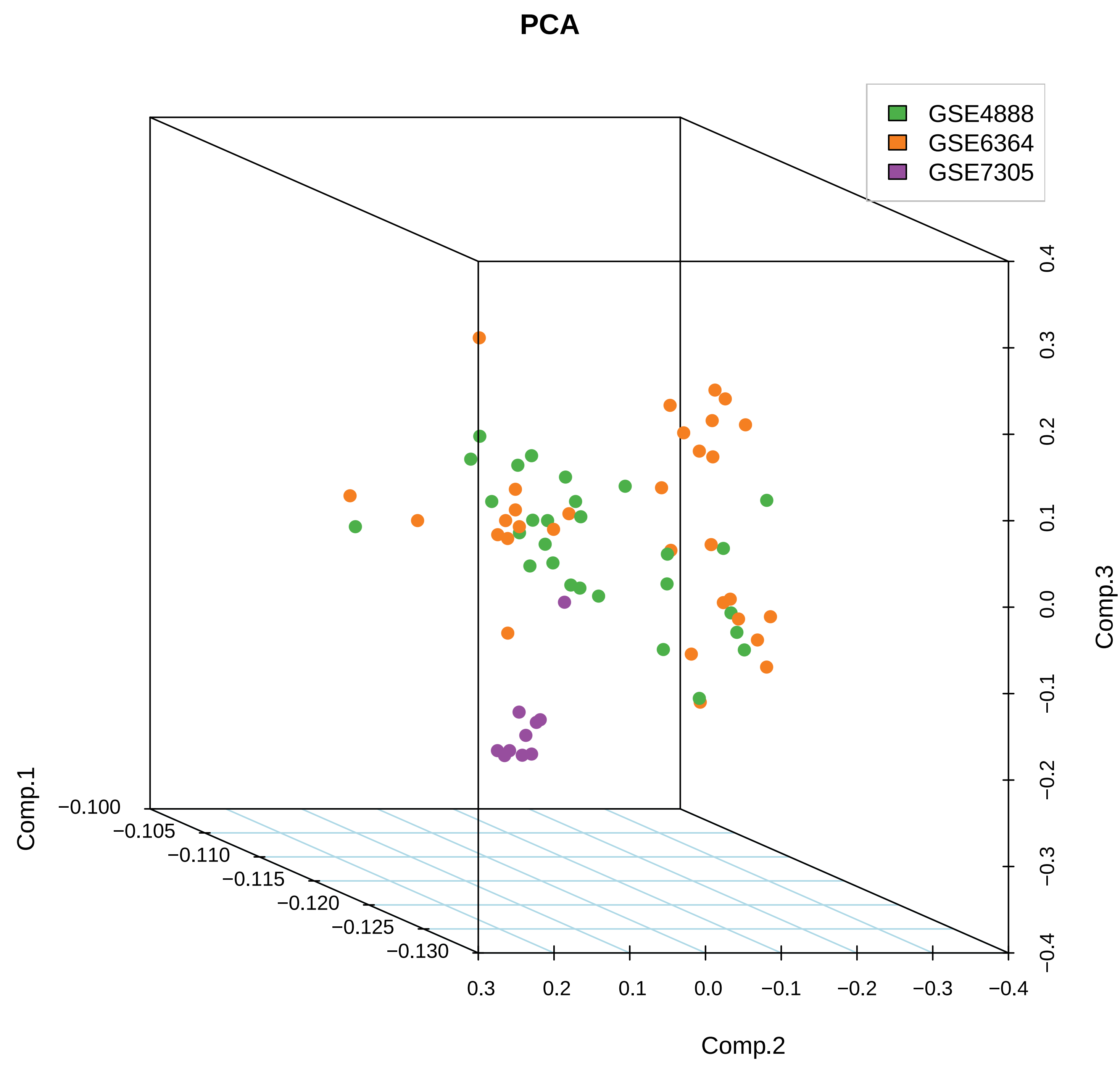

PCA
GSE4888
GSE6364
GSE7305
Comp.1
Comp.2
Comp.3
−0.100
−0.105
−0.110
−0.115
−0.120
−0.125
−0.130
0.3
0.2
0.1
0.0
−0.1
−0.2
−0.3
−0.4
0.4
0.3
0.2
0.1
0.0
−0.1
−0.2
−0.3
−0.4

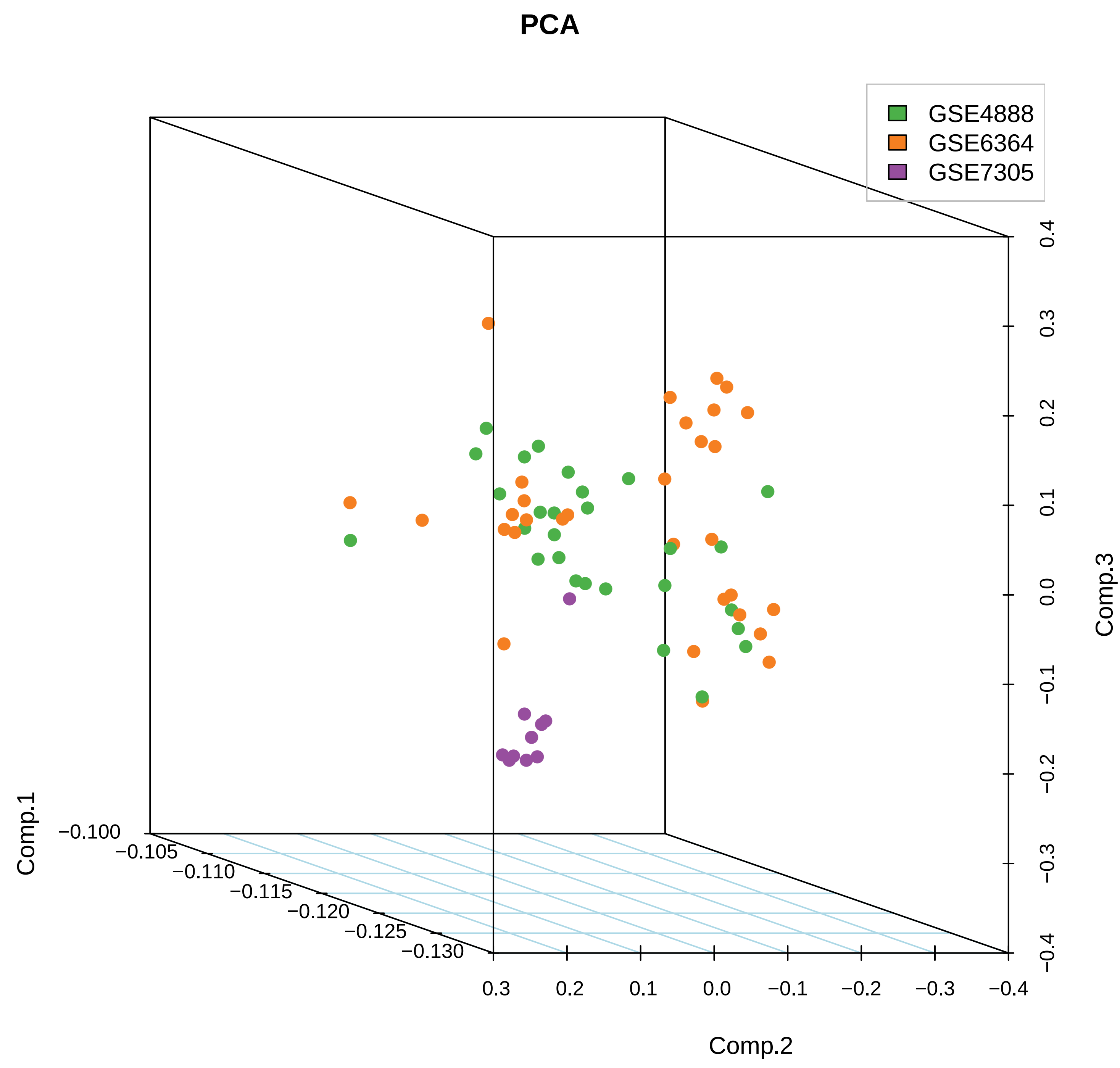

PCA
GSE4888
GSE6364
GSE7305
Comp.1
Comp.2
Comp.3
−0.100
−0.105
−0.110
−0.115
−0.120
−0.125
−0.130
0.3
0.2
0.1
0.0
−0.1
−0.2
−0.3
−0.4
0.4

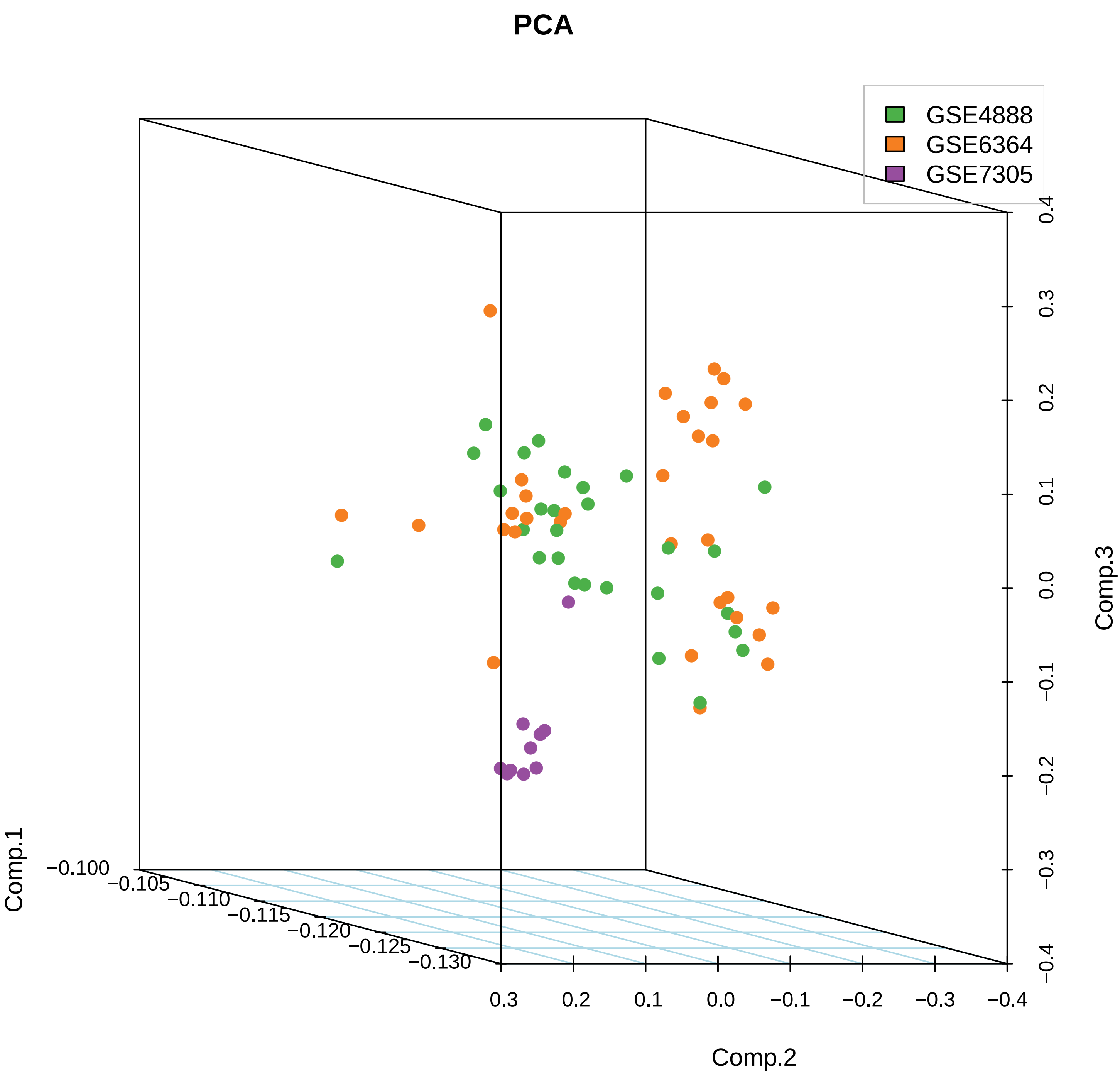
PCA
GSE4888
GSE6364
GSE7305
Comp.1
Comp.2
Comp.3
−0.100
−0.105
−0.110
−0.115
−0.120
−0.125
−0.130
0.3
0.2
0.1
0.0
−0.1
−0.2
−0.3
−0.4
0.4
0.3
0.2
0.1
0.0
−0.1
−0.2
−0.3
−0.4

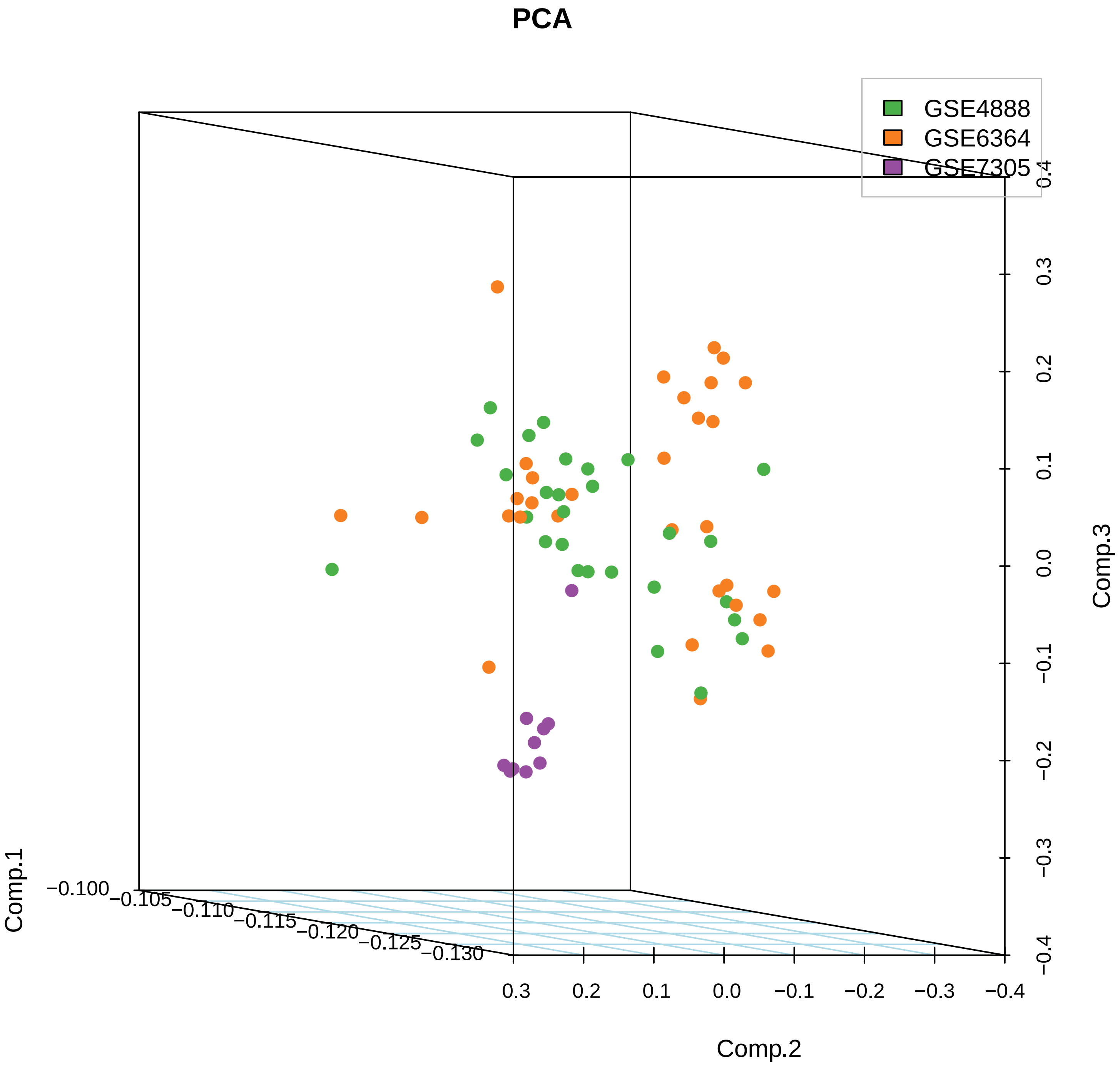
PCA
GSE4888
GSE6364
GSE7305
Comp.1
−0.100
−0.105
−0.110
−0.115
−0.120
−0.125
−0.130
Comp.2
0.3
0.2
0.1
0.0
−0.1
−0.2
−0.3
−0.4
Comp.3
0.4
0.3
0.2
0.1
0.0
−0.1
−0.2
−0.3
−0.4

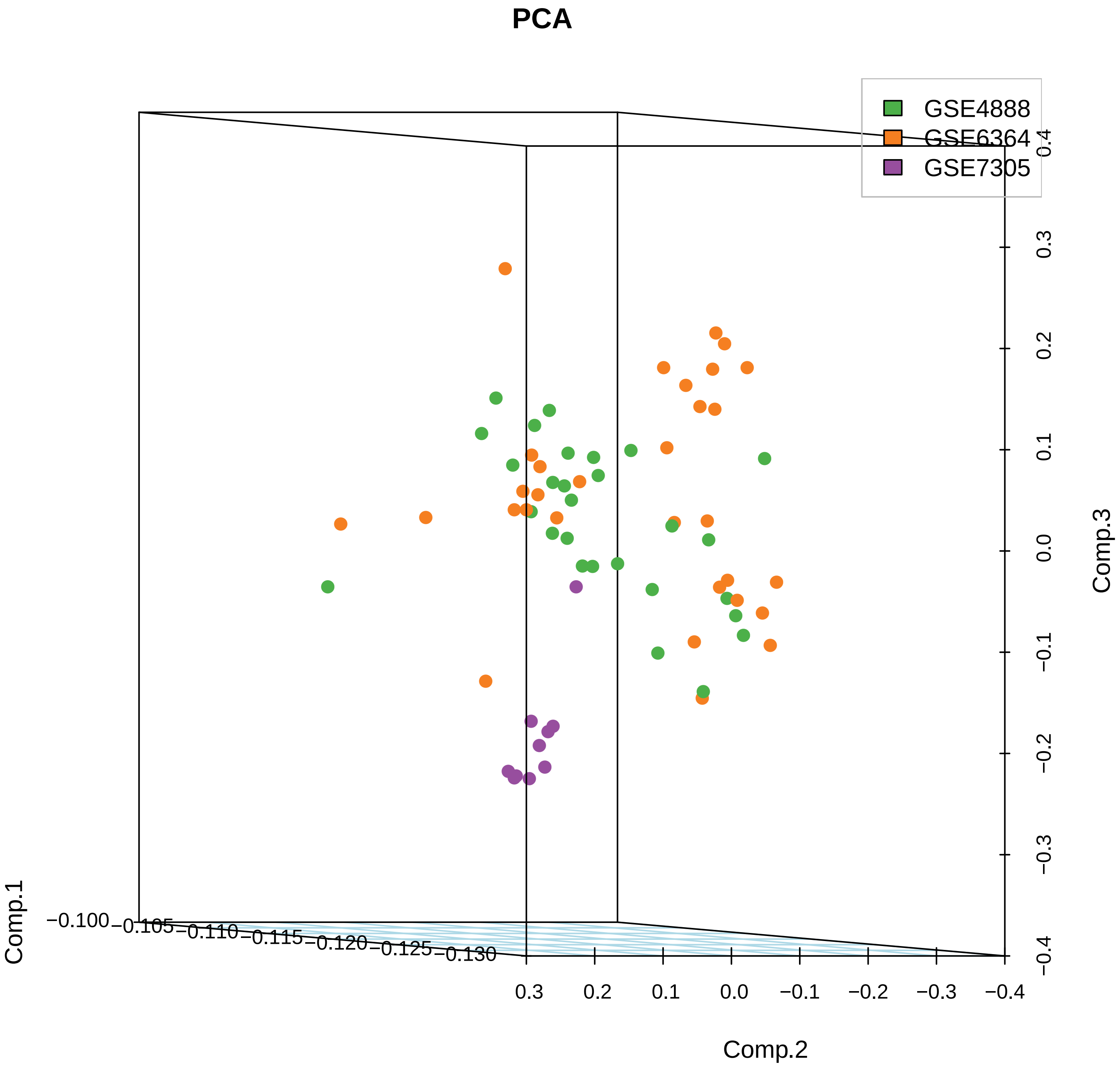

PCA
GSE4888
GSE6364
GSE7305
Comp.1
Comp.2
Comp.3
−0.100
−0.105
−0.110
−0.115
−0.120
−0.125
−0.130
0.3
0.2
0.1
0.0
−0.1
−0.2
−0.3
−0.4
0.4

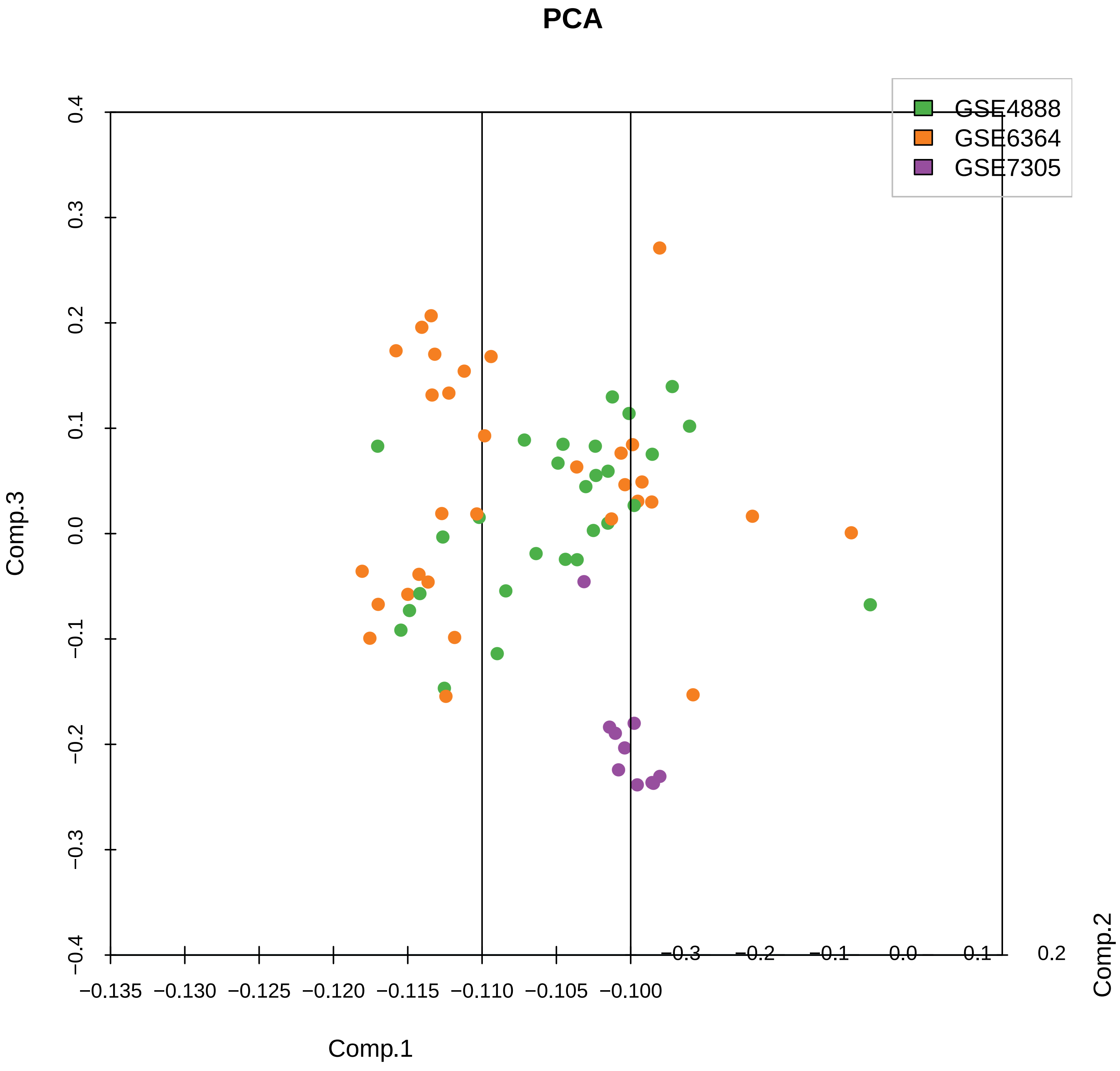
PCA
GSE4888
GSE6364
GSE7305
Comp.3
Comp.1
Comp.2
0.4
0.3
0.2
0.1
0.0
−0.1
−0.2
−0.3
−0.4
−0.135
−0.130
−0.125
−0.120
−0.115
−0.110
−0.105
−0.100
−0.3
−0.2
−0.1
0.0
0.1
0.2
