# Supplementary figures and images for: Identification of key genes and pathways in endometriosis by integrated expression profiles analysis
Source: PeerJ. 2020 Dec 7;8:e10171. doi: 10.7717/peerj.10171 (PMC7727381; doi:10.7717/peerj.10171)

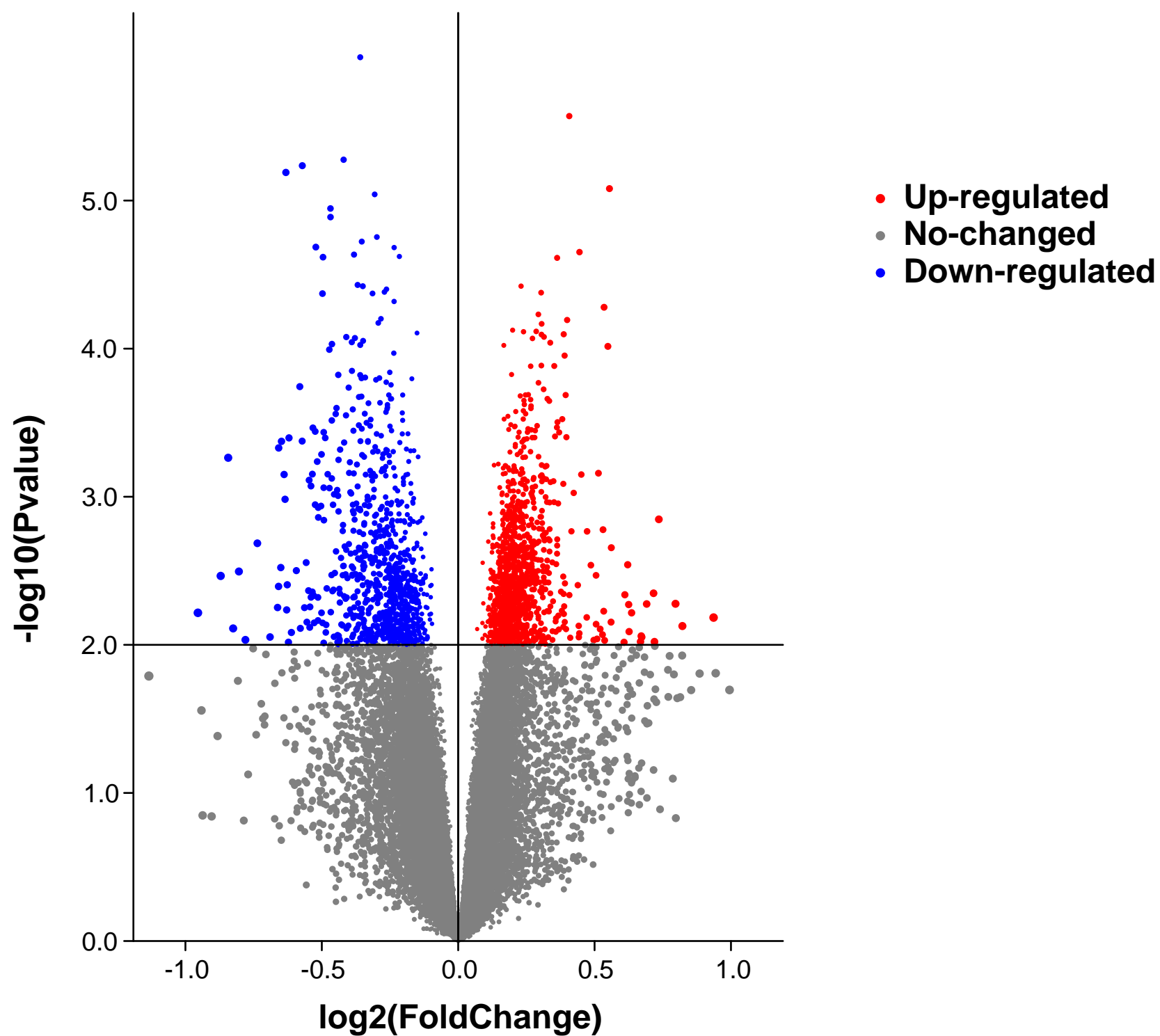

Supplement: Supplemental Information 4 [file peerj-08-10171-s004.pdf]

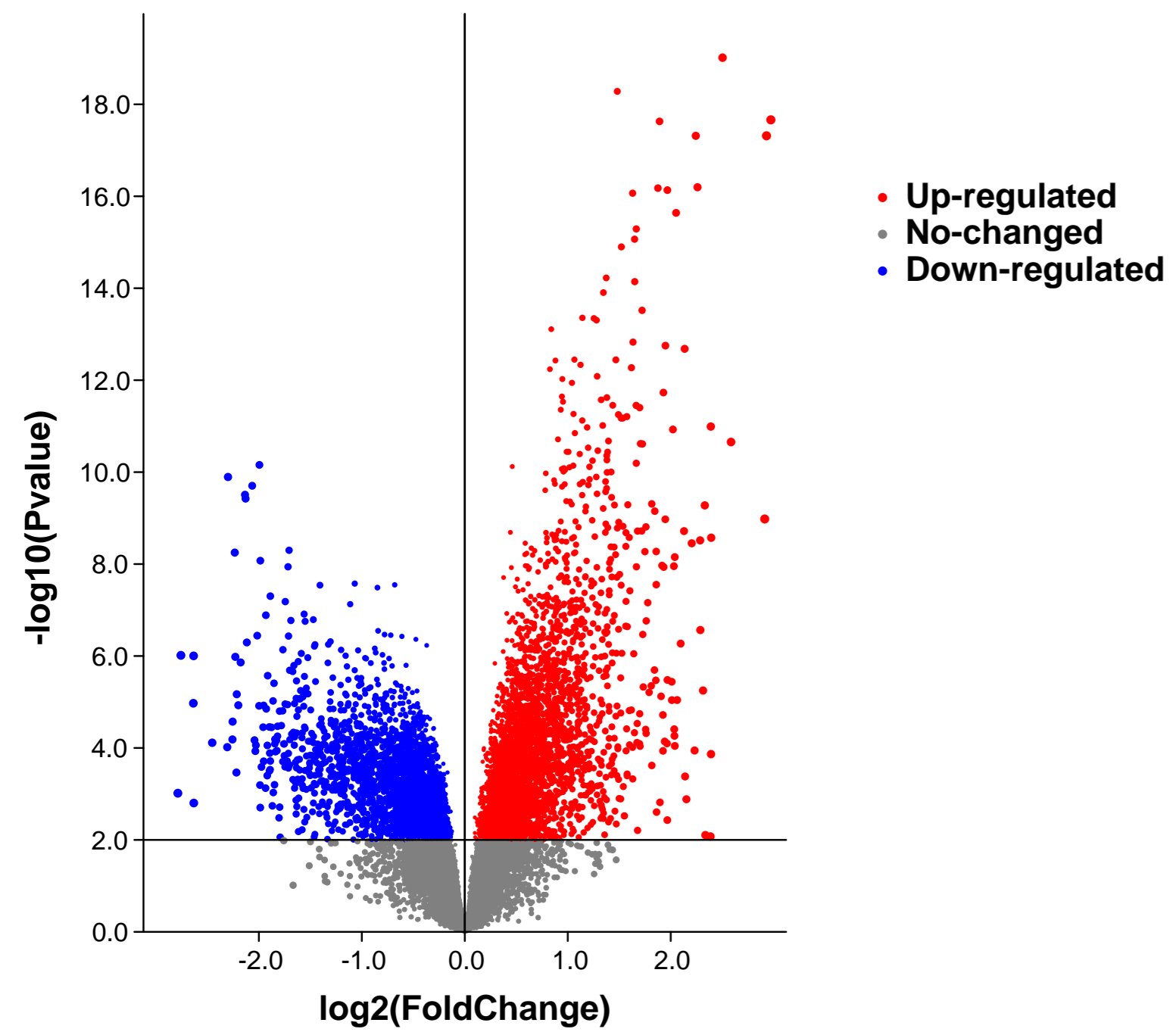

Supplement: Supplemental Information 5 [file peerj-08-10171-s005.pdf]

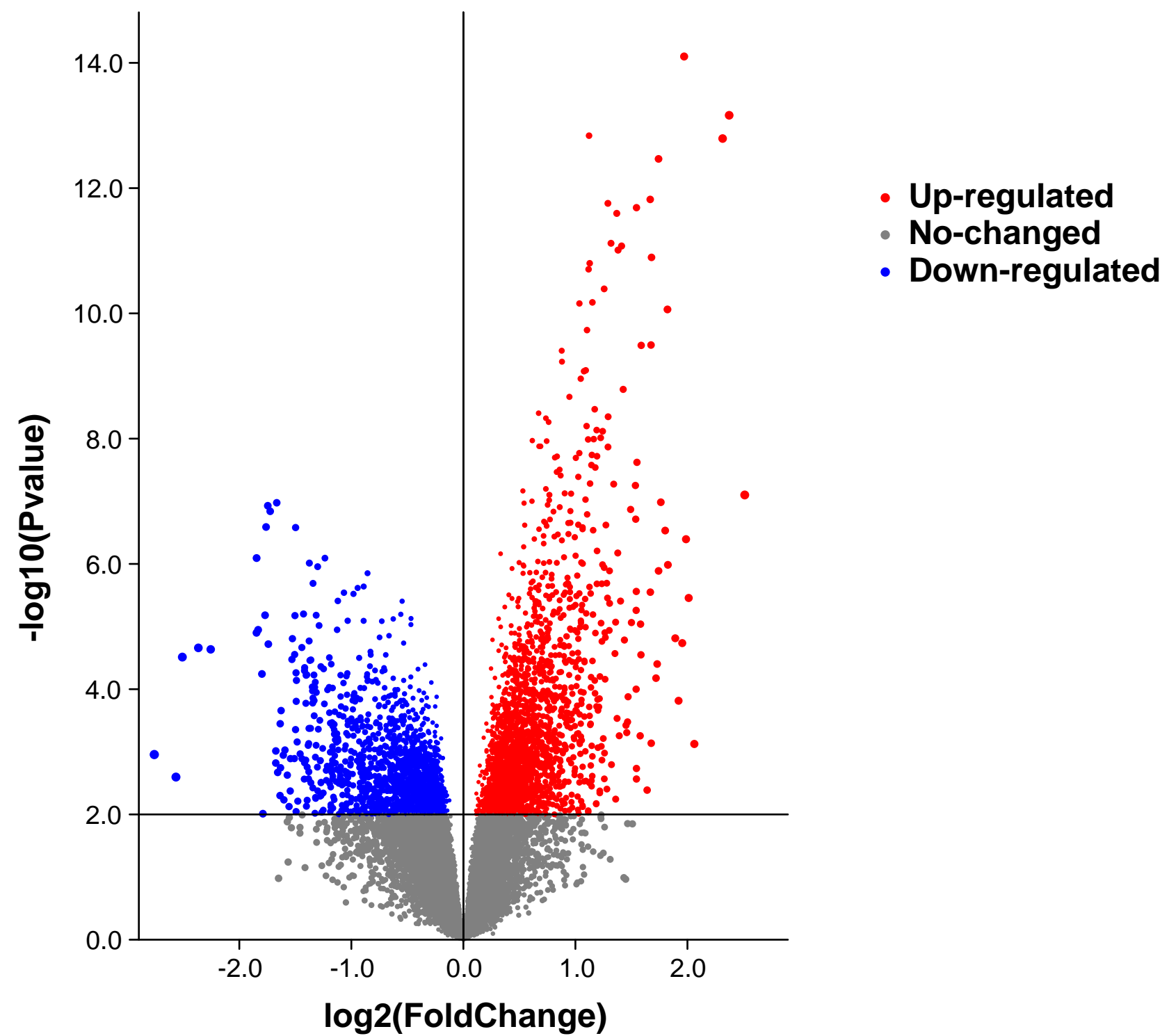

Supplement: Supplemental Information 6 [file peerj-08-10171-s006.pdf]
